# Longitudinal digital phenotyping of activity rhythms and biological aging using commercial wearables

**DOI:** 10.1038/s41467-026-76147-6

**Published:** 2026-08-22

**Authors:** Jinjoo Shim, Jukka-Pekka Onnela

**Affiliations:** Department of Biostatistics, Harvard T.H. Chan School of Public Health, Boston, MA USA

**Keywords:** Epidemiology, Public health, Disease prevention

## Abstract

Disrupted rest-activity rhythms have been associated with aging and chronic disease, yet longitudinal evidence from free-living populations has been lacking. Here we integrate multi-year Fitbit activity data from the *All of Us* Research Program with clinical biomarker-derived PhenoAge from 2,222 participants (8,447 person-years). Through high-dimensional digital phenotyping, we show that circadian rest-activity rhythm intensity, timing, and stability are associated with biological aging trajectories. Higher rhythm intensity was associated with 26–46% lower odds of accelerated aging. Associations of timing and regularity were stronger in females. In males, accelerated aging followed a biphasic instability pattern with early-morning surges and late-evening rebounds. These findings provide large-scale longitudinal evidence that consumer wearable-derived rest-activity rhythms may serve as digital biomarkers of aging trajectories. By linking population-scale digital phenotyping to biological aging, this work highlights the potential of scalable digital measures for aging-related risk assessment and future healthy-aging research.

## Abstract (web summary)

Long-term links between free-living rest-activity rhythms and biological aging remain unclear. Here, the authors show that Fitbit-derived rest-activity rhythms are associated with biological aging trajectories in All of Us Research Program.

## Introduction

Circadian rhythms are fundamental biological processes that regulate sleep-wake cycles, metabolism, cognition, and immune function^1–4^. They align internal physiology with the external environment by shaping the amplitude and timing of daily functions to optimize health and survival. With aging, circadian organization deteriorates, manifested as dampened amplitude, increased fragmentation, and phase shifts^5,6^. While these changes are largely driven by intrinsic impairments in rhythmicity that perturb cellular functions and hormone regulation, circadian disruption may also be driven by extrinsic stressors such as shift work, social jetlag, nighttime light exposure, and irregular activity schedules^7,8^. Thus, both intrinsic aging processes and extrinsic challenges progressively undermine circadian integrity, with downstream consequences for health and longevity.

Accumulating evidence links circadian disruption to a broad spectrum of adverse outcomes, including metabolic dysfunction, cardiovascular disease, neurodegeneration, mental illness, frailty, and elevated mortality risk^2,9–14^. More recently, circadian rest-activity rhythm disruption has been recognized as a correlate of accelerated biological aging, a measure of physiological and functional state that more accurately predicts morbidity and mortality than chronological age^15–18^. Cross-sectional studies consistently demonstrate that attenuated amplitude, delayed acrophase, and irregular or fragmented activity rhythms are associated with reduced lifespan and healthspan. Yet, most evidence comes from single, short-duration activity recordings, and the longitudinal associations between rest-activity rhythmicity and biological aging trajectories remain unresolved.

This limitation reflects methodological constraints. Conventional circadian assessments, such as melatonin profiling, require repeated invasive, labor-intensive sampling and are impractical for large-scale studies^19^. As an alternative, research-grade actigraphy has been implemented in large cohort studies to characterize rest-activity rhythms, sleep-wake patterns, and physical activity in a non-invasive and continuous manner^15,20,21^. However, a majority of studies using wearable actigraphy rely on 7-day recordings, providing only a limited snapshot of activity patterns. Such short assessments are also highly susceptible to transient influences such as acute illness, travel, environmental factors, or seasonal variation^22^. These constraints hinder the identification of persistent rest-activity rhythm features that could serve as robust and modifiable biomarkers of biological aging.

Recent advances in digital phenotyping and widespread adoption of consumer-grade digital devices now offer a way forward^23,24^. Wearables and smartphones allow passive, continuous, and long-term monitoring of rest-activity rhythms, sleep, and activity in free-living settings, enabling scalable longitudinal characterization^25,26^. This creates a unique opportunity to integrate repeated measures of biological age assessments with digital phenotyping of real-world rest-activity rhythm dynamics, thereby capturing persistent rest-activity rhythm features that short-term actigraphy is limited in detecting^27,28^.

To this end, we leveraged the *All of Us* Research Program, a large nationwide prospective cohort in the United States integrating multi-year, multi-modal data to examine the relationship between longitudinal wearable-based activity rhythms and biological age acceleration. Using Fitbit activity data, we extracted circadian rest-activity rhythm metrics over time and applied data-driven functional principal component analysis to characterize latent 24 h rhythm profiles. By linking these longitudinal activity rhythm profiles with repeated measures of biological age, quantified using the clinical biomarker-based phenotypic age (PhenoAge)^29^, we aim to characterize biological aging trajectories. PhenoAge is calculated based on a mortality prediction score derived from nine clinical biomarkers (albumin, alkaline phosphatase, creatinine, C-reactive protein (CRP), glucose, mean cell volume, red cell distribution width, white blood cell count, and lymphocyte percentage) and chronological age, reflecting the age at which the average NHANES III mortality risk matches the predicted mortality risk^30^. It has been validated across multi-ethnic cohorts to predict disease, physical functioning, cognitive performance, and mortality, and longitudinal changes have also been reported to provide prognostic information beyond baseline levels^30–35^. Further supporting the use of PhenoAge as a longitudinal phenotypic marker, recent evidence demonstrates that within-individual changes in a PhenoAge estimate derived from a reduced biomarker set are systematically associated with modifiable lifestyle and environmental factors^36^. Our findings provide large-scale longitudinal evidence that wearable-derived rest-activity rhythms are robustly associated with trajectories of biological aging and support the potential utility of rest-activity measures as scalable and accessible digital biomarkers of biological aging trajectories.

## Results

### Baseline characteristics

Table 1 describes the baseline characteristics of the study cohort (*N* = 2222), stratified by biological aging status. Of the participants, 1021 (45.9%) were classified as fast agers and 1201 (54.1%) as slow agers at baseline. Chronological age did not differ significantly between the groups (61.09 years, SD 13.82 vs. 60.23 years, SD 14.17; two-sample *t* test of Δ≠0: *p* = 0.147), whereas biological age was significantly higher in fast agers (66.43 years, SD 14.57) compared with slow agers (54.34 years, SD 14.48, two-sample *t* test of Δ≠0: *p* < 0.001). The mean length of follow-up was 6.84 years (SD 2.2).

**Table 1:** Baseline characteristics of the study population by biological aging status

Mean (SD) or n (%)	All (*n* = 2222)	Fast Aging (*n* = 1021)	Slow Aging (*n* = 1201)	*P-*value
Age, years	60.62 (14.01)	61.09 (13.82)	60.23 (14.17)	0.147
PhenoAge, years	59.90 (15.72)	66.43 (14.57)	54.34 (14.48)	1.90 × 10^−78^
Sex				1.31 × 10^−7^
Female	1522 (68.5)	636 (62.3)	886 (73.8)	
Male	693 (31.2)	380 (37.2)	313 (26.1)	
Race				0.008
white participants	1839 (82.8)	838 (82.1)	1001 (83.3)	
black participants	134 (6.0)	78 (7.6)	56 (4.7)	
Other categories combined	249 (11.2)	105 (10.3)	144 (12.0)	
BMI, kg/m^2^	29.84 (6.98)	31.76 (7.48)	28.21 (6.07)	1.00 × 10^−32^
Education				0.187
College or above	2084 (93.8)	958 (93.8)	1126 (93.8)	
12 years or GED	119 (5.4)	50 (4.9)	69 (5.7)	
Income				2.08 × 10^−5^
High	1015 (45.7)	428 (41.9)	587 (48.9)	
Medium	669 (30.1)	303 (29.7)	366 (30.5)	
Low	395 (17.8)	224 (21.9)	171 (14.2)	
Missing	143 (6.4)	66 (6.5)	77 (6.4)	
Employment				0.011
Employed	1260 (56.7)	564 (55.2)	696 (58.0)	
Retired	732 (32.9)	345 (33.8)	387 (32.2)	
Unemployed	159 (7.2)	88 (8.6)	71 (5.9)	
Other employment status	71 (3.2)	24 (2.4)	47 (3.9)	
Smoking				0.048
Yes	788 (35.5)	394 (38.6)	394 (32.8)	
No	1434 (64.5)	627 (61.4)	807 (67.2)	
Current alcohol consumption				0.36
Yes	2168 (97.6)	1000 (97.9)	1168 (97.3)	
No	54 (2.4)	21 (2.1)	33 (2.7)	
Comorbidities				
Cancer	207 (9.3)	92 (9.0)	115 (9.6)	0.702
CVD	247 (11.1)	107 (10.5)	140 (11.7)	0.417
Type 2 diabetes	212 (9.5)	97 (9.5)	115 (9.5)	1.000
Daily steps	7715.83 (3429.04)	7215.22 (3374.21)	8141.42 (3419.17)	1.79 × 10^−10^
Sleep duration, hours	6.39 (1.67)	6.32 (1.67)	6.46 (1.66)	0.048
No recent healthcare contact	181 (8.1)	84 (8.2)	97 (8.1)	0.959
Limited healthcare access	518 (23.3)	232 (22.7)	286 (23.8)	0.578
Logistic barrier	429 (19.3)	202 (19.8)	227 (18.9)	0.637
Finance barrier	312 (14.0)	147 (14.4)	165 (13.7)	0.701
Length of follow-up, years	6.84 (2.2)	6.77 (2.23)	6.90 (2.15)	0.165
Charlson Comorbidity Index				4.17 × 10^−12^
0	841 (37.8)	315 (30.9)	526 (43.8)	
1	463 (20.8)	212 (20.8)	251 (20.9)	
2	343 (15.4)	163 (16.0)	180 (15.0)	
≥3	575 (25.9)	331 (32.4)	244 (20.3)	
Medication use				
Antihypertensive	824 (37.1)	464 (45.4)	360 (30.0)	7.44 × 10^-14^
Statin	530 (23.9)	303 (29.7)	227 (18.9)	3.87 × 10^-9^
Antidiabetic	251 (11.3)	189 (18.5)	62 (5.2)	7.62 × 10^-23^
CNS-active	289 (13.0)	171 (16.7)	118 (9.8)	1.83 × 10^−6^

Values are mean (SD) for continuous variables and n (%) for categorical variables. *P*-values were calculated using two-sided two-sample t-tests for continuous variables and two-sided chi-square tests for categorical variables, as appropriate. Sex category counts do not sum to the total N because sex categories other than male or female were suppressed according to the AoURP Data and Statistics Dissemination Policy.

Compared to slow agers, the fast agers had a greater proportion of males (37.2% vs. 26.1%, *p* < 0.001) and a higher mean body mass index (BMI: 31.76 kg/m² vs. 28.21 kg/m²; *p* < 0.001). Socioeconomic indicators showed consistent differences as low income was more prevalent among fast agers (21.9% vs. 14.2%; *p* < 0.001), as was unemployment (8.6% vs. 5.9%; *p* = 0.011). Fast agers also had a higher prevalence of lifetime smoking (38.6% vs. 32.8%; *p* = 0.048). In line with behavioral differences, fast agers showed shorter average sleep duration (6.32 hours vs. 6.46 hours; *p* = 0.048) and lower wearable-derived daily step counts (7215 steps vs. 8141 steps; *p* < 0.001). Education, current alcohol consumption, and comorbidities of cancer, cardiovascular disease (CVD), and diabetes were comparable between groups. However, comorbidity burden, as measured by the Charlson Comorbidity Index (CCI), differed significantly between groups (*p* < 0.001). Individuals with no comorbidity (CCI = 0) were more prevalent among slow agers (43.8% vs. 30.9%), whereas a high comorbidity burden (CCI ≥ 3) was more common among fast agers (32.4% vs. 20.3%), indicating that differences were primarily driven by the extremes of comorbidity burden. Similarly, medication use differed significantly by biological aging status (all *p* < 0.001). Fast agers had a higher prevalence of antihypertensive (45.4% vs. 30.0%), statin (29.7% vs. 18.9%), antidiabetic (18.5% vs. 5.2%), and CNS-active medication use (16.7% vs. 9.8%) compared to slow agers. These patterns suggest a higher treatment burden and greater cardiometabolic and neurocognitive morbidity among individuals with accelerated biological aging.

PhenoAge was strongly correlated with chronological age (Pearson correlation coefficient, *r* = 0.87, *p* < 0.001) in the full sample, with consistent associations observed in males (*r* = 0.85, *p* < 0.001) and females (*r* = 0.88, *p* < 0.001) (Supplementary Fig. 1). The distribution of biological age acceleration (ΔAge) approximated normality and was centered near zero. Sex-specific distributions of ΔAge were comparable in terms of central tendency and dispersion.

Baseline rest-activity profiles by biological aging status (Supplementary Fig. 2) further highlighted rhythmicity distinctions. Across age strata, fast agers exhibited consistently lower peak activity levels and attenuated daytime intensity compared to slow agers. They also showed higher nocturnal activity between 00:00-06:00, whereas slow agers maintained a more consolidated rest period, consistent with a more robust rest-activity rhythm.

### Age-related trends in circadian rest-activity rhythms (CRAR) and functional components

**Fig. 1:**
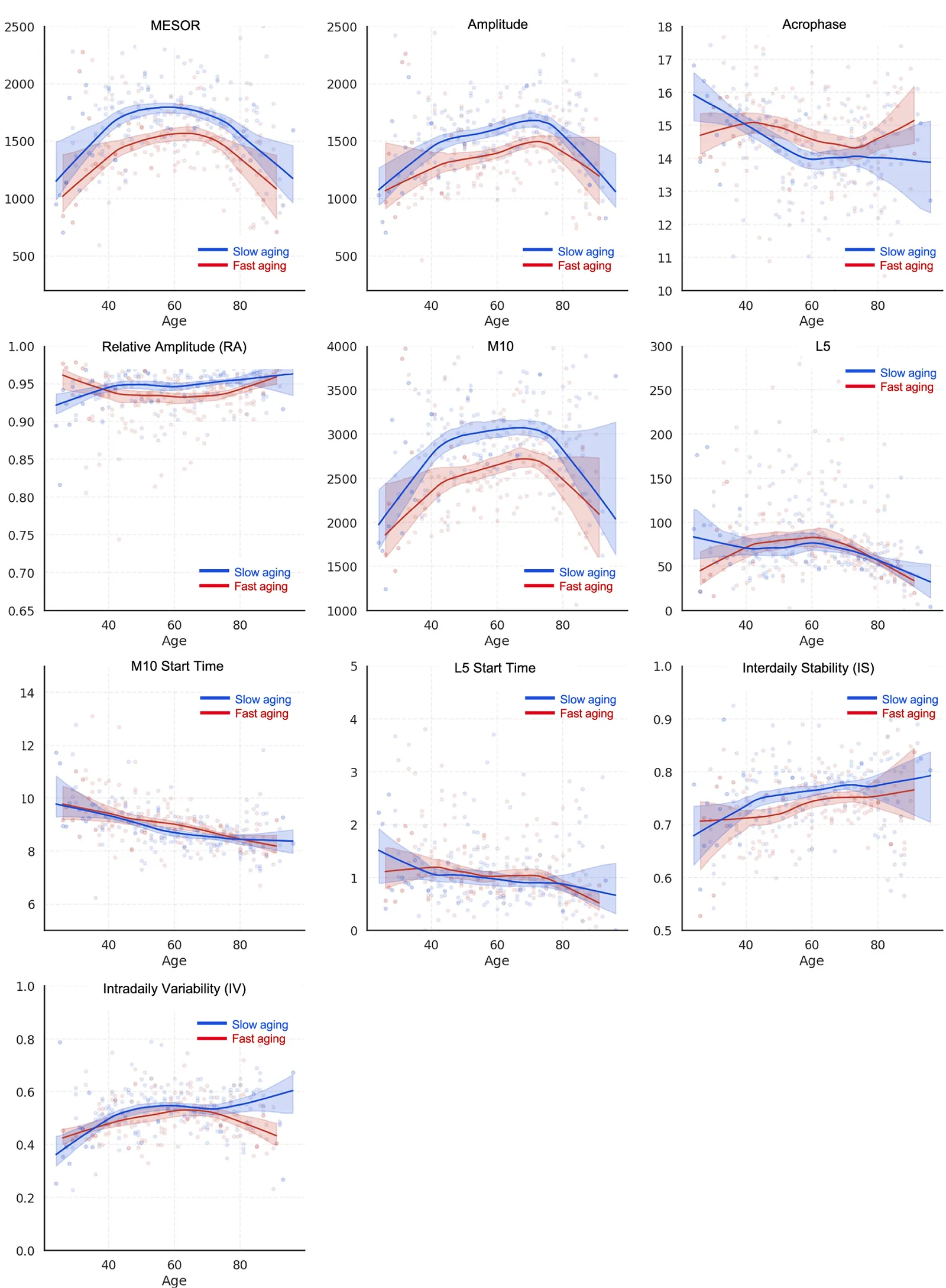
Age-associated trends in circadian rest-activity rhythm (CRAR) metrics by biological age acceleration. Points are color-coded by biological aging status at baseline (blue = slow aging, red = fast aging). Lines represent locally weighted scatterplot smoothing (LOESS) estimates of the smoothed mean trajectory for each aging group. Shaded bands indicate 95% confidence intervals around the LOESS smoothed mean trajectory derived from 1000 bootstrap resamples. Source data are provided as a Source Data file.

Figure 1 illustrates age-related trends in Circadian Rest-Activity Rhythm (CRAR) metrics. Across age ranges, slow agers steadily exhibited higher MESOR (Midline Estimating Statistic Of Rhythm, a rhythm-adjusted mean level of activity across the 24 h cycle estimated from the cosinor model), cosinor amplitude, RA (relative amplitude), M10 (mean activity during the 10 most active hours), and IS (interdaily stability; day-to-day rhythm regularity) compared with fast agers. Differences in acrophase emerged from midlife ( ~ 40 years) onward, with fast agers showing delayed acrophase. Sex-stratified analyses confirmed the robustness of these trends (Supplementary Fig. 3). In both sexes, fast aging was linked to lower MESOR, amplitude, M10, and IS, but the magnitude of the difference varied. In females, differences remained stable with age, whereas in males they attenuated after 60. For L5 (mean activity during the least active hours), differences were more pronounced in males.

**Fig. 2:**
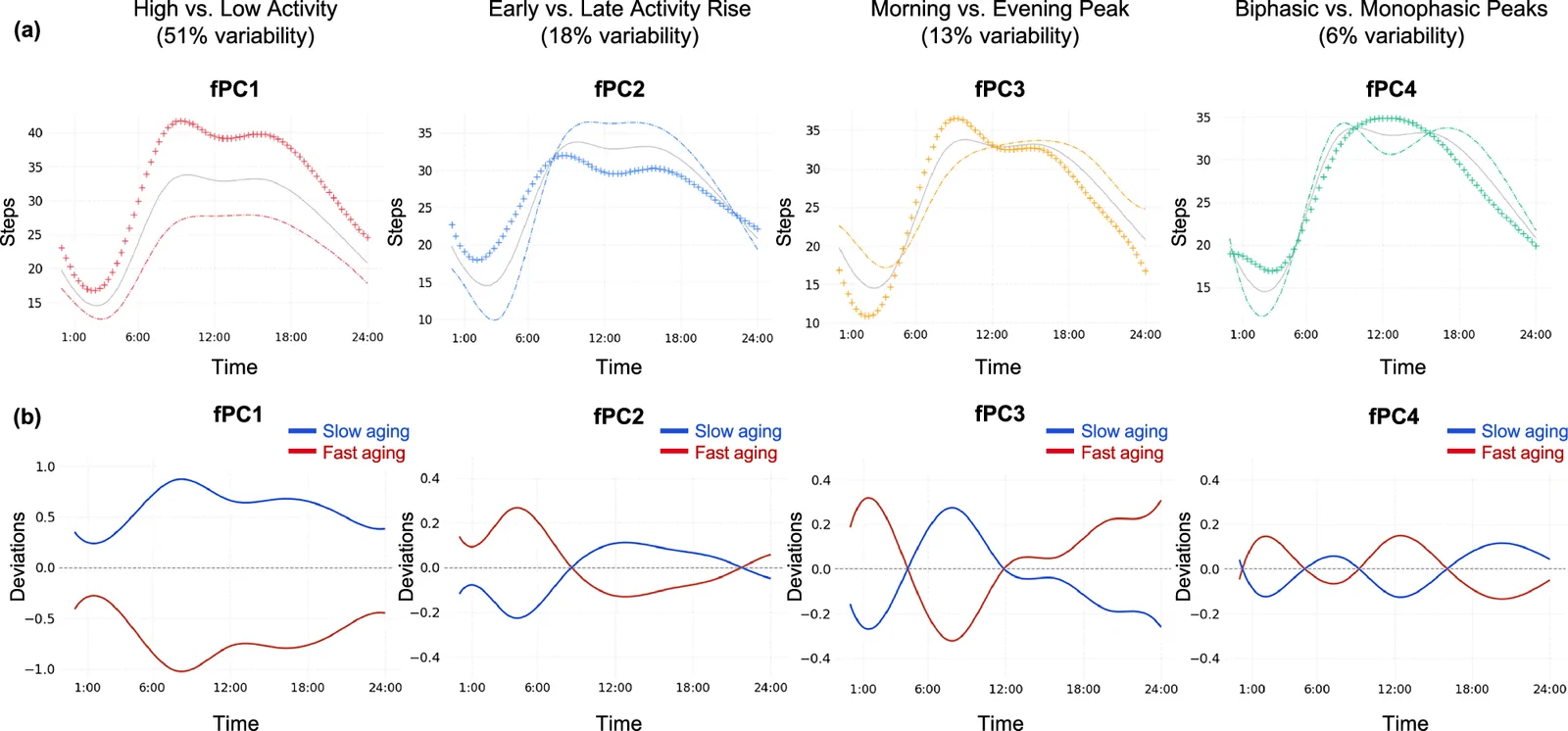
Functional principal components (fPCs) of 24-hour activity profiles and their association with biological age acceleration. **a** The mean 24-hour activity profile (gray) and variation along each functional principal component are shown. Colored curves represent positive (++++) and negative (----) deviations from the mean trajectory (i.e., mean ± scaled eigenfunctions), illustrating the dominant temporal patterns captured by each fPC. **b** Line plots depict group-level deviations from the population mean, computed as the mean fPC score multiplied by the corresponding eigenfunction for each biological aging group. These curves represent deviations from the mean activity profile and illustrate how circadian rest-activity patterns differ across fast aging vs. slow aging groups. fPC scores were standardized (mean = 0, variance = 1), and eigenfunctions were scaled to the range [− 1, 1] for visualization. Source data are provided as a Source Data file.

Functional principal component analysis (fPCA) was performed on the 24-hour smartwatch-based step-count trajectories to identify dominant modes of variation in daily activity rhythms (Fig. 2). The first four fPCs accounted for 88% of total variance. fPC1 (accounting for 51% of the variance) quantified overall daytime activity amplitude, with higher scores indicating greater activity levels. fPC2 (18% of the variance) represented the timing of activity onset, where lower scores reflected delayed transitions from rest to activity. fPC3 (13% of the variance) described the timing of peak activity, differentiating morning- from evening-dominant patterns. fPC4 (6% of the variance) distinguished biphasic from monophasic activity structures.

Group-level comparisons revealed distinct rhythm signatures of fast aging. Fast agers exhibited lower fPC1 scores reflecting reduced daytime activity. fPC2 trajectories indicated delayed activity rise with blunted midday recovery. fPC3 revealed late-evening activity peaks, which suggests rest-activity timing disruption or shifted behavioral rhythms. fPC4 indicated biphasic activity with early-morning and late-day peaks. Correlation analyses (Supplementary Fig. 4) confirmed fPC1 aligned with MESOR, amplitude, RA, and M10, underscoring its representation of rhythm intensity. fPC2 and fPC3 correlated with timing metrics such as acrophase, M10 start time, and L5 start time.

To further evaluate whether the fPCs correspond to sleep-related behavioral patterns, we examined their associations with independently derived Fitbit sleep metrics at the sequence level (Supplementary Fig. 5), focusing on components reflecting timing and fragmentation. fPC3 showed consistent associations with sleep timing measures, including sleep onset (*r* = 0.38) and sleep end time (*r* = − 0.19), indicating a coordinated shift in the sleep-wake schedule. These findings align with the activity-based timing associations observed for fPC3, supporting its interpretation as a circadian phase-related component. In contrast, fPC4 showed weak associations across sleep metrics, with the largest correlation observed for nap frequency (*r* = 0.08), and no dominant relationship with sleep timing, duration, or efficiency, suggesting that fPC4 may capture distributed irregularity in daily activity organization rather than a specific sleep construct.

Sex-specific fPC profiles sharpened these patterns (Supplementary Fig. 6). Females showed more pronounced timing-related shifts (fPC2, fPC3), while males displayed distinct fPC4 separation, with fast-aging males exhibiting bimodal rhythms and increased nocturnal activity. Together, CRAR metrics and fPCs delineate a fast-aging phenotype characterized by reduced amplitude, delayed activity onset/peak, and rhythm fragmentation, with sex-specific nuances.

### Associations between functional and CRAR features and accelerated biological aging at baseline and follow-up

**Fig. 3:**
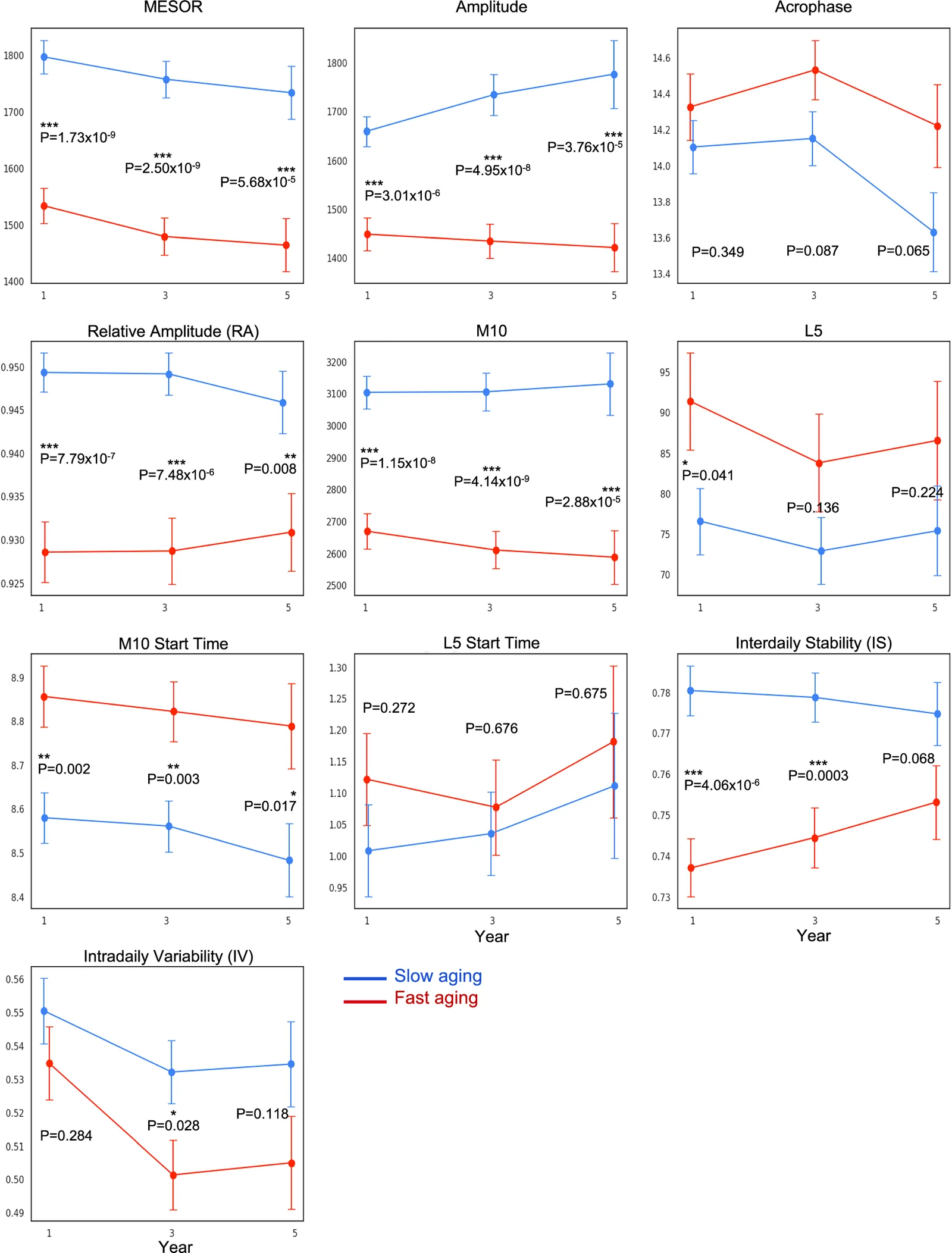
Trajectories of CRAR metrics over 5 years stratified by biological age acceleration. Fast and slow aging groups included participant-year observations from participants who were classified in the corresponding biological aging category at two or more time points across Years 1−5. Data are presented as mean values ± standard error of the mean (SEM). The unit of analysis was the participant-year observation with concurrent wearable-derived features and biological aging measures. Sample sizes were Year 1: fast aging *n* = 552 and slow aging *n* = 726; Year 3: fast aging* n* = 546 and slow aging* n* = 722; Year 5: fast aging *n* = 287 and slow aging *n *= 382. *P*- values are from two-sided between-group* t* tests at each time point. Asterisks denote *P < 0.05, **P < 0.01, ***P < 0.001. Source data are provided as a Source Data file.

To evaluate temporal patterns in CRAR features in relation to biological aging, we first characterized descriptive trajectories of CRAR features across years 1, 3, and 5 (Fig. 3). Overall, fast agers exhibited lower rhythm intensity (MESOR, amplitude, RA, M10), reduced regularity (IS), and delayed timing (M10 start time) compared to slow agers. These differences were generally consistent across time points, although their magnitude varied.

Building on these descriptive patterns, we next assessed the associations of rhythmicity with accelerated biological aging at baseline and 5-year follow-up (Table 2). Across both time points, higher rhythm intensity (fPC1, MESOR, amplitude, RA, M10) and greater rhythm regularity (IS) were associated with lower odds of accelerated aging, whereas delayed timing metrics (acrophase, M10 start time, L5 start time) were associated with higher odds. For example, each quartile increase in fPC1 corresponded to 19.0% lower odds of accelerated aging at baseline (OR = 0.810; 95% CI: 0.745-0.878) and a 19.4% reduction at follow-up (OR = 0.806; 95% CI: 0.697-0.933). Comparable associations in the protective direction were observed for MESOR (baseline OR = 0.778; follow-up OR = 0.787), amplitude (baseline OR = 0.781; follow-up OR = 0.831), RA (baseline OR = 0.829; follow-up OR = 0.889), and M10 (baseline OR = 0.762; follow-up OR = 0.779).

**Table 2:** Associations between functional and CRAR metrics and accelerated biological aging at baseline and follow-up

Features	Baseline OR (95% CI)	P value	Follow-up OR (95% CI)	*P*- value
fPC1	**0.810 (0.745, 0.878)**	**4.94 × 10**^**−7**^	**0.806 (0.697, 0.933)**	**0.004**
fPC2	1.044 (0.965, 1.130)	0.287	0.974 (0.853, 1.169)	0.699
fPC3	0.956 (0.884, 1.035)	0.265	0.909 (0.799, 1.036)	0.152
fPC4	1.003 (0.927, 1.086)	0.934	0.978 (0.857, 1.108)	0.694
MESOR	**0.778 (0.717, 0.845)**	**2.09 × 10**^**−9**^	**0.787 (0.680, 0.910)**	**0.001**
Amplitude	**0.781 (0.718, 0.847)**	**4.51 × 10**^**−9**^	**0.831 (0.721, 0.958)**	**0.011**
Acrophase	**1.109 (1.024, 1.200)**	**0.011**	1.014 (0.887, 1.161)	0.835
Relative amplitude (RA)	**0.829 (0.764, 0.899)**	**6.26 × 10**^**−6**^	0.889 (0.776, 1.019)	0.093
M10	**0.762 (0.703, 0.829)**	**1.03 × 10**^**−10**^	**0.779 (0.674, 0.901)**	**0.001**
L5	1.047 (0.966, 1.134)	0.266	1.018 (0.891, 1.163)	0.795
M10 start time	1.066 (0.983, 1.155)	0.124	**1.149 (1.001, 1.318)**	**0.048**
L5 start time	**1.106 (1.021, 1.198)**	**0.014**	1.122 (0.982, 1.280)	0.09
Interdaily stability (IS)	**0.889 (0.820, 0.964)**	**0.004**	0.879 (0.768, 1.006)	0.061
Intradaily variability (IV)	0.938 (0.866, 1.016)	0.116	0.910 (0.795, 1.042)	0.172

Odds ratios (ORs) and 95% confidence intervals (CIs) were estimated using multivariable logistic regression models. *P*-values are from two-sided Wald tests for the regression coefficients. Models were adjusted for age, sex, race, education, income, employment, lifetime smoking, current alcohol consumption, and comorbidities (cancer, cardiovascular disease, type 2 diabetes). Significant associations (*P* < 0.05) are shown in bold.

Higher rhythm regularity (IS) was associated with reduced odds of accelerated aging at baseline (OR = 0.889; 95% CI: 0.820-0.964), with a similar but non-significant association at follow-up (OR = 0.879; 95% CI: 0.768−1.006).

In contrast, delayed acrophase (peak activity timing) and late L5 start time (the least active 5-hour onset timing) increased baseline odds by 10.9% (OR = 1.109; 95% CI: 1.024-1.200) and 10.6% (OR = 1.106; 95% CI: 1.021-1.198), respectively, and delayed M10 start time (the most active 10 h onset timing) raised follow-up odds by 14.9% (OR = 1.149; 95% CI: 1.001-1.318).

**Fig. 4:**
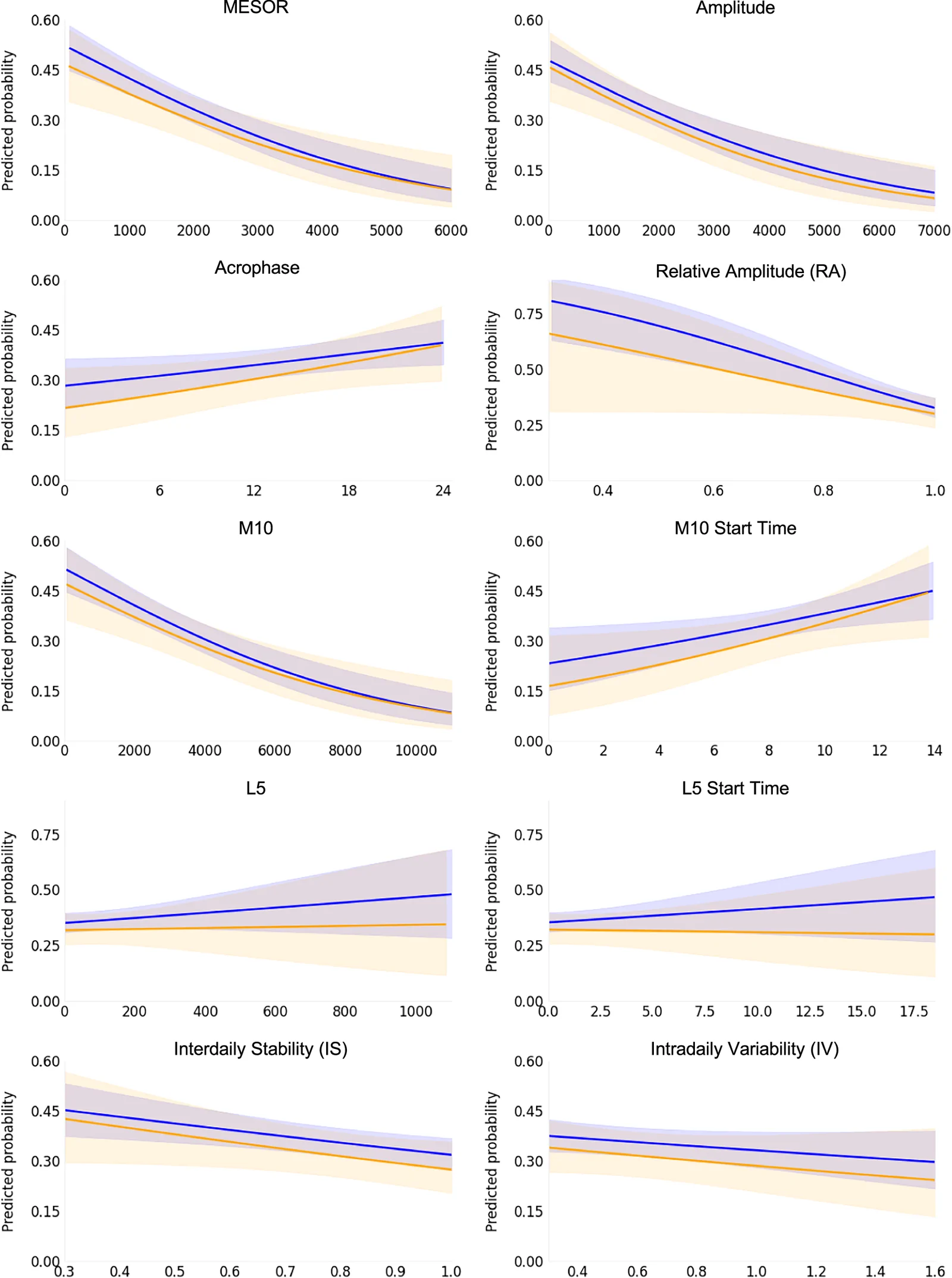
Predicted probabilities of accelerated biological aging across CRAR metrics. Each panel displays the model-estimated predicted probability of fast aging across the observed range of a given CRAR metric. Blue and orange lines correspond to baseline and follow-up, respectively. Shaded bands indicate 95% confidence intervals around the model-estimated predicted probabilities. Predicted probabilities were derived from multivariable logistic regression models, holding covariates at their mean (continuous variables) or mode (categorical variables) values. All models are adjusted for age, sex, race, education, income, employment, lifetime smoking, current alcohol consumption, and comorbidities (cancer, cardiovascular disease, type 2 diabetes). Source data are provided as a Source Data file.

For each CRAR feature, we further estimated model-based predicted probabilities of accelerated aging at baseline and follow-up across the observed range of the feature to facilitate interpretation of results (Fig. 4). Sex-stratified analyses (Supplementary Table 1) showed overall consistency, though timing effects were more pronounced in females, while rhythm intensity remained protective across both sexes. While several associations were statistically significant in sex-stratified analyses, not all corresponding interaction terms reached statistical significance (Supplementary Table 3), indicating that between-sex differences in effect size were modest for some features.

### Longitudinal associations of functional and CRAR metrics with biological aging

To move beyond cross-sectional contrasts, we evaluated whether changes in rhythm were associated with changes in biological aging over time (Table 3). Generalized linear mixed-effects models demonstrated robust longitudinal associations, accounting for within-subject repeated measurements. Higher activity intensity and rhythm robustness were associated with reduced odds of accelerated aging across the study period. A one-quartile increase in fPC1 corresponded to a 36.9% reduction in the odds (OR = 0.631; 95% CI: 0.526-0.757; *p* < 0.001). Similarly, increases in MESOR, amplitude, RA, and M10 were linked to 42.4% (OR = 0.576; 95% CI: 0.475-0.699), 40.2% (OR = 0.598, 95% CI: 0.494-0.723), 26.5% (OR = 0.735, 95% CI: 0.610-0.884), and 46.3% (OR = 0.537, 95% CI: 0.442-0.652) reductions, respectively (all *p* ≤ 0.001). Increased rhythm regularity was associated with a non-significant 9% reduced odds (OR = 0.910, 95% CI: 0.756-1.095).

**Table 3:** Longitudinal associations of functional and CRAR metrics with biological aging over time

Features	OR (95% CI)	*P*-value
fPC1	**0.631 (0.526, 0.757)**	**7.13 × 10**^**−7**^
fPC2	0.983 (0.825, 1.172)	0.851
fPC3	0.955 (0.801, 1.140)	0.611
fPC4	1.022 (0.857, 1.219)	0.807
MESOR	**0.576 (0.475, 0.699)**	**2.18 × 10**^**−8**^
Amplitude	**0.598 (0.494, 0.723)**	**1.21 × 10**^**−7**^
Acrophase	**1.221 (1.015, 1.468)**	**0.034**
Relative amplitude (RA)	**0.735 (0.610, 0.884)**	**0.001**
M10	**0.537 (0.442, 0.652)**	**3.62 × 10**^**−10**^
L5	1.070 (0.974, 1.175)	0.157
M10 start time	1.060 (0.878, 1.279)	0.545
L5 start time	1.082 (0.899, 1.302)	0.405
Interdaily stability (IS)	0.910 (0.756, 1.095)	0.319
Intradaily variability (IV)	0.872 (0.723, 1.053)	0.155

Odds ratios (ORs) and 95% confidence intervals (CIs) were estimated using generalized linear mixed-effects regression models with participant-level random intercepts. *P*-values are from two-sided Wald tests of the regression coefficients. Models were adjusted for age, sex, race, education, income, employment, lifetime smoking, current alcohol consumption, and comorbidities (cancer, cardiovascular disease, type 2 diabetes). Significant associations (*P* < 0.05) are shown in bold.

In contrast, delayed acrophase was associated with increased odds by 22.1% (OR = 1.221; 95% CI: 1.015-1.468; *p* = 0.034), while later M10 and L5 start timings showed modest, non-significant increases by 6.0% (OR = 1.060; 95% CI: 0.878-1.279) and 8.2% (OR = 1.082; 95% CI: 0.899-1.302), respectively.

The attenuation of statistical significance for certain timing and regularity metrics in longitudinal models, despite consistent cross-sectional associations, highlights the distinction between between-individual and within-individual effects. For some features, limited intra-individual variability over time may reduce the ability to detect longitudinal associations, even when they exhibit strong population-level differences.

**Fig. 5:**
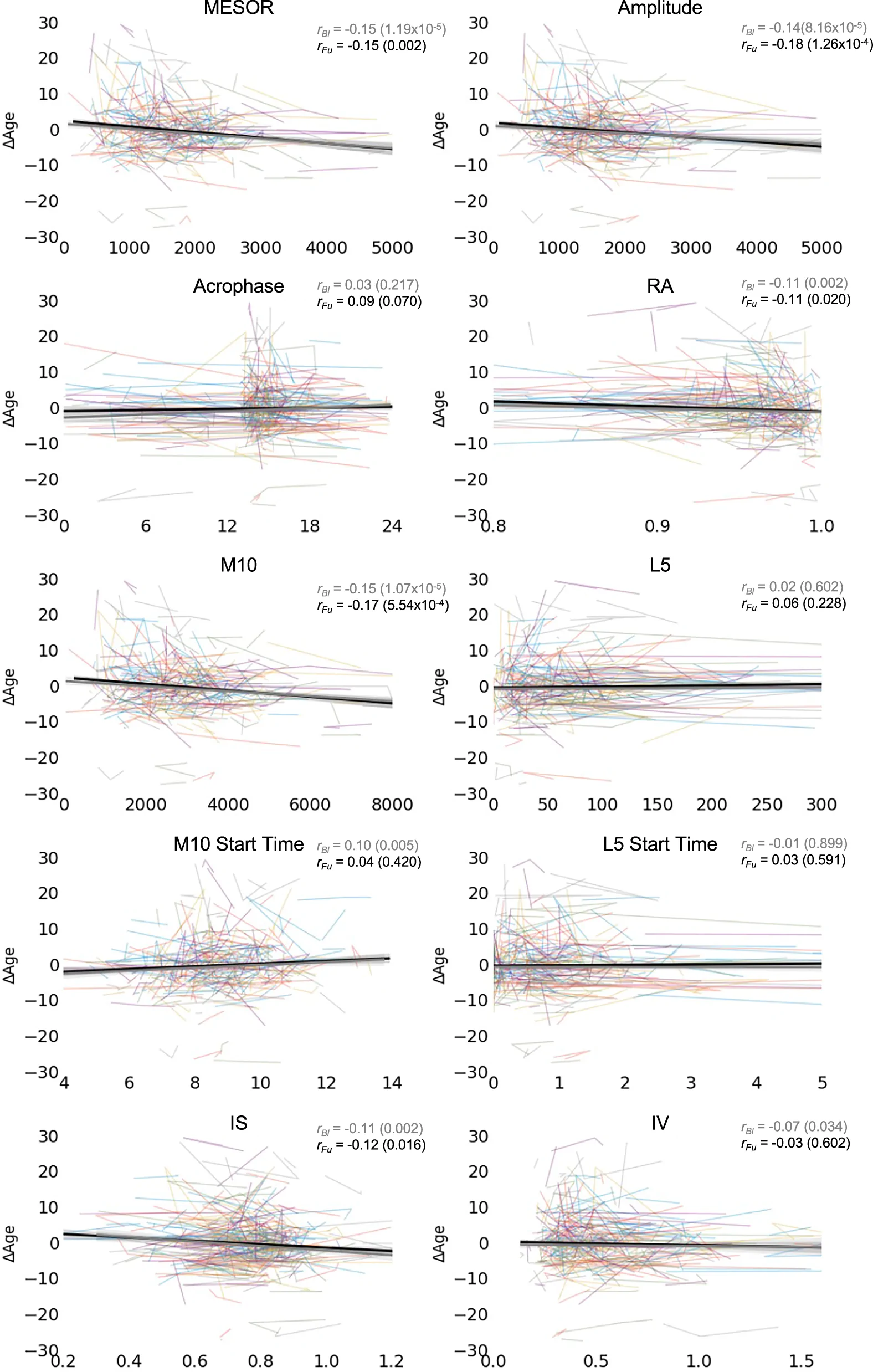
Within-individual changes in CRAR metrics versus change in biological aging. Each panel shows the association between one wearable-derived feature and within-person change in biological age acceleration (ΔAge, years). The bold lines represent population-level linear regression fits, and shaded bands indicate 95% confidence intervals around the fitted regression lines. Colored thin lines represent individual-level regression slopes estimated for a random sample of 100 participants with at least two observations. Pearson correlation coefficients at baseline (r_Bl_) and follow-up (r_Fu_) are shown within each panel, with *P*-values in parentheses. *P*-values are from two-sided Pearson correlation tests. Source data are provided as a Source Data file.

Within-person analyses further supported these findings (Fig. 5). Increases in intensity metrics (MESOR, amplitude, RA, M10) were associated with negative ΔAge slopes, consistent with slower biological aging, whereas delays in acrophase and M10 start time produced positive ΔAge slopes, indicating accelerated aging trajectories. Dispersion of slopes was narrower for intensity metrics, suggesting more uniform associations in the protective direction across individuals, while timing effects were more heterogeneous. To further isolate within-person associations, we conducted additional change–change (Δ–Δ) regression analyses (Supplementary Table 4). Consistent with the mixed-effects models, increases in activity intensity metrics, including MESOR, amplitude, and M10, were significantly associated with decreases in ΔAge (all *p* < 0.05), indicating that improvements in activity levels are linked to slower biological aging within individuals. In addition, greater interdaily stability (IS) was associated with lower ΔAge, while later nocturnal timing, reflected by L5 start time, was associated with higher ΔAge.

Sex-stratified longitudinal analyses (Supplementary Table 2) confirmed these trends and highlighted sex-specific nuances. Intensity-related lower odds of accelerated aging was evident in both sexes. In males, higher fPC1, MESOR, amplitude, and M10 consistently were associated with lower odds of fast aging by 15-30%, whereas the effect sizes were larger in females, ranging from 22 to 40%. Increased rhythm regularity was associated with lower odds of accelerated aging by 15% in females, while showing no statistically significant effect in males. Similarly, delayed acrophase was associated with higher odds of fast aging by 12% in males and 18% in females, but was statistically significant only in females.

The longitudinal analyses extend the cross-sectional findings by showing that within-individual changes in CRAR are associated with changes in biological aging. Specifically, fPC1 (reflecting overall rhythm intensity and robustness) exhibited evidence of time-dependent variation (OR = 1.218, 95% CI: 1.073-1.382). In contrast, other rhythmicity metrics, including timing and regularity measures, did not show significant time-dependent effects. Consistent with the cross-sectional results, intensity- and robustness-related features emerged as the features most consistently associated with accelerated biological aging, while timing and regularity showed more sex-specific or weaker longitudinal effects.

To assess the robustness of our findings, we performed extensive sensitivity analyses. To address time-varying confounding, including comorbidities, medication use, and acute health events, we conducted inverse probability of treatment weighting (IPTW) analyses (Supplementary Table 5). The IPTW-adjusted estimates were largely consistent with the primary analyses, with several associations strengthened or reaching statistical significance. Higher CRAR intensity metrics remained associated with reduced odds of accelerated aging, whereas delayed timing metrics were associated with increased odds, indicating that the observed associations were stable after accounting for time-varying confounding.

Our findings were also robust to the handling of missing biomarker data. Using multivariate imputation by chained equations (MICE), the resulting PhenoAge estimates were strongly correlated with those obtained using mean imputation across the overall sample and by sex (*r* = 0.916-0.943). Longitudinal associations between functional and CRAR features and biological aging also remained consistent in both magnitude and direction across imputation approaches (Supplementary Fig. 8 and Supplementary Table 6).

Additional sensitivity analyses yielded consistent results across multiple model specifications. Associations remained stable when modeling ΔAge as a continuous outcome and across quartiles of CRAR features, demonstrating graded dose-response relationships (Supplementary Tables 7–9). Key CRAR metrics, including intensity, regularity, and timing, remained significantly associated with accelerated aging and PhenoAge at baseline and during follow-up (Supplementary Table 10). Interaction analyses showed limited evidence of effect modification across CRAR domains or by chronological age, with most interaction terms close to the null (Supplementary Tables 11, 12).

Adjustment for physical activity such as moderate-to-vigorous activity (MVPA) minutes and sleep-related metrics resulted in only partial attenuation of effect estimates, with substantial associations remaining for CRAR measures (Supplementary Tables 13, 14).

We additionally assessed potential selection bias related to healthcare access and completeness of laboratory-based outcome data. Excluded individuals were younger, had lower income, fewer comorbidities, and greater barriers to healthcare across logistical, financial, and access domains (Supplementary Table 15). However, further adjustment for healthcare utilization, access to care, and length of follow-up did not materially alter the observed associations (Supplementary Table 16). These findings indicate that the observed associations were robust to potential selection and observation biases.

Several CRAR metrics also showed longitudinal stability between baseline and follow-up, supporting the reproducibility of wearable-derived rhythm measures over time (Supplementary Fig. 10).

Lastly, supervised approaches based on partial least squares (PLS) components were closely aligned with fPCs, consistent with both methods capturing overlapping aspects of the same underlying functional variation under different optimization criteria (Supplementary Fig. 9 and Supplementary Table 17). PLS1 was strongly correlated with fPC1 (*r* = 0.96), and PLS2 was strongly correlated with fPC3 (*r* = − 0.84), indicating overlap with activity-intensity and timing-related variation. PLS3 was correlated with both fPC2 (*r* = 0.53) and fPC4 (*r* = − 0.65), while PLS4 showed weaker and more distributed correlations across fPCs. These results support the use of PLS as a complementary sensitivity analysis and fPCA as an outcome-agnostic representation of the major modes of rest-activity variation.

## Discussion

In this large-scale, prospective cohort with multi-year wearable monitoring in free-living settings, we demonstrated that circadian rest-activity rhythms were robustly associated with biological aging trajectories, assessed using clinical biomarker-based PhenoAge. Rhythm intensity, regularity, and timing emerged as important features associated with accelerated aging and PhenoAge at baseline and during follow-up. Longitudinal analyses indicated that changes in rest-activity rhythms were associated with biological aging trajectories across five years. Specifically, higher rhythm intensity was associated with lower odds of accelerated aging by 26–46%, greater regularity by 9–13%, whereas delayed timing was associated with higher odds by 22%. Rhythm intensity was consistently associated with lower odds across both sexes, while reduced regularity and delayed timing were specific features associated with higher odds in females. Males exhibited a distinctive biphasic instability phenotype linked to accelerated aging. By leveraging repeated longitudinal assessments, these findings move beyond single-time-point associations and support the potential utility of consumer wearable-derived rest-activity rhythms as digital biomarkers of biological aging. Given the increasing scalability and ubiquity of consumer wearables, this work shows the potential and may inform future studies of continuous monitoring and interventions to support digital precision medicine and promote healthy aging.

Among the CRAR domains, timing- and regularity-related features (e.g., acrophase, IS) exhibited greater variability across models compared to intensity-related metrics (e.g., amplitude, RA). This likely reflects their smaller effect sizes and greater sensitivity to model parameterization and behavioral context. In addition, these features tend to show non-linear and heterogeneous relationships across their distributions, with effects more pronounced at the extremes. Such variability is further expected in real-world, free-living data, where timing and regularity are likely to be influenced by external constraints (e.g., work schedules, social timing, environmental factors). Despite this variability, effect directions remained consistent across analyses, supporting the robustness of the overall findings. This variability also enhances the external validity and generalizability of the results by reflecting behavior under real-world conditions. Consistent with this variability, differences between modeling approaches provide additional context for interpreting these features. Activity intensity metrics (MESOR, amplitude, and M10) showed consistent associations with biological aging across both linear mixed-effects models and change–change (Δ–Δ) analyses. In contrast, timing and stability features exhibited model-specific patterns, with relative amplitude (RA) and daytime timing (M10 start time) identified in mixed-effects models, and rhythm stability (IS) and nocturnal timing (L5 start time) emerging in Δ–Δ analyses. This divergence is consistent with their greater sensitivity to model specification and suggests that these features may reflect more context-dependent and within-person processes.

Our results extend prior evidence linking CRAR disruption to health outcomes. Reduced amplitude has been consistently identified as a strong predictor of mortality, cardiovascular disease, cancer, and neurodegenerative disorders^15,37–40^, with more recent associations to preclinical conditions such as frailty^41^ and cognitive decline^42^. Decrease in amplitude is also further tied to systemic inflammation (“inflammaging”), a hallmark of aging and a common pathway for multiple chronic diseases^3,43^, suggesting that weakening rest-activity rhythm may be associated with accelerated aging even at younger ages^44^. Our findings extend this by showing that, in longitudinal analyses, amplitude and rhythm intensity remain the features most consistently associated with accelerated aging.

Timing-related disruption provides another pathway of vulnerability. Delayed acrophase or later activity onset has been associated with elevated risk of cardiovascular disease, obesity, cancer, and dementia^45–47^, though effect sizes and significance vary across studies. Chronic night shift work, an exemplar of sustained circadian misalignment, has been linked to higher brain age indices, greater prevalence of metabolic syndrome, and increased risk of atrial fibrillation and coronary heart disease^48–50^. These cumulative, persistent effects are often underestimated or missed in cross-sectional studies. Consistent with this, our findings demonstrated that delayed acrophase was prospectively associated with accelerated aging, suggesting a potential link between rest-activity timing disruption and long-term aging trajectories.

Furthermore, rhythm regularity adds complementary insights. While day-to-day regularity often increases with aging^51^, reduced stability and greater fragmentation have been associated with a higher risk of neurodegenerative disease^52^. In our cohort, greater regularity was associated with lower odds of accelerated aging in females, whereas in males, we observed a biphasic instability phenotype, characterized by an early-morning surge and late-evening rebound, uniquely linked to accelerated aging.

Together, these results emphasize the importance of assessing rhythm amplitude, regularity, and timing as an integrated multidimensional framework rather than as isolated parameters. In this line of work, prior studies have shown that wearable-based CRAR-based age outperforms physical activity-based age in predicting morbidity and mortality across cardiovascular, metabolic, and neurodegenerative diseases, reinforcing the value of comprehensive rhythm profiling^18^. Evidence from a cohort of 191 older adults further showed that rhythm intensity, regularity, and timing jointly associated with epigenetic age acceleration, showing their interdependent effects^53^. By combining repeated measures of biological age acceleration and high-dimensional functional analyses, our study advances prior cross-sectional evidence and supports a longitudinal association of rest-activity rhythm patterns and aging trajectories.

A distinctive contribution of this work lies in the integration of data-driven functional principal components analysis (fPCA) with conventional scalar CRAR metrics, enabling high-dimensional digital phenotyping of rhythm intensity, regularity, and timing. fPCA revealed additional latent signatures characterizing accelerated aging, including decreased overall robustness (fPC1), delayed initiation (fPC2), peak misalignment (fPC3), and biphasic instability (fPC4). These functional dimensions demonstrated strong concordance with scalar measures while providing enhanced biological granularity. Notably, fPC1, reflecting rhythm robustness, was one of the most sensitive predictors of accelerated aging, underscoring the primacy of rhythm intensity as a feature associated with lower odds. This demonstrates the value of multidimensional digital phenotyping for uncovering hidden vulnerabilities in circadian-aging biology.

Importantly, the interpretation of these fPCs is further informed by their alignment with independently derived sleep measures. While fPC1 and fPC2 are well anchored to CRAR metrics, fPC3 and fPC4 require additional context. In our additional analyses leveraging Fitbit-derived sleep data, fPC3, which reflects variation in activity timing, was associated with sleep onset and wake timing, indicating coordinated shifts in the sleep–wake schedule across domains. By contrast, fPC4 showed only weak and non-specific associations with sleep metrics, including a modest relationship with nap frequency, without a consistent pattern across timing, duration, or efficiency. This distinction suggests that fPC3 captures a coherent timing-related dimension observable across behavioral modalities and is consistent with a timing-related construct, providing support for its biological plausibility. In contrast, fPC4 reflects more diffuse irregularity in daily activity organization that is not readily captured by conventional sleep measures.

Sex-stratified analyses further characterized these insights by revealing differential patterns. Rhythm intensity was broadly associated with lower odds of accelerated aging in both sexes, whereas timing- and regularity-related associations were stronger in females, where delayed acrophase and later rest onset was associated with higher odds of accelerated aging by 12–18% and greater regularity was related to lower odds by 15%. In males, timing-related effects were weaker, yet fPCA identified the biphasic instability phenotype as a distinctive marker of accelerated aging.

Methodologically, partial least squares (PLS) and functional principal component analysis (fPCA) yielded highly concordant low-dimensional representations of rest-activity trajectories. Both methods form linear combinations of the same underlying functional variation but optimize different criteria: variance explained for fPCA and covariance with the outcome for PLS. We present fPCA as the primary representation in this study because it is outcome-agnostic and provides an orthogonal decomposition of the functional trajectories, with components that are mutually orthogonal in function space (i.e., with zero inner products over time), thereby facilitating interpretation and subsequent analyses.

We did not observe significant differences in the baseline prevalence of major comorbidities, including cancer, cardiovascular disease, and diabetes, between fast and slow agers. However, overall comorbidity burden, as measured by the Charlson Comorbidity Index (CCI), and medication use differed substantially between groups, with fast agers exhibiting higher multimorbidity and greater treatment burden. This may suggest that accelerated biological aging is more strongly reflected in the accumulation and severity of conditions rather than the presence of individual diagnoses at a single time point.

This pattern further supports that biological age measures may capture underlying physiological processes that may precede clinically manifest disease and, therefore, may not be fully reflected in cross-sectional disease prevalence. Prior evidence similarly shows that biological aging metrics are more strongly associated with incident disease and mortality than with prevalent conditions^54^.

In addition, the *All of Us* cohort, particularly the subset with wearable data, represents a relatively healthy population, with a higher proportion of younger, non-Hispanic white female participants. Such characteristics may reduce variability in baseline disease prevalence and attenuate cross-sectional differences between groups. Accordingly, differences in biological aging may not necessarily manifest as differences in diagnosed conditions at baseline, particularly in relatively healthy population-based cohorts.

Several mechanisms may explain these sex-specific associations. First, aging is accompanied by intrinsic weakening of circadian amplitude, reduced output from the suprachiasmatic nucleus (SCN), and impaired peripheral clock coordination. Importantly, the SCN can regulate circadian phase and rhythm in a sex-specific manner through hormonal modulation, behavioral differences in rest-activity and sleep quality, and immunological dimorphisms^55–57^. For example, estrogen enhances pacemaker sensitivity and circadian amplitude, potentially accounting for the stronger phase-related effects observed in females. In addition, females generally exhibit more robust and stable circadian rhythms, whereas males more often display rhythm fragmentation and inconsistency^51^ and lower sleep efficiency^58^. Under real-world conditions, males also tend to have later chronotypes^59^, predisposing them to higher rates of social jetlag^59^ and associated circadian disruption. Collectively, these results suggest that circadian aging trajectories diverge by sex, shaped by an interplay of intrinsic molecular regulation and behavioral-environmental exposures. It is also noteworthy that the relatively smaller number of males in our cohort likely reduced statistical power.

Our findings were robust to the handling of missing biomarker data. Sensitivity analyses using multivariate imputation by chained equations (MICE) yielded highly consistent results, with strong agreement in PhenoAge estimates and stable associations between CRAR metrics and biological aging across imputation approaches. These results reduce the likelihood that our findings are driven by the choice of imputation method.

Results were also consistent across outcome specifications. When modeling ΔAge as a continuous outcome, associations remained similar in both direction and magnitude across baseline, follow-up, and longitudinal analyses. In addition, analyses across quartiles of CRAR features showed dose-response patterns, indicating graded relationships rather than threshold effects.

An important consideration in longitudinal observational studies is time-varying confounding, particularly from evolving comorbidities, medication use, and acute health events, which may both influence and be influenced by CRAR. To address this, we conducted inverse probability of treatment weighting (IPTW) analyses that account for these dynamic factors. The resulting estimates remained consistent or were strengthened, indicating that the observed associations are not readily explained by time-varying confounding.

We found limited evidence for interaction effects across CRAR domains or with age. Effect estimates were generally close to the null, suggesting that these features contribute largely independently, or with only weak interaction, to biological aging. These findings support a parsimonious modeling approach. Furthermore, adjustment for physical activity and sleep regularity led to only partial attenuation of effect estimates, with substantial associations remaining for CRAR measures.

We also evaluated whether selection related to healthcare engagement may influence inclusion in the analytic sample and the completeness of laboratory-based measurements used to derive PhenoAge. Individuals with higher healthcare utilization are more likely to have repeated laboratory data captured in EHRs, whereas those with limited access may be underrepresented. In our data, inclusion was associated with healthcare engagement, as excluded individuals were younger, had lower income, fewer comorbidities, and a higher prevalence of barriers to care. Adjustment for healthcare utilization, access to care, and the length of follow-up did not materially change the observed associations. Because behavioral phenotypes were derived from wearable data independently of healthcare utilization, whereas selection primarily affects the availability and completeness of laboratory-based outcomes, this form of selection may preferentially influence measurement completeness rather than the underlying associations. Accordingly, while selection related to healthcare engagement may affect sample representativeness, it is less likely to fully account for the observed relationships.

These findings have potential translational implications. Our dual-domain framework, highlighting rhythm intensity as a broadly consistent marker associated with lower odds of accelerated aging alongside more individualized vulnerabilities in timing and regularity, may help guide circadian and activity-related strategies for healthy aging. At the population level, future intervention studies could test whether strategies that enhance rhythm intensity, such as structured physical activity programs, are associated with slower biological aging trajectories. In contrast, individualized strategies that address phase misalignment or rhythm regularity, circadian-aligned nutrition and behavioral scheduling, hold promise for mitigating personal vulnerabilities. Importantly, because these digital phenotypes can be passively derived from consumer-grade wearable devices, scalable, ecologically valid, and continuous monitoring of rest-activity rhythm is now achievable, paving the way toward personalized rest-activity monitoring and intervention research.

Several limitations warrant consideration. First, the AoURP cohort with Fitbit data was predominantly young, female, white participants with a college education^20^. In addition, participants provided their own devices, and the middle-aged to older adults in this cohort were found more physically active than the national average^20^. Despite this relatively healthy and high-activity profile, we still observed robust associations between rest-activity rhythm and biological aging, suggesting that even stronger associations may be present in more sedentary or clinically vulnerable populations. Validation studies in more heterogeneous cohorts across diverse demographic, socioeconomic, and environmental contexts are therefore needed. Second, reverse causality cannot be excluded given the observational design, although we attempted to mitigate this concern by testing time-varying effects. Third, Fitbit step counts may underestimate non-stepping activities such as cycling or resistance training^20^. In addition, day-level valid-wear criterion based on total steps and device-reported wear time does not capture intra-day nonwear bouts. Future work is warranted to incorporate raw accelerometry to derive more precise activity characterization and develop methodologies for consumer device-specific nonwear algorithms, improving exposure ascertainment and rest-activity rhythm estimates. Finally, we acknowledge the limitations of EHR-based outcome ascertainment. Certain events, such as laboratory assessments performed outside routine clinical encounters, may not be fully captured. Nevertheless, prior studies have demonstrated the validity of EHR data for estimating biological age measures^18,34^, supporting the reliability of our approach. We interpret ΔPhenoAge as a longitudinal phenotypic marker of physiological aging trajectories, rather than as a definitive measure of biological aging rate. Changes in PhenoAge may reflect true physiological change, but may also be affected by short-term within-person biomarker variability, EHR measurement timing, and unmeasured confounding. Given the observational design of this study, our findings should be interpreted as associations rather than causal effects.

Despite these limitations, our study brings several notable strengths. Unlike prior investigations that relied on research-grade accelerometers, cross-sectional measurements, or short-term follow-up, our analysis leveraged commercially available devices, a large-scale prospective cohort, and multi-year monitoring periods, thereby enhancing translational potential to real-world applications. In addition, a substantial portion of Fitbit data was collected before participants formally enrolled in AoURP, minimizing observer effects^20^ and reflecting free-living rest-activity patterns. Methodologically, by jointly quantifying between-person variability and within-person temporal stability, we demonstrated that 24 h rhythm estimates captured both population-level associations and individual-level longitudinal robustness. Moreover, sex-stratified models allowed us to rigorously examine sex differences in CRAR-aging association, revealing nuanced, biologically plausible activity patterns. We also found that the associations of CRAR metrics remained robust across extensive sensitivity analyses, including adjustment for physical activity, sleep regularity, health utilization, and the length of follow-up, as well as across different imputation methods. These findings support the robustness of CRAR metrics as indicators of biological aging trajectories. Finally, strict data quality control, extended longitudinal monitoring, and the integration of both scalar metrics and functional principal components provide a comprehensive framework for deep digital phenotyping.

Importantly, PhenoAge in this study is a clinical biomarker-based phenotypic aging measure, distinct from a molecular “biological clock.” It reflects integrated, multi-system physiological dysregulation captured through routine clinical biomarkers. While individual biomarkers are associated with chronic disease risk, the strength of clinical biomarker-based PhenoAge lies in its multivariate construction, which captures system-level aging processes beyond single-marker effects. Previous work has largely established its utility in cross-sectional settings, linking rest-activity patterns to mortality, disease incidence, systemic inflammation, and frailty.

Building on this foundation, this study provides longitudinal evidence that wearable-derived CRAR metrics are associated with biological aging trajectories. By leveraging both conventional CRAR metrics and functional data analysis, we show that rhythm intensity is consistently associated with slower PhenoAge acceleration, while timing and fragmentation show sex-specific associations. These insights support the potential utility of CRAR as a digital biomarker of aging-related physiology and may open a pathway toward scalable, personalized, and temporally targeted interventions. If future interventional studies demonstrate that modifying these digital phenotypes can alter aging trajectories, wearable-guided rest-activity behavioral interventions may contribute to prevention approaches for healthy longevity.

## Methods

### Study participants

We focused on participants from the *All of Us* Research Program (AoURP), a prospective, longitudinal cohort study in the United States funded by the National Institutes of Health, which aims to enroll at least one million individuals and create a rich research biobank integrating diverse health data, including electronic health records (EHR), biospecimens, surveys, wearables, and medical imaging^60^. As of 2024, AoURP had enrolled more than 800,000 participants, providing a uniquely comprehensive resource for multi-modal, population-level longitudinal research. Participants were recruited nationwide. Recruitment strategies were designed to promote inclusion of historically underrepresented populations in biomedical research. Participants aged 18 years or older were enrolled after providing written informed consent at clinics and regional medical centers participating in the AoURP network, including consent for EHR data sharing. AoURP protocol and materials have been approved by the All of Us Institutional Review Board. For this study, we used Registered Tier and Controlled Tier data available through the All of Us Researcher Workbench v8. Analyses were conducted within the secure Researcher Workbench environment, and all reported results comply with the All of Us Data and Statistics Dissemination Policy, which prohibits disclosure of group counts fewer than 20.

From this cohort, we identified 34,217 participants who had contributed Fitbit data through the Bring Your Own Device (BYOD) program, additionally authorized access to their Fitbit data through the AoURP Participant Portal, and provided valid physical measurements (Supplementary Fig. 7). We excluded individuals younger than 18 years during the monitoring period or those who did not meet valid wear day criteria (refer to Fitbit data acquisition and processing section). To ensure continuous and stable assessment of circadian rest-activity rhythmicity, only participants with complete step data for a 12-month period within a given calendar year were retained. Among the 11,698 participants meeting these criteria, we estimated phenotypic age for each calendar year using linked EHR data. Baseline was defined as the earliest date of the valid accelerometer recording period for each participant. Participants were followed from baseline until their last available wearable monitoring date. The final analytic cohort consisted of 2,222 participants with multi-year valid Fitbit data and corresponding biological age estimates, contributing 8,447 person-years of follow-up. This research complied with ethical regulations for research involving human participants.

### Fitbit data acquisition and processing

Participants linked their personal Fitbit accounts to the AoURP Participant Portal through the BYOD program, authorizing access to both prospective and historical data. All data were de-identified according to AoURP privacy protocols, with direct identifiers removed and all date-time fields randomly shifted by 1 to 365 days. Step count data were obtained at both daily and intraday resolutions, with intraday time series used to quantify daily activity patterns and compute rest-activity rhythm metrics. Valid wear days were defined as at least 10 hours of wear time and a minimum of 100 steps, consistent with prior validation studies^20,61^. Fitbit devices have been shown to provide accurate estimates of physical activity and circadian rest-activity rhythm metrics^62^ compared with research-grade accelerometers.

### Biological age estimation

The primary outcome was clinical biomarker-based phenotypic age (PhenoAge), a validated biomarker-based estimate of biological age that predicts morbidity, functional decline, and all-cause mortality^29^. Beyond cross-sectional associations, longitudinal changes in phenotypic age have been shown to be associated with disease and mortality risk, supporting its use as a dynamic marker of aging trajectories^32^. PhenoAge has also been associated with wearable-derived rhythmicity in prior cross-sectional analyses^16,18^. It is derived from nine clinical biomarkers, including albumin, alkaline phosphatase, creatinine, C-reactive protein (CRP), glucose, mean cell volume, red cell distribution width, white blood cell count, and lymphocyte percentage, along with chronological age^29^. These biomarkers were selected using a Cox proportional hazards elastic net model for mortality prediction in the National Health and Nutrition Examination Survey III (NHANES III) cohort^29^. PhenoAge estimation is based on the parametrization of two Gompertz proportional hazards models: one incorporating the selected biomarkers and chronological age to estimate an individual-level mortality risk, and the other using chronological age alone as a baseline. PhenoAge is then derived by equating the predicted mortality risks from both models, reflecting the age at which an individual’s mortality risk is equivalent to that of the reference population. Annual biomarker values were extracted from linked EHR records for each year of Fitbit monitoring (Supplementary Table 18). Biomarker units were harmonized to match the original PhenoAge algorithm. Outliers exceeding five standard deviations (SD) from the mean were manually reviewed and excluded. When up to two biomarkers were missing, values were imputed using the cohort mean; for CRP, which exhibited >50% missing values, the global mean was substituted (Supplementary Table 22). This imputation has been shown to exert minimal influence on downstream analyses. To evaluate the robustness of this approach, we conducted sensitivity analyses using alternative imputation strategies, including multiple imputation by chained equations (MICE). PhenoAge estimates were recalculated using imputed biomarker values, and agreement between estimates derived from mean imputation and MICE was assessed. In addition, we evaluated whether associations between circadian rest-activity rhythm metrics and biological aging were consistent across imputation methods.

To examine associations between rest-activity rhythms and biological aging, we calculated biological age acceleration (ΔAge) as the residual of PhenoAge regressed on chronological age^63^. Participants with ΔAge > 0 were classified as having positive age acceleration (fast aging), and those with ΔAge ≤ 0 as having non-positive age acceleration (slow aging). The final analytic cohort included only individuals with at least two valid PhenoAge measurements temporally aligned with years of valid Fitbit monitoring, thus allowing us to examine longitudinal relationships.

### Assessment of circadian rest-activity rhythms and functional principal components

Circadian rest-activity rhythm (CRAR) metrics quantify the 24-hour structure of behavioral activity patterns, capturing aspects of rhythm intensity, timing, regularity, and overall activity level. These metrics were derived using parametric and non-parametric approaches^41,51,64^ and data-driven functional principal component analysis^65^. For parametric estimation, we applied cosinor analysis to step count time series to quantify the intensity and timing of circadian rest-activity rhythmic fluctuations. The cosinor model has been applied to analyze circadian patterns from melatonin, core body temperature, and wearable-derived rest-activity rhythm^66,67^. Specifically, a 24 h single-component cosinor model was fitted to each individual’s data to estimate the MESOR (Midline Estimating Statistic of Rhythm), amplitude (half the range of rhythmic variation within the cycle), and acrophase (timing of peak activity)^68^. MESOR reflects the rhythm-adjusted mean level of activity across the 24 h cycle and can be interpreted as the overall activity level. Amplitude captures the strength of rhythmic oscillation, and acrophase reflects the timing of peak activity. Model fitting used an iterative least-squares procedure, assuming a fixed circadian period of 24 h, consistent with prior work^18^.

We derived seven non-parametric CRAR metrics that have been significantly associated with aging-related outcomes^15,16,69^. These metrics include M10, M10 start time, L5, L5 start time, relative amplitude (RA), interdaily stability (IS), and intradaily variability (IV). M10 represents the mean activity during the most active 10 consecutive hours within a 24-hour period and serves as a measure of daytime activity intensity. L5 denotes the average activity during the least active 5 consecutive hours, typically corresponding to the nighttime rest period. The timing of these periods is captured by the M10 start time and L5 start time, defined as the clock time at which the M10 and L5 windows begin, respectively. These timing variables provide non-parametric estimates of the activity phase. RA, calculated as (M10 − L5)/(M10 + L5), measures rhythm strength, with higher values indicating greater separation between active and rest phases and lower values suggesting dampened or flattened rhythmicity.

We quantified the degree of rhythm regularity using IS and IV metrics. IS reflects the degree of consistency in activity patterns over multiple days^64,70^, comparing the variance of mean activity at each clock time across days to the total variance. A higher IS value indicates greater day-to-day regularity, whereas a lower IS suggests irregular, unstable daily patterns. IS ranges from 0 to 1. IV captures the degree of fragmentation in activity within a 24 h period. It is calculated as the ratio of the variance of successive differences in activity to the overall variance in daily activity. Lower IV values reflect more consolidated rhythms with distinct separation between active and rest periods, while higher IV values indicate frequent transitions between activity and inactivity, suggestive of fragmented rhythm. IV ranges from 0 to 2. All CRAR metrics were computed from 10 min epoch data, aggregated monthly, and then averaged across each calendar year.

To characterize higher-order latent patterns in daily activity profiles beyond conventional CRAR metrics, we applied functional principal component analysis (fPCA)^65^. Step count trajectories were treated as functional data because daily activity follows a continuous and smooth temporal process^71,72^. For each participant-year, step counts were aggregated into 5-minute epochs, yielding 288 observations per day. We then projected these trajectories onto a nine-term Fourier basis expansion, consistent with prior studies showing that this basis effectively captures diurnal periodicity with minimal overfitting^72,73^. This step reduces high-frequency variation prior to dimensionality reduction, allowing fPCA to capture dominant modes of variability rather than noise-driven fluctuations. Empirical evaluation of 7–10 harmonics demonstrated that harmonics beyond nine terms provided only marginal gains in variance explained while increasing model complexity. The resulting smoothed functions were subjected to fPCA, which decomposes the covariance matrix into an orthonormal basis of eigenfunctions that capture principal component modes of variation. We retained the first four functional principal components (fPCs), which jointly explained 88% of the total variance. Additional components contributed minimal incremental variance and did not improve model stability or interpretability, supporting a parsimonious representation of CRAR variation. Each participant-year was represented by a vector of four orthogonal fPC scores. These scores represent distinct and interpretable dimensions of behavioral rhythmicity at the population level. For consistency across models, each fPC score was standardized and categorized into quartiles for regression and mixed-effects analyses.

### Assessment of sleep metrics

Sleep metrics were derived from the Fitbit daily summary and sequence-level sleep data. Sleep episodes were reconstructed from timestamped sleep stage records by ordering consecutive segments within each participant and defining distinct episodes using gaps of ≥ 30 min. Main sleep episodes were identified using records with is_main_sleep = TRUE, while non-main sleep records (is_main_sleep = FALSE) were used to characterize daytime napping behavior. For each episode, sleep onset was defined as the earliest start time and wake time as the latest end time, and sleep duration was calculated as the elapsed time between onset and wake. Sleep midpoint was defined as the temporal midpoint between sleep onset and wake time.

From the main sleep episodes, we derived total sleep duration, sleep onset time, sleep end time, sleep midpoint, and sleep efficiency, calculated as the ratio of minutes asleep to minutes in bed. Daytime sleep (naps) was identified from non-main sleep episodes using the same episode definition. For each participant, we derived nap count, average nap duration, and mean nap onset time. All sleep metrics were first computed at the daily level and subsequently aggregated to the participant-year level by averaging across valid days. These sleep-derived measures were used to evaluate the correspondence between activity-derived circadian patterns and independently measured sleep behaviors.

### Covariates

We pre-specified a comprehensive set of covariates based on prior evidence and theoretical considerations to account for potential confounding in statistical models^16,18,20^. At initial enrollment, demographic and socioeconomic data were collected using self-identified information provided by participants^74^. Because sex-related differences in rest-activity rhythms and biological aging were expected, sex assigned at birth was considered a priori, included as a covariate in adjusted models, and used for sex-stratified analyses and sex-by-metric interaction tests. Sex assigned at birth was determined from the *All of Us* Research Program self-reported “sex assigned at birth” variable and categorized as male or female for analysis. Self-reported gender identity was also collected, but was not used as an analytic variable in this study. Additional demographic variables included chronological age at the time of Fitbit monitoring, in years, and race. Race was categorized as white participants, black participants, and other race categories combined from Asian, Middle Eastern or North African, more than one race, and not indicated. These categories were combined to support model convergence. Socioeconomic status was assessed through educational attainment (categorized as college or above, high school graduate or GED, and missing/other), employment status (employed, retired, unemployed, or other employment status, including responses not classified in the three primary categories or suppressed low-count categories), and annual household income (US dollars), categorized as high (annual income > $100,000), medium (between $50,000 and $100,000), low (< $50,000), or missing. Body mass index (BMI) was calculated from measurements of height (m) and weight (kg) using weight divided by height^2^ in physical measurements data obtained closest in time to the Fitbit data. BMI categories were defined as normal or underweight ( < 25 kg/m²), overweight (25–29.9 kg/m²), or obese ( ≥ 30 kg/m²). Smoking history was self-reported using concept code 1585857 (“yes” if lifetime cigarette use exceeded 100 cigarettes, otherwise “no”), and current alcohol consumption was coded as a binary variable using concept code 1586198. The presence of major comorbidities, including cardiovascular disease (CVD), type 2 diabetes, and cancer, was ascertained from structured EHR data using predefined International Classification of Diseases (ICD) codes (Supplementary Table 19). In addition, we quantified overall comorbidity burden using the Charlson Comorbidity Index (CCI) using ICD codes^75^ (Supplementary Table 20). CCI was computed as the weighted sum of predefined comorbid conditions and categorized as 0, 1, 2, and ≥ 3. We further assessed medication use as a proxy for treatment burden and underlying health status. We focused on major medication classes relevant to cardiometabolic and neurocognitive aging based on recent research on medication and biological aging^76^, including antihypertensive medications, lipid-lowering agents (statins), antidiabetic medications, and central nervous system (CNS)-active medications (antidepressants and sedatives) (Supplementary Table 21).

Sleep duration was computed as total minutes asleep per day, and sleep onset was defined as the start time of the main sleep episode. We then quantified variability across days per year by calculating the standard deviation of daily sleep duration and the sleep onset timing. For both metrics, we focused on the wearable-derived main sleep episode identified for each day.

### Statistical analysis

Baseline characteristics were summarized as means and SDs for continuous variables and counts with percentages for categorical variables. To characterize temporal patterns in CRAR features, we summarized unadjusted trajectories at years 1, 3, and 5 using year-specific analytic subsets of participants with concurrent wearable and biological aging data. Mean values (± standard error) were computed for fast and slow agers at each time point, and between-group differences were assessed using two-sample *t* tests. To examine cross-sectional associations between CRAR metrics or fPC scores and accelerated biological aging at both baseline and 5-year follow-up, we fitted generalized linear models with a logit link, adjusting for all pre-specified covariates.

To evaluate longitudinal associations between activity metrics and biological age acceleration trajectories, we employed generalized linear mixed-effects models with accelerated aging status as the binary, time-dependent outcome, the metric of interest as a fixed effect, and participant-level random intercepts to account for individual heterogeneity and correlation induced by repeated measures. Models were adjusted for age, sex, race, education, income, employment, lifetime smoking, current alcohol consumption, and comorbidities (cancer, cardiovascular disease, type 2 diabetes). To further assess whether within-person changes in CRAR metrics are associated with concurrent changes in biological aging, we conducted change–change (Δ–Δ) regression analyses. For each participant, changes in CRAR metrics and ΔAge between baseline and follow-up were calculated, and linear regression models were fitted with ΔAge as the outcome and the change in CRAR metrics as predictors. Models were adjusted for the same set of covariates as in the primary analyses.

Age-related trajectories of CRAR metrics were visualized using locally weighted scatterplot smoothing (LOWESS). To quantify uncertainty, we constructed 95% confidence bands using bootstrap resampling (*n* = 1000).

We evaluated the robustness of our findings through multiple sensitivity analyses. First, sex-stratified analyses were performed to evaluate whether associations differed by sex, repeating the regression models separately for males and females and by conducting sex-stratified fPCA. Second, we tested for time-by-metric interaction effects in the linear mixed-effect models to assess whether the associations between behavioral metrics and biological aging change over the follow-up period, thereby assessing potential time-dependent effects. Third, we evaluated effect modification by sex by including sex-by-metric interaction terms in regression models across baseline, follow-up, and longitudinal analyses. Fourth, to assess potential selection related to healthcare engagement, we compared baseline characteristics between individuals included in the longitudinal analysis (i.e., those with wearable data and repeated laboratory measurements) and those excluded (i.e., those with wearable data but without repeated laboratory measurements). Healthcare access and utilization were further characterized using the *All of Us* Healthcare Utilization and Access survey, including domains of recent healthcare contact, access to care, logistical barriers, and financial barriers. Fifth, we additionally adjusted for healthcare utilization and the length of follow-up in statistical models to account for differential healthcare engagement and observation time. Sixth, to evaluate the impact of outcome specification, we conducted sensitivity analyses modeling ΔAge as a continuous outcome and examining associations across quartiles of key CRAR features. Seventh, we evaluated interaction terms between CRAR domains (e.g., intensity, timing, and stability) as well as between CRAR metrics and chronological age. Eighth, we examined baseline wearable-derived features as predictors of PhenoAge at follow-up. Finally, we quantified the correspondence between fPCs and Fitbit-derived sleep metrics to assess cross-domain consistency in CRAR, sleep, and behavioral patterns.

To account for time-varying confounding, we additionally conducted sensitivity analyses using inverse probability of treatment weighting (IPTW). For each wearable metric, binary exposure groups were defined based on quartiles. For fPC1-4, MESOR, amplitude, RA, M10, and IS, the lowest quartile was defined as low exposure and the remaining as high (reference), whereas for acrophase, L5, M10 start time, L5 start time, and IV, the highest quartile was defined as high exposure and the remaining as low (reference). Stabilized weights were estimated using logistic models incorporating demographic, socioeconomic, and time-varying covariates, including comorbidities, medication use, mental health status, and acute health events. Weighted logistic regression models were then fitted to estimate associations between wearable-derived CRAR metrics and accelerated biological aging.

To complement the fPCA-based analysis, we conducted additional analyses using partial least squares (PLS), a supervised dimensionality reduction approach. PLS components were defined to maximize covariance between the predictor matrix and the outcome. The number of PLS components was selected as four based on a 10-fold cross-validated receiver operating characteristic–area under the curve (ROC-AUC). For each fold, models were trained on the training set and evaluated on the held-out set, and the number of components corresponding to the optimal cross-validated performance was retained. To compare the latent structures identified by fPCA and PLS, we quantified the pairwise Pearson correlations. We then evaluated associations of both fPCA- and PLS-derived components with accelerated biological aging consistent with the primary analysis framework.

For direct comparison of effect sizes across heterogeneous metrics, all CRAR and fPC variables were categorized into quartile groups prior to model fitting. This facilitated consistent quartile definitions across metrics, while model coefficients represented differences in outcome between quartile categories relative to the reference group. Consistent with the AoURP Data and Statistics Dissemination Policy, model results with cell sizes ≤20 observations were not reported. All analyses were conducted in Python (version 3.12) within the AoURP Workbench using pandas 2.3.3, numpy 2.4.3, scipy 1.17.1, statsmodels 0.14.6, scikit-learn 1.8.0, scikit-fda 0.10.1, matplotlib 3.10.8, CosinorPy 3.1, pymer4 0.8.2, seaborn 0.13.2, and pingouin 0.6.1, with parallel processing on four nodes when applicable. Statistical significance was defined as a two-sided *p*-value < 0.05.

### Reporting summary

Further information on research design is available in the Nature Portfolio Reporting Summary linked to this article.

## Supplementary information

Supplementary Information

Reporting Summary

Transparent Peer Review file

## Source data

Source Data

## Data Availability

Source data are provided with this paper. These source data include aggregate and model-derived data. Participant-level data are controlled and are not publicly shared to protect participant privacy. Access to All of Us data may be requested through the All of Us Researcher Workbench (https://workbench.researchallofus.org/login) after institutional Data Use and Registration Agreement approval, registration, identity verification, completion of Responsible Conduct of Research training, and attestation to the Data User Code of Conduct and data-use agreement. Source data are provided in this paper.

## Code availability

Analysis code supporting the findings of this study can be made available to approved users of the All of Us Researcher Workbench platform by contacting the corresponding author (jinjooshim@hsph.harvard.edu). Access is limited to approved Workbench users because the code is designed to run within the All of Us controlled-access environment and may reference controlled-access data tables and workspace-specific workflows. Requests will be reviewed within 10 business days, and any use of the code must comply with All of Us data use policies and applicable institutional requirements.
