# Supplementary material for: Longitudinal digital phenotyping of activity rhythms and biological aging using commercial wearables
Source: Nat Commun. 2026 Aug 22;17:10155. doi: 10.1038/s41467-026-76147-6 (PMC13601627; doi:10.1038/s41467-026-76147-6)
Supplement: Supplementary file 1 [file 41467_2026_76147_MOESM1_ESM.pdf]

SUPPLEMENTAL MATERIAL

Longitudinal Digital Phenotyping of Activity Rhythms and Biological Aging Using Commercial Wearables

Jinjoo Shim[1*], Jukka-Pekka Onnela[1]

[1]Department of Biostatistics, Harvard T.H. Chan School of Public Health, Boston, MA, USA

***Corresponding author:**
Jinjoo Shim
Department of Biostatistics
Harvard T.H. Chan School of Public Health
655 Huntington Avenue
Boston, MA 02115, USA
Email: jinjooshim@hsph.harvard.edu

Table of Contents

Supplementary Fig. 1 Correlation between biological age (PhenoAge) and chronological age.
Supplementary Fig. 2 Baseline 24-hour activity profiles stratified by biological age acceleration and chronological age group.
Supplementary Fig. 3 Age-associated trends in circadian rest-activity rhythm (CRAR) metrics by biological age acceleration stratified by sex.
Supplementary Fig. 4 Pearson correlation matrix between functional principal components (fPC1-fPC4) and CRAR metrics.
Supplementary Fig. 5 Pearson correlation matrix between functional principal components (fPC1-fPC4) and sleep metrics.
Supplementary Fig. 6 Functional principal components (fPCs) of 24-hour activity profiles and their association with biological age acceleration stratified by sex.
Supplementary Fig. 7 Cohort selection flowchart.
Supplementary Fig. 8 Correlation of PhenoAge estimates derived from mean imputation and multivariate imputation by chained equations (MICE).
Supplementary Fig. 9 Correlation between functional principal components (fPCs) and partial least squares (PLS) components.
Supplementary Fig. 10 Longitudinal stability of CRAR metrics.
Supplementary Table 1. Sex-stratified associations between functional and CRAR metrics and accelerated biological aging at baseline and follow-up.
Supplementary Table 2. Sex-stratified longitudinal associations between functional and CRAR metrics and biological aging.
Supplementary Table 3. Sex interaction effects for functional and CRAR metrics on accelerated biological aging.
Supplementary Table 4. Change-change regression analysis of the association between within-person changes in CRAR metrics and biological aging (ΔAge).
Supplementary Table 5. IPTW-weighted associations between wearable-derived CRAR metrics and accelerated biological aging.
Supplementary Table 6. Comparison of longitudinal associations between CRAR metrics and biological aging across imputation methods.
Supplementary Table 7. Sensitivity analyses of associations between functional and CRAR metrics and continuous ΔAge.
Supplementary Table 8. Sensitivity analyses of associations between wearable feature quartiles and continuous ΔAge.
Supplementary Table 9. Sensitivity analyses of associations between wearable feature quartiles and accelerated biological aging.
Supplementary Table 10. Baseline functional and CRAR metrics as predictors of PhenoAge at follow-up.
Supplementary Table 11. Interaction effects between CRAR metrics and chronological age.
Supplementary Table 12. Interaction effects in the linear mixed-effect models of longitudinal biological age acceleration.
Supplementary Table 13. Associations of CRAR metrics with biological aging before and after adjustment for moderate-to-vigorous physical activity (MVPA).
Supplementary Table 14. Associations of CRAR metrics with biological aging before and after adjustment for MVPA and sleep regularity.
Supplementary Table 15. Baseline characteristics of the study population by inclusion and exclusion status.
Supplementary Table 16. Sensitivity analyses adjusting for healthcare engagement and the length of follow-up.
Supplementary Table 17. Associations between fPCA- and PLS-derived components and accelerated biological aging at baseline.
Supplementary Table 18. Codes used for identifying lab biomarkers in the All of Us Research Program.

Supplementary Table 19. International Classification of Diseases (ICD) codes used for identifying comorbidities in the All of Us Research Program.
Supplementary Table 20. ICD-9 and ICD-10 coding algorithms for Charlson Comorbidity Index (CCI) score.
Supplementary Table 21. Medication groups defined using RxNorm ancestor concepts.
Supplementary Table 22. Missing rate of biomarkers.

Supplementary Fig. 1 Correlation between biological age (PhenoAge) and chronological age. (a) Scatter plots illustrate the strong linear correlation between PhenoAge and chronological age among all participants and stratified by sex. Pearson correlation coefficients (*r*) and corresponding p-values are shown for each subgroup. **(b)** Histograms display the distribution of biological age acceleration (ΔAge). **(c)** Histogram displays chronological age. Source data are provided as a Source Data file.

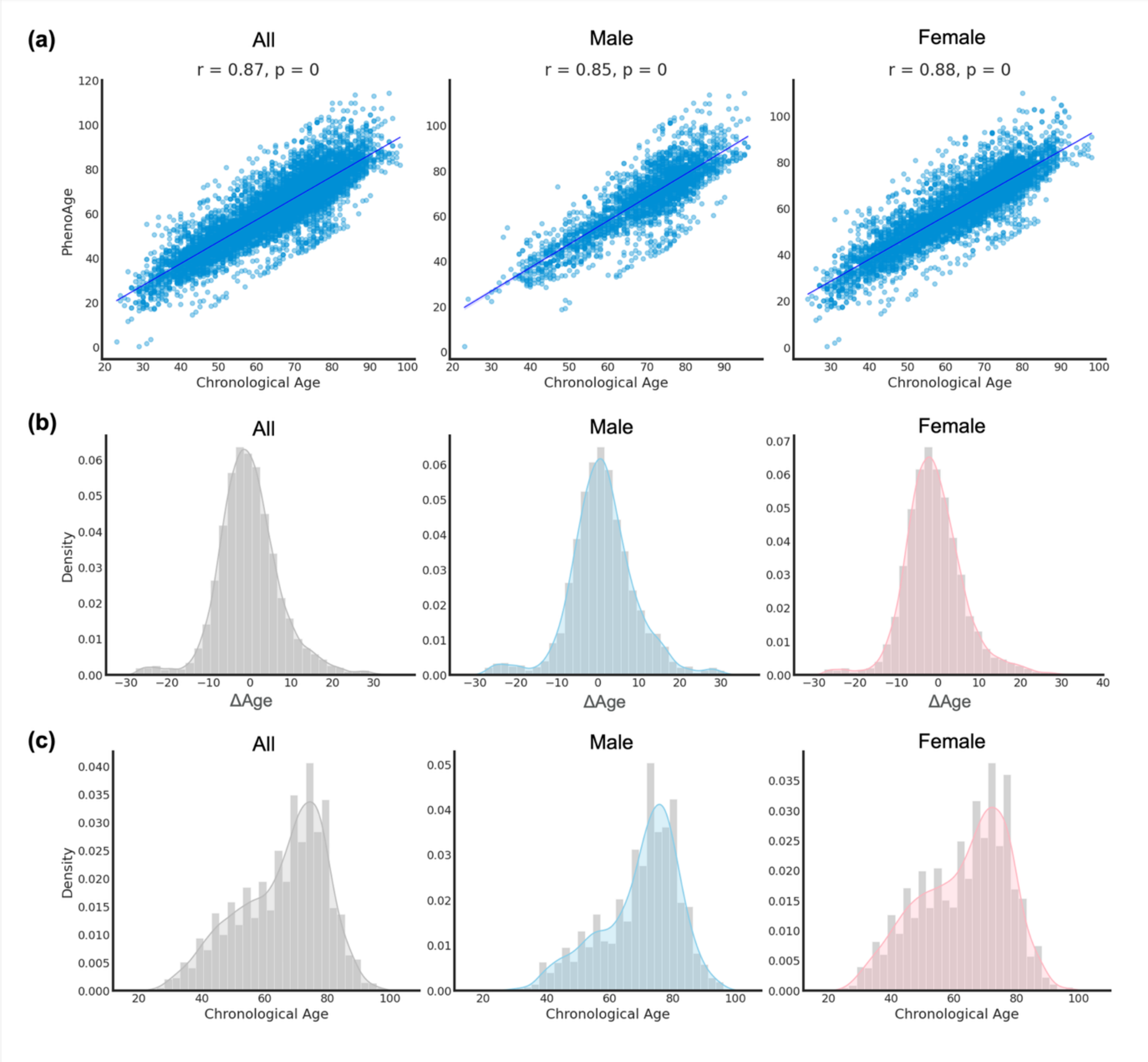

Supplementary Fig. 2 Baseline 24-hour activity profiles stratified by biological age acceleration and chronological age group. Panels depict **(a)** slow agers and **(b)** fast agers, classified according to biological age acceleration. Within each panel, heatmaps (left) illustrate the distribution of activity intensity over the 24-hour cycle, and corresponding mean activity curves (right) summarize diurnal rest-activity patterns for four chronological age strata (40s (<49 years), 50s (50-59), 60s (60-69), and 70+ (≥70 years)). Source data are provided as a Source Data file.

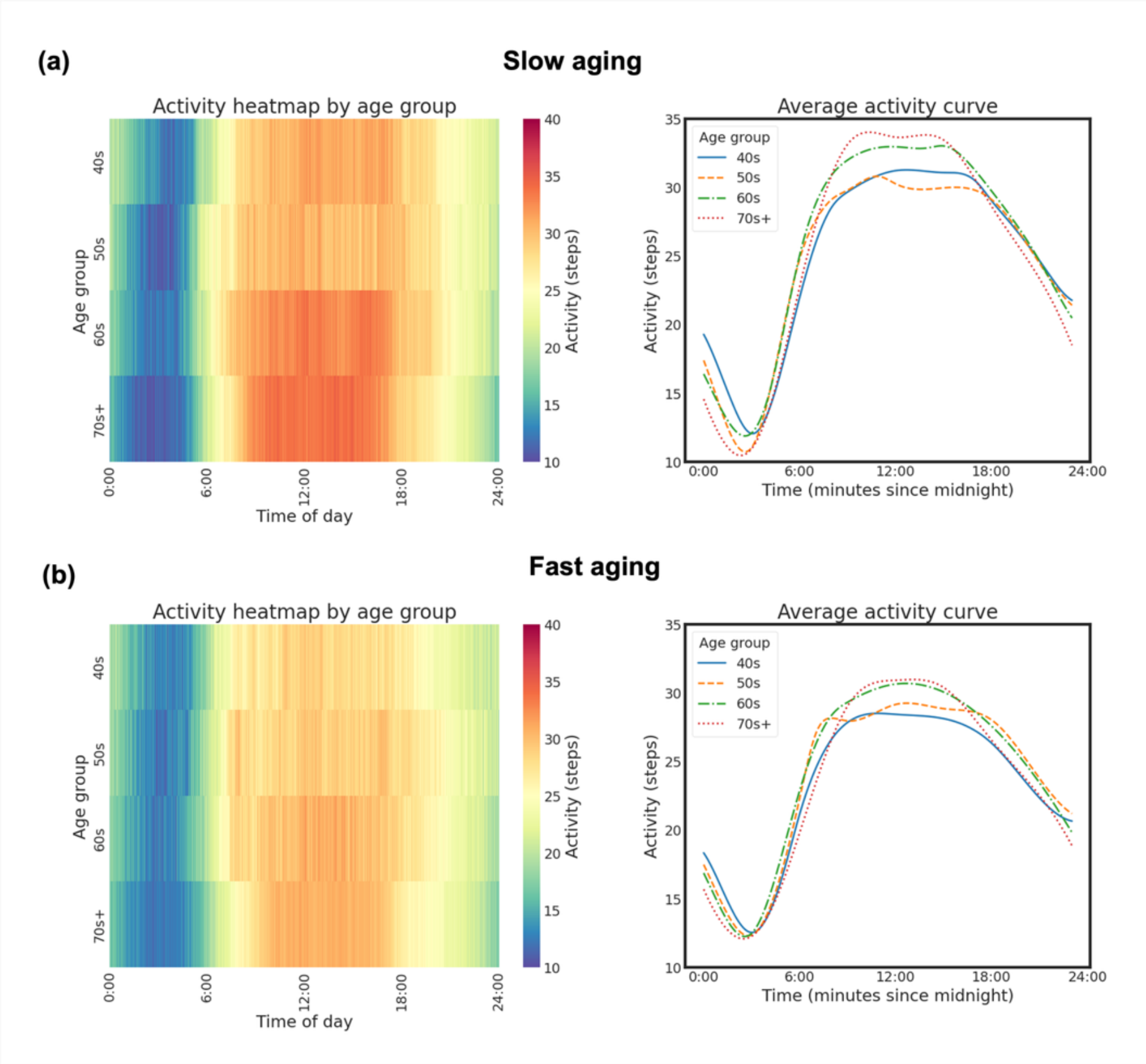

Supplementary Fig. 3 Age-associated trends in circadian rest-activity rhythm (CRAR) metrics by biological age acceleration stratified by sex. Each panel displays a distinct CRAR metric for **(a)** males and **(b)** females. Data points are color-coded by biological aging status at baseline (blue=slow aging, red=fast aging). Locally weighted scatterplot smoothing (LOWESS) lines are overlaid to visualize smoothed trajectories by aging group. Source data are provided as a Source Data file.

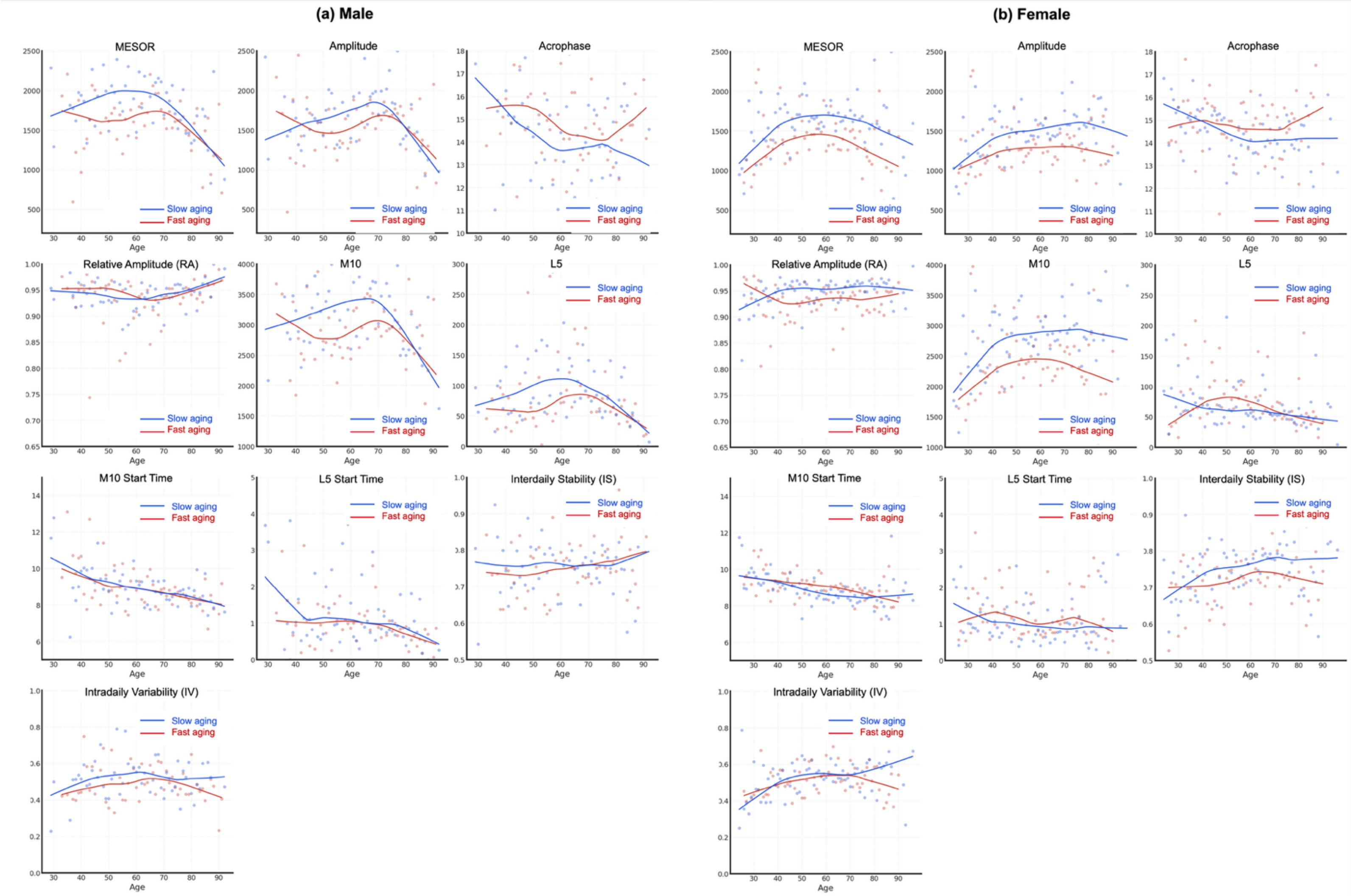

Supplementary Fig. 4 Pearson correlation matrix between functional principal components (fPC1-fPC4) and CRAR metrics. Color scale represents the strength and direction of correlations (red=positive, blue=negative), with numerical values indicating Pearson correlation coefficients (r). Source data are provided as a Source Data file.

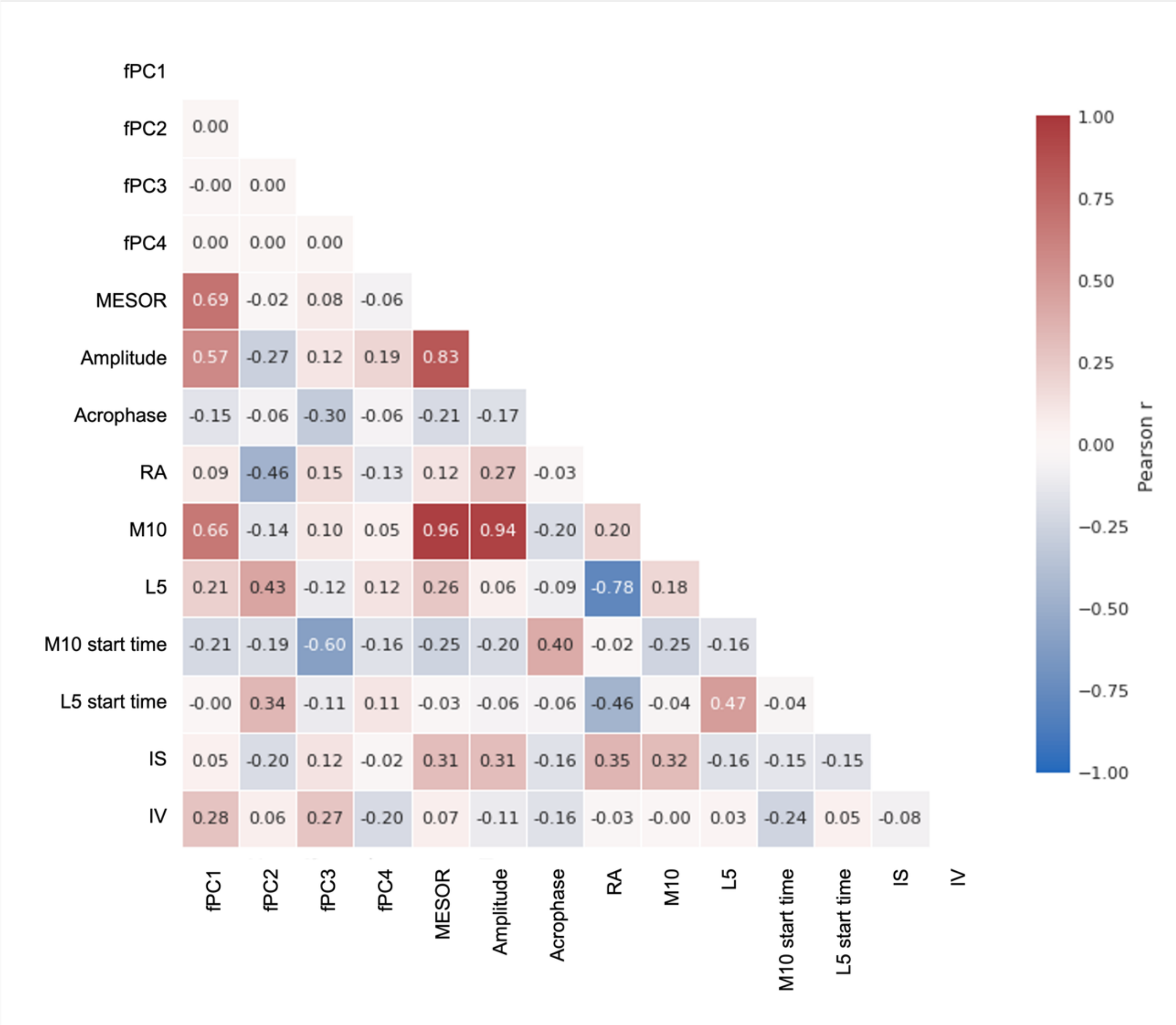

Supplementary Fig. 5 Pearson correlation matrix between functional principal components (fPC1-fPC4) and sleep metrics. Color scale represents the strength and direction of correlations (red=positive, blue=negative), with numerical values indicating Pearson correlation coefficients (r). Source data are provided as a Source Data file.

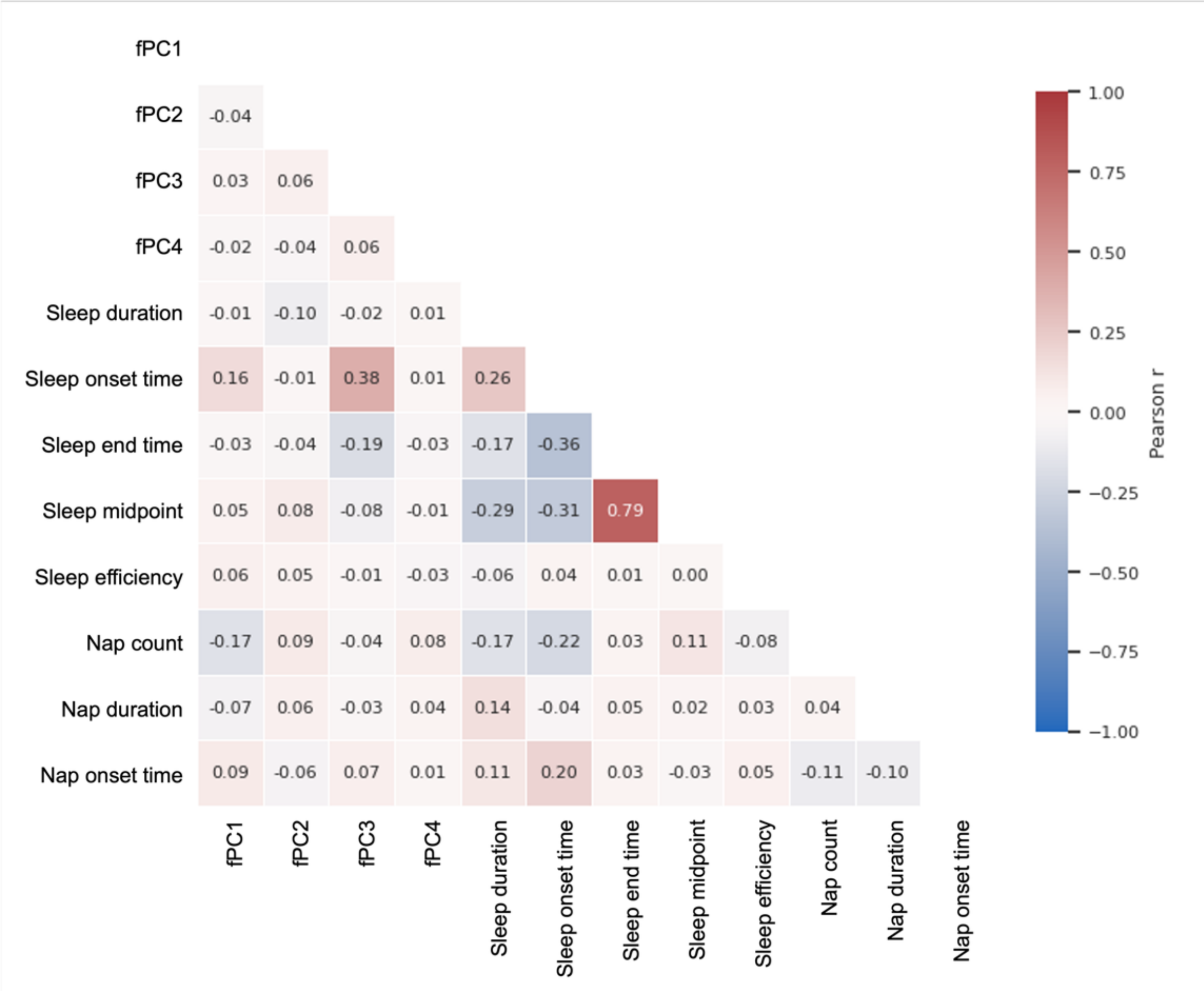

Supplementary Fig. 6 Functional principal components (fPCs) of 24-hour activity profiles and their association with biological age acceleration stratified by sex. **(a)** Mean 24-hour activity profiles are plotted separately by the sign of each fPC score, illustrating the predominant temporal patterns captured by each component. **(b)** Line plots depict the fPC x mean score within each biological aging group at baseline (blue=slow aging, red=fast aging). Source data are provided as a Source Data file.

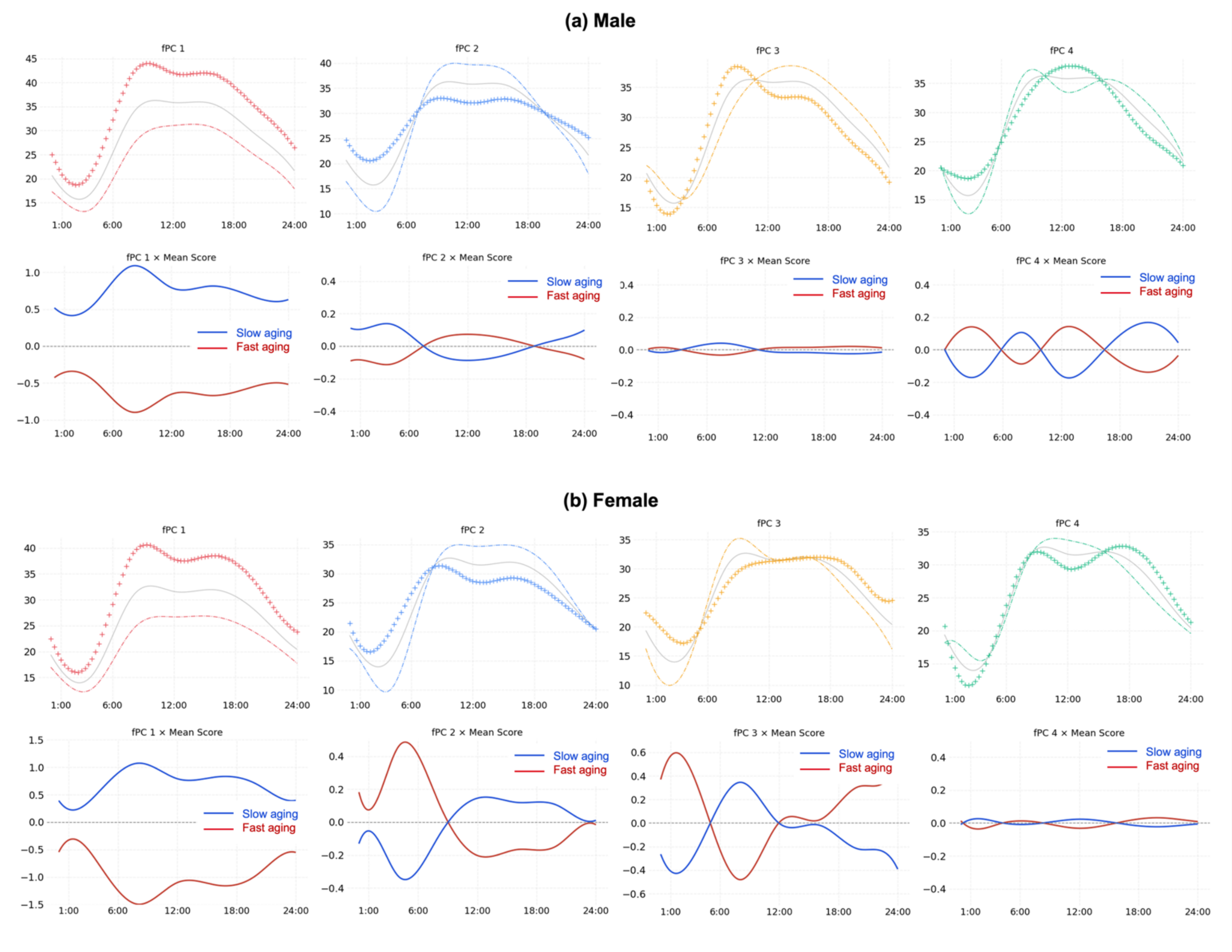

Supplementary Fig. 7 Cohort selection flowchart.

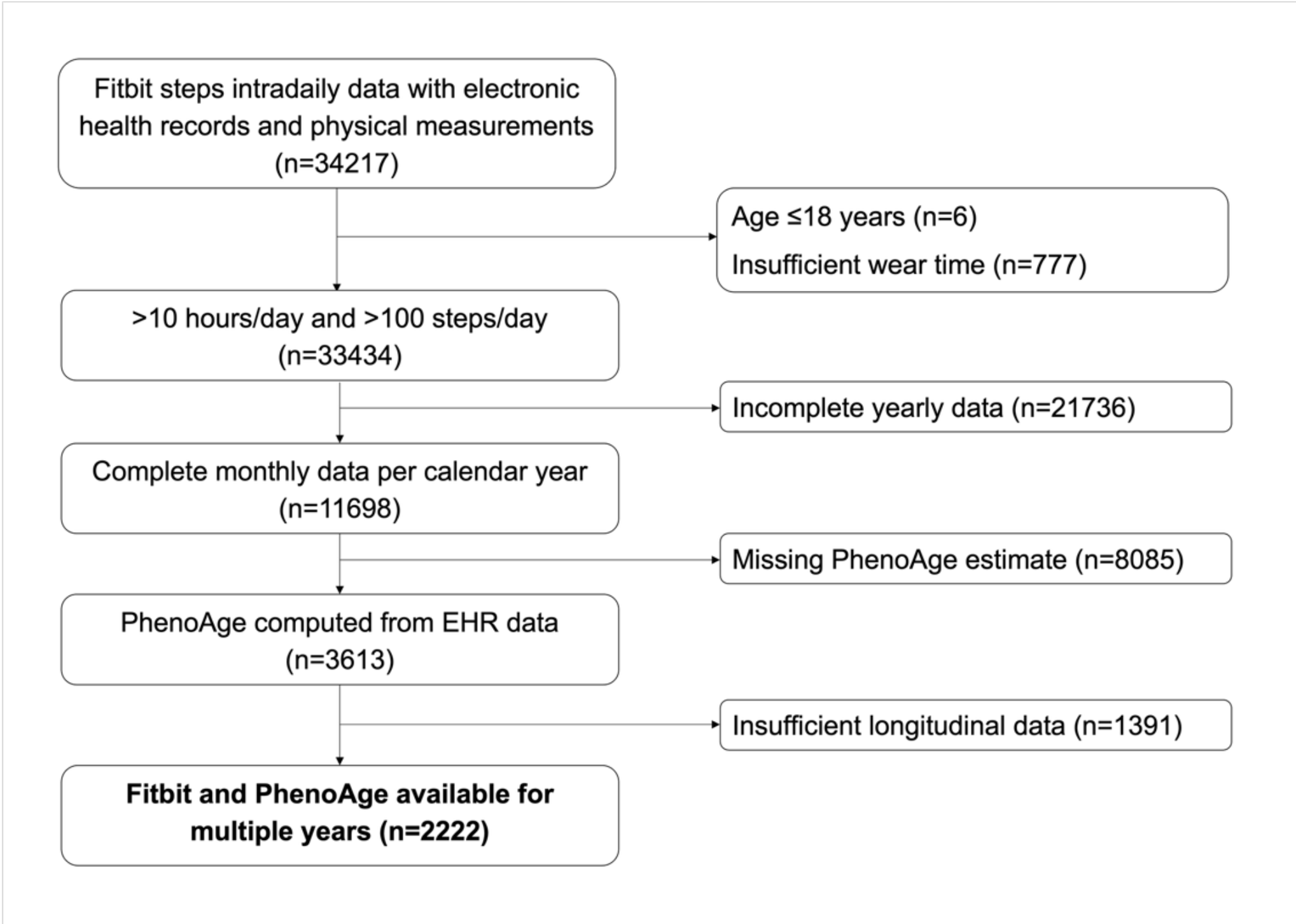

Supplementary Fig. 8 Correlation of PhenoAge estimates derived from mean imputation and multivariate imputation by chained equations (MICE). Pearson correlation coefficients (r). MICE comparison included n=8,415 person-year records with both mean-imputed and MICE-derived PhenoAge estimates. Source data are provided as a Source Data file.

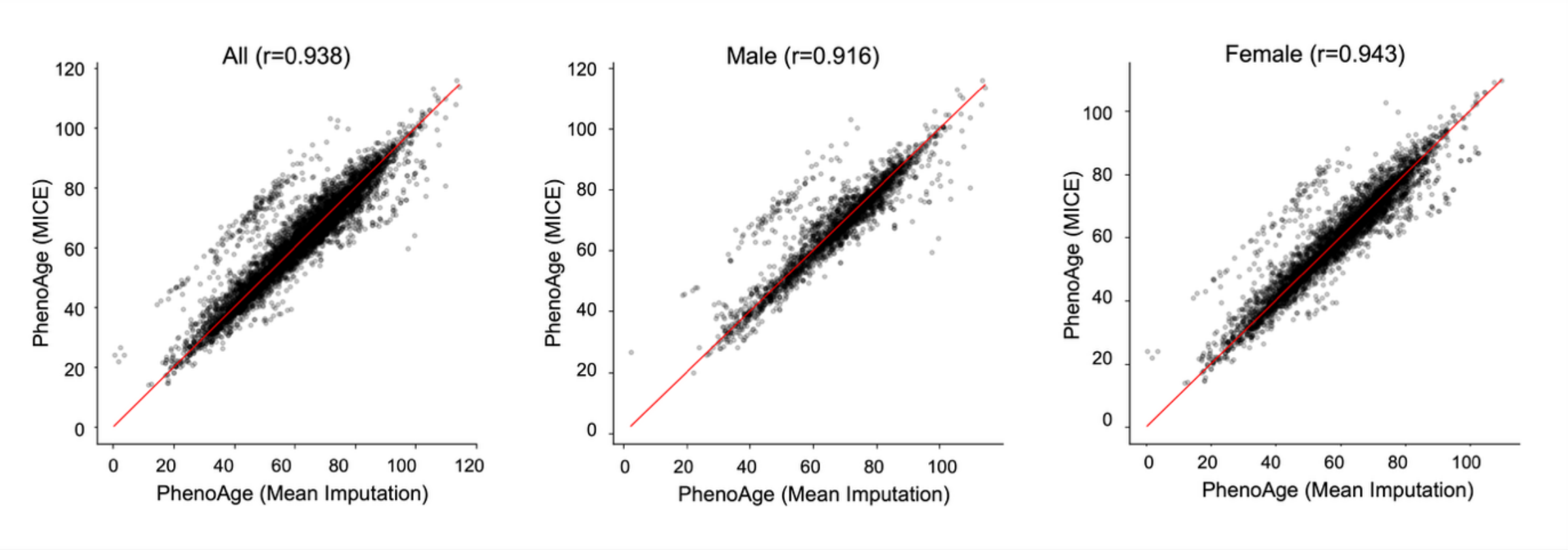

Supplementary Fig. 9 Correlation between functional principal components (fPCs) and partial least squares (PLS) components. Source data are provided as a Source Data file.

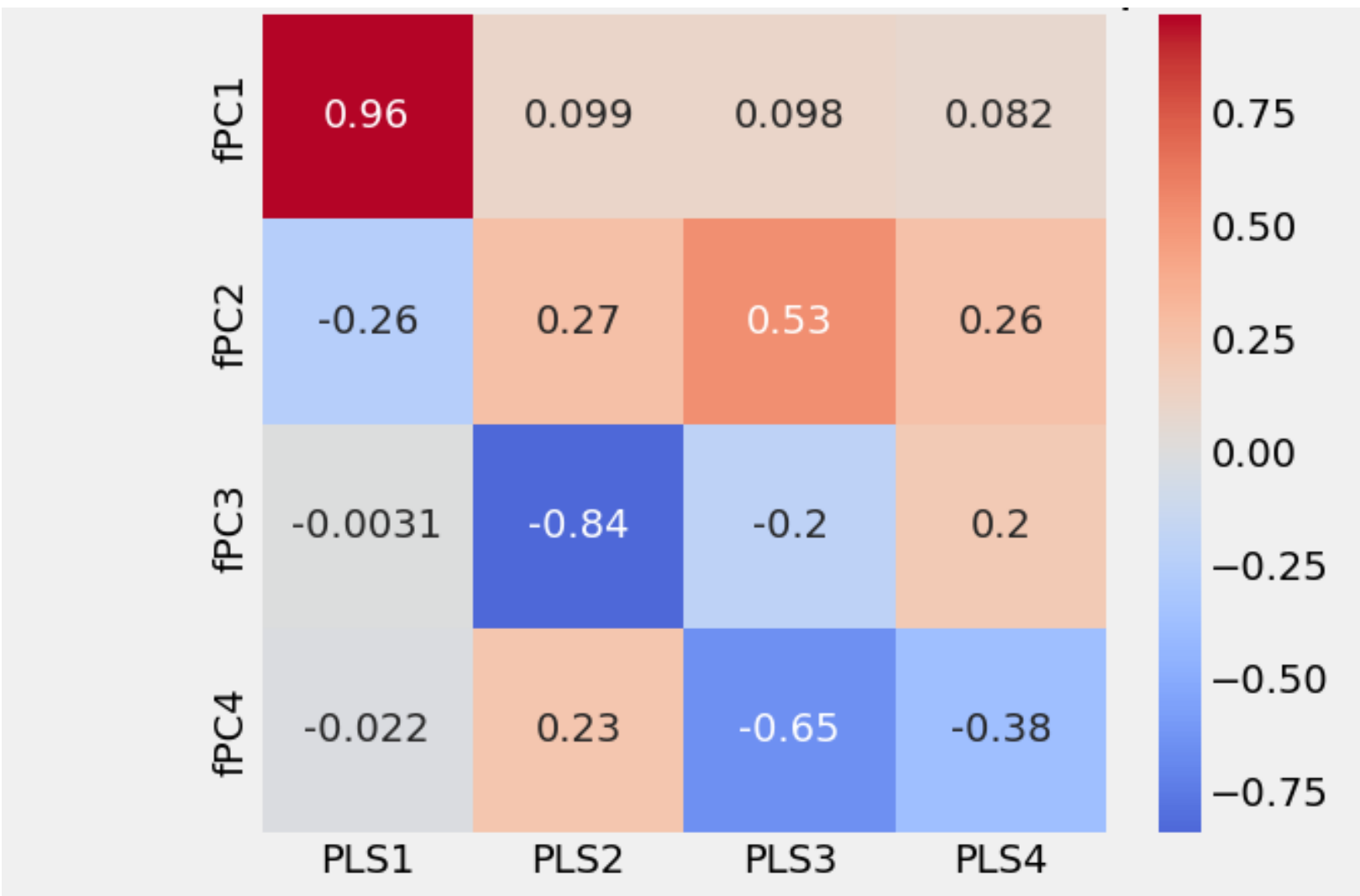

Supplementary Fig. 10 Longitudinal stability of CRAR metrics. Scatter plots comparing baseline and follow-up values for circadian rest-activity rhythm (CRAR) metrics. Each point represents an individual participant. Red lines indicate linear regression fits, and dashed lines represent the identity line. Pearson correlation coefficients (r) and P values are shown. P values are from two-sided Pearson correlation tests. Source data are provided as a Source Data file.

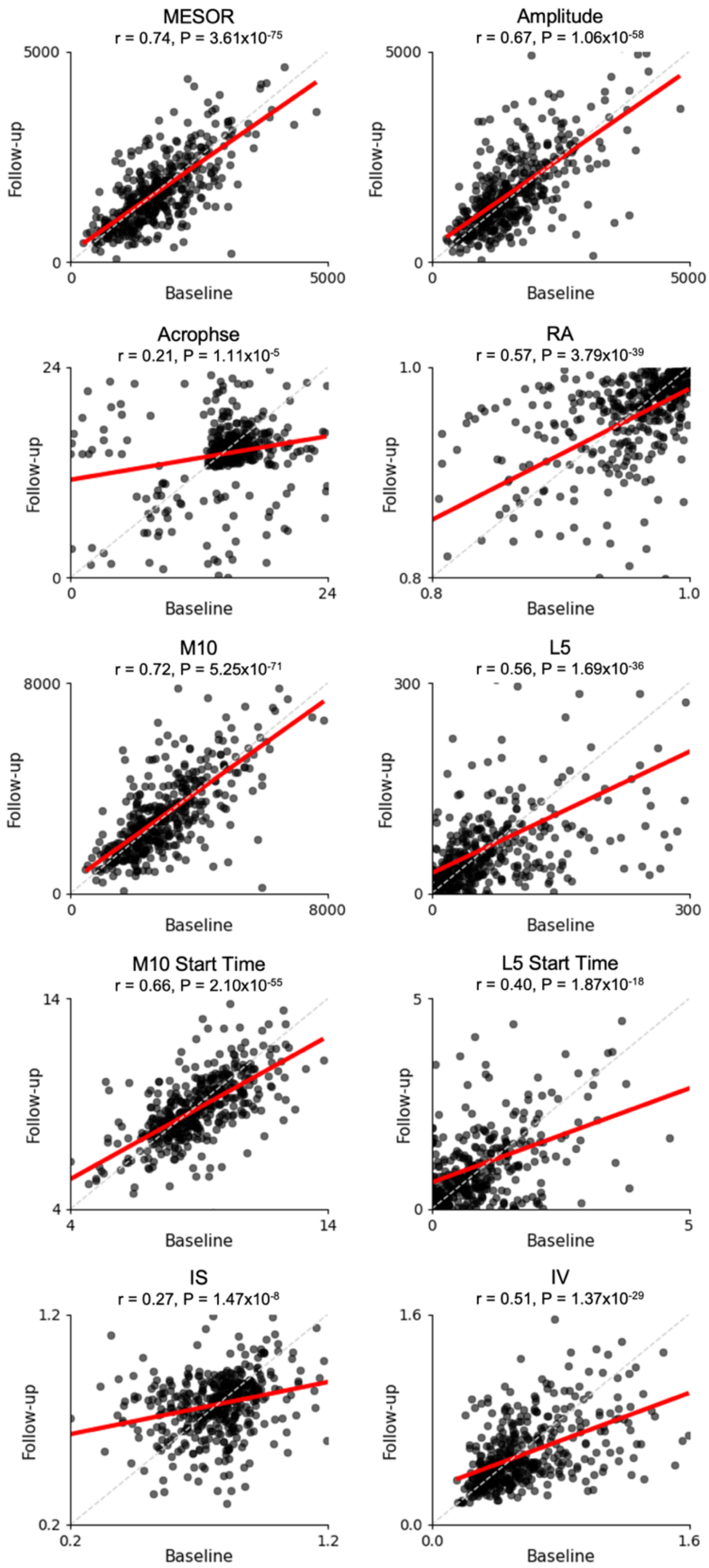

Supplementary Table 1. Sex-stratified associations between functional and CRAR metrics and accelerated biological aging at baseline and follow-up.

	Male				Female			
Features	Baseline OR (95% CI)	P value	Follow-up OR (95% CI)	P value	Baseline OR (95% CI)	P value	Follow-up OR (95% CI)	P value
fPC1	**0.834 (0.715, 0.973)**	**0.021**	**0.675 (0.503, 0.898)**	**0.007**	**0.797 (0.722, 0.880)**	**0.000**	**0.835 (0.702, 0.995)**	**0.044**
fPC2	0.976 (0.851, 1.120)	0.730	0.949 (0.760, 1.184)	0.641	1.084 (0.982, 1.197)	0.108	1.000 (0.843, 1.186)	1.000
fPC3	0.997 (0.870, 1.144)	0.969	0.836 (0.668, 1.046)	0.118	0.929 (0.842, 1.024)	0.140	0.966 (0.820, 1.139)	0.682
fPC4	1.074 (0.935, 1.235)	0.313	0.913 (0.730, 1.141)	0.424	0.971 (0.879, 1.072)	0.556	0.989 (0.840, 1.164)	0.892
MESOR	**0.836 (0.719, 0.973)**	**0.021**	0.801 (0.619, 1.036)	0.091	**0.762 (0.690, 0.841)**	**7.29×10^{-8}**	**0.766 (0.639, 0.918)**	**0.004**
Amplitude	0.902 (0.779, 1.044)	0.168	0.943 (0.734, 1.212)	0.647	**0.734 (0.664, 0.812)**	**1.70×10^{-9}**	**0.782 (0.655, 0.935)**	**0.007**
Acrophase	1.143 (0.993, 1.315)	0.063	0.982 (0.778, 1.24)	0.88	**1.112 (1.008, 1.226)**	**0.033**	1.041 (0.879, 1.234)	0.64
Relative amplitude (RA)	0.972 (0.84, 1.125)	0.7	0.979 (0.775, 1.237)	0.86	**0.764 (0.692, 0.843)**	**8.99×10^{-8}**	**0.827 (0.694, 0.986)**	**0.034**
M10	**0.847 (0.73, 0.983)**	**0.029**	0.835 (0.647, 1.078)	0.167	**0.733 (0.662, 0.810)**	**1.59×10^{-9}**	**0.741 (0.616, 0.890)**	**0.001**
L5	0.94 (0.814, 1.085)	0.396	1.003 (0.8, 1.259)	0.977	**1.11 (1.006, 1.224)**	**0.037**	1.037 (0.875, 1.230)	0.674
M10 start time	0.964 (0.836, 1.111)	0.609	1.213 (0.959, 1.534)	0.108	**1.131 (1.024, 1.249)**	**0.015**	1.101 (0.925, 1.311)	0.281
L5 start time	0.983 (0.855, 1.129)	0.804	1.202 (0.959, 1.507)	0.11	**1.181 (1.069, 1.313)**	**0.001**	1.079 (0.913, 1.274)	0.373
Interdaily stability (IS)	0.975 (0.842, 1.128)	0.73	0.913 (0.722, 1.156)	0.45	**0.854 (0.775, 0.941)**	**0.001**	0.868 (0.732, 1.028)	0.101
Intradaily variability (IV)	0.898 (0.779, 1.035)	0.138	0.847 (0.674, 1.064)	0.153	0.966 (0.877, 1.065)	0.489	0.924 (0.779, 1.096)	0.366

Odds ratios (ORs) and 95% confidence intervals (CIs) were estimated using sex-stratified multivariable logistic regression models. P values are from two-sided Wald tests of the regression coefficients. Models were adjusted for age, race, education, income, employment, lifetime smoking, current alcohol consumption, and comorbidities (cancer, cardiovascular disease, and type 2 diabetes). Significant associations ($P<0.05$) are shown in bold.

Supplementary Table 2. Sex-stratified longitudinal associations between functional and CRAR metrics and biological aging.

	Male		Female	
**Features**	**OR (95% CI)**	**P value**	**OR (95% CI)**	**P value**
fPC1	0.855 (0.717, 1.020)	0.082	**0.816 (0.719, 0.925)**	**0.002**
fPC2	0.997 (0.845, 1.177)	0.975	1.105 (0.976, 1.251)	0.114
fPC3	1.049 (0.888, 1.240)	0.573	0.911 (0.807, 1.029)	0.133
fPC4	1.132 (0.956, 1.340)	0.152	0.955 (0.847, 1.078)	0.455
MESOR	**0.708 (0.563, 0.890)**	**0.003**	**0.615 (0.525, 0.720)**	**1.61×10^{-9}**
Amplitude	0.797 (0.632, 1.006)	0.056	**0.560 (0.478, 0.656)**	**6.06×10^{-13}**
Acrophase	1.122 (0.941, 1.338)	0.200	**1.176 (1.031, 1.343)**	**0.016**
Relative amplitude (RA)	0.928 (0.755, 1.141)	0.478	**0.669 (0.577, 0.776)**	**1.05×10^{-7}**
M10	**0.708 (0.564, 0.889)**	**0.003**	**0.590 (0.503, 0.693)**	**1.09×10^{-10}**
L5	0.840 (0.681, 1.037)	0.104	**1.172 (1.013, 1.355)**	**0.033**
M10 start time	0.902 (0.730, 1.113)	0.334	**1.240 (1.068, 1.441)**	**0.005**
L5 start time	1.007 (0.822, 1.232)	0.949	1.107 (0.956, 1.281)	0.174
Interdaily stability (IS)	1.051 (0.872, 1.267)	0.603	**0.856 (0.750, 0.978)**	**0.022**
Intradaily variability (IV)	0.830 (0.676, 1.019)	0.075	0.940 (0.815, 1.085)	0.399

Odds ratios (ORs) and 95% confidence intervals (CIs) were estimated using sex-stratified generalized linear mixed-effects regression models with participant-level random intercepts. P values are from two-sided Wald tests of the regression coefficients. Models were adjusted for age, race, education, income, employment, lifetime smoking, current alcohol consumption, and comorbidities (cancer, cardiovascular disease, and type 2 diabetes). Significant associations ($P<0.05$) are shown in bold.

Supplementary Table 3. Sex interaction effects for functional and CRAR metrics on accelerated biological aging.

	Baseline		Follow-up		Longitudinal	
Features	OR (95% CI)	P value	OR (95% CI)	P value	OR (95% CI)	P value
fPC1*sex	1.049 (0.881, 1.249)	0.592	1.316 (0.884, 1.958)	0.176	1.044 (0.837, 1.303)	0.701
fPC2*sex	0.886 (0.751, 1.046)	0.153	0.968 (0.657, 1.427)	0.871	0.865 (0.697, 1.073)	0.186
fPC3*sex	1.114 (0.946, 1.313)	0.196	1.181 (0.812, 1.719)	0.384	1.214 (0.980, 1.504)	0.076
fPC4*sex	1.122 (0.951, 1.325)	0.172	1.223 (0.828, 1.805)	0.312	1.218 (0.983, 1.510)	0.072
MESOR*sex	1.055 (0.885, 1.259)	0.550	0.983 (0.735, 1.315)	0.908	1.115 (0.846, 1.470)	0.440
Amplitude*sex	**1.207 (1.013, 1.438)**	**0.035**	1.106 (0.825, 1.481)	0.501	**1.427 (1.084, 1.879)**	**0.011**
Acrophase*sex	0.978 (0.827, 1.156)	0.793	**0.705 (0.530, 0.937)**	**0.016**	0.924 (0.736, 1.161)	0.497
RA*sex	**1.298 (1.092, 1.543)**	**0.003**	1.309 (0.986, 1.738)	0.063	**1.531 (1.178, 1.988)**	**0.001**
M10*sex	1.115 (0.935, 1.330)	0.226	1.017 (0.757, 1.365)	0.913	1.235 (0.938, 1.626)	0.133
L5*sex	**0.827 (0.697, 0.982)**	**0.030**	**0.741 (0.559, 0.984)**	**0.038**	**0.697 (0.537, 0.905)**	**0.007**
M10 start*sex	**0.806 (0.681, 0.953)**	**0.012**	0.917 (0.693, 1.214)	0.547	**0.714 (0.548, 0.930)**	**0.013**
L5 start*sex	**0.816 (0.690, 0.964)**	**0.017**	1.045 (0.792, 1.379)	0.754	0.872 (0.679, 1.120)	0.283
IS*sex	1.162 (0.978, 1.380)	0.087	1.249 (0.940, 1.660)	0.126	1.203 (0.949, 1.526)	0.127
IV*sex	0.926 (0.782, 1.097)	0.375	0.859 (0.650, 1.134)	0.283	0.878 (0.681, 1.133)	0.318

Odds ratios (ORs) and 95% confidence intervals (CIs) were estimated from multivariable logistic regression or generalized linear mixed-effects regression models including metric-by-sex interaction terms, as appropriate for the analysis time point. P values are from two-sided Wald tests of the interaction terms. Models were adjusted for age, race, education, income, employment, lifetime smoking, current alcohol consumption, and comorbidities (cancer, cardiovascular disease, and type 2 diabetes). Significant associations ($P<0.05$) are shown in bold.

Supplementary Table 4. Change-change regression analysis of the association between within-person changes in CRAR metrics and biological aging (ΔAge).

Features	Beta Coefficient (95% CI)	P value
MESOR	**-0.427 (-0.793, -0.060)**	**0.023**
Amplitude	**-0.627 (-0.992, -0.261)**	**0.001**
Acrophase	0.185 (-0.184, 0.554)	0.326
Relative amplitude (RA)	-0.101 (-0.467, 0.266)	0.591
M10	**-0.374 (-0.940, -0.207)**	**0.002**
L5	-0.173 (-0.542, 0.197)	0.360
M10 start time	-0.046 (-0.416, 0.324)	0.807
L5 start time	**0.420 (0.053, 0.786)**	**0.025**
Interdaily stability (IS)	**-0.370 (-0.737, -0.002)**	**0.048**
Intradaily variability (IV)	0.272 (-0.094, 0.638)	0.145

Beta coefficients and 95% confidence intervals (CIs) were estimated using multivariable linear regression models of within-person change in biological age acceleration. P values are from two-sided t-tests of the regression coefficients. Models were adjusted for age, sex, race, education, income, employment, lifetime smoking, current alcohol consumption, and comorbidities (cancer, CVD, type 2 diabetes). Significant associations (P<0.05) are shown in bold.

Supplementary Table 5. IPTW-weighted associations between wearable-derived CRAR metrics and accelerated biological aging.

Features	OR (95% CI)	P value
fPC1	**0.740 (0.639, 0.858)**	**6.58×10^{-5}**
fPC2	1.129 (0.973, 1.309)	0.109
fPC3	0.965 (0.833, 1.119)	0.639
fPC4	**1.163 (1.002, 1.349)**	**0.047**
MESOR	**0.635 (0.553, 0.728)**	**9.50×10^{-11}**
Amplitude	**0.603 (0.525, 0.692)**	**7.00×10^{-13}**
Acrophase	**1.213 (1.057, 1.391)**	**0.006**
Relative amplitude (RA)	**0.704 (0.613, 0.807)**	**5.62×10^{-7}**
M10	**0.603 (0.525, 0.692)**	**6.98×10^{-13}**
L5	1.106 (0.964, 1.269)	0.150
M10 start time	**1.152 (1.004, 1.322)**	**0.043**
L5 start time	**1.154 (1.006, 1.324)**	**0.041**
Interdaily stability (IS)	**0.799 (0.697, 0.917)**	**0.001**
Intradaily variability (IV)	0.863 (0.731, 1.021)	0.080

Odds ratios (ORs) and 95% confidence intervals (CIs) were estimated using inverse probability of treatment weighting (IPTW). Binary exposure groups were defined based on quartiles for each metric. Weighting models incorporated demographic, socioeconomic, and time-varying covariates, including age, sex, race, education, income, employment, lifetime smoking, current alcohol consumption, comorbidities (cancer, cardiovascular disease, and type 2 diabetes), medication use (antihypertensive, statins, antidiabetic, and CNS-active medications), depression, and acute health events (hospitalization and emergency room visits). P values are from two-sided Wald tests of the regression coefficients. Significant associations ($P<0.05$) are shown in bold.

Supplementary Table 6. Comparison of longitudinal associations between CRAR metrics and biological aging across imputation methods.

Features	Mean Imputation OR (95% CI)	P value	MICE OR (95% CI)	P value
fPC1	**0.832 (0.757, 0.915)**	**1.43×10^{-4}**	**0.818 (0.737, 0.884)**	**1.49×10^{-5}**
fPC2	1.059 (0.966, 1.162)	0.219	**1.108 (1.026, 1.195)**	**0.008**
fPC3	0.938 (0.857, 1.028)	0.172	0.962 (0.892, 1.036)	0.306
fPC4	1.016 (0.928, 1.113)	0.726	1.061 (0.984, 1.144)	0.124
MESOR	**0.654 (0.574, 0.744)**	**1.39×10^{-10}**	**0.649 (0.597, 0.707)**	**1.23×10^{-23}**
Amplitude	**0.623 (0.553, 0.702)**	**7.53×10^{-15}**	**0.637 (0.586, 0.693)**	**5.58×10^{-26}**
Acrophase	**1.150 (1.036, 1.277)**	**0.009**	1.034 (0.953, 1.122)	0.423
RA	**0.764 (0.682, 0.855)**	**3.05×10^{-6}**	**0.709 (0.649, 0.775)**	**3.01×10^{-14}**
M10	**0.612 (0.536, 0.698)**	**3.13×10^{-13}**	**0.632 (0.581, 0.688)**	**1.92×10^{-26}**
L5	1.055 (0.945, 1.178)	0.343	**1.138 (1.043, 1.242)**	**0.004**
M10 start	**1.133 (1.004, 1.278)**	**0.043**	1.061 (0.971, 1.159)	0.188
L5 start	1.052 (0.943, 1.173)	0.363	**1.090 (1.000, 1.188)**	**0.049**
IS	**0.895 (0.809, 0.991)**	**0.033**	**0.843 (0.780, 0.910)**	**1.40×10^{-5}**
IV	0.891 (0.792, 1.002)	0.054	0.954 (0.881, 1.034)	0.468

Odds ratios (ORs) and 95% confidence intervals (CIs) were estimated using generalized linear mixed-effects regression models under each imputation strategy. P values are from two-sided Wald tests of the regression coefficients. Models were adjusted for age, sex, race, education, income, employment, lifetime smoking, current alcohol consumption, and comorbidities (cancer, CVD, type 2 diabetes). MICE, multivariate imputation by chained equations. Significant associations (P<0.05) are shown in bold.

Supplementary Table 7. Sensitivity analyses of associations between functional and CRAR metrics and continuous ΔAge.

	Baseline		Follow-up		Longitudinal	
Features	Beta (95% CI)	P value	Beta (95% CI)	P value	Beta (95% CI)	P value
fPC1	**-0.925 (-1.220, -0.629)**	**9.42×10^{-10}**	0.459 (-0.175, 1.093)	0.156	**-0.251 (-0.404, -0.097)**	**0.001**
fPC2	-0.245 (-0.041, 0.531)	0.093	0.380 (-0.245, 1.005)	0.233	-0.085 (-0.234, 0.076)	0.262
fPC3	-0.101 (-0.385, 0.183)	0.486	0.102 (-0.504, 0.708)	0.741	-0.051 (-0.197, 0.095)	0.493
fPC4	0.001 (-0.286, 0.288)	0.994	0.504 (-0.103, 1.110)	0.104	0.019 (-0.126, 0.164)	0.799
MESOR	**-1.050 (-1.346, -0.754)**	**3.23×10^{-12}**	**-1.258 (-1.760, -0.756)**	**9.03×10^{-7}**	**-0.756 (-0.947, -0.564)**	**1.01×10^{-14}**
Amplitude	**-1.039 (-1.333, -0.746)**	**3.90×10^{-12}**	**-1.241 (-1.743, -0.738)**	**1.27×10^{-6}**	**-0.786 (-0.973, -0.599)**	**1.75×10^{-16}**
Acrophase	**0.437 (0.146, 0.728)**	**0.003**	**0.502 (0.008, 0.997)**	**0.047**	0.096 (-0.046, 0.238)	0.187
RA	**-0.791 (-1.087, -0.496)**	**1.36×10^{-7}**	-0.410 (-0.927, 0.107)	0.120	**-0.401 (-0.577, -0.225)**	**7.98×10^{-6}**
M10	**-1.165 (-1.459, -0.872)**	**8.99×10^{-15}**	**-1.316 (-1.816, -0.817)**	**2.66×10^{-7}**	**-0.871 (-1.063, -0.679)**	**6.03×10^{-19}**
L5	0.186 (-0.109, 0.481)	0.567	-0.219 (-0.724, 0.286)	0.394	-0.063 (-0.237, 0.111)	0.478
M10 start	**0.303 (0.006, 0.600)**	**0.046**	0.419 (-0.078, 0.915)	0.098	**0.192 (0.010, 0.374)**	**0.039**
L5 start	**0.454 (0.160, 0.748)**	**0.002**	0.179 (-0.316, 0.673)	0.478	0.117 (-0.052, 0.285)	0.175
IS	**-0.468 (-0.762, -0.173)**	**0.002**	**-0.721 (-1.221, -0.221)**	**0.005**	**-0.270 (-0.416, -0.123)**	**3.04×10^{-4}**
IV	-0.155 (-0.448, 0.138)	0.300	-0.203 (-0.701, 0.295)	0.812	-0.020 (-0.186, 0.145)	0.809

Beta coefficients and 95% confidence intervals (CIs) were estimated using linear regression or mixed-effects models for continuous biological age acceleration (ΔAge), as appropriate for the analysis time point. P values are from two-sided t-tests of the regression coefficients. Models were adjusted for age, sex, race, education, income, employment, lifetime smoking, current alcohol consumption, and comorbidities (cancer, CVD, type 2 diabetes). Longitudinal model includes a random intercept. Significant associations (P<0.05) are shown in bold.

Supplementary Table 8. Sensitivity analyses of associations between wearable feature quartiles and continuous ΔAge.

Quartile Groups	Amplitude Beta (95% CI)	P value	Acrophase Beta (95% CI)	P value	IS Beta (95% CI)	P value
**Baseline**						
Q1	Reference		Reference		Reference	
Q2	**-1.940 (-2.852, -1.029)**	**3.12×10^{-5}**	0.447 (-0.479, 1.372)	0.344	-0.182 (-1.098, 0.735)	0.698
Q3	**-3.088 (-4.006, -2.169)**	**5.43×10^{-11}**	**1.037 (0.110, 1.963)**	**0.028**	**-0.936 (-1.866, -0.006)**	**0.049**
Q4	**-3.106 (-4.030, -2.183)**	**5.37×10^{-11}**	**1.233 (0.312, 2.155)**	**0.009**	**-1.322 (-2.252, -0.391)**	**0.005**
**Follow up**						
**Quartile Groups**	**Amplitude Beta (95% CI)**	**P value**	**Acrophase Beta (95% CI)**	**P value**	**IS Beta (95% CI)**	**P value**
Q1	Reference		Reference		Reference	
Q2	**-3.008 (-4.552, -1.464)**	**1.42×10^{-4}**	0.314 (-1.224, 1.852)	0.689	**-1.782 (-3.314, -0.250)**	**0.023**
Q3	**-2.822 (-4.390, -1.254)**	**4.36×10^{-4}**	**2.030 (0.478, 3.583)**	**0.010**	**-1.752 (-3.317, -0.186)**	**0.028**
Q4	**-4.245 (-5.823, -2.667)**	**1.68×10^{-7}**	1.114 (-0.443, 2.670)	0.160	**-2.390 (-3.965, -0.814)**	**0.003**
**Longitudinal**						
**Quartile Groups**	**Amplitude Beta (95% CI)**	**P value**	**Acrophase Beta (95% CI)**	**P value**	**IS Beta (95% CI)**	**P value**
Q1	Reference		Reference		Reference	
Q2	**-1.428 (-1.898, -0.958)**	**2.60×10^{-9}**	0.028 (-0.428, 0.483)	0.905	**-0.697 (-1.098, -0.296)**	**0.001**
Q3	**-1.995 (-2.511, -1.480)**	**3.32×10^{-14}**	0.156 (-0.313, 0.625)	0.515	**-0.641 (-1.061, -0.222)**	**0.003**
Q4	**-2.440 (-3.021, -1.859)**	**1.85×10^{-16}**	0.269 (-0.181, 0.718)	0.242	**-0.911 (-1.370, -0.452)**	**1.00×10^{-4}**

Beta coefficients and 95% confidence intervals (CIs) were estimated using regression models with wearable-derived features categorized into quartiles. P values are from two-sided tests of the regression coefficients. Models were adjusted for age, sex, race, education, income, employment, lifetime smoking, current alcohol consumption, and comorbidities (cancer, CVD, type 2 diabetes). Significant associations ($P<0.05$) are shown in bold.

Supplementary Table 9. Sensitivity analyses of associations between wearable feature quartiles and accelerated biological aging.

Baseline						
**Quartile Groups**	**Amplitude OR (95% CI)**	**P value**	**Acrophase OR (95% CI)**	**P value**	**IS OR (95% CI)**	**P value**
Q1	Reference		Reference		Reference	
Q2	**0.622 (0.484, 0.800)**	**2.15×10^{-4}**	1.176 (0.914, 1.514)	0.207	0.929 (0.725, 1.192)	0.564
Q3	**0.484 (0.375, 0.625)**	**2.82×10^{-8}**	1.061 (0.823, 1.367)	0.648	0.847 (0.658, 1.090)	0.196
Q4	**0.476 (0.368, 0.616)**	**1.70×10^{-8}**	**1.456 (1.132, 1.871)**	**0.003**	**0.690 (0.535, 0.890)**	**0.004**
**Follow up**						
**Quartile Groups**	**Amplitude OR (95% CI)**	**P value**	**Acrophase OR (95% CI)**	**P value**	**IS OR (95% CI)**	**P value**
Q1	Reference		Reference		Reference	
Q2	**0.447 (0.289, 0.690)**	**2.84×10^{-4}**	1.210 (0.790, 1.853)	0.380	0.701 (0.460, 1.066)	0.097
Q3	**0.472 (0.303, 0.733)**	**8.45×10^{-4}**	1.470 (0.959, 2.253)	0.077	0.658 (0.428, 1.012)	0.057
Q4	**0.339 (0.215, 0.532)**	**2.68×10^{-6}**	1.337 (0.870, 2.055)	0.185	0.742 (0.482, 1.142)	0.175
**Longitudinal**						
**Quartile Groups**	**Amplitude OR (95% CI)**	**P value**	**Acrophase OR (95% CI)**	**P value**	**IS OR (95% CI)**	**P value**
Q1	Reference		Reference		Reference	
Q2	**0.438 (0.308, 0.624)**	**4.58×10^{-6}**	1.296 (0.920, 1.825)	0.138	0.831 (0.611, 1.129)	0.236
Q3	**0.334 (0.229, 0.486)**	**1.11×10^{-8}**	1.021 (0.719, 1.450)	0.909	0.803 (0.584, 1.103)	0.175
Q4	**0.256 (0.171, 0.386)**	**5.37×10^{-11}**	**1.582 (1.127, 2.223)**	**0.008**	0.739 (0.527, 1.104)	0.079

Odds ratios (ORs) and 95% confidence intervals (CIs) were estimated using logistic regression or generalized linear mixed-effects regression models with wearable-derived features categorized into quartiles, as appropriate for the analysis time point. P values are from two-sided Wald tests of the regression coefficients. Models were adjusted for age, sex, race, education, income, employment, lifetime smoking, current alcohol consumption, and comorbidities (cancer, CVD, type 2 diabetes). Significant associations ($P<0.05$) are shown in bold.

Supplementary Table 10. Baseline functional and CRAR metrics as predictors of PhenoAge at follow-up.

Features	Beta Coefficient (95% CI)	P value
fPC1	**-0.976 (-1.502, -0.449)**	**2.92×10^{-4}**
fPC2	-0.085 (-0.566, 0.397)	0.730
fPC3	-0.358 (-0.828, 0.113)	0.136
fPC4	-0.098 (-0.568, 0.372)	0.681
MESOR	**-1.057 (-1.580, -0.535)**	**7.83×10^{-5}**
Amplitude	**-0.931 (-1.446, -0.415)**	**4.18×10^{-4}**
Acrophase	0.272 (-0.226, 0.770)	0.284
Relative amplitude (RA)	**-0.593 (-1.098, -0.088)**	**0.021**
M10	**-1.198 (-1.720, -0.676)**	**7.60×10^{-6}**
L5	0.130 (-0.365, 0.625)	0.607
M10 start time	**0.649 (0.144, 1.154)**	**0.012**
L5 start time	0.368 (-0.121, 0.857)	0.140
Interdaily stability (IS)	-0.479 (-0.976, 0.017)	0.058
Intradaily variability (IV)	**-0.512 (-1.008, -0.015)**	**0.043**

Beta coefficients and 95% confidence intervals (CIs) were estimated using multivariable linear regression models with follow-up PhenoAge as the outcome. P values are from two-sided t-tests of the regression coefficients. Models were adjusted for age, sex, race, education, income, employment, lifetime smoking, current alcohol consumption, and comorbidities (cancer, CVD, type 2 diabetes). Significant associations (P<0.05) are shown in bold.

Supplementary Table 11. Interaction effects between CRAR metrics and chronological age.

Interaction Term	Domain	OR (95% CI)	P value
Amplitude*Acrophase	Intensity*Timing	0.997 (0.909, 1.093)	0.945
Amplitude*IS	Intensity*Stability	0.910 (0.823, 1.005)	0.063
Acrophase*IS	Timing*Stability	1.039 (0.947, 1.139)	0.417
Amplitude*Age	Intensity*Age	0.999 (0.990, 1.008)	0.807
Acrophase*Age	Timing*Age	1.003 (0.995, 1.011)	0.508
IS*Age	Stability*Age	1.009 (1.001, 1.018)	0.024

Odds ratios (ORs) and 95% confidence intervals (CIs) were estimated from models including metric-by-chronological age interaction terms. P values are from two-sided Wald tests of the interaction terms. Models were adjusted for age, sex, race, education, income, employment, lifetime smoking, current alcohol consumption, and comorbidities (cancer, CVD, type 2 diabetes).

Supplementary Table 12. Interaction effects in the linear mixed-effect models of longitudinal biological age acceleration.

Features	OR (95% CI)
fPC1*year	1.218 (1.073, 1.382)
fPC2*year	1.062 (0.943, 1.196)
fPC3*year	1.007 (0.894, 1.135)
fPC4*year	1.052 (0.930, 1.191)
MESOR*year	1.036 (0.976, 1.100)
Amplitude*year	1.017 (0.958, 1.079)
Acrophase*year	0.978 (0.919, 1.040)
Relative amplitude (RA)*year	0.995 (0.938, 1.057)
M10*year	1.050 (0.989, 1.114)
L5*year	1.032 (0.972, 1.096)
M10 start time*year	1.020 (0.961, 1.083)
L5 start time*year	0.990 (0.933, 1.051)
Interdaily stability (IS)*year	0.996 (0.936, 1.060)
Intradaily variability (IV)*year	1.020 (0.960, 1.084)

Odds ratios (ORs) and 95% confidence intervals (CIs) were estimated using generalized linear mixed-effects regression models with participant-level random intercepts and metric-by-year interaction terms. Models were adjusted for age, sex, race, education, income, employment, lifetime smoking, current alcohol consumption, and comorbidities (cancer, CVD, type 2 diabetes).

Supplementary Table 13. Associations of CRAR metrics with biological aging before and after adjustment for moderate-to-vigorous physical activity (MVPA).

Features	Unadjusted ($\widehat{\beta_1}$)	MVPA-adjusted ($\widehat{\beta_2}$)	Attenuation (%)
fPC1	-0.184	-0.134	27.2
fPC2	0.057	0.052	8.8
fPC3	-0.064	-0.038	40.6
fPC4	0.016	0.014	12.5
MESOR	-0.424	-0.450	-6.1
Amplitude	-0.473	-0.472	0.2
Acrophase	0.140	0.134	4.3
RA	-0.269	-0.274	-1.9
M10	-0.491	-0.525	-6.9
L5	0.054	0.068	-25.9
M10 start	0.125	0.083	33.6
L5 start	0.051	0.057	-11.8
IS	-0.111	-0.094	15.3
IV	-0.115	-0.088	23.5

Supplementary Table 14. Associations of CRAR metrics with biological aging before and after adjustment for MVPA and sleep regularity.

Features	Unadjusted ($\widehat{\beta_1}$)	MVPA- and sleep-adjusted ($\widehat{\beta_2}$)	Attenuation (%)
fPC1	-0.184	-0.157	14.7
fPC2	0.057	0.052	8.8
fPC3	-0.064	-0.064	0.0
fPC4	0.016	0.009	43.8
MESOR	-0.424	-0.459	-8.3
Amplitude	-0.473	-0.452	4.4
Acrophase	0.140	0.117	16.4
RA	-0.269	-0.301	-11.9
M10	-0.491	-0.542	-10.4
L5	0.054	0.096	-77.8
M10 start	0.125	0.077	38.4
L5 start	0.051	0.035	31.4
IS	-0.111	-0.118	-6.3
IV	-0.115	-0.088	23.5

Supplementary Table 15. Baseline characteristics of the study population by inclusion and exclusion status.

Mean (SD) or n (%)	Included (n=2222)	Excluded (n=2249)	P value
Age, years	60.62 (14.01)	55.67 (15.09)	**1.59×10^{-29}**
PhenoAge, years	59.90 (15.72)	55.07 (17.08)	**1.45×10^{-22}**
Sex			0.107
Female	1522 (68.5)	1604 (71.3)	
Male	693 (31.2)	634 (28.2)	
Race			**3.67×10^{-5}**
white participants	1839 (82.8)	1763 (78.4)	
black participants	134 (6.0)	130 (5.8)	
Other categories combined	249 (11.2)	356 (15.8)	
BMI, kg/m^2	29.84 (6.98)	30.18 (8.04)	0.139
Education			0.237
College or above	2084 (93.8)	2100 (93.4)	
12 years or GED	119 (5.4)	122 (5.4)	
Income			**1.27×10^{-5}**
High	1015 (45.7)	894 (39.8)	
Medium	669 (30.1)	683 (30.4)	
Low	395 (17.8)	522 (23.2)	
Missing	143 (6.4)	150 (6.7)	
Employment			**4.12×10^{-11}**
Employed	1260 (56.7)	1403 (62.4)	
Retired	732 (32.9)	534 (23.7)	
Unemployed	159 (7.2)	207 (9.2)	
Other employment status	71 (3.2)	105 (4.7)	
Current alcohol consumption			0.228
Yes	788 (35.5)	758 (33.7)	
No	1434 (64.5)	1496 (66.3)	
Drinking			0.033
Yes	2168 (97.6)	2169 (96.4)	
No	54 (2.4)	80 (3.6)	
Comorbidities			
Cancer	207 (9.3)	155 (6.9)	0.007
CVD	247 (11.1)	185 (8.2)	0.001
Type 2 diabetes	212 (9.5)	33 (1.5)	**4.17×10^{-32}**
Daily steps	7715.83 (3429.04)	6566.24 (3175.47)	**1.81×10^{-22}**
Sleep duration, hours	6.39 (1.67)	6.24 (1.65)	0.013
No recent healthcare contact	181 (8.1)	274 (12.2)	**1.01×10^{-5}**
Limited healthcare access	518 (23.3)	706 (31.4)	**1.7×10^{-9}**
Logistic barrier	429 (19.3)	555 (24.7)	**1.73×10^{-5}**
Finance barrier	312 (14.0)	446 (19.8)	**3.08×10^{-7}**
Follow-up duration	6.84 (2.2)	5.15 (2.5)	**5.08×10^{-10}**

Values are mean (SD) for continuous variables and n (%) for categorical variables. P values were calculated using two-sided two-sample t-tests for continuous variables and two-sided chi-square tests for categorical variables, as appropriate. Sex category counts do not sum to total N because sex other categories other than male or female were suppressed according to the AoURP Data and Statistics Dissemination Policy.

Supplementary Table 16. Sensitivity analyses adjusting for healthcare engagement and the length of follow-up.

Features	Model 1 OR (95% CI)	P value	Model 2 OR (95% CI)	P value	Model 3 OR (95% CI)	P value
fPC1	**0.832 (0.757, 0.915)**	**1.43×10^{-4}**	**0.834 (0.752, 0.925)**	**0.001**	**0.823 (0.747, 0.905)**	**6.90×10^{-5}**
fPC2	1.059 (0.966, 1.162)	0.219	1.053 (0.953, 1.164)	0.309	1.049 (0.956, 1.151)	0.309
fPC3	0.938 (0.857, 1.028)	0.172	0.940 (0.852, 1.038)	0.220	0.932 (0.851, 1.022)	0.135
fPC4	1.016 (0.928, 1.113)	0.726	1.030 (0.933, 1.137)	0.554	1.010 (0.921, 1.107)	0.837
MESOR	**0.654 (0.574, 0.744)**	**1.39×10^{-10}**	**0.652 (0.580, 0.732)**	**5.87×10^{-13}**	**0.647 (0.570, 0.736)**	**2.43×10^{-11}**
Amplitude	**0.623 (0.553, 0.702)**	**7.53×10^{-15}**	**0.621 (0.547, 0.705)**	**1.84×10^{-13}**	**0.666 (0.595, 0.747)**	**2.49×10^{-12}**
Acrophase	**1.150 (1.036, 1.277)**	**0.009**	**1.156 (1.045, 1.278)**	**0.005**	**1.169 (1.051, 1.301)**	**0.004**
RA	**0.764 (0.682, 0.855)**	**3.05×10^{-6}**	**0.738 (0.659, 0.825)**	**1.15×10^{-7}**	**0.725 (0.649, 0.811)**	**1.54×10^{-8}**
M10	**0.612 (0.536, 0.698)**	**3.13×10^{-13}**	**0.618 (0.545, 0.702)**	**9.16×10^{-14}**	**0.636 (0.565, 0.716)**	**6.89×10^{-14}**
L5	1.055 (0.945, 1.178)	0.343	1.055 (0.943, 1.181)	0.346	1.072 (0.960, 1.198)	0.216
M10 start	**1.133 (1.004, 1.278)**	**0.043**	1.112 (0.993, 1.244)	0.065	1.116 (0.996, 1.250)	0.059
L5 start	1.052 (0.943, 1.173)	0.363	1.067 (0.948, 1.201)	0.283	1.065 (0.949, 1.195)	0.285
IS	**0.895 (0.809, 0.991)**	**0.033**	**0.894 (0.808, 0.989)**	**0.030**	**0.891 (0.800, 0.992)**	**0.036**
IV	0.891 (0.792, 1.002)	0.054	0.905 (0.811, 1.010)	0.073	**0.891 (0.800, 0.992)**	**0.035**

Odds ratios (ORs) and 95% confidence intervals (CIs) were estimated using generalized linear mixed-effects regression models with participant-level random intercepts. P values are from two-sided Wald tests of the regression coefficients. Model 1 was adjusted for age, sex, race, education, income, employment, lifetime smoking, current alcohol consumption, and comorbidities (cancer, CVD, type 2 diabetes). Model 2 was additionally adjusted for healthcare utilization and access (no recent healthcare contact, limited healthcare access, logistic barrier, and financial barrier). Model 3 was additionally adjusted for the length of follow-up. Significant associations ($p<0.05$) are shown in bold.

Supplementary Table 17. Associations between fPCA- and PLS-derived components and accelerated biological aging at baseline.

fPC components	OR (95% CI)	PLS components	OR (95% CI)
fPC1	0.810 (0.745, 0.878)	PLS1	0.985 (0.980, 0.990)
fPC2	1.044 (0.965, 1.130)	PLS2	1.037 (1.024, 1.051)
fPC3	0.956 (0.884, 1.035)	PLS3	0.945 (0.929, 0.961)
fPC4	1.003 (0.927, 1.086)	PLS4	1.161 (1.130, 1.193)

Odds ratios (ORs) and 95% confidence intervals (CIs) were estimated using multivariable logistic regression models for accelerated biological aging. Functional principal component analysis (fPCA) components and partial least squares (PLS) components were modeled separately using the same covariate adjustment set. Models were adjusted for age, sex, race, education, income, employment, lifetime smoking, current alcohol consumption, and comorbidities (cancer, cardiovascular disease, and type 2 diabetes). Partial least squares (PLS) components were selected based on 10-fold cross-validated receiver operating characteristic area under the curve (ROC-AUC).

Supplementary Table 18. Codes used for identifying lab biomarkers in the *All of Us* Research Program.

Biomarker (unit)	Concept ID	Concept Code
Albumin (g/dL)	'1751-7','61151-7', '61152-5', '2862-1'	3024561, 40763912, 40763913, 3028286
Alkaline phosphatase (U/L)	'6768-6'	3035995
Creatinine (umol/L)	'2160-0'	3016723
C-reactive protein (mg/dL)	'1988-5','30522-7'	3020460, 3010156
Glucose (mmol/L)	'2345-7', '6777-7', '1558-6'	3004501, 3037110
Mean cell volume (fL)	'787-2', '30428-7'	3023599, 3024731
Red cell distribution width (%)	'788-0', '21000-5', '30385-9'	3019897, 3015182, 3002385
White blood cell count (10^9 cells/L)	‘6690-2’	3000905
Lymphocyte percentage (%)	'736-9','26478-8', '737-7', '71673-8', '731-0'	3037511, 3002030, 3038058, 43055366, 3004327

Supplementary Table 19. International Classification of Diseases (ICD) codes used for identifying comorbidities in the *All of Us* Research Program.

Disease Type	ICD-9	ICD-10
CVD	390-459	I00-I99
Type 2 Diabetes	250	E11
Cancer	140-239	C00-C97

Supplementary Table 20. ICD-9 and ICD-10 coding algorithms for Charlson Comorbidity Index (CCI) score.

Condition	ICD-9 Codes	ICD-10 Codes	Weight
Acute myocardial infarction	410, 412	I21.x, I22.x, I25.2	1
Congestive heart failure	398.91, 402.01, 402.11, 402.91, 404.01, 404.03, 404.11, 404.13, 404.91, 404.93, 425.4–425.9, 428	I09.9, I11.0, I13.0, I13.2, I25.2, I42.0, I42.5–I42.9, I43.x, I50.x, P29.0	1
Peripheral vascular disease	093.0, 437.3, 443.1–443.9, 447.1, 557.1, 557.9, V43.4, 440, 441	I70.x, I71.x, I73.1, I73.8, I73.9, I77.1, I79.0, I79.2, K55.1, K55.8, K55.9, Z95.8, Z95.9	1
Cerebrovascular disease	430–438, 362.34	G45.x, G46.x, H34.0, I60.x–I69.x	1
Dementia	290, 294.1, 331.2	F00.x–F03.x, F05.1, G30.x, G31.1	1
Chronic pulmonary disease	490–496, 500–505, 506.4, 508.1, 508.8	I27.8, I27.9, J40.x–J47.x, J60.x–J67.x, J68.4, J70.1, J70.3	1
Rheumatic disease	446.5, 710.0–710.4, 714.0–714.2, 714.8, 725	M05.x, M06.x, M31.5, M32.x–M34.x, M35.1, M35.3, M36.0	1
Peptic ulcer disease	531–534	K25.x–K28.x	1
Mild liver disease	070.2–070.5, 070.6, 070.9, 570, 571, 573.3–573.9, V42.7	B18.x, K70.0–K70.3, K70.9, K71.3–K71.5, K71.7, K73.x, K74.x, K76.0, K76.2–K76.4, K76.8, K76.9, Z94.4	1
Moderate/severe liver disease	456.0–456.2, 572.2–572.4, 572.8	I85.0, I85.9, I86.4, I98.2, K70.4, K71.1, K72.1, K72.9, K76.5–K76.7	3
Diabetes without complications	250.0–250.3, 250.8–250.9	E10.0–E10.1, E10.6, E10.8–E10.9, E11.0–E11.1, E11.6, E11.8–E11.9, E12.0–E12.1, E12.6, E12.8–E12.9, E13.0–E13.1, E13.6, E13.8–E13.9, E14.0–E14.1, E14.6, E14.8–E14.9	1
Diabetes with complications	250.4–250.7	E10.2–E10.5, E10.7, E11.2–E11.5, E11.7, E12.2–E12.5, E12.7, E13.2–E13.5, E13.7, E14.2–E14.5, E14.7	2
Hemiplegia or paraplegia	334.1, 342–344	G04.1, G11.4, G80.1–G80.2, G81.x, G82.x, G83.0–G83.4, G83.9	2
Renal disease	403–404, 582–588, V42.0, V45.1, V56	I12.0, I13.1, N03.2–N03.7, N05.2–N05.7, N18.x, N19.x, N25.0, Z49.0–Z49.2, Z94.0, Z99.2	2
Cancer (non-metastatic)	140–195, 200–208	C00.x–C26.x, C30.x–C34.x, C37.x–C41.x, C43.x, C45.x–C58.x, C60.x–C76.x, C81.x–C85.x, C88.x, C90.x–C97.x	2
Metastatic cancer	196–199	C77.x–C80.x	6
AIDS/HIV	042–044	B20.x–B22.x, B24.x	6

Supplementary Table 21. Medication groups defined using RxNorm ancestor concepts.

Medication Group	RxNorm Ancestor Concept ID	ATC Class	Description
Lipid-lowering agents (statins)	21601855	C10AA	HMG-CoA reductase inhibitors (statins)
Antihypertensive medications	21600381, 21601461, 21601560, 21601664, 21601744, 21601782	C02, C03, C07, C08, C09	Antihypertensive drug classes including diuretics, beta-blockers, calcium channel blockers, and renin–angiotensin system agents
Antidiabetic medications	21600712, 21600713	A10A, A10B	Insulins and blood glucose–lowering drugs (non-insulin)
CNS-active medications	21604652, 21604643, 21604642	N06A, N05C, N05B	Antidepressants, hypnotics/sedatives, and anxiolytics

Supplementary Table 22. Missing rate of biomarkers.

Features	Missing Rate (%)
Albumin	0.7
Glucose	0.8
Creatinine	2.8
Red blood cell distribution width	3.1
Mean cell volume	3.6
Lymphocyte percent	10.7
White blood cell count	12.5
Alkaline phosphatase	27.1
C-reactive protein	59.6
