# Supplementary material for: Longitudinal digital phenotyping of activity rhythms and biological aging using commercial wearables
Source: Nat Commun. 2026 Aug 22;17:10155. doi: 10.1038/s41467-026-76147-6 (PMC13601627; doi:10.1038/s41467-026-76147-6)
Supplement: Supplementary file 2 [file 41467_2026_76147_MOESM2_ESM.pdf]

nature portfolio

Corresponding author(s): Jinjoo Shim

Last updated by author(s): July 4, 2026

Reporting Summary

Nature Portfolio wishes to improve the reproducibility of the work that we publish. This form provides structure for consistency and transparency in reporting. For further information on Nature Portfolio policies, see our Editorial Policies and the Editorial Policy Checklist.

Statistics

For all statistical analyses, confirm that the following items are present in the figure legend, table legend, main text, or Methods section.

n/a	Confirmed	
☐	☒	The exact sample size (n) for each experimental group/condition, given as a discrete number and unit of measurement
☐	☒	A statement on whether measurements were taken from distinct samples or whether the same sample was measured repeatedly
☐	☒	The statistical test(s) used AND whether they are one- or two-sided<br>*Only common tests should be described solely by name; describe more complex techniques in the Methods section.*
☐	☒	A description of all covariates tested
☐	☒	A description of any assumptions or corrections, such as tests of normality and adjustment for multiple comparisons
☐	☒	A full description of the statistical parameters including central tendency (e.g. means) or other basic estimates (e.g. regression coefficient) AND variation (e.g. standard deviation) or associated estimates of uncertainty (e.g. confidence intervals)
☐	☒	For null hypothesis testing, the test statistic (e.g. F, t, r) with confidence intervals, effect sizes, degrees of freedom and P value noted<br>*Give P values as exact values whenever suitable.*
☒	☐	For Bayesian analysis, information on the choice of priors and Markov chain Monte Carlo settings
☒	☐	For hierarchical and complex designs, identification of the appropriate level for tests and full reporting of outcomes
☐	☒	Estimates of effect sizes (e.g. Cohen's d, Pearson's r), indicating how they were calculated

Our web collection on statistics for biologists contains articles on many of the points above.

Software and code

Policy information about availability of computer code

Data collection	Participants aged 18 years and older were enrolled in the All of Us Research Program after providing informed consent at participating clinics and regional medical centers within the program network.
Data analysis	Due to data privacy protections, All of Us Research Program data can only be accessed and analyzed through a secure cloud-based platform, the Researcher Workbench. All data processing and statistical analyses were conducted using Python (version 3.12; pandas 2.3.3; numpy 2.4.3; scipy 1.17.1; statsmodels 0.14.6; scikit-learn 1.8.0; scikit-fda 0.10.1; matplotlib 3.10.8; CosinorPy 3.1; pymer4 0.8.2; seaborn 0.13.2; pingouin 0.6.1) within the AoU Researcher Workbench, with parallel processing implemented across four compute nodes when applicable.

For manuscripts utilizing custom algorithms or software that are central to the research but not yet described in published literature, software must be made available to editors and reviewers. We strongly encourage code deposition in a community repository (e.g. GitHub). See the Nature Portfolio guidelines for submitting code & software for further information.

Data

Policy information about availability of data

All manuscripts must include a data availability statement. This statement should provide the following information, where applicable:

- Accession codes, unique identifiers, or web links for publicly available datasets
- A description of any restrictions on data availability
- For clinical datasets or third party data, please ensure that the statement adheres to our policy

Due to data use agreements and participant privacy protections, these data are not publicly available. Data supporting the results in this study are available for approved researchers following registration, completion of ethics training, and attestation of a data use agreement through the All of Us Researcher Workbench platform.

Research involving human participants, their data, or biological material

Policy information about studies with human participants or human data. See also policy information about sex, gender (identity/presentation), and sexual orientation and race, ethnicity and racism.

Reporting on sex and gender	Because sex-related differences in rest-activity rhythms and biological aging were expected, sex assigned at birth was considered a priori, included as a covariate in adjusted models, and used for sex-stratified analyses and sex-by-metric interaction tests. Sex assigned at birth was determined from the All of Us Research Program self-reported "sex assigned at birth" variable and categorized as male or female for analysis. Self-reported gender identity was also collected but was not used as an analytic variable in this study.
Reporting on race, ethnicity, or other socially relevant groupings	Race was categorized as white participants, black participants, and other race categories combined from Asian, Middle Eastern or North African, more than one race, and not indicated. These categories were combined to support model convergence.
Population characteristics	Table 1 describes the baseline characteristics of the study cohort (N=2,222), stratified by biological aging status. Of the participants, 1,021 (45.9%) were classified as fast agers and 1,201 (54.1%) as slow agers at baseline. Chronological age did not differ significantly between the groups (61.09 years, SD 13.82 vs. 60.23 years, SD 14.17; two-sample t-test of Δ≠0: p=0.147), whereas biological age was significantly higher in fast agers (66.43 years, SD 14.57) compared with slow agers (54.34 years, SD 14.48, two-sample t-test of Δ≠0: p<0.001). The mean length of follow-up was 6.84 years (SD 2.2).<br>Compared to slow agers, the fast agers had a greater proportion of males (37.2% vs. 26.1%, p<0.001) and a higher mean body mass index (BMI: 31.76 kg/m² vs. 28.21 kg/m²; p<0.001). Socioeconomic indicators showed consistent differences as low income was more prevalent among fast agers (21.9% vs. 14.2%; p<0.001), as was unemployment (8.6% vs. 5.9%; p=0.011). Fast agers also had a higher prevalence of lifetime smoking (38.6% vs. 32.8%; p=0.048). In line with behavioral differences, fast agers showed shorter average sleep duration (6.32 hours vs. 6.46 hours; p=0.048) and lower wearable-derived daily step counts (7,215 steps vs. 8,141 steps; p<0.001). Education, current alcohol consumption, and comorbidities of cancer, cardiovascular disease (CVD), and diabetes were comparable between groups. However, comorbidity burden, as measured by the Charlson Comorbidity Index (CCI), differed significantly between groups (p<0.001). Individuals with no comorbidity (CCI=0) were more prevalent among slow agers (43.8% vs. 30.9%), whereas a high comorbidity burden (CCI≥3) was more common among fast agers (32.4% vs. 20.3%), indicating that differences were primarily driven by the extremes of comorbidity burden. Similarly, medication use differed significantly by biological aging status (all p<0.001). Fast agers had a higher prevalence of antihypertensive (45.4% vs. 30.0%), statin (29.7% vs. 18.9%), antidiabetic (18.5% vs. 5.2%), and CNS-active medication use (16.7% vs. 9.8%) compared to slow agers. These patterns suggest a higher treatment burden and greater cardiometabolic and neurocognitive morbidity among individuals with accelerated biological aging.<br>PhenoAge was strongly correlated with chronological age (Pearson correlation coefficient, r=0.87, p<0.001) in the full sample, with consistent associations observed in males (r=0.85, p<0.001) and females (r=0.88, p<0.001) (Supplementary Fig. 1). The distribution of biological age acceleration (ΔAge) approximated normality and was centered near zero. Sex-specific distributions of ΔAge were comparable in terms of central tendency and dispersion.<br>Baseline rest-activity profiles by biological aging status (Supplementary Fig. 2) further highlighted rhythmicity distinctions. Across age strata, fast agers exhibited consistently lower peak activity levels and attenuated daytime intensity compared to slow agers. They also showed higher nocturnal activity between 00:00-06:00, whereas slow agers maintained a more consolidated rest period, consistent with a more robust rest-activity rhythm.
Recruitment	Participants were recruited nationwide. Recruitment strategies were designed to promote inclusion of historically underrepresented populations in biomedical research.
Ethics oversight	Participants aged 18 years or older were enrolled after providing written informed consent at clinics and regional medical centers participating in the AoURP network, including consent for EHR data sharing. AoURP protocol and materials have been approved by the All of Us Institutional Review Board. For this study, we used Registered Tier and Controlled Tier data available through the All of Us Researcher Workbench v8. Analyses were conducted within the secure Researcher Workbench environment, and all reported results comply with the All of Us Data and Statistics Dissemination Policy, which prohibits disclosure of group counts fewer than 20.<br>From this cohort, we identified 34,217 participants who had contributed Fitbit data through the Bring Your Own Device (BYOD) program, additionally authorized access to their Fitbit data through the AoURP Participant Portal, and provided valid physical measurements.

Note that full information on the approval of the study protocol must also be provided in the manuscript.

Field-specific reporting

Please select the one below that is the best fit for your research. If you are not sure, read the appropriate sections before making your selection.

☐ Life sciences ☒ Behavioural & social sciences ☐ Ecological, evolutionary & environmental sciences

For a reference copy of the document with all sections, see nature.com/documents/nr-reporting-summary-flat.pdf

Behavioural & social sciences study design

All studies must disclose on these points even when the disclosure is negative.

Study description	This study is a quantitative longitudinal observational cohort study using All of Us wearable, EHR, and survey data.
Research sample	For this study, we used registered and controlled tier data available through the All of Us (AoU) Researcher Workbench (version 8). The study sample comprised 2,222 participants with available wearable-derived activity data and linked clinical and survey information. The All of Us Research Program is a large, diverse, longitudinal cohort designed to reflect the demographic diversity of the United States, with intentional recruitment of populations historically underrepresented in biomedical research. While not a nationally representative sample, it provides broad coverage across demographic, socioeconomic, and health-related characteristics.<br><br>The study sample was restricted to participants with sufficient wearable data quality and complete covariate information to enable robust estimation of circadian rhythm metrics and their associations with biological aging. This selection was necessary to ensure data completeness, consistency of exposure measurement, and validity of longitudinal analyses.
Sampling strategy	Participants were not selected through random or probability-based sampling. Instead, the analytic sample represents a subset of the All of Us Research Program participants who voluntarily contributed wearable data through the Bring Your Own Device (BYOD) program and met predefined inclusion criteria. Therefore, the study sample can be considered a convenience sample derived from an existing cohort.<br><br>No formal a priori sample size calculation was performed. The sample size was determined by the number of participants with sufficient wearable data quality, complete covariate information, and longitudinal biological age measurements. From an initial pool of 34,217 participants with available Fitbit data, we applied predefined inclusion and exclusion criteria to ensure valid and continuous data capture, resulting in a final analytic cohort of 2,222 participants contributing 8,447 person-years of follow-up, restricted to participants with sufficient Fitbit and repeated biological-age data; not nationally representative, convenience subset of an existing cohort.<br><br>The resulting sample size is sufficient to support the statistical analyses performed, including multivariable regression and longitudinal mixed-effects models, given the number of events, repeated measurements, and covariates included. The use of a well-characterized cohort with repeated measures further enhances statistical power and robustness of inference despite the absence of formal sample size calculation.
Data collection	Data were collected through the All of Us Research Program using a combination of wearable device data, electronic health records (EHR), and survey responses. Wearable-derived rest-activity rhythm data were obtained from Fitbit devices contributed by participants through the Bring Your Own Device (BYOD) program. Participants voluntarily linked their personal Fitbit accounts to the All of Us Participant Portal, allowing access to both prospective and historical data. Wearable data were passively recorded under free-living conditions without any intervention or supervision by the research team. No researchers were present during data collection, and no experimental conditions were imposed. All data were de-identified by the All of Us Research Program prior to researcher access, with date-time fields shifted to protect participant privacy. Because this study used existing, observational data collected independently of the current research question, researchers were effectively blinded to participant identity and did not influence data generation. There was no assignment to experimental conditions or intervention, and thus no requirement for blinding to intervention status. Covariates include age at the time of Fitbit monitoring, sex assigned at birth, race, educational attainment, employment status, annual household income, body mass index, smoking history, alcohol consumption, and major comorbidities including cardiovascular disease, type 2 diabetes, and cancer.
Timing	The analytic dataset includes participant data from 2011 to 2023 (cut-off date: October 1, 2023). Exact calendar dates of data collection are not reported due to the All of Us Research Program's de-identification procedures, which include random date shifting to protect participant privacy. For each participant, baseline was defined as the earliest date of a valid wearable (Fitbit) data recording period. Follow-up extended from baseline to the last available date of valid wearable monitoring. Only participants with complete step count data for a full 12-month period within a given calendar year were included, and analyses were conducted at the participant-year level.
Data exclusions	Data exclusions were applied based on pre-specified criteria. From 34,217 participants with available Fitbit data, individuals with insufficient wearable data quality or incomplete follow-up information were excluded, resulting in a final analytic cohort of 2,222 participants. Exclusion criteria were defined a priori to ensure data quality and validity of longitudinal analyses.
Non-participation	No participants were prospectively enrolled, and therefore no participants dropped out or declined participation in the context of this study. The analysis was conducted using de-identified data from the All of Us Research Program, and the final analytic sample was defined based on pre-specified inclusion criteria.
Randomization	Participants were not allocated to experimental groups.

Reporting for specific materials, systems and methods

We require information from authors about some types of materials, experimental systems and methods used in many studies. Here, indicate whether each material, system or method listed is relevant to your study. If you are not sure if a list item applies to your research, read the appropriate section before selecting a response.

Materials & experimental systems

n/a	Involved in the study
☒	☐ Antibodies
☒	☐ Eukaryotic cell lines
☒	☐ Palaeontology and archaeology
☒	☐ Animals and other organisms
☒	☐ Clinical data
☒	☐ Dual use research of concern
☒	☐ Plants

Methods

n/a	Involved in the study
☒	☐ ChIP-seq
☒	☐ Flow cytometry
☒	☐ MRI-based neuroimaging

Plants

Seed stocks	*Report on the source of all seed stocks or other plant material used. If applicable, state the seed stock centre and catalogue number. If plant specimens were collected from the field, describe the collection location, date and sampling procedures.*
Novel plant genotypes	*Describe the methods by which all novel plant genotypes were produced. This includes those generated by transgenic approaches, gene editing, chemical/radiation-based mutagenesis and hybridization. For transgenic lines, describe the transformation method, the number of independent lines analyzed and the generation upon which experiments were performed. For gene-edited lines, describe the editor used, the endogenous sequence targeted for editing, the targeting guide RNA sequence (if applicable) and how the editor was applied.*
Authentication	*Describe any authentication procedures for each seed stock used or novel genotype generated. Describe any experiments used to assess the effect of a mutation and, where applicable, how potential secondary effects (e.g. second site T-DNA insertions, mosiacism, off-target gene editing) were examined.*
