# Supplementary material for: Longitudinal digital phenotyping of activity rhythms and biological aging using commercial wearables
Source: Nat Commun. 2026 Aug 22;17:10155. doi: 10.1038/s41467-026-76147-6 (PMC13601627; doi:10.1038/s41467-026-76147-6)
Supplement: Supplementary file 3 [file 41467_2026_76147_MOESM3_ESM.pdf]

nature portfolio

Peer Review File

Longitudinal Digital Phenotyping of Activity Rhythms and Biological Aging Using Commercial Wearables

Corresponding Author: Dr Jinjoo Shim

This file contains all reviewer reports in order by version, followed by all author rebuttals in order by version.

Version 0:

Reviewer comments:

Reviewer #1

(Remarks to the Author)
This manuscript reports a longitudinal analysis of circadian rest-activity rhythm CRAR patterns derived from Fitbit data in over 2,200 participants in the All of Us Research Program, linked to annual biological age estimates using a method called PhenoAge. The authors find that higher rhythm intensity (e.g., MESOR, amplitude, fPC1) and regularity are associated with slower biological aging, whereas delayed activity timing (e.g., acrophase, M10 start time) predicts accelerated aging, especially in females. The paper presents a technically well-executed analysis leveraging both parametric and functional data analysis (fPCA) and makes a credible case for digital phenotyping of aging-related physiology. Strengths include a large longitudinal cohort with consumer device data, advanced circadian phenotyping, and sex-stratified and time-varying models. Limitations include the application of the PhenoAge construct using EHR data, and perhaps underappreciated potential for residual confounding.

Major Comments:
1. Limitations of PhenoAge
The PhenoAge metric is a central outcome but concerns about its use here remain. It is derived from EHR lab values, which may be sparsely measured and influenced by clinical care patterns. The authors mention imputation, including over 50% of CRP values were missing and substituted with a global mean. Imputation rates of other measures are not reported (perhaps I missed them). This likely attenuates associations and biases ΔAge estimates. The PhenoAgo "validation" study focused on an epigenetic application, not use of the clinical data alone. PhenoAge, as used here, is simply a regression analysis linking lab values to clinical outcomes. The results of which are somewhat predictable because the nine markers used are known to be associated with chronic diseases (e.g. glucose, RDW, CRP). Despite "validation", I am a bit dubious that these markers represent a biological clock per se. It would help the reader if these limitations were more transparent and acknowledged.

2. Causal Interpretation and Residual Confounding
Although the authors acknowledge observational limitations, the tone at times verges on causal inference (e.g., "changes in rhythms shaped aging trajectories"). Moreover, key time-varying covariates such as medications, mental health, acute illness, or frailty markers are not adjusted for or considered. These could confound both activity rhythms and biomarker profiles. Stronger language cautioning against causal interpretation is warranted, and inverse probability weighting or marginal structural models should be considered.

3. Biological Interpretation of fPCs
The fPCA approach is elegant but opaque. While fPC1 aligns well with rhythm amplitude, fPC3 and fPC4 (e.g., "biphasic instability") are harder to interpret physiologically. The authors should clarify whether these patterns correspond to known biological phenomena (e.g., chronotype, sleep fragmentation, nap patterns) or may be device artifacts. Without biological anchoring (e.g., hormonal rhythms, inflammatory markers), the link between latent components and aging trajectories is speculative. Perhaps incorporation of the Fitbit-derived sleep data would be complementary (albeit imperfect).

Minor Comments:
• Abstract: Suggest clarifying that the "PhenoAge" application here is not the same as the reference provided from Levine et al, which included epigenetic changes in the model. Otherwise, the reader is left concluding that the application presented

here has been studied in this way.
• Figures: Figure 2 (fPC plots) could benefit from clearer labeling of axes and units (e.g., what does a "score of 1" mean in activity terms?).
• Table 1: Consider including comorbidity burden (e.g., Charlson index) or medication use to help contextualize aging status groups.

Reviewer #2

(Remarks to the Author)
This is a very interesting manuscript that aims to examine the relationship between longitudinal wearable-based activity rhythms and biological age acceleration by leveraging data from the All of Us Research Program. It is well designed, and the data analyses are comprehensive. The results are solid and consistent with previous findings. However, there are still some issues that need to be clarified.
(1) It is unclear when the baseline data were collected and how many years the participants were followed up.
(2) The analyses of Year 1, Year 3, and Year 5 are confusing. Were the follow-up visits conducted at Year 3 and Year 5? Were all participants included or only a sub-population? The results from Figure 3 are not matched to those from Table 2. For example, it seems that Acrophase was significantly different between slow and fast aging groups at Year 3 and Year 5 (Figure 3), but this association was not significant during the follow-up shown in Table 2.
(3) Figure 4 is hard to understand. In all situations for all parameters, the ORs for fasting age were above 1, suggesting a significant increased risk, which is not consistent with the primary results.
(4) Some results are not consistent between Table 2 and Table 3. For example, the association between IS and BioAge seems significant in cross-sectional data (significant at the baseline and borderline at the follow-up), while it is completely gone in the mixed analysis. This may need to be further clarified.
(5) Figure 5 is very interesting. Are the associations between ΔCRMetrics and ΔBioAge significant? If so, in my opinion it may provide more novel findings than those revealed from the mixed analysis.
(6) Since these are longitudinal data, it would be interesting to know whether the baseline CRMetrics can predict BioAge at the follow-up.
(7) The analysis of interaction with follow-up years is confusing (Suppl Table 3). What is the purpose of this analysis? How to interpret the results? There is significant interaction between fPC1 and year (OR=1.218) on BioAge. What does this OR mean?

Reviewer #3

(Remarks to the Author)
Thank you for the interesting submission! Below I will try to concisely articulate the central claims of the work, followed by comments / questions. Of course please correct any errors in my interpretation.

Claims made

* The central claim in this work is that rhythmic features of "activity" (here step count measurements obtained via a wearable) are associated with pace of aging. Crucially, "aging" is assessed using a PhenoAge constructed through EHR records, with some amount of imputation. Rhythmic features examined include standard features and nonparametric fPCA latent variables.
* Rhythm intensity is protective — here higher amplitude of MESOR, amplitude, M10, and fPC1 are consistently associated with reduced likelihood of accelerated PhenoAge. This finding holds across cross-sectional and longitudinal analyses.
* Timing is a Risk Factor: Delayed circadian timing, particularly a later acrophase (timing of peak activity) and later onset of the least active period (L5 start time), is associated with an increased likelihood of accelerated PhenoAge. Greater day-to-day regularity is associated with slower biological aging, however may be sex-specific.
* Some associations appear to be sex-dependent. The effects of timing and regularity are claimed to be more pronounced in females. In contrast, males with accelerated aging are uniquely characterized by a "biphasic instability" phenotype (early-morning and late-evening activity peaks, captured by fPC4).

Comments & Questions

* "... as a digital biomarker of aging risk" (line 86) — "aging risk" strikes me as an odd construction. One might be more precise and say "digital biomarker of accelerated aging risk," or better yet, "digital biomarker of accelerated PhenoAge". For clarity, it might be appropriate to distinguish between "accelerated PhenoAge," the measured variable, and "accelerated aging," the biological process it is intended to measure.
* Within subject change in PhenoAge is a major end point in this study. What is the evidence in the literature longitudinal PhenoAge is a reliable surrogate of accelerated "biological aging"? It would bolster the motivation to point to evidence that longitudinal, accelerated PhenoAge independently correlates with increased disease or mortality risk, above just cross sectional PhenoAge measurements.
* Regarding fast vs slow agers, "... cardiovascular disease, and diabetes did not differ significantly between the two groups" — is that odd? Would you not expect faster agers to also have more diabetes and CVD diagnoses?
* "slow agers ... exhibited higher MESOR" (line 124) — first mention of MESOR, would appreciate a definition/description of this statistic.

* “latent nonlinear variation in 24-h profiles” (line 134) — on initial read it was unclear that these refer to a representation of the daily step-count data, could be clarifying to be explicit that these are 24-h step-count profiles.
* The fPCA analysis is super interesting, and the fPCs seem to clearly identify general behavioral patterns wrt activity. These bases are, however, not informed by aging or pace of aging — would an analyses like partial least squares (or a functional variant) yield bases that reveal variability specific to accelerated aging?
* “Sex-stratified analyses further refined these insights ...” (line 282) — How confident can we be that the sex-specific differences reveal genuine variability between the sexes as opposed to some statistical artifact?
* In fact, elsewhere in the manuscript (line 128, line 501) sex-stratified analyses are viewed as a robustness or sensitivity check — finding similar effects bolsters the finding of the full-data model. Here, seeing timing and regularity related associations appearing stronger in women, while weaker in men. What sensitivity checks or robustness would give us faith in this differential result?
* “circadian rhythmicity exerts a lasting influence on biological aging trajectories” (line 347-348) — This strikes me as a pretty squarely causal claim. I would temper this kind of claim, as the evidence from the analysis certainly doesn’t definitely show that step-count circadian rhythms cause accelerated or decelerated aging (based on PhenoAge).
* “When up to two biomarkers were missing, values were imputed using the cohort mean” (line 401-402) — Why not impute missing PhenoAge variables with more sophisticated approach like MICE? Is there a reason to think that these values are missing not at random, or at least if missing at random other observables can explain away the missingness patterns? What effect would this have on the conclusions of the analyses?
* Further, what kind of selection effects could influence your analysis by relying on the EHR for reconstructing the outcome? What kind of selection effects could result from relying on longitudinal labs that go into the PhenoAge calculation?
* Would this imputation strategy result — more or less constant imputation for missing values — push longitudinal delta PhenoAges toward zero?
* “Participants with delta Age > 0 were classified as fast aging, and those with delta Age <= 0 as slow aging” (line 407-408) — Why dichotomize delta Age in your analyses? Could intrinsic variability in the lab create noise around delta Age = 0? Would grouping variables in to tertiles or quantiles demonstrate a stronger effect in the extremes? Why not analyze delta Age as it relates to activity rhythmicity and amplitude directly?
* Lines 294-296 (and more generally) pluralizing female → females, male → males
* “The cosinor model has been applied to analyze circadian patterns ...” (lines 417-420) — the reader (and this reviewer) would benefit from a bit more discussion about what these features (the CRAR and the MESOR) really capture. The other statistics — M10/L5, RA, IS,and IV — are discussed at a high level with good intuition conveyed (e.g., “A higher IS value indicates greater day-to-day regularity”). What does a higher MESOR really mean in this sense?
* “Then we projected these trajectories onto a nine-term Fourier basis, ...” (line 452) — why do both a 9-term Fourier basis projection followed by only retaining the first four functional PCs? Is it possible that the initial Fourier smoothing removes relevant or predictive variability in the signal? What kinds of artifacts does this first Fourier stage jettison? Why retain only four PCs?
* Fig 1 — It’s difficult to suss out statistical artifacts from genuine cohort differences in these plots. Could they benefit by visualizing some notion of uncertainty, or perhaps the difference between two averages (with error bars)?
* Fig 2(a) — what is the range depicted by the multiple curves? The extremes of the data distribution? Quartiles?
* In the linear models used and presented, could there be any interactions between features? Could timing factors have different effect sizes given a fixed amplitude level? Similarly, could effects differ by chronological age?
* It seems like the most durable/robust findings of these analyses is that intensity of activity is associated with slower PhenoAge. How much of this effect could be explained by the fact that “exercise is protective” in general?
* A key differentiating factor of this study is the deep dive into rhythmic factors (like timing, relative amplitude, acrophase, stability). However those seem to be less reliable or variable from analysis to analysis.
* Furthermore, could a “strong circadian rhythm” (e.g., measured by MESOR, if i’m not mistaken) also pick up on regularity in sleep patterns? To what extent are we seeing evidence of the benefits of exercise and a predictable sleep schedule?

I look forward to your response.

Version 1:

Reviewer comments:

Reviewer #1

(Remarks to the Author)
The authors have done an excellent job of addressing my concerns in a remarkably thorough response. I appreciate the effort to temper causal language and provide more transparency about the study limitations. I have no further comments.

Reviewer #2

(Remarks to the Author)
The authors have addressed my concerns.
Minor edit error: The Table 3 is missing OR values.

Reviewer #3

(Remarks to the Author)
Thank you to the authors for their thorough and substantive responses to the reviews. The revisions address many of my concerns, and the additional (many) sensitivity analyses assuage concerns about robustness and generalizability of the effect; I genuinely appreciate your taking the time to conduct these analyses and curate this evidence. A few points remain where I'd appreciate further clarification or refinement.

PhenoAge as a longitudinal endpoint (Response 3):

I appreciate the additional context on PhenoAge and the references provided. My remaining concern is specifically about ΔPhenoAge as a longitudinal endpoint. The cited Kuo et al. study, as I read it, developed a new phenotypic aging metric explicitly designed to be longitudinally meaningful — which, if my interpretation is correct, doesn't directly speak to the longitudinal utility of the original PhenoAge construct (the specific nine-biomarker combination used here). I don't doubt that longitudinal changes in PhenoAge can be predictive of health outcomes, but it would strengthen the manuscript to cite evidence supporting that claim directly, rather than via Kuo. As written, the citation risks being read as stronger support than it actually provides.

To be clear: I'm not arguing against PhenoAge as an endpoint — it's a reasonable choice. I would just like the evidence base for its longitudinal use, and its limitations in that setting, to be presented transparently. No endpoint is perfect, and acknowledging that explicitly would strengthen rather than weaken the paper.

Further, in response 2, your proposed text reads “Our findings provide among the first large-scale longitudinal evidence that rest-activity rhythm shapes trajectories of biological aging...” Again I would temper such a causal claim, and say that rest-activity rhythm associates with (or even robustly associates with, as you’ve shown) trajectories of biological aging.

PLS vs. fPCA (Figure 2a):

I take the authors' point that the PLS and fPCA bases overlap substantially, which is largely tautological given that they are different rotations of the same underlying variation. My original question was about interpreting the bases themselves rather than the overlap (as in Figure 2). The framing that PLS "redistributes existing fPCA-derived variation" reads as though one analysis precedes the other in a meaningful way, but I don't think that's quite right — neither basis is more fundamental, they are simply different rotations chosen under different criteria.

Sensitivity analyses:

It's heartening to see that the results are robust to both the additional sensitivity checks and alternative adjustment-variable sets. This substantially increases my confidence in the core findings.

Open Access This Peer Review File is licensed under a Creative Commons Attribution 4.0 International License, which permits use, sharing, adaptation, distribution and reproduction in any medium or format, as long as you give appropriate credit to the original author(s) and the source, provide a link to the Creative Commons license, and indicate if changes were made.

In cases where reviewers are anonymous, credit should be given to 'Anonymous Referee' and the source.
The images or other third party material in this Peer Review File are included in the article's Creative Commons license, unless indicated otherwise in a credit line to the material. If material is not included in the article's Creative Commons license and your intended use is not permitted by statutory regulation or exceeds the permitted use, you will need to obtain permission directly from the copyright holder.
To view a copy of this license, visit https://creativecommons.org/licenses/by/4.0/

We thank the reviewers for their meticulous reading of our manuscript and valuable feedback, which significantly improved the quality of our work. The reviewers' insightful comments allowed us to incorporate important revisions, resulting in a stronger manuscript. We have included all modifications made in response to the reviewers' feedback and addressed each comment individually. Reviewer comments are presented in blue, and our responses in black.

Reviewer #1

This manuscript reports a longitudinal analysis of circadian rest-activity rhythm CRAR patterns derived from Fitbit data in over 2,200 participants in the All of Us Research Program, linked to annual biological age estimates using a method called PhenoAge. The authors find that higher rhythm intensity (e.g., MESOR, amplitude, fPC1) and regularity are associated with slower biological aging, whereas delayed activity timing (e.g., acrophase, M10 start time) predicts accelerated aging, especially in females. The paper presents a technically well-executed analysis leveraging both parametric and functional data analysis (fPCA) and makes a credible case for digital phenotyping of aging-related physiology. Strengths include a large longitudinal cohort with consumer device data, advanced circadian phenotyping, and sex-stratified and time-varying models. Limitations include the application of the PhenoAge construct using EHR data, and perhaps underappreciated potential for residual confounding.

Major Comments:

1. Limitations of PhenoAge

The PhenoAge metric is a central outcome but concerns about its use here remain. It is derived from EHR lab values, which may be sparsely measured and influenced by clinical care patterns. The authors mention imputation, including over 50% of CRP values were missing and substituted with a global mean. Imputation rates of other measures are not reported (perhaps I missed them). This likely attenuates associations and biases ΔAge estimates. The PhenoAge “validation” study focused on an epigenetic application, not use of the clinical data alone. PhenoAge, as used here, is simply a regression analysis linking lab values to clinical outcomes. The results of which are somewhat predictable because the nine markers used are known to be associated with chronic diseases (e.g. glucose, RDW, CRP). Despite “validation”, I am a bit dubious that these markers represent a biological clock per se. It would help the reader if these limitations were more transparent and acknowledged.

Response 1, Reviewer 1: We appreciate the reviewer’s valuable time for reviewing our manuscripts and providing insightful feedback and suggestions. We also thank the reviewer for this thoughtful and important critique regarding the use of PhenoAge as the primary outcome. We have carefully addressed each of the concerns raised, including issues related to EHR-based measurement, missingness and imputation, construct validity, and interpretation. Our response is structured point-by-point below.

1. **Conceptual clarification of PhenoAge:** PhenoAge, as used in this study, is derived from clinical biomarkers obtained through EHR data and is therefore distinct from molecular or epigenetic “clocks.” Levine’s PhenoAge has been used to refer to two related but distinct constructs: (1) the original multi-system estimate of ‘Phenotypic Age’ (PhenoAge) derived from clinical biomarkers, and (2) the later-developed epigenetic ‘DNAm PhenoAge’[1,2]. While these constructs are conceptually related, they are based on different data modalities, with the former derived from blood-based clinical biomarkers

and the latter representing an epigenetic clock. In this study, PhenoAge refers to the clinical biomarker-based measure.

To avoid any potential ambiguity, we have revised the manuscript to clarify that our analyses are based on the clinical biomarker-based PhenoAge derived from blood chemistry measures, rather than the epigenetic DNAm PhenoAge. This clarification is consistent with prior literature that distinguishes these constructs using this definition[3–6].

Specifically, we now (1) define this distinction at first mention in the Abstract and main text, (2) introduce the term "clinical biomarker-based PhenoAge," and (3) maintain consistent terminology throughout the manuscript to avoid ambiguity. These revisions have been incorporated in the Abstract, Introduction, Methods, and Discussion sections, and we believe this clarification resolves the potential for misinterpretation. → (Abstract, p. 2; Introduction, p. 4; Methods, p. 24; Discussion, p. 13)

- (Abstract, p. 2) "Leveraging the All of Us Research Program, we integrated multi-year Fitbit activity data with annual biological age estimates <u>based on clinical biomarker-derived PhenoAge</u> from 2,222 participants (8,447 person-years)."

- (Introduction, p. 4) "By linking these longitudinal activity rhythm profiles with repeated measures of biological age, quantified <u>using the clinical biomarker-based</u> phenotypic age (PhenoAge)[1], we <u>aim to capture the dynamic progression of biological aging. PhenoAge is calculated based on a mortality prediction score derived from nine clinical biomarkers (albumin, alkaline phosphatase, creatinine, C-reactive protein (CRP), glucose, mean cell volume, red cell distribution width, white blood cell count, and lymphocyte percentage) and chronological age, reflecting the age at which the average NHANES III mortality risk matches the predicted mortality risk[1]. It has been validated across multi-ethnic cohorts to predict disease, physical functioning, cognitive performance, and mortality, and whose longitudinal changes provide prognostic information beyond baseline levels[1,3,7–10].</u>"

- (Methods, p. 24) "**Biological age estimation**
  The primary outcome was <u>clinical biomarker-based</u> phenotypic age (PhenoAge), a validated biomarker-based estimate of biological age that predicts morbidity, functional decline, and all-cause mortality[2]. <u>Beyond cross-sectional associations, longitudinal changes in phenotypic age have been shown to be associated with disease and mortality risk, supporting its use as a dynamic marker of aging trajectories[8].</u> PhenoAge has also been associated with wearable-derived rhythmicity in prior cross-sectional analyses[11,12]. It is calculated using the Gompertz proportional hazards model trained on nine clinical biomarkers, including albumin, alkaline phosphatase, creatinine, C-reactive protein (CRP), glucose, mean cell volume, red cell distribution width, white blood cell count, and lymphocyte percentage, along with chronological age[2]. <u>These biomarkers were selected using a Cox proportional hazards elastic net model for mortality prediction with ten-fold cross validation in the National Health and Nutrition Examination Survey III (NHANES III) cohort[2]. PhenoAge estimation is based on the parametrization of two Gompertz proportional hazards models: one incorporating the selected biomarkers and chronological age to estimate an individual-level mortality risk, and the other using chronological age alone as a baseline. PhenoAge is then derived by equating the predicted mortality</u>

risks from both models, reflecting the age at which an individual's mortality risk is equivalent of the refence population."

- (Discussion, p. 13) "In this large-scale, prospective cohort with multi-year wearable monitoring in free-living settings, we demonstrated that circadian rest-activity rhythms are robust determinants of biological aging trajectories, assessed using clinical biomarker-based PhenoAge."

2. **Addressing concerns that PhenoAge is derived from EHR data and may reflect healthcare utilization rather than biology:** We acknowledge that EHR-derived biomarkers may be influenced by healthcare utilization patterns, including measurement frequency and access to care. Individuals who are more engaged with the healthcare system are more likely to have repeated laboratory measurements captured in the EHR, which increases the likelihood of both (1) being included in the analytic sample and (2) having more complete estimates of longitudinal biomarkers such as PhenoAge. Conversely, individuals with limited healthcare access or lower engagement may have fewer recorded laboratory measurements, leading to underrepresentation and potential bias in both exposure and outcome ascertainment. To address this concern, we conducted several targeted analyses designed to evaluate distinct sources of potential selection bias.

(1) Comparison of included vs. excluded participants: First, we compared baseline characteristics between individuals included in the longitudinal analysis (i.e., those with wearable data and repeated laboratory measurements) and those excluded (i.e., those with wearable data but without repeated laboratory measurements). In this comparison, we further characterized healthcare access and engagement using the All of Us Healthcare Utilization and Access survey. We constructed four domains: (1) no recent healthcare contact within the past 12 months, capturing lack of recent interaction with the healthcare system; (2) limited healthcare access, defined by absence of a usual source of care or prolonged gaps in care; (3) sociological and logistical barriers, including challenges such as transportation difficulties, inability to take time off work, or caregiving responsibilities; and (4) financial barriers, including cost-related obstacles such as inability to afford copayments, deductibles, or out-of-pocket expenses.

As shown in **Supplementary Table 15**, inclusion in the analytic sample was associated with healthcare engagement. Excluded individuals were younger, had lower income, lower comorbidities, and higher prevalence of limited healthcare access and barriers, indicating systematically reduced clinical capture (all $p<0.05$).

- (Supplementary Information, p. 28)

Supplementary Table 15. Baseline characteristics of the study population by inclusion and exclusion status.

Mean (SD) or n (%)	Included (n=2222)	Excluded (n=2249)	*p*-value
Age, years	60.62 (14.01)	55.67 (15.09)	<0.001
PhenoAge, years	59.90 (15.72)	55.07 (17.08)	<0.001
Sex			0.107
Female	1522 (68.5)	1604 (71.3)	

Male	693 (31.2)	634 (28.2)	
Race			<0.001
White	1839 (82.8)	1763 (78.4)	
Black	134 (6.0)	130 (5.8)	
Other	249 (11.2)	356 (15.8)	
BMI, kg/m^2	29.84 (6.98)	30.18 (8.04)	0.139
Education			0.237
College or above	2084 (93.8)	2100 (93.4)	
12 years or GED	119 (5.4)	122 (5.4)	
Income			<0.001
High	1015 (45.7)	894 (39.8)	
Medium	669 (30.1)	683 (30.4)	
Low	395 (17.8)	522 (23.2)	
Missing	143 (6.4)	150 (6.7)	
Employment			<0.001
Employed	1260 (56.7)	1403 (62.4)	
Retired	732 (32.9)	534 (23.7)	
Unemployed	159 (7.2)	207 (9.2)	
Other	71 (3.2)	105 (4.7)	
Smoking			0.228
Yes	788 (35.5)	758 (33.7)	
No	1434 (64.5)	1496 (66.3)	
Drinking			0.033
Yes	2168 (97.6)	2169 (96.4)	
No	54 (2.4)	80 (3.6)	
Comorbidities			
Cancer	207 (9.3)	155 (6.9)	0.007
CVD	247 (11.1)	185 (8.2)	0.001
Type 2 diabetes	212 (9.5)	33 (1.5)	<0.001
Daily steps	7715.83 (3429.04)	6566.24 (3175.47)	<0.001
Sleep duration, hours	6.39 (1.67)	6.24 (1.65)	0.013
No recent healthcare contact	181 (8.1)	274 (12.2)	<0.001
Limited healthcare access	518 (23.3)	706 (31.4)	<0.001
Logistic barrier	429 (19.3)	555 (24.7)	<0.001
Finance barrier	312 (14.0)	446 (19.8)	<0.001
Length of follow-up, years	6.84 (2.2)	5.15 (2.5)	<0.001

Values are mean (SD) for continuous variables and n (%) for categorical variables. *p*-values are based on *t*-tests or chi-square tests, as appropriate.

(2) Adjusting for healthcare utilization and access: Second, to account for these observed differences, we additionally adjusted for healthcare utilization and access measures in statistical analyses, including visit frequency, usual source of care, and structural, logistical, and financial barriers to care (**Supplementary Table 16, Model 2, see next page**). Effect estimates remained consistent after this adjustment, suggesting that differential healthcare engagement does not materially explain the observed associations.

(3) **Adjusting for the length of follow-up:** Finally, we further adjusted for the length of follow-up as individuals with longer monitoring periods are more likely to have repeated laboratory measurements. Because length of follow-up may be related to the probability of observing both exposures and outcomes in EHR data, this adjustment provides an additional control for differential ascertainment. The results remained robust after this adjustment (**Supplementary 16, Model 3**).

- (Supplementary Information, p. 29)

Supplementary Table 16. Sensitivity analyses adjusting for healthcare engagement and the length of follow-up.

Features	Model 1 OR (95% CI)	*p*-value	Model 2 OR (95% CI)	*p*-value	Model 3 OR (95% CI)	*p*-value
fPC1	**0.832 (0.757, 0.915)**	**<0.001**	**0.834 (0.752, 0.925)**	**0.001**	**0.823 (0.747, 0.905)**	**<0.001**
fPC2	1.059 (0.966, 1.162)	0.219	1.053 (0.953, 1.164)	0.309	1.049 (0.956, 1.151)	0.309
fPC3	0.938 (0.857, 1.028)	0.172	0.940 (0.852, 1.038)	0.220	0.932 (0.851, 1.022)	0.135
fPC4	1.016 (0.928, 1.113)	0.726	1.030 (0.933, 1.137)	0.554	1.010 (0.921, 1.107)	0.837
MESOR	**0.654 (0.574, 0.744)**	**<0.001**	**0.652 (0.580, 0.732)**	**<0.001**	**0.647 (0.570, 0.736)**	**<0.001**
Amplitude	**0.623 (0.553, 0.702)**	**<0.001**	**0.621 (0.547, 0.705)**	**<0.001**	**0.666 (0.595, 0.747)**	**<0.001**
Acrophase	**1.150 (1.036, 1.277)**	**0.009**	**1.156 (1.045, 1.278)**	**0.005**	**1.169 (1.051, 1.301)**	**0.004**
RA	**0.764 (0.682, 0.855)**	**<0.001**	**0.738 (0.659, 0.825)**	**<0.001**	**0.725 (0.649, 0.811)**	**<0.001**
M10	**0.612 (0.536, 0.698)**	**<0.001**	**0.618 (0.545, 0.702)**	**<0.001**	**0.636 (0.565, 0.716)**	**<0.001**
L5	1.055 (0.945, 1.178)	0.343	1.055 (0.943, 1.181)	0.346	1.072 (0.960, 1.198)	0.216
M10 start	**1.133 (1.004, 1.278)**	**0.043**	1.112 (0.993, 1.244)	0.065	1.116 (0.996, 1.250)	0.059
L5 start	1.052 (0.943, 1.173)	0.363	1.067 (0.948, 1.201)	0.283	1.065 (0.949. 1.195)	0.285
IS	**0.895 (0.809, 0.991**	**0.033**	**0.894 (0.808, 0.989)**	**0.030**	**0.891 (0.800, 0.992)**	**0.036**
IV	0.891 (0.792, 1.002)	0.054	0.905 (0.811, 1.010)	0.073	**0.891 (0.800, 0.992)**	**0.035**

Odds ratios (ORs) and 95% confidence intervals (CIs) are derived from linear mixed-effects regression models. Model 1 include a random intercept and adjust for age, sex, race/ethnicity, education, income, employment, lifetime smoking, current alcohol consumption, and comorbidities (cancer, CVD, type 2 diabetes). Model 2 additionally adjust for healthcare utilization and access (no recent healthcare contact, limited healthcare access, logistic barrier, and financial barrier). Model 3 additionally adjust for the length of follow-up. Significant associations ($p<0.05$) are shown in bold.

The consistency of findings after adjustment for healthcare utilization, access to care, and the length of follow-up suggests that the observed associations are unlikely to be materially driven by selection bias. We have clarified these points in the Methods, Discussion, and Supplementary Information. → (Methods, p. 30; Results, p. 12; Discussion, p. 19; Supplementary Tables 15-16).

- (Methods, p. 30) "<u>Fourth, to assess potential selection related to healthcare engagement, we compared baseline characteristics between individuals included in the longitudinal analysis (i.e., those with wearable data and repeated laboratory measurements) and those excluded (i.e., those with wearable data but without</u>

repeated laboratory measurements). Healthcare access and utilization were further characterized using the *All of Us* Healthcare Utilization and Access survey, including domains of recent healthcare contact, access to care, logistical barriers, and financial barriers. Fifth, we additionally adjusted for healthcare utilization and the length of follow-up in statistical models to account for differential healthcare engagement and observation time."

- (Results, p. 12) "We additionally assessed potential selection bias related to healthcare access and completeness of laboratory-based outcome data. Excluded individuals were younger, had lower income, fewer comorbidities, and greater barriers to healthcare across logistical, financial, and access domains (**Supplementary Table 15).** However, further adjustment for healthcare utilization, access to care, and length of follow-up did not materially alter the observed associations (**Supplementary Table 16**). These findings indicate that the observed associations are robust to potential selection and observation biases."

- (Discussion, p. 19) "We also evaluated, in sensitivity analyses, whether selection related to healthcare engagement may influence inclusion in the analytic sample and the completeness of laboratory-based measurements used to derive PhenoAge. Individuals with higher healthcare utilization are more likely to have repeated laboratory data captured in EHRs, whereas those with limited access may be underrepresented. In our data, inclusion was associated with healthcare engagement, as excluded individuals were younger, had lower income, fewer comorbidities, and a higher prevalence of barriers to care. Adjustment for healthcare utilization, access to care, and the length of follow-up did not materially change the observed associations. Because behavioral phenotypes were derived from wearable data independently of healthcare utilization, whereas selection primarily affects the availability and completeness of laboratory-based outcomes, this form of selection may preferentially influence measurement completeness rather than the underlying associations. Accordingly, while selection related to healthcare engagement may affect sample representativeness, it is less likely to fully account for the observed relationships."

3. **Addressing concerns regarding high missingness and potential bias from imputation:** We addressed this concern through three complementary approaches: (1) transparently reporting the extent of missingness for each biomarker, (2) conducting sensitivity analyses using alternative imputation strategies, and (3) evaluating the stability of the observed associations across imputation methods.

(1) **Biomarker missingness:** We now report missingness rates for all biomarkers (**Supplementary Table 22**), showing that most biomarkers have low missingness (<15%), and the C-reactive protein has higher missing rate of ~60%.

- (Supplementary Information, p. 35)

Supplementary Table 22. Missing rate of biomarkers.

Features	Missing Rate (%)
Albumin	0.7

Glucose	0.8
Creatinine	2.8
Red blood cell distribution width	3.1
Mean cell volume	3.6
Lymphocyte percent	10.7
White blood cell count	12.5
Alkaline phosphatase	27.1
C-reactive protein	59.6

(2) **Sensitivity to imputation strategy:** To evaluate whether imputation method biases results, we performed extensive sensitivity analyses to assess the impact of the imputation strategy. Specifically, we implemented multiple imputation by chained equations (MICE) and derived PhenoAge using imputed biomarker values. The resulting PhenoAge estimates were highly correlated with those obtained using mean imputation across the overall sample and by sex (r=0.916-0.943; **Supplementary Fig. 8**), indicating that the overall ranking and distribution of biological aging measures were largely preserved across imputation approaches.

- (Supplementary Information, p. 11)

Supplementary Fig. 8 Correlation of PhenoAge estimates derived from mean imputation and multiple imputation (MICE). Pearson correlation coefficients (r).

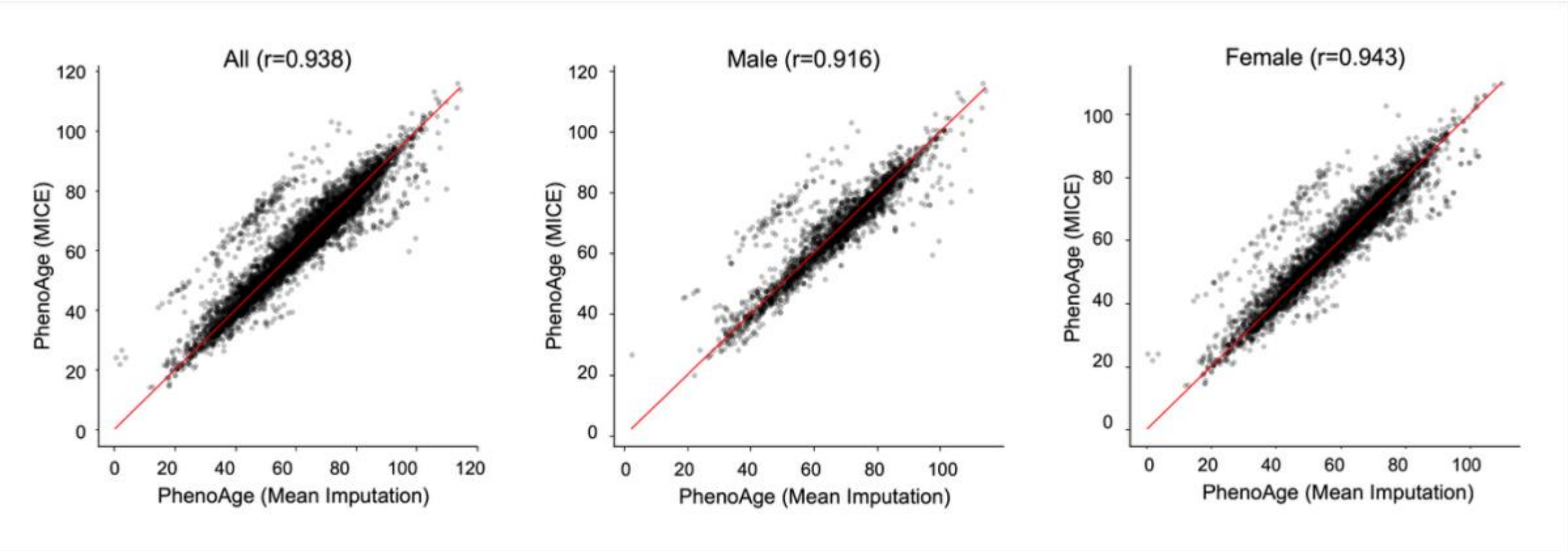

(3) **Robustness of associations:** We also compared the longitudinal associations between circadian rhythm features and biological aging across imputation methods (**Supplementary Table 12**). Effect estimates were highly consistent in both direction and magnitude. Key findings, including the protective associations of rhythm robustness (e.g., amplitude, relative amplitude, M10) and the adverse associations of nocturnal activity (e.g., L5), remained stable across methods. While minor differences in statistical significance were observed for some timing-related variables (e.g., acrophase, M10 start, L5 start), these differences were modest and did not alter the overall interpretation.

- (Supplementary Information, p. 19)

Supplementary Table 6. Comparison of longitudinal associations between circadian rhythm metrics and biological aging across imputation methods.

Features	Mean Imputation OR (95% CI)	*p*-value	MICE OR (95% CI)	*p*-value
fPC1	**0.832 (0.757, 0.915)**	**<0.001**	**0.818 (0.737, 0.884)**	**<0.001**
fPC2	1.059 (0.966, 1.162)	0.219	**1.108 (1.026, 1.195)**	**0.008**
fPC3	0.938 (0.857, 1.028)	0.172	0.962 (0.892, 1.036)	0.306
fPC4	1.016 (0.928, 1.113)	0.726	1.061 (0.984, 1.144)	0.124
MESOR	**0.654 (0.574, 0.744)**	**<0.001**	**0.649 (0.597, 0.707)**	**<0.001**
Amplitude	**0.623 (0.553, 0.702)**	**<0.001**	**0.637 (0.586, 0.693)**	**<0.001**
Acrophase	**1.150 (1.036, 1.277)**	**0.009**	1.034 (0.953, 1.122)	0.423
RA	**0.764 (0.682, 0.855)**	**<0.001**	**0.709 (0.649, 0.775)**	**<0.001**
M10	**0.612 (0.536, 0.698)**	**<0.001**	**0.632 (0.581, 0.688)**	**<0.001**
L5	1.055 (0.945, 1.178)	0.343	**1.138 (1.043, 1.242)**	**0.004**
M10 start	**1.133 (1.004, 1.278)**	**0.043**	1.061 (0.971, 1.159)	0.188
L5 start	1.052 (0.943, 1.173)	0.363	**1.090 (1.000, 1.188)**	**0.049**
IS	**0.895 (0.809, 0.991**	**0.033**	**0.843 (0.780, 0.910)**	**<0.001**
IV	0.891 (0.792, 1.002)	0.054	0.954 (0.881, 1.034)	0.468

All models are adjusted for age, sex, race/ethnicity, education, income, employment, lifetime smoking, current alcohol consumption, and comorbidities (cancer, CVD, type 2 diabetes). Longitudinal model includes a random intercept. Significant associations ($p<0.05$) are shown in bold.

These analyses demonstrate that our findings are robust to the choice of imputation method and are unlikely to be driven by the imputation strategy. This has been clarified in the Discussion, and the corresponding sensitivity analyses are provided in the Supplementary Information. → (Methods, p. 25; Results, p. 11, Discussion, p. 14; Supplementary Fig. 8 & Table 6)

- (Methods, p. 25) "To evaluate the robustness of this approach, we conducted sensitivity analyses using alternative imputation strategies, including multiple imputation by chained equations (MICE). PhenoAge estimates were recalculated using imputed biomarker values, and agreement between estimates derived from mean imputation and MICE was assessed. In addition, we evaluated whether associations between circadian rhythm metrics and biological aging were consistent across imputation methods."

- (Results, p. 11) "Our findings were also robust to the handling of missing biomarker data. Using multivariate imputation by chained equations (MICE), the resulting PhenoAge estimates were strongly correlated with those obtained using mean imputation across the overall sample and by sex (r=0.916-0.943). Furthermore, longitudinal associations between functional and circadian rhythm features and biological aging remained consistent in both magnitude and direction across imputation approaches (**Supplementary Fig. 8; Supplementary Table 6**)."

- (Discussion, p. 14) "Our findings were robust to the handling of missing biomarker data. Sensitivity analyses using multivariate imputation by chained equations imputation (MICE) yielded highly consistent results, with strong agreement in PhenoAge estimates and stable associations between circadian rhythm metrics and biological aging across imputation approaches. These results reduce the likelihood that our findings are driven by the choice of imputation method."

In conclusion, we (1) clarified the conceptual interpretation of PhenoAge as a clinically derived phenotypic aging measure distinct from a molecular "biological clock"; (2) evaluated and adjusted for potential bias from healthcare utilization and measurement frequency; (3) assessed the impact of missingness, including high CRP missingness, using alternative imputation strategies; and (4) demonstrated the robustness of findings across multiple sensitivity analyses. We also expanded the Discussion to better contextualize these limitations and interpretations within EHR-derived data. → (Discussion, p. 21)

- (Discussion, p. 21) "Importantly, PhenoAge in this study is a clinical biomarker-based phenotypic aging measure, distinct from a molecular "biological clock." It reflects integrated, multi-system physiological dysregulation captured through routine clinical biomarkers. While individual biomarkers are associated with chronic disease risk, the strength of clinical biomarker-based PhenoAge lies in its multivariate construction, which captures system-level aging processes beyond single-marker effects. Previous work has largely established its utility in cross-sectional settings, linking circadian activity patterns to mortality, disease incidence, systemic inflammation, and frailty."

2. Causal Interpretation and Residual Confounding
Although the authors acknowledge observational limitations, the tone at times verges on causal inference (e.g., "changes in rhythms shaped aging trajectories"). Moreover, key time-varying covariates such as medications, mental health, acute illness, or frailty markers are not adjusted for or considered. These could confound both activity rhythms and biomarker profiles. Stronger language cautioning against causal interpretation is warranted, and inverse probability weighting or marginal structural models should be considered.

Response 2, Reviewer 1: We thank the reviewer for this constructive feedback. In response, we have made three key revisions: we (1) removed causal language and now speaking about associations only throughout the manuscript; (2) incorporated time-varying covariates, including medication use, mental health, and acute health events, into the statistical models; and (3) conducted additional sensitivity analyses using inverse probability weighting.

1. **Removing causal language:** We revised the wording throughout the manuscript to avoid causal interpretation and consistently describe our findings as longitudinal associations. Specifically, statements implying causal effects (e.g., "shaped biological aging trajectories" or "reduced/increased risk") have been replaced with neutral expressions such as "were associated with" or "were prospectively associated with."

    - (p. 2) "Through high-dimensional digital phenotyping, we show that circadian rhythm intensity, timing, and stability are associated with biological aging trajectories. Higher rhythm intensity was related with 26-46% lower odds of accelerated aging. Associations of timing and regularity were stronger in females."

- (p. 8) “Across both time points, higher rhythm intensity (fPC1, MESOR, amplitude, RA, M10) and greater rhythm regularity (IS) were associated with lower odds of accelerated aging, whereas delayed timing metrics (acrophase, M10 start time, L5 start time) were associated with higher odds.”
- (p. 9) “To move beyond cross-sectional contrasts, we evaluated whether changes in rhythm were associated with changes in biological aging over time (Table 3).”
- (p. 9) “Higher activity intensity and rhythm robustness were associated with reduced odds of accelerated aging across the study period.”
- (p. 10) “Participants with increases in intensity metrics (MESOR, amplitude, RA, M10) were associated with negative ΔAge slopes, consistent with slower biological aging, whereas delays in acrophase and M10 start time produced positive ΔAge slopes, indicating accelerated aging trajectories.”
- (p. 10) “In contrast, delayed acrophase was associated with increased odds by 22.1% (OR=1.221; 95% CI: 1.015-1.468; *p*=0.034),…”
- (p. 10) “In males, higher fPC1, MESOR, amplitude, and were associated with lower odds of fast aging by 15-30%, whereas the effect sizes were larger in females, ranging from 22 to 40%. Increased rhythm regularity was related with lower odds of accelerated aging by 15% in females, while showing no statistically significant effect in males. Similarly, delayed acrophase was associated with higher odds of fast aging by 12% in males and 18% in females but was statistically significant only in females.”
- (p. 13) “In this large-scale, prospective cohort with multi-year wearable monitoring in free-living settings, we demonstrated that circadian rest-activity rhythms are robust correlates of biological aging trajectories, assessed using clinical biomarker-based PhenoAge.”
- (p. 13) “Longitudinal analyses indicated that changes in rest-activity rhythms prospectively associated with biological aging trajectories across five years.”
- (p. 12) “Rhythm intensity was consistently associated with lower odds across both sexes, while reduced regularity and delayed timing were specific risk factors in females.”
- (p. 13) “Specifically, higher rhythm intensity was associated with lower odds of accelerated aging by 26-46%, greater regularity by 9-13%, whereas delayed timing was associated with higher odds by 22%.”
- (p. 17) “Consistent with this, our findings demonstrated that delayed acrophase was prospectively associated with accelerated aging, suggesting a potential link between rest-activity timing disruption and long-term aging trajectories.”
- (p. 17) “Rhythm intensity was broadly protective in both sexes, but timing- and regularity-related associations were markedly stronger in females, where delayed acrophase and later rest onset was associated with higher odds of accelerated aging by 12-18% and greater regularity was related to lower odds by 15%.”
- (p. 22) “Building on this foundation, this study provides longitudinal evidence that wearable-derived circadian rhythmicity metrics are associated with biological aging trajectories. By leveraging both conventional CRAR metrics and functional data analysis, we show that rhythm intensity is consistently associated with slower PhenoAge acceleration, while timing and fragmentation show sex-specific associations.”
- (p. 22) “If future interventional studies demonstrate that modifying these digital phenotypes can alter aging trajectories, wearable-guided circadian interventions may redefine preventive medicine for healthy longevity.”

2. **Incorporating time-varying covariates:** To rigorously account for potential time-varying confounding, we extended our models by incorporating covariates that vary across participant-years and are temporally aligned with activity rhythm measurement period. Specifically, we included time-varying measures of medication use (e.g., antihypertensive agents, lipid-lowering agents (statins), antidiabetic medications, and CNS-active medications (antidepressants and sedatives)), indicators of mental health status (e.g., depression diagnosis), and acute health events (e.g., hospitalization visits, ER visits). These variables were included as additional covariates in the statistical models in addition to originals covariates of demographics, socioeconomics, and time-varying comorbities (e.g., cardiovascular diseases, diabetes, and cancer).

    As shown in **Table R1**, adjustment for these additional time-varying covariates did not substantially change the estimated associations, in either magnitude or direction, indicating that the primary findings are not driven by time-dependent confounding. For instance, higher rest-activity rhythm intensity, captured by fPC1, MESOR, amplitude, RA, and M10, remained consistently associated with lower odds of accelerated aging, in both cross-sectional and longitudinal analyses. In contrast, delayed activity timing, such as delayed acrophase, M10 start time, and L5 start time, was associated with higher odds. Greater rhythm stability (IS) was associated with reduced odds of accelerated aging. The persistence of effect supports the robustness and internal consistency of the observed relationships across model specifications.

Table R1. Sensitivity analyses accounting for time-varying medication, mental health, and acute health events in association of functional and circadian rhythm metrics on accelerated biological aging.

	Baseline		Follow-up		Longitudinal	
**Features**	**OR (95% CI)**	***p*-value**	**OR (95% CI)**	***p*-value**	**OR (95% CI)**	***p*-value**
fPC1	**0.852 (0.783, 0.928)**	**<0.001**	1.089 (0.928, 1.278)	0.295	**0.836 (0.753, 0.928)**	**0.001**
fPC2	1.018 (0.938, 1.105)	0.665	1.103 (0.947, 1.285)	0.206	1.030 (0.932, 1.139)	0.558
fPC3	0.975 (0.899, 1.057)	0.539	0.972 (0.819, 1.155)	0.750	0.967 (0.876, 1.067)	0.501
fPC4	0.994 (0.916, 1.078)	0.878	1.048 (0.900, 1.220)	0.547	1.022 (0.926, 1.128)	0.663
MESOR	**0.824 (0.755, 0.898)**	**<0.001**	**0.796 (0.690, 0.918)**	**0.002**	**0.655 (0.576, 0.745)**	**<0.001**
Amplitude	**0.824 (0.757, 0.897)**	**<0.001**	**0.788 (0.683, 0.908)**	**0.001**	**0.640 (0.564, 0.727)**	**<0.001**
Acrophase	**1.086 (1.001, 1.179)**	**0.048**	1.038 (0.906, 1.189)	0.591	**1.117 (1.006, 1.241)**	**0.039**
RA	**0.865 (0.795, 0.940)**	**0.001**	0.892 (0.774, 1.028)	0.115	**0.744 (0.660, 0.838)**	**<0.001**
M10	**0.808 (0.741, 0.881)**	**<0.001**	**0.766 (0.665, 0.883)**	**<0.001**	**0.621 (0.545, 0.708)**	**<0.001**
L5	1.032 (0.950, 1.121)	0.459	0.974 (0.849, 1.117)	0.702	1.051 (0.936, 1.181)	0.399
M10 start	1.044 (0.960, 1.135)	0.316	1.061 (0.927, 1.214)	0.390	1.082 (0.960, 1.219)	0.198
L5 start	1.069 (0.984, 1.162)	0.115	1.060 (0.926, 1.213)	0.399	1.035 (0.922, 1.163)	0.556
IS	**0.916 (0.843, 0.996)**	**0.039**	0.899 (0.783, 1.031)	0.127	**0.893 (0.803, 0.993)**	**0.036**
IV	0.963 (0.887, 1.046)	0.373	0.960 (0.838, 1.099)	0.552	0.891 (0.762, 1.011)	0.105

All models are adjusted for age, sex, race/ethnicity, education, income, employment, lifetime smoking, current alcohol consumption, comorbidities (cancer, CVD, type 2 diabetes), medication use

(antihypertensive, statins, antidiabetic, and CNS-active medications), depression, and acute health events (hospitalization, ER visits). Longitudinal model includes a random intercept and model comorbidities, medication use, depression, and acute health events as time-varying covariates. Significant associations (*p*<0.05) are shown in bold.

3. **Inverse probability weighting:** To further account for time-varying confounding, we conducted sensitivity analyses using inverse probability of treatment weighting (IPTW). For each wearable-based metric, we defined binary exposure groups based on quartiles: for intensity-related and fPCA metrics (fPC1-4, MESOR, amplitude, RA, M10, IS), the lowest quartile was defined as low exposure and the remaining as high (reference), whereas for timing- and fragmentation-related metrics (acrophase, L5, M10 start time, L5 start time, IV), the highest quartile was defined as high exposure and the remaining as low (reference). Stabilized weights were estimated using models incorporating demographics, socioeconomic factors, and time-varying covariates, including comorbidities, medication use, mental health status, and acute illness.

    As shown in **Supplementary Table 5**, IPTW-adjusted estimates were largely consistent with the primary analyses, with several associations becoming stronger or reaching statistical significance. Higher circadian intensity and robustness metrics, including fPC1 (OR=0.74, 95% CI: 0.64-0.86), MESOR (OR=0.64, 95% CI: 0.55-0.73), amplitude (OR=0.60, 95% CI: 0.53-0.69), RA (OR=0.70, 95% CI: 0.61-0.81), M10 (OR=0.60, 95% CI: 0.53-0.69), and IS (OR=0.80, 95% CI: 0.70-0.92), were associated with reduced odds of accelerated aging. Conversely, higher fPC4 (OR=1.16, 95% CI: 1.00-1.35), later M10 start time (OR=1.15, 95% CI: 1.00-1.32), and later L5 start time (OR=1.15, 95% CI: 1.01-1.32) were significantly associated with increased odds of accelerated biological aging.

    Other features, including fPC2, fPC3, L5, and IV, showed directionally consistent but non-significant associations. Overall, these findings indicate that adjustment for time-varying confounding does not change the observed relationships and, in some cases, strengthens the estimated effects, supporting the robustness of the primary results.

- (Supplementary Information, p. 18)

Supplementary Table 5. IPTW-weighted associations between wearable-derived circadian metrics and accelerated biological aging.

Features	OR (95% CI)	*p*-value
fPC1	**0.740 (0.639, 0.858)**	**<0.001**
fPC2	1.129 (0.973, 1.309)	0.109
fPC3	0.965 (0.833, 1.119)	0.639
fPC4	**1.163 (1.002, 1.349)**	**0.047**
MESOR	**0.635 (0.553, 0.728)**	**<0.001**
Amplitude	**0.603 (0.525, 0.692)**	**<0.001**
Acrophase	**1.213 (1.057, 1.391)**	**0.006**
Relative amplitude (RA)	**0.704 (0.613, 0.807)**	**<0.001**
M10	**0.603 (0.525, 0.692)**	**<0.001**
L5	1.106 (0.964, 1.269)	0.150

M10 start time	**1.152 (1.004, 1.322)**	**0.043**
L5 start time	**1.154 (1.006, 1.324)**	**0.041**
Interdaily stability (IS)	**0.799 (0.697, 0.917)**	**0.001**
Intraday variability (IV)	0.863 (0.731, 1.021)	0.080

Odds ratios (ORs) and 95% confidence intervals (CIs) were estimated using inverse probability of treatment weighting (IPTW). Binary exposure groups were defined based on quartiles for each metric. Weighting models incorporated demographic, socioeconomic, and time-varying covariates, including age, sex, race/ethnicity, education, income, employment, lifetime smoking, current alcohol consumption, comorbidities (cancer, cardiovascular disease, and type 2 diabetes), medication use (antihypertensive, statins, antidiabetic, and CNS-active medications), depression, and acute health events (hospitalization and emergency room visits).

In summary, these additional analyses collectively strengthen the robustness of our findings. Adjustment for time-varying covariates, including medication use, mental health, and acute health events, did not change the observed associations. Furthermore, IPTW analyses, which explicitly account for time-varying exposure and confounding, yielded consistent or stronger effect estimates across key circadian features. These results demonstrate that the observed associations between circadian rest-activity rhythms and biological aging trajectories are stable across multiple analytical approaches and are unlikely to be driven by measured time-varying confounders. We have incorporated these clarifications into the revised manuscript and Supplementary Information. → (Methods, p. 31; Results, p. 11; Discussion, p. 14; Supplementary Table 5)

- (Methods, p. 31) "To account for time-varying confounding, we additionally conducted sensitivity analyses using inverse probability of treatment weighting (IPTW). For each wearable metric, binary exposure groups were defined based on quartiles. For intensity-related and fPCA-derived metrics (fPC1-4, MESOR, amplitude, RA, M10, and IS), the lowest quartile was defined as low exposure and the remaining as high (reference), whereas for timing- and fragmentation-related metrics (acrophase, L5, M10 start time, L5 start time, and IV), the highest quartile was defined as high exposure and the remaining as low (reference). Stabilized weights were estimated using logistic models incorporating demographic, socioeconomic, and time-varying covariates, including comorbidities, medication use, mental health status, and acute health events. Weighted logistic regression models were then fitted to estimate associations between wearable-derived circadian metrics and accelerated biological aging."

- (Results, p. 11) "To assess the robustness of our findings, we performed extensive sensitivity analyses. In particular, to address time-varying confounding, including comorbidities, medication use, and acute health events, we conducted inverse probability of treatment weighting (IPTW) analyses (**Supplementary Table 5**). The IPTW-adjusted estimates were largely consistent with the primary analyses, with several associations strengthened or reaching statistical significance. Specifically, higher circadian intensity metrics remained associated with reduced odds of accelerated aging, whereas delayed timing metrics were associated with increased odds, indicating that the observed associations are stable after accounting for time-varying confounding."

- (Discussion, p. 14) "An important consideration in longitudinal observational studies is time-varying confounding, particularly from evolving comorbidities, medication use, and acute health events, which may both influence and be influenced by circadian rhythms. To address this, we conducted inverse probability of treatment weighting (IPTW) analyses that account for these dynamic factors. The resulting estimates remained consistent or were strengthened, indicating that the observed associations are not readily explained by time-varying confounding."

3. Biological Interpretation of fPCs
The fPCA approach is elegant but opaque. While fPC1 aligns well with rhythm amplitude, fPC3 and fPC4 (e.g., "biphasic instability") are harder to interpret physiologically. The authors should clarify whether these patterns correspond to known biological phenomena (e.g., chronotype, sleep fragmentation, nap patterns) or may be device artifacts. Without biological anchoring (e.g., hormonal rhythms, inflammatory markers), the link between latent components and aging trajectories is speculative. Perhaps incorporation of the Fitbit-derived sleep data would be complementary (albeit imperfect).

Response 3, Reviewer 1: We appreciate the reviewer for this important point regarding the physiological interpretability of fPC3 and fPC4. In response, we conducted additional analyses to (1) quantify alignment with established circadian rhythm metrics, (2) evaluate correspondence with independently derived Fitbit sleep measures, and (3) assess consistency with known behavioral physiology rather than device artifacts.

1. **Alignment with circadian rhythm metrics:** We first examined correlations between fPCs and standard circadian rhythm metrics (**Supplementary Fig. 4**). fPC3 demonstrated moderate-to-strong associations with activity timing measures, including M10 start time (r= −0.60), which indexes the onset of the most active 10-hour period, and acrophase (r= −0.30), representing the timing of peak activity within the 24-hour rest-activity rhythm cycle. The consistent alignment across multiple timing metrics indicates that fPC3 primarily captures variation in circadian phase, reflecting shifts in rest-activity timing (i.e., morningness-eveningness), a proxy for chronotype.

   In contrast, fPC4 showed its strongest association with intradaily variability (IV; r= −0.20), a measure of within-day fragmentation of rest-activity rhythms. fPC4 exhibited minimal correlation with intensity (e.g., MESOR, amplitude) or canonical timing metrics, indicating that it reflects irregular or fragmented activity organization rather than shifts in activity level or circadian phase.

- (Supplementary Information, p. 7)

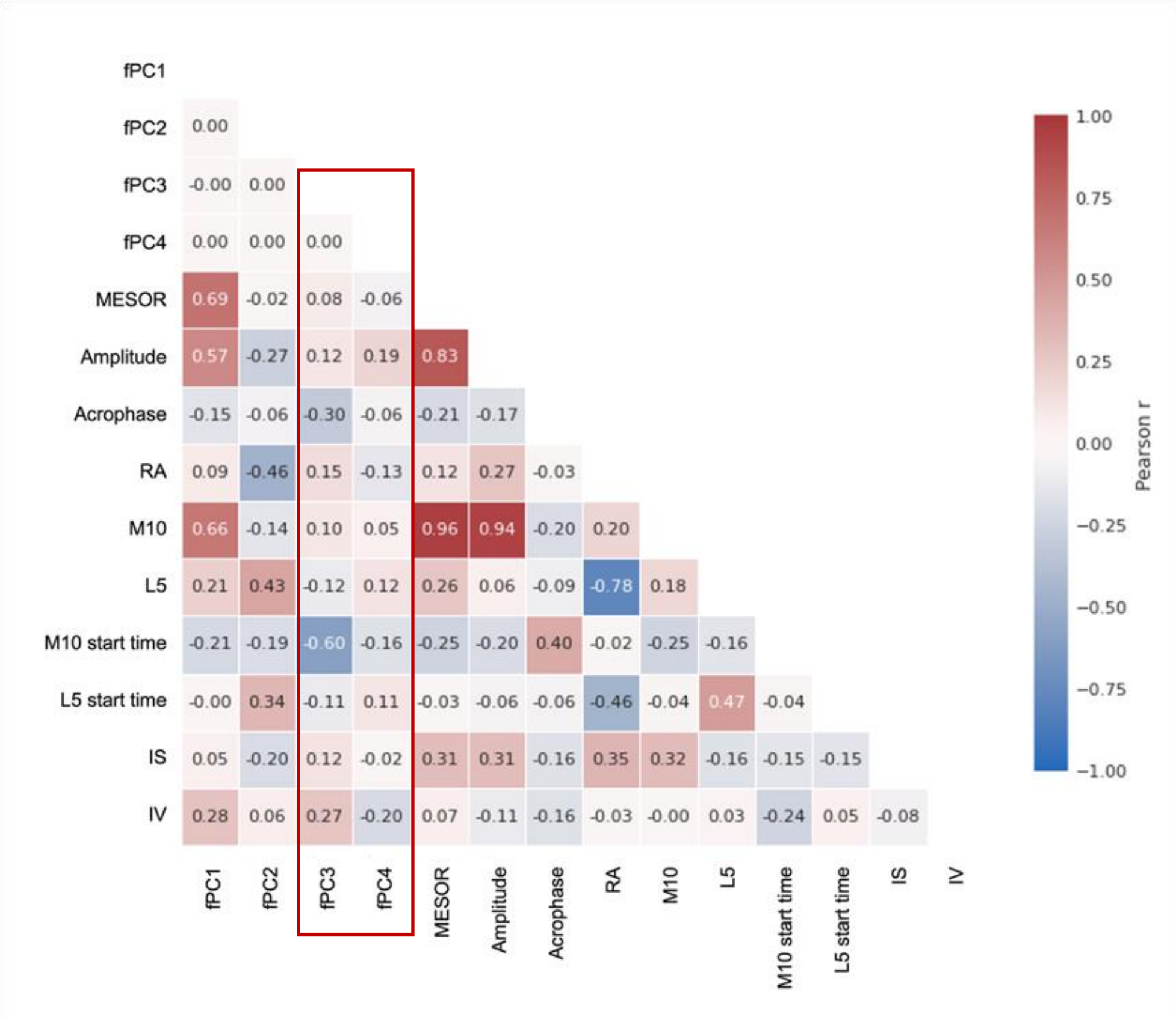

Supplementary Fig. 4 Pearson correlation matrix between functional principal components (fPC1-fPC4) and CRAR metrics. Color scale represents the strength and direction of correlations (red=positive, blue=negative), with numerical values indicating Pearson's *r*.

2. **Alignment with sleep metrics:** To evaluate whether fPCs map onto sleep-related phenomena such as chronotype, sleep timing, and nap behavior, we incorporated Fitbit-derived sleep daily summary and sequence-level data. We reconstructed sleep episodes at the sequence level (main sleep defined by is_main_sleep = TRUE, nap sleep defined by is_main_sleep = FALSE, with ≥30-minute gaps used to define separate episodes). Sleep stages were harmonized across device-specific categories.

    Among 2,222 participants, 2,104 had valid overlapping sleep and activity data, where we obtained sleep duration, sleep onset time, sleep end time, sleep midpoint, sleep efficiency, nap count, nap duration, and nap onset time. We then examined correlations between fPCs and these sleep metrics.

    At the episode level, *sleep onset* was defined as the earliest start time and wake time as the latest end time, with *sleep duration* calculated as the elapsed time between these time points. *Sleep midpoint* was defined as the temporal midpoint between sleep onset and wake time. *Sleep efficiency* was defined as the ratio of minutes asleep to time in bed.

    Daytime sleep (naps) was identified from non-main sleep episodes using the same episode definition. For each participant, we computed *nap count*, *nap duration*, and *nap onset time*. All sleep metrics were first calculated at the daily level and subsequently aggregated to the participant-year level by averaging across valid days.

- (Supplementary Information, p. 8)

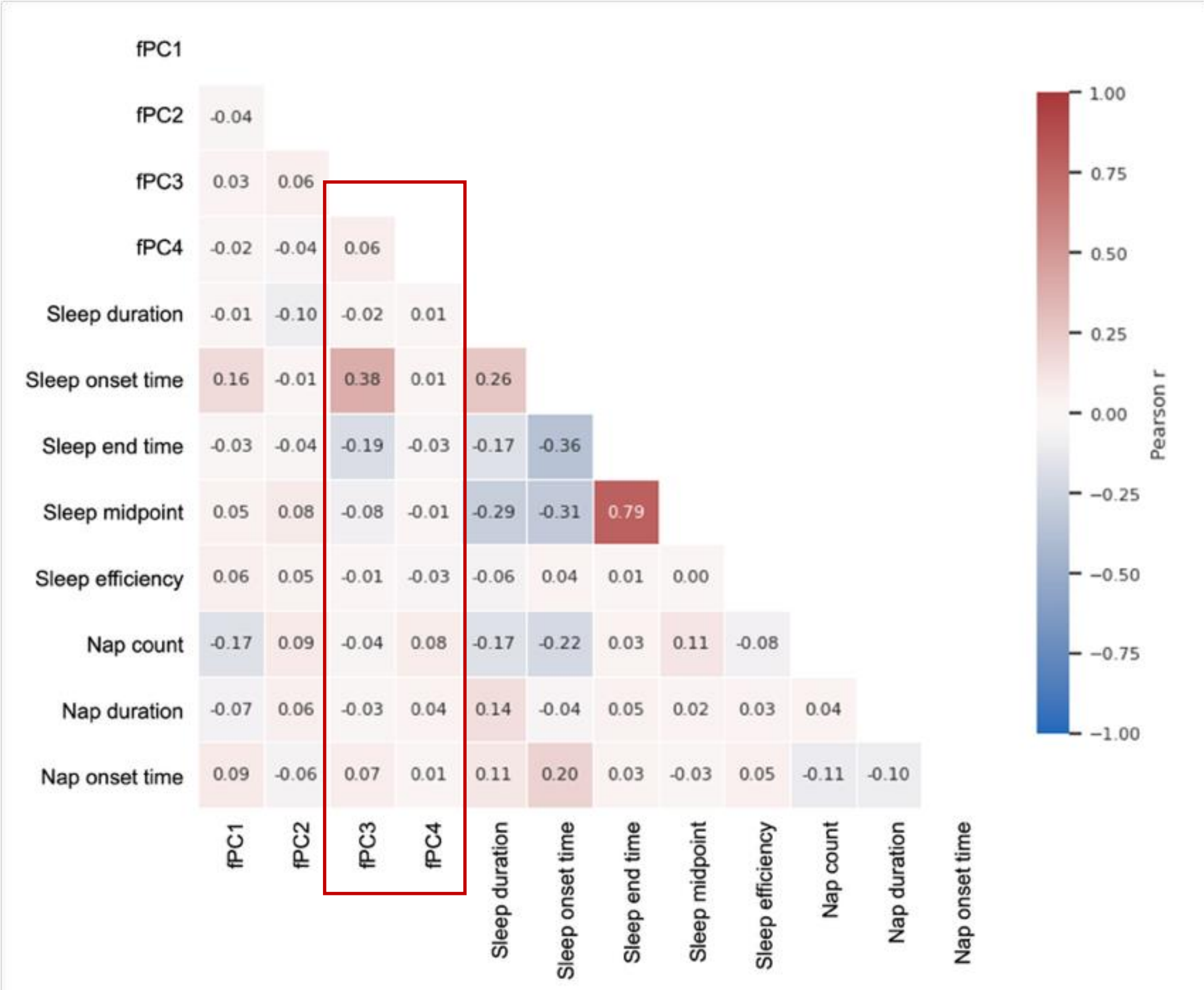

Supplementary Fig. 5 Pearson correlation matrix between functional principal components (fPC1-fPC4) and sleep metrics. Color scale represents the strength and direction of correlations (red=positive, blue=negative), with numerical values indicating Pearson's r.

As shown in **Supplementary Fig. 5**, fPC3 demonstrated consistent associations with sleep onset (r=0.38) and sleep end timing (r= −0.19). The opposing directions of these associations indicate a coordinated shift in the sleep–wake schedule, rather than isolated changes in individual time points. This pattern is consistent with the activity-based timing results, supporting that fPC3 captures a systematic shift in overall circadian phase across the daily cycle. In contrast, fPC4 showed uniformly weak associations across all sleep metrics, with the largest correlation observed for nap frequency (r=0.08). No single sleep dimension, timing, duration, or efficiency, demonstrated a dominant relationship with fPC4. This pattern indicates that fPC4 does not map onto a specific sleep construct, but instead captures distributed irregularity in daily activity organization, consistent with its association with intradaily variability.

In summary, these findings demonstrate cross-domain consistency for fPC3 as a circadian rest-activity timing component across both activity and sleep measures, while fPC4 reflects fragmentation-related structure that is not reducible to conventional sleep metrics.

3. **Consistency with behavioral physiology and exclusion of device artifacts:** Finally, our findings are consistent with known behavioral physiology and are unlikely to be explained by device-related artifacts. Specifically, fPC1 aligns with activity intensity metrics, fPC3 aligns specifically with timing-related measures across both activity (e.g., M10 start time, acrophase) and independently derived sleep metrics (e.g., sleep onset

and end timing), and fPC4 aligns with fragmentation (i.e., IV), without corresponding associations with intensity or phase. This domain-specific alignment is not likely due to a device artifact explanation and instead suggests that each component captures a distinct dimension of circadian rest-activity rhythm organization.

This interpretation is further supported by cross-domain consistency. The alignment of fPC3 with both Fitbit activity-derived activity timing and independently measured Fitbit sleep-derived timing, as shown in **Supplementary Fig. 5**, indicates reproducibility across distinct measurement modalities, reducing the likelihood that the observed patterns are driven by device-specific biases. In addition, circadian rest-activity rhythm (CRAR) metrics exhibited high longitudinal stability between baseline and follow-up (**Supplementary Fig. 10**), indicating that the underlying signal is robust over time and not dominated by measurement variability or device-related artifacts.

- (Supplementary Information, p. 13)

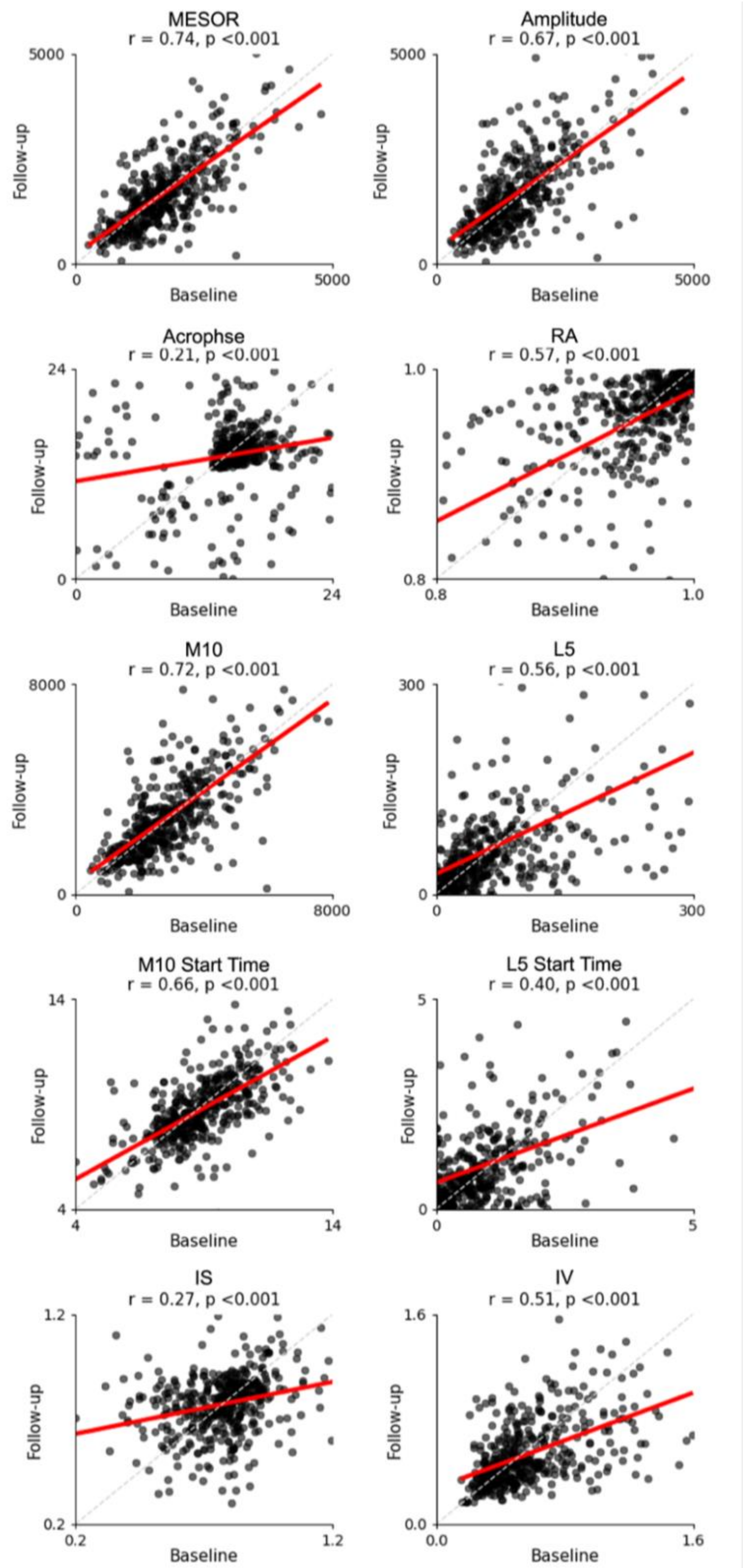

Supplementary Fig. 10 Longitudinal stability of CRAR metrics. Scatter plots comparing baseline and follow-up values for circadian rest-activity rhythm (CRAR) metrics. Each point represents an individual participant. Red lines indicate linear regression fits, and dashed lines represent the identity line. Pearson correlation coefficients (r) and p-values are shown.

We have revised the manuscript and Supplementary Information to add this information. → (Methods, p. 27, p. 31; Results, p. 7; Discussion, p. 15; Supplementary Fig. 5 & Fig. 10)

- (Methods – New subsection added, p. 27)
  "**Assessment of sleep metrics**
  Sleep metrics were derived from Fitbit daily summary and sequence-level sleep data. Sleep episodes were reconstructed from timestamped sleep stage records by ordering consecutive segments within each participant and defining distinct episodes using gaps of ≥30 minutes. Main sleep episodes were identified using records with `is_main_sleep = TRUE`, while non-main sleep records (`is_main_sleep = FALSE`) were used to characterize daytime napping behavior. For each episode, sleep onset was defined as the earliest start time and wake time as the latest end time, and sleep duration was calculated as the elapsed time between onset and wake. Sleep midpoint was defined as the temporal midpoint between sleep onset and wake time.
  From the main sleep episodes, we derived total sleep duration, sleep onset time, sleep end time, sleep midpoint, and sleep efficiency, calculated as the ratio of minutes asleep to minutes in bed. Daytime sleep (naps) was identified from non-main sleep episodes using the same episode definition. For each participant, we derived nap count, average nap duration, and mean nap onset time. All sleep metrics were first computed at the daily level and subsequently aggregated to the participant-year level by averaging across valid days. These sleep-derived measures were used to evaluate correspondence between activity-derived circadian patterns and independently measured sleep behaviors."

- (Methods – Statistical analysis, p. 31) "… Finally, we quantified the correspondence between fPCs and Fitbit-derived sleep metrics to assess cross-domain consistency in circadian, sleep, and behavioral patterns."

- (Results, p. 7) "To further evaluate whether the fPCs correspond to sleep-related behavioral patterns, we examined their associations with independently derived Fitbit sleep metrics at the sequence level (**Supplementary Fig. 5**), focusing on components reflecting timing and fragmentation. fPC3 showed consistent associations with sleep timing measures, including sleep onset (r=0.38) and sleep end time (r= −0.19), indicating a coordinated shift in the sleep-wake schedule. These findings align with the activity-based timing associations observed for fPC3, supporting its interpretation as a circadian phase-related component. In contrast, fPC4 showed weak associations across sleep metrics, with the largest correlation observed for nap frequency (r=0.08), and no dominant relationship with sleep timing, duration, or efficiency, suggesting that fPC4 may capture distributed irregularity in daily activity organization rather than a specific sleep construct."

- (Discussion, p. 15) "Importantly, the interpretation of these fPCs is further informed by their alignment with independently derived sleep measures. While fPC1 and fPC2 are well anchored to CRAR metrics, fPC3 and fPC4 require additional context. In our additional analyses leveraging Fitbit-derived sleep data, fPC3, which reflects variation in activity timing, was associated with sleep onset and wake timing,

indicating coordinated shifts in the sleep–wake schedule across domains. By contrast, fPC4 showed only weak and non-specific associations with sleep metrics, including a modest relationship with nap frequency, without a consistent pattern across timing, duration, or efficiency. This distinction suggests that fPC3 captures a coherent timing-related dimension observable across behavioral modalities and is consistent with a circadian timing-related construct, providing support for its biological plausibility. In contrast, fPC4 reflects more diffuse irregularity in daily activity organization that is not readily captured by conventional sleep measures."

Minor Comments:

• Abstract: Suggest clarifying that the "PhenoAge" application here is not the same as the reference provided from Levine et al, which included epigenetic changes in the model. Otherwise, the reader is left concluding that the application presented here has been studied in this way.

Response 4, Reviewer 1: We thank the reviewer for this valuable suggestion. As addressed in Response 1, Reviewer 1, we clarified that all analyses are based on clinical biomarker-based PhenoAge derived from blood chemistry measures, rather than epigenetic DNAm PhenoAge. Specifically, we now define this distinction at first mention and have ensured consistent use of the term "clinical biomarker–based PhenoAge" throughout the manuscript to avoid ambiguity.

• Figures: Figure 2 (fPC plots) could benefit from clearer labeling of axes and units (e.g., what does a "score of 1" mean in activity terms?).

Response 5, Reviewer 1: We have revised the y-axis labeling and the caption of Figure 2 to improve clarity. Specifically, panel (b) now shows group-level deviations from the population mean, computed as the mean fPC score multiplied by the corresponding eigenfunction for each biological aging group. These curves illustrate how circadian rest-activity patterns differ across fast aging vs. slow aging groups. This representation is analogous to standard PCA, where component scores scale the corresponding principal components to reconstruct deviations from the mean pattern. fPC scores were standardized (mean=0, variance=1), and eigenfunctions were scaled to the range [-1, 1] for visualization. We have revised the manuscript accordingly. → (Fig. 2, p. 4)

- (Fig. 2, p. 4)

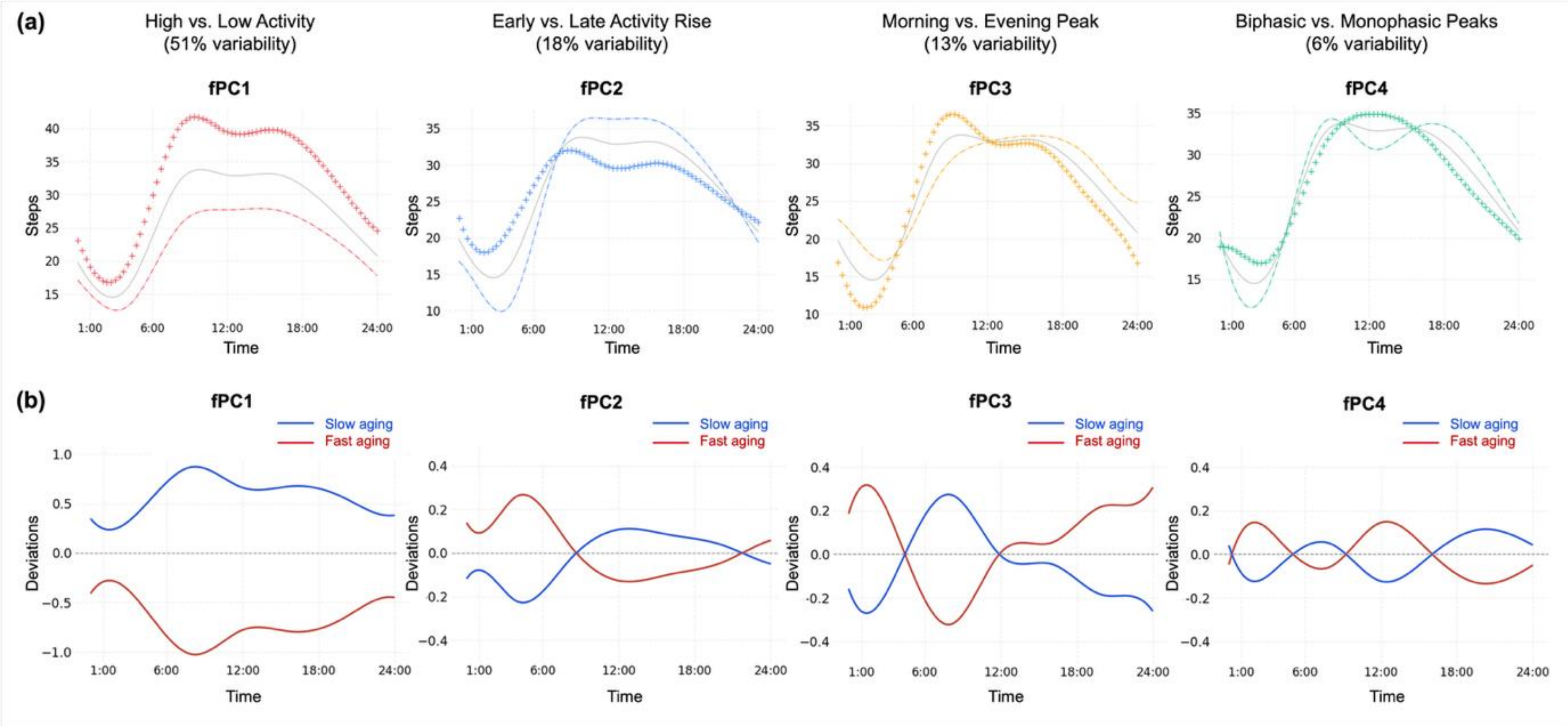

Fig. 2 Functional principal components (fPCs) of 24-hour activity profiles and their association with biological age acceleration. (a) The mean 24-hour activity profile (gray) and variation along each functional principal component are shown. Colored curves represent positive (++++) and negative (----) deviations from the mean trajectory (i.e., mean ± scaled eigenfunctions), illustrating the dominant temporal patterns captured by each fPC. Mean 24-hour activity profiles are plotted separately by the sign of each fPC score, illustrating the predominant temporal patterns captured by each component. **(b)** Line plots depict group-level deviations from the population mean, computed as the mean fPC score multiplied by the corresponding eigenfunction for each biological aging group. These curves illustrate how circadian rest-activity patterns differ across fast aging vs. slow aging groups. fPC scores were standardized (mean=0, variance=1), and eigenfunctions were scaled to the range [-1, 1] for visualization.

• Table 1: Consider including comorbidity burden (e.g., Charlson index) or medication use to help contextualize aging status groups.

Response 6, Reviewer 1: We thank the reviewer for this constructive suggestion. In response, we have added the Charlson Comorbidity Index (CCI) and medication use to Table 1.

1. **Charlson Comorbidity Index (CCI):** CCI was calculated using ICD-9 and ICD-10 diagnosis codes based on the coding algorithms proposed by Glasheen et al. (2019)[13]. Specifically, comorbid conditions were identified using predefined ICD code groupings, and the CCI was computed as the weighted sum of these conditions. As the All of Us data are structured in the Observational Medical Outcomes Partnership (OMOP) common data model, ICD-9 and ICD-10 codes were retrieved using the *condition_source_value* field. We categorized individuals into CCI groups of 0, 1, 2, and ≥3, as presented in Table 1.

   The distribution of CCI differed significantly between the fast and slow aging groups at baseline. Individuals with no comorbidity (CCI=0) were more prevalent in the slow aging group (43.8% vs 30.9%), indicating that a healthier baseline is associated with slower biological aging. In contrast, a high comorbidity burden (CCI≥3) was substantially more common in the fast aging group (32.4% vs 20.3%), suggesting a strong link between multimorbidity and accelerated aging. Intermediate levels (CCI score of 1 or 2) showed minimal differences between two groups, indicating that the observed separation is largely driven by the extremes of comorbidity burden rather than moderate levels.

2. **Medication Use:** We focused on major medication classes with relevance to cardiometabolic and neurocognitive aging based on recent research on medication and biological aging[14]. Specifically, we included antihypertensive medications, lipid-lowering agents (statins), antidiabetic medications, and CNS-active medications (antidepressants/sedatives). Medication use was defined based on prescriptions recorded during the same calendar year as the baseline assessment using RxNorm ingredient concepts mapped to Anatomical Therapeutic Chemical (ATC) classes. Drug exposures were identified through descendant concepts using the OMOP *concept_ancestor* table in the All of Us Research Program database.

   As shown in updated Table 1, all medication classes were more prevalent in the fast aging group compared to the slow aging group, with the largest differences observed for antidiabetic medications (18.5% vs 5.2%) and antihypertensive use (45.4% vs 30.0%). These patterns indicate a higher overall treatment burden and greater cardiometabolic and neurocognitive morbidity among individuals with accelerated biological aging at baseline.

To enhance transparency and reproducibility, we have revised the manuscript to include the full list of ICD and concept ID codes used to define CCI and medication use in the Supplementary Information (**Supplementary Tables 20-21**). Corresponding updates have been made in the Methods and Results sections. → (Methods, p. 29; Results, p. 5; Supplemental Tables 20-21)

- (Methods, p. 29) "<u>In addition, we quantified overall comorbidity burden using the Charlson Comorbidity Index (CCI) using ICD codes</u>[13] <u>(**Supplementary Table 20**).</u> <u>CCI was computed as the weighted sum of predefined comorbid conditions and</u>

categorized as 0, 1, 2, and ≥3. We further assessed medication use as a proxy for treatment burden and underlying health status. We focused on major medication classes relevant to cardiometabolic and neurocognitive aging based on recent research on medication and biological aging[14], including antihypertensive medications, lipid-lowering agents (statins), antidiabetic medications, and central nervous system (CNS)-active medications (antidepressants and sedatives) (**Supplementary Table 21**)."

- (Results, p. 5) "However, comorbidity burden, as measured by the Charlson Comorbidity Index (CCI), differed significantly between groups (p<0.001). Individuals with no comorbidity (CCI=0) were more prevalent among slow agers (43.8% vs. 30.9%), whereas a high comorbidity burden (CCI≥3) was more common among fast agers (32.4% vs. 20.3%), indicating that differences were primarily driven by the extremes of comorbidity burden. Similarly, medication use differed significantly by biological aging status (all p<0.001). Fast agers had a higher prevalence of antihypertensive (45.4% vs. 30.0%), statin (29.7% vs. 18.9%), antidiabetic (18.5% vs. 5.2%), and CNS-active medication use (16.7% vs. 9.8%) compared to slow agers. These patterns suggest a higher treatment burden and greater cardiometabolic and neurocognitive morbidity among individuals with accelerated biological aging."

- (Table 1, p. 1)

Table 1. Baseline characteristics of the study population by biological aging status.

Mean (SD) or n (%)	All (n=2222)	Fast Aging (n=1021)	Slow Aging (n=1201)	*p*-value
Age, years	60.62 (14.01)	61.09 (13.82)	60.23 (14.17)	0.147
PhenoAge, years	59.90 (15.72)	66.43 (14.57)	54.34 (14.48)	<0.001
Sex				<0.001
Female	1522 (68.5)	636 (62.3)	886 (73.8)	
Male	693 (31.2)	380 (37.2)	313 (26.1)	
Race				0.008
White	1839 (82.8)	838 (82.1)	1001 (83.3)	
Black	134 (6.0)	78 (7.6)	56 (4.7)	
Other	249 (11.2)	105 (10.3)	144 (12.0)	
BMI, kg/m^2	29.84 (6.98)	31.76 (7.48)	28.21 (6.07)	<0.001
Education				0.187
College or above	2084 (93.8)	958 (93.8)	1126 (93.8)	
12 years or GED	119 (5.4)	50 (4.9)	69 (5.7)	
Income				<0.001
High	1015 (45.7)	428 (41.9)	587 (48.9)	
Medium	669 (30.1)	303 (29.7)	366 (30.5)	
Low	395 (17.8)	224 (21.9)	171 (14.2)	
Missing	143 (6.4)	66 (6.5)	77 (6.4)	
Employment				0.011
Employed	1260 (56.7)	564 (55.2)	696 (58.0)	
Retired	732 (32.9)	345 (33.8)	387 (32.2)	

Unemployed	159 (7.2)	88 (8.6)	71 (5.9)	
Other	71 (3.2)	24 (2.4)	47 (3.9)	
Smoking				0.048
Yes	788 (35.5)	394 (38.6)	394 (32.8)	
No	1434 (64.5)	627 (61.4)	807 (67.2)	
Drinking				0.36
Yes	2168 (97.6)	1000 (97.9)	1168 (97.3)	
No	54 (2.4)	21 (2.1)	33 (2.7)	
Comorbidities				
Cancer	207 (9.3)	92 (9.0)	115 (9.6)	0.702
CVD	247 (11.1)	107 (10.5)	140 (11.7)	0.417
Type 2 diabetes	212 (9.5)	97 (9.5)	115 (9.5)	1.000
Daily steps	7715.83 (3429.04)	7215.22 (3374.21)	8141.42 (3419.17)	<0.001
Sleep duration, hours	6.39 (1.67)	6.32 (1.67)	6.46 (1.66)	0.048
No recent healthcare contact	181 (8.1)	84 (8.2)	97 (8.1)	0.959
Limited healthcare access	518 (23.3)	232 (22.7)	286 (23.8)	0.578
Logistic barrier	429 (19.3)	202 (19.8)	227 (18.9)	0.637
Finance barrier	312 (14.0)	147 (14.4)	165 (13.7)	0.701
Length of follow-up, years	6.84 (2.2)	6.77 (2.23)	6.90 (2.15)	0.165
Charlson Comorbidity Index (CCI) score				<0.001
0	841 (37.8)	315 (30.9)	526 (43.8)	
1	463 (20.8)	212 (20.8)	251 (20.9)	
2	343 (15.4)	163 (16.0)	180 (15.0)	
≥3	575 (25.9)	331 (32.4)	244 (20.3)	
Medication use				
Antihypertensive	824 (37.1)	464 (45.4)	360 (30.0)	<0.001
Statin	530 (23.9)	303 (29.7)	227 (18.9)	<0.001
Antidiabetic	251 (11.3)	189 (18.5)	62 (5.2)	<0.001
CNS-active	289 (13.0)	171 (16.7)	118 (9.8)	<0.001

Values are mean (SD) for continuous variables and n (%) for categorical variables. *p*-values are based on *t*-tests or chi-square tests, as appropriate.

- (Supplementary Information, p. 33)

Supplementary Table 20. ICD-9 and ICD-10 coding algorithms for Charlson Comorbidity Index (CCI) score.

Condition	ICD-9 Codes	ICD-10 Codes	Weight
Acute myocardial infarction	410, 412	I21.x, I22.x, I25.2	1
Congestive heart failure	398.91, 402.01, 402.11, 402.91, 404.01, 404.03, 404.11, 404.13, 404.91, 404.93, 425.4–425.9, 428	I09.9, I11.0, I13.0, I13.2, I25.2, I42.0, I42.5–I42.9, I43.x, I50.x, P29.0	1
Peripheral vascular disease	093.0, 437.3, 443.1–443.9, 447.1, 557.1, 557.9, V43.4, 440, 441	I70.x, I71.x, I73.1, I73.8, I73.9, I77.1, I79.0, I79.2, K55.1, K55.8, K55.9, Z95.8, Z95.9	1
Cerebrovascular disease	430–438, 362.34	G45.x, G46.x, H34.0, I60.x–I69.x	1
Dementia	290, 294.1, 331.2	F00.x–F03.x, F05.1, G30.x, G31.1	1
Chronic pulmonary disease	490–496, 500–505, 506.4, 508.1, 508.8	I27.8, I27.9, J40.x–J47.x, J60.x–J67.x, J68.4, J70.1, J70.3	1
Rheumatic disease	446.5, 710.0–710.4, 714.0–714.2, 714.8, 725	M05.x, M06.x, M31.5, M32.x–M34.x, M35.1, M35.3, M36.0	1
Peptic ulcer disease	531–534	K25.x–K28.x	1
Mild liver disease	070.2–070.5, 070.6, 070.9, 570, 571, 573.3–573.9, V42.7	B18.x, K70.0–K70.3, K70.9, K71.3–K71.5, K71.7, K73.x, K74.x, K76.0, K76.2–K76.4, K76.8, K76.9, Z94.4	1
Moderate/severe liver disease	456.0–456.2, 572.2–572.4, 572.8	I85.0, I85.9, I86.4, I98.2, K70.4, K71.1, K72.1, K72.9, K76.5–K76.7	3
Diabetes without complications	250.0–250.3, 250.8–250.9	E10.0–E10.1, E10.6, E10.8–E10.9, E11.0–E11.1, E11.6, E11.8–E11.9, E12.0–E12.1, E12.6, E12.8–E12.9, E13.0–E13.1, E13.6, E13.8–E13.9, E14.0–E14.1, E14.6, E14.8–E14.9	1
Diabetes with complications	250.4–250.7	E10.2–E10.5, E10.7, E11.2–E11.5, E11.7, E12.2–E12.5, E12.7, E13.2–E13.5, E13.7, E14.2–E14.5, E14.7	2
Hemiplegia or paraplegia	334.1, 342–344	G04.1, G11.4, G80.1–G80.2, G81.x, G82.x, G83.0–G83.4, G83.9	2
Renal disease	403–404, 582–588, V42.0, V45.1, V56	I12.0, I13.1, N03.2–N03.7, N05.2–N05.7, N18.x, N19.x, N25.0, Z49.0–Z49.2, Z94.0, Z99.2	2
Cancer (non-metastatic)	140–195, 200–208	C00.x–C26.x, C30.x–C34.x, C37.x–C41.x, C43.x, C45.x–C58.x, C60.x–C76.x, C81.x–C85.x, C88.x, C90.x–C97.x	2
Metastatic cancer	196–199	C77.x–C80.x	6
AIDS/HIV	042–044	B20.x–B22.x, B24.x	6

- (Supplementary Information, p. 34)

Supplementary Table 21. Medication groups defined using RxNorm ancestor concepts.

Medication Group	RxNorm Ancestor Concept ID	ATC Class	Description
Lipid-lowering agents (statins)	21601855	C10AA	HMG-CoA reductase inhibitors (statins)
Antihypertensive medications	21600381, 21601461, 21601560, 21601664, 21601744, 21601782	C02, C03, C07, C08, C09	Antihypertensive drug classes including diuretics, beta-blockers, calcium channel blockers, and renin–angiotensin system agents
Antidiabetic medications	21600712, 21600713	A10A, A10B	Insulins and blood glucose–lowering drugs (non-insulin)
CNS-active medications	21604652, 21604643, 21604642	N06A, N05C, N05B	Antidepressants, hypnotics/sedatives, and anxiolytics

Reviewer #2

This is a very interesting manuscript that aims to examine the relationship between longitudinal wearable-based activity rhythms and biological age acceleration by leveraging data from the All of Us Research Program. It is well designed, and the data analyses are comprehensive. The results are solid and consistent with previous findings. However, there are still some issues that need to be clarified.

(1) It is unclear when the baseline data were collected and how many years the participants were followed up.

Response 1, Reviewer 2: We appreciate the reviewer's careful evaluation of our manuscript and the insightful feedback provided. Baseline was defined as the start date of the valid accelerometer recording period for each participant. Participants were followed from this baseline until their last available wearable monitoring date, with a mean length of follow-up of 6.84 years (standard deviation: 2.2 years). Valid wear days were defined as ≥10 hours of wear time and ≥100 steps, consistent with prior validation studies. To ensure reliable estimation of activity patterns, we restricted the analytic sample to participants with step data across a full 12-month period within a given calendar year, as stated in Methods.

We have revised our manuscript to clarify the definition of baseline and follow-up in the Methods and to report the summary statistic in Table 1. → (Methods, p. 23; Results, p. 5; Table 1, p. 1)

- (Methods, p. 23) "Baseline was defined as the earliest date of the valid accelerometer recording period for each participant. Participants were followed from baseline until their last available wearable monitoring date."

- (Results, p. 5) "The mean length of follow-up was 6.84 years (SD 2.2)."

- (Table 1, p. 1)

Table 1. Baseline characteristics of the study population by biological aging status.

Mean (SD) or n (%)	All (n=2222)	Fast Aging (n=1021)	Slow Aging (n=1201)	*p*-value
Age, years	60.62 (14.01)	61.09 (13.82)	60.23 (14.17)	0.147
PhenoAge, years	59.90 (15.72)	66.43 (14.57)	54.34 (14.48)	<0.001
Sex				<0.001
Female	1522 (68.5)	636 (62.3)	886 (73.8)	
Male	693 (31.2)	380 (37.2)	313 (26.1)	
Race				0.008
White	1839 (82.8)	838 (82.1)	1001 (83.3)	
Black	134 (6.0)	78 (7.6)	56 (4.7)	
Other	249 (11.2)	105 (10.3)	144 (12.0)	
BMI, kg/m^2	29.84 (6.98)	31.76 (7.48)	28.21 (6.07)	<0.001
Education				0.187
College or above	2084 (93.8)	958 (93.8)	1126 (93.8)	
12 years or GED	119 (5.4)	50 (4.9)	69 (5.7)	
Income				<0.001
High	1015 (45.7)	428 (41.9)	587 (48.9)	
Medium	669 (30.1)	303 (29.7)	366 (30.5)	

Low	395 (17.8)	224 (21.9)	171 (14.2)	
Missing	143 (6.4)	66 (6.5)	77 (6.4)	
Employment				0.011
Employed	1260 (56.7)	564 (55.2)	696 (58.0)	
Retired	732 (32.9)	345 (33.8)	387 (32.2)	
Unemployed	159 (7.2)	88 (8.6)	71 (5.9)	
Other	71 (3.2)	24 (2.4)	47 (3.9)	
Smoking				0.048
Yes	788 (35.5)	394 (38.6)	394 (32.8)	
No	1434 (64.5)	627 (61.4)	807 (67.2)	
Drinking				0.36
Yes	2168 (97.6)	1000 (97.9)	1168 (97.3)	
No	54 (2.4)	21 (2.1)	33 (2.7)	
Comorbidities				
Cancer	207 (9.3)	92 (9.0)	115 (9.6)	0.702
CVD	247 (11.1)	107 (10.5)	140 (11.7)	0.417
Type 2 diabetes	212 (9.5)	97 (9.5)	115 (9.5)	1.000
Daily steps	7715.83 (3429.04)	7215.22 (3374.21)	8141.42 (3419.17)	<0.001
Sleep duration, hours	6.39 (1.67)	6.32 (1.67)	6.46 (1.66)	0.048
No recent healthcare contact	181 (8.1)	84 (8.2)	97 (8.1)	0.959
Limited healthcare access	518 (23.3)	232 (22.7)	286 (23.8)	0.578
Logistic barrier	429 (19.3)	202 (19.8)	227 (18.9)	0.637
Finance barrier	312 (14.0)	147 (14.4)	165 (13.7)	0.701
Length of follow-up, years	6.84 (2.2)	6.77 (2.23)	6.90 (2.15)	0.165
Charlson Comorbidity Index (CCI) score				<0.001
0	841 (37.8)	315 (30.9)	526 (43.8)	
1	463 (20.8)	212 (20.8)	251 (20.9)	
2	343 (15.4)	163 (16.0)	180 (15.0)	
≥3	575 (25.9)	331 (32.4)	244 (20.3)	
Medication use				
Antihypertensive	824 (37.1)	464 (45.4)	360 (30.0)	<0.001
Statin	530 (23.9)	303 (29.7)	227 (18.9)	<0.001
Antidiabetic	251 (11.3)	189 (18.5)	62 (5.2)	<0.001
CNS-active	289 (13.0)	171 (16.7)	118 (9.8)	<0.001

Values are mean (SD) for continuous variables and n (%) for categorical variables. *p*-values are based on *t*-tests or chi-square tests, as appropriate.

(2) The analyses of Year 1, Year 3, and Year 5 are confusing. Were the follow-up visits conducted at Year 3 and Year 5? Were all participants included or only a sub-population? The results from Figure 3 are not matched to those from Table 2. For example, it seems that Acrophase was significantly different between slow and fast aging groups at Year 3 and Year 5 (Figure 3), but this association was not significant during the follow-up shown in Table 2.

Response 2, Reviewer 2: We thank the reviewer for this important question, and we provide two clarifying points to address your question.

First, Year 1, Year 3, and Year 5 represent time since baseline, defined based on the availability of valid wearable data (Year 1=first year with wearable data), rather than fixed follow-up visits. We selected Year 3 and Year 5 to represent intermediate and longer-term follow-up windows that provide sufficient temporal separation to detect changes in both circadian metrics and biological aging.

Second, Figure 3 and Table 2 address distinct but complementary analytical objectives. **Figure 3 presents unadjusted group differences** at selected time points to illustrate temporal patterns. These analyses were conducted using year-specific analytic subsets, restricted to participants with concurrent wearable-based features and biological aging measures at each time point. Figure 3 displays mean values with standard errors for each circadian metric, and p-values were obtained using two-sample t-tests comparing group means.

In contrast, **Table 2 reports results from multivariable regression models** that adjust for demographic, socioeconomic, and clinical covariates, thereby enabling population-level inference. For acrophase, the changes in statistical significance after covariate adjustment suggest that the unadjusted associations are at least partially explained by confounding. In contrast, metrics capturing activity intensity, such as MESOR, amplitude, and M10, demonstrate consistent associations across both baseline and follow-up even after adjustment, indicating that these relationships are robust to measured confounding and reflect independent associations with the outcome.

We have revised the manuscript to clarify (1) the definition of time as years since baseline, (2) the sample composition across year-specific analytic subsets, and (3) the distinction between unadjusted and adjusted analyses. In addition, we have reordered the Results to present Figure 3 prior to Table 2 to improve interpretability and reduce potential confusion. → (Methods, p. 29; Results, p. 8; Fig. 3, p. 5).

- (Methods, p. 29) "<u>To characterize temporal patterns in circadian rhythm features, we summarized unadjusted trajectories at years 1, 3, and 5 using year-specific analytic subsets of participants with concurrent wearable and biological aging data. Mean values (± standard error) were computed for fast and slow agers at each time point, and between-group differences were assessed using two-sample t-tests.</u>"

- (Results, p. 8) "<u>To evaluate temporal patterns in circadian rhythm features in relation to biological aging, we first characterized descriptive trajectories of circadian rhythm features across years 1, 3, and 5 (**Fig. 3**). Overall, fast agers exhibited lower rhythm intensity (MESOR, amplitude, RA, M10), reduced regularity (IS), and delayed timing (M10 start time) compared to slow agers. These differences were generally consistent across time points, although their magnitude varied.</u>

Building on these descriptive patterns, we next assessed the associations of rhythmicity with accelerated biological aging at baseline and 5-year follow-up (**Table 2**)."

- (Fig. 3 caption, p. 5) "**Fig. 3 Trajectories of circadian rhythm metrics over 5 years stratified by biological age acceleration.** Mean values (± standard error) are shown for fast agers (red) and slow agers (blue), classified according to biological age acceleration at each time point. Analyses are based on year-specific analytic subsets, restricted to participants with concurrent availability of wearable-derived features and biological aging measures. Asterisks denote the statistical significance of between-group differences at each time point (*$p<0.05$, **$p<0.01$, ***$p<0.001$)."

(3) Figure 4 is hard to understand. In all situations for all parameters, the ORs for fasting age were above 1, suggesting a significant increased risk, which is not consistent with the primary results.

Response 3, Reviewer 2: To address this concern, we have revised Figure 4 to present model-based predicted probabilities, which provide a more interpretable representation on the absolute risk scale. We estimated the predicted probability of accelerated biological aging at baseline and follow-up across the observed range of each circadian rhythm feature, while holding covariates constant at their mean (for continuous variables) or mode (for categorical variables) values.

This revised figure clarifies both the direction and magnitude of associations. As shown, higher levels of rhythm intensity metrics (e.g., MESOR, amplitude, RA, and M10) are associated with a monotonic decrease in the predicted probability of accelerated aging, whereas delayed timing metrics (e.g., acrophase and M10 start time) show increasing risk gradients. These patterns are not only consistent with the primary results but are now also interpretable as absolute differences in risk. We have revised the manuscript accordingly. → (Results, p. 9; Fig. 4, p. 7).

- (Results, p. 9) "For each circadian feature, we further estimated model-based predicted probabilities of accelerated aging at baseline and follow-up across the observed range of the feature to facilitate interpretation of results (**Fig. 4**)."

- (Fig. 4, p. 7)

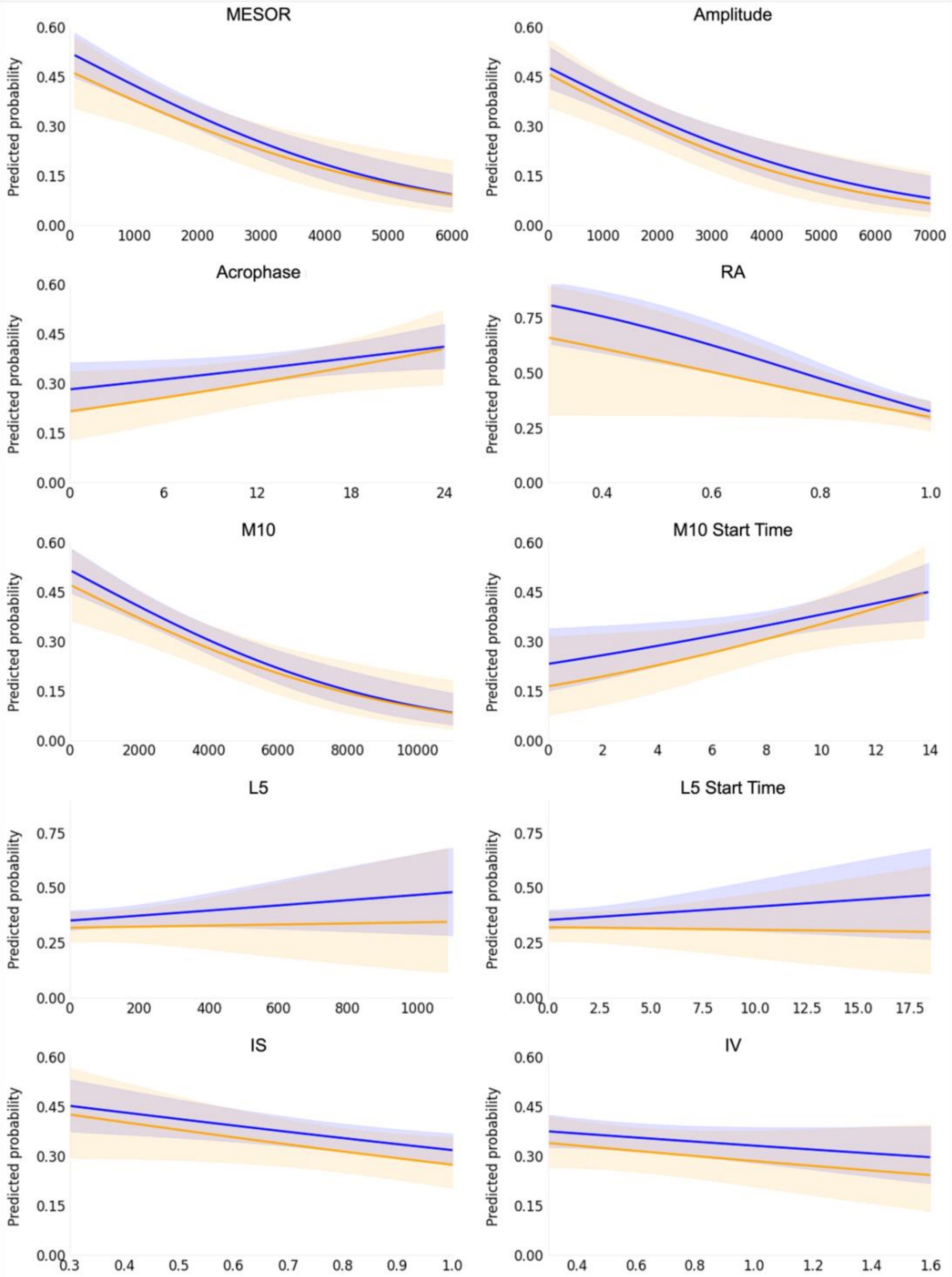

Fig. 4 Predicted probabilities of accelerated biological aging across functional and circadian rhythm features. Each panel displays the model-estimated predicted probability of fast aging across the observed range of a given circadian rhythm metric. Blue and orange lines correspond to baseline and follow-up respectively, with shaded areas representing 95% confidence intervals. Predicted probabilities were derived from multivariable logistic regression models, holding covariates at their mean (continuous variables) or mode (categorical variables) values. All models are adjusted for age, sex, race/ethnicity, education, income, employment, lifetime smoking, current alcohol consumption, and comorbidities (cancer, CVD, type 2 diabetes).

(4) Some results are not consistent between Table 2 and Table 3. For example, the association between IS and BioAge seems significant in cross-sectional data (significant at the baseline and borderline at the follow-up), while it is completely gone in the mixed analysis. This may need to be further clarified.

Response 4, Reviewer 2: These differences are expected and arise from the distinct model structures used in each analysis. **Table 2** presents cross-sectional associations at specific time points (baseline and follow-up), which reflect *between-individual differences* at a given time point. While our results demonstrate robust associations at the between-individual level, our research question further extends to whether within-individual changes in circadian metrics over time are associated with changes in biological aging. To address this, **Table 3** is based on linear mixed-effects models that incorporate repeated measurements and estimate within-individual associations over time while accounting for subject-specific random effects. As a result, a feature may show significant cross-sectional associations but attenuated or non-significant longitudinal effects if it exhibits limited within-individual variability over time, thereby reducing the ability to detect longitudinal associations.

The example of interdaily stability (IS) illustrates this distinction. IS shows a significant association at baseline (OR: 0.889, 95% CI: 0.820-0.964, p=0.004), and a borderline association at follow-up (OR: 0.879, 95% CI: 0.768-1.006, p=0.061), in cross-sectional models (**Table 2**), indicating that individuals with higher IS tends to have lower accelerated aging at a given time point. However, in the mixed-effects model (**Table 3**), the association is attenuated and no longer statistically significant (OR: 0.910, 95% CI: 0.756-1.095, p=0.319), suggesting that within-individual changes in IS over time are modest. A similar pattern is observed for M10 start time and L5 start time, which retain consistent directions and comparable effect sizes but lose statistical significance in longitudinal models. This likely reflects reduced within-individual variability over time, leading to reduced precision in longitudinal estimates.

Critically, these findings are not inconsistent but instead reflect complementary perspectives: cross-sectional analyses capture population-level differences at specific time points, whereas longitudinal analyses using multi-year data enable evaluation of how these relationships evolve within individuals over time.

We have clarified this distinction in the revised manuscript by distinguishing between cross-sectional (between-individual) and longitudinal (within-individual) associations. → (Results, p. 10)

- (Results, p. 10) "The attenuation of statistical significance for certain timing and regularity metrics in longitudinal models, despite consistent cross-sectional associations, highlights the distinction between between-individual and within-individual effects. For some features, limited intra-individual variability over time may reduce the ability to detect longitudinal associations, even when they exhibit strong population-level differences."

(5) Figure 5 is very interesting. Are the associations between ΔCRMetrics and ΔBioAge significant? If so, in my opinion it may provide more novel findings than those revealed from the mixed analysis.

Response 5, Reviewer 2: We thank the reviewer for this insightful comment. To evaluate whether within-person changes in circadian rhythm metrics (ΔCRMetrics) are associated with changes in biological aging (ΔAge), we conducted additional change–change (Δ–Δ) regression analyses (**Supplementary Table 4**).

- (Supplementary Information, p. 17)

Supplementary Table 4. Change-change regression analysis of the association between within-person changes in circadian rhythm metrics and biological aging (ΔAge).

Features	Beta Coefficient (95% CI)	*p*-value
MESOR	**-0.427 (-0.793, -0.060)**	**0.023**
Amplitude	**-0.627 (-0.992, -0.261)**	**0.001**
Acrophase	0.185 (-0.184, 0.554)	0.326
Relative amplitude (RA)	-0.101 (-0.467, 0.266)	0.591
M10	**-0.374 (-0.940, -0.207)**	**0.002**
L5	-0.173 (-0.542, 0.197)	0.360
M10 start time	-0.046 (-0.416, 0.324)	0.807
L5 start time	**0.420 (0.053, 0.786)**	**0.025**
Interdaily stability (IS)	**-0.370 (-0.737, -0.002)**	**0.048**
Intraday variability (IV)	0.272 (-0.094, 0.638)	0.145

Beta coefficients and 95% confidence intervals (CIs) are derived from linear regression models. Models are adjusted for age, sex, race/ethnicity, education, income, employment, lifetime smoking, current alcohol consumption, and comorbidities (cancer, CVD, type 2 diabetes). Significant associations ($p<0.05$) are shown in bold.

We observed statistically significant associations for several key circadian features. In particular, increases in activity intensity metrics, including MESOR, amplitude, and M10, were significantly associated with decreases in ΔAge (all p<0.05), indicating that improvements in overall activity level are linked to slower biological aging within individuals. In addition, greater interdaily stability (IS) was associated with lower ΔAge (p=0.048), suggesting that more stable daily activity patterns are beneficial for healthy aging. Conversely, later nocturnal timing, reflected by L5 start time, was associated with higher ΔAge (p=0.025), indicating that delayed rest-phase timing may contribute to accelerated aging.

Importantly, while our linear mixed-effects models identified consistent longitudinal associations for activity intensity metrics (e.g., MESOR, amplitude, and M10), the Δ–Δ analyses revealed additional associations for circadian stability (IS) and nocturnal timing (L5 start time). This difference arises from the distinct estimands of the two approaches. Mixed-effects models leverage both within- and between-person variation over time and primarily capture average associations across individuals. In contrast, Δ–Δ models isolate within-person changes by differencing, thereby removing all time-invariant between-person heterogeneity and directly capturing the coupling between changes in exposure and outcome.

As a result, features that demonstrate robust associations in both models (e.g., activity intensity) likely reflect stable and biologically meaningful relationships with biological aging that operate at both the between- and within-person levels. In contrast, features with greater intra-individual variability over time, such as circadian timing and rhythm stability, are slightly differently captured by Δ–Δ and linear mixed effect models.

Together, these findings demonstrate that the two approaches are complementary: mixed-effects models establish robust longitudinal associations, while Δ–Δ analyses provide

direct evidence that changes in circadian rhythm characteristics track changes in biological aging within individuals. This convergence of evidence strengthens the overall interpretation that circadian organization, particularly activity intensity, stability, and timing, is dynamically linked to the progression of biological aging.

We have clarified these analyses in the revised manuscript and provided additional interpretation in the Discussion. Relevant revisions have been incorporated in the Methods, Discussion, and Supplementary Information. → (Methods, p. 30; Results, p. 10; Discussion, p. 15; Supplementary Table 4).

- (Methods, p. 30) "We further assessed whether within-person changes in circadian rhythm metrics are associated with concurrent changes in biological aging, we conducted change–change (Δ–Δ) regression analyses. For each participant, changes in circadian metrics and ΔAge between baseline and follow-up were calculated, and linear regression models were fitted with ΔAge as the outcome and the change in circadian rhythm metrics as predictors. Models were adjusted for the same set of covariates as in the primary analyses."

- (Results, p. 10) "To further isolate within-person associations, we conducted additional change–change (Δ–Δ) regression analyses (**Supplementary Table 4**). Consistent with the mixed-effects models, increases in activity intensity metrics, including MESOR, amplitude, and M10, were significantly associated with decreases in ΔAge (all $p<0.05$), indicating that improvements in activity levels are linked to slower biological aging within individuals. In addition, greater interdaily stability (IS) was associated with lower ΔAge, while later nocturnal timing, reflected by L5 start time, was associated with higher ΔAge."

- (Discussion, p. 15) "Consistent with this variability, differences between modeling approaches provide additional context for interpreting these features. Activity intensity metrics (MESOR, amplitude, and M10) showed consistent associations with biological aging across both linear mixed-effects models and change–change (Δ–Δ) analyses. In contrast, timing and stability features exhibited model-specific patterns, with relative amplitude (RA) and daytime timing (M10 start time) identified in mixed-effects models, and rhythm stability (IS) and nocturnal timing (L5 start time) emerging in Δ–Δ analyses. This divergence is consistent with their greater sensitivity to model specification and suggests that these features may reflect more context-dependent and within-person processes."

(6) Since these are longitudinal data, it would be interesting to know whether the baseline CRMetrics can predict BioAge at the follow-up.

Response 6, Reviewer 2: Thank you for the insightful suggestion. In response, we performed additional analyses to evaluate whether baseline circadian rhythm metrics predict biological aging at follow-up. As shown in **Supplementary Table 10**, several baseline circadian rhythm features were significantly associated with PhenoAge at follow-up. In particular, higher levels of activity rhythm intensity metrics (e.g., fPC1, MESOR, amplitude, RA, and M10) were significantly associated with lower PhenoAge at follow-up. For instance, each unit increase in fPC1 score and MESOR was associated with 0.98-year (95% CI: -1.052 to -0.449) and 1.06-year (95% CI: -1.580 to -0.535) lower PhenoAge at follow-up,

respectively. In contrast, later activity timing metrics (e.g., M10 start time) were associated with higher PhenoAge at follow-up at follow-up (0.65 years, 95% CI: 0.144-1.154).

- (Supplementary Information, p. 23)

Supplementary Table 10. Baseline functional and circadian rhythm metrics as predictors of PhenoAge at follow-up.

Features	Beta Coefficient (95% CI)	*p*-value
fPC1	**-0.976 (-1.502, -0.449)**	**<0.001**
fPC2	-0.085 (-0.566, 0.397)	0.730
fPC3	-0.358 (-0.828, 0.113)	0.136
fPC4	-0.098 (-0.568, 0.372)	0.681
MESOR	**-1.057 (-1.580, -0.535)**	**<0.001**
Amplitude	**-0.931 (-1.446, -0.415)**	**<0.001**
Acrophase	0.272 (-0.226, 0.770)	0.284
Relative amplitude (RA)	**-0.593 (-1.098, -0.088)**	**0.021**
M10	**-1.198 (-1.720, -0.676)**	**<0.001**
L5	0.130 (-0.365, 0.625)	0.607
M10 start time	**0.649 (0.144, 1.154)**	**0.012**
L5 start time	0.368 (-0.121, 0.857)	0.140
Interdaily stability (IS)	-0.479 (-0.976, 0.017)	0.058
Intraday variability (IV)	**-0.512 (-1.008, -0.015)**	**0.043**

Beta coefficients and 95% confidence intervals (CIs) are derived from linear regression models. Models are adjusted for age, sex, race/ethnicity, education, income, employment, lifetime smoking, current alcohol consumption, and comorbidities (cancer, CVD, type 2 diabetes). Significant associations (p<0.05) are shown in bold.

These findings are consistent with our primary analyses using the binary fast aging outcome, supporting the robustness of the observed relationships across different outcome definitions and highlighting the predictive relevance of circadian rest-activity rhythm organization for biological aging. We have clarified these analyses in the manuscript and included the corresponding results in the Supplementary Information. → (Methods, p. 31; Results, p. 12; Discussion, p. 13; Supplementary Table 10).

- (Methods, p. 31) "Eighth, we examined baseline wearable-derived features as predictors of PhenoAge at follow-up."

- (Results, p. 12) "Key CRAR metrics, including intensity, regularity, and timing, remained significantly associated with accelerated aging and PhenoAge at baseline and during follow-up (**Supplementary Table 10**)."

- (Discussion, p. 13) "Rhythm intensity, regularity, and timing emerged as important predictors of accelerated aging and PhenoAge at baseline and during follow-up."

(7) The analysis of interaction with follow-up years is confusing (Suppl Table 3). What is the purpose of this analysis? How to interpret the results? There is significant interaction between fPC1 and year (OR=1.218) on BioAge. What does this OR mean?

Response 7, Reviewer 2: We thank the reviewer for requesting this important clarification. We included the interaction between fPC1 and follow-up time to assess whether the association between circadian rhythm (captured by fPC1) and biological aging is time-dependent. Given that aging is a dynamic and progressive process, this analysis allows us to evaluate whether circadian disruption has a progressively stronger or weaker impact on biological aging as individuals are followed longitudinally.

The estimated OR of 1.218 indicates that, for each additional year of follow-up, the odds ratio relating fPC1 to accelerated PhenoAge increases by 21.8%. Importantly, this does not represent a direct increase in PhenoAge risk per year. Rather, it indicates that the magnitude of the association between fPC1 and biological age becomes progressively stronger over time. In other words, individuals with unfavorable circadian profiles (as reflected by fPC1) exhibit increasing divergence in biological aging trajectory as follow-up time increases.

We have revised the manuscript to clarify the purpose and interpretation of this interaction term in both the Methods and Results sections. → (Methods, p. 30; Results, p. 11)

- (Methods, p. 30) "Second, we tested for time-by-metric interaction effects in the linear mixed effect models to assess whether the associations between behavioral metrics and biological aging varied <u>change over the follow-up period, thereby assessing potential time-dependent effects</u>."

- (Results, p. 11) "Together, the longitudinal analyses extend the cross-sectional findings by showing that within-individual changes in circadian rhythmicity dynamically shape the pace of biological aging. <u>Specifically, fPC1 (reflecting overall rhythm intensity and robustness) exhibited a significant time-dependent effect (OR=1.218, 95% CI: 1.073-1.382), indicating that its association with biological aging strengthens over follow-up. In contrast, other rhythmicity metrics, including timing and regularity measures, did not show significant time-dependent effects. Consistent with cross-sectional results, intensity- and robustness-related features emerged as the most consistent predictors, while timing and regularity showed more sex-specific or weaker longitudinal effects.</u>"

Reviewer #3
Thank you for the interesting submission! Below I will try to concisely articulate the central claims of the work, followed by comments / questions. Of course please correct any errors in my interpretation.

Claims made
* The central claim in this work is that rhythmic features of "activity" (here step count measurements obtained via a wearable) are associated with pace of aging. Crucially, "aging" is assessed using a PhenoAge constructed through EHR records, with some amount of imputation. Rhythmic features examined include standard features and nonparametric fPCA latent variables.
* Rhythm intensity is protective — here higher amplitude of MESOR, amplitude, M10, and fPC1 are consistently associated with reduced likelihood of accelerated PhenoAge. This finding holds across cross-sectional and longitudinal analyses.
* Timing is a Risk Factor: Delayed circadian timing, particularly a later acrophase (timing of peak activity) and later onset of the least active period (L5 start time), is associated with an increased likelihood of accelerated PhenoAge. Greater day-to-day regularity is associated with slower biological aging, however may be sex-specific.

* Some associations appear to be sex-dependent. The effects of timing and regularity are claimed to be more pronounced in females. In contrast, males with accelerated aging are uniquely characterized by a "biphasic instability" phenotype (early-morning and late-evening activity peaks, captured by fPC4).

Response 1, Reviewer 3: We thank the reviewer for the careful evaluation of our manuscript and for the insightful comments. Below, we present a detailed, point-by-point response.

Comments & Questions
* "... as a digital biomarker of aging risk" (line 86) — "aging risk" strikes me as an odd construction. One might be more precise and say "digital biomarker of accelerated aging risk," or better yet, "digital biomarker of accelerated PhenoAge". For clarity, it might be appropriate to distinguish between "accelerated PhenoAge," the measured variable, and "accelerated aging," the biological process it is intended to measure.

Response 2, Reviewer 3: We appreciate the reviewer's valuable suggestion. In response, we clearly distinguish between (1) accelerated PhenoAge, the measured variable derived from clinical biomarkers, and (2) accelerated biological aging, the broader biological construct that PhenoAge is intended to approximate. Accordingly, we have replaced "digital biomarker of aging risk" with "digital biomarker of accelerated biological age" where appropriate and clarified this distinction in the Introduction and Discussion sections. → (Introduction, p. 4; Discussion, p. 20)

- (Introduction, p. 4) "By linking these longitudinal activity rhythm profiles with repeated measures of biological age, quantified <u>using the clinical biomarker-based</u> phenotypic age (PhenoAge)[1], we <u>aim to capture the dynamic progression of biological aging. PhenoAge is calculated based on a mortality prediction score derived from nine clinical biomarkers (albumin, alkaline phosphatase, creatinine, C-reactive protein (CRP), glucose, mean cell volume, red cell distribution width, white blood cell count, and lymphocyte percentage) and chronological age, reflecting the age at which the average NHANES III mortality risk matches the predicted mortality risk[1]. It has been</u>

validated across multi-ethnic cohorts to predict disease, physical functioning, cognitive performance, and mortality, and whose longitudinal changes provide prognostic information beyond baseline levels[1,3,7–10]. Our findings provide among the first large-scale longitudinal evidence that rest-activity rhythm shapes trajectories of biological aging and support its role as a scalable and accessible biomarker, laying the groundwork for precision prevention strategies targeting circadian health to promote healthy longevity."

- (Discussion, p. 20) "At the population level, interventions that enhance rhythm intensity, such as structured physical activity programs, may broadly reduce accelerated biological age."

* Within subject change in PhenoAge is a major end point in this study. What is the evidence in the literature longitudinal PhenoAge is a reliable surrogate of accelerated "biological aging"? It would bolster the motivation to point to evidence that longitudinal, accelerated PhenoAge independently correlates with increased disease or mortality risk, above just cross sectional PhenoAge measurements.

Response 3, Reviewer 3: PhenoAge is a clinically derived biomarker of biological aging that has been extensively validated across diverse populations. It predicts morbidity, functional decline, and mortality independent of chronological age, including major outcomes such as cardiovascular disease, cancer, and diabetes[1,3,9,10], even among initially healthy individuals[15]. In addition, while PhenoAge is correlated with epigenetic aging measures, it captures complementary physiological dimensions of aging, reflecting multi-system dysregulation[16,17].

Critically, emerging longitudinal evidence supports that within-individual changes in biological age provide prognostic information beyond baseline levels. In the Baltimore Longitudinal Study of Aging, Kuo et al. demonstrated that longitudinal changes in phenotypic aging metrics are associated with functional decline and adverse health outcomes[8]. Similarly, longitudinal analyses across multiple biological aging measures have shown that increases in biological age over time are associated with subsequent mortality risk beyond baseline measurements[7].

These findings support a key conceptual distinction: while cross-sectional PhenoAge reflects accumulated biological aging, longitudinal changes may capture the rate of aging, which is more proximal to ongoing physiological decline. Consistent with this framework, increases in PhenoAge over time can be interpreted as accelerated biological aging and serve as a valuable measure to estimate incremental prognostic value beyond baseline measurements. These prior works support both the validity of PhenoAge as a biomarker of biological aging and the interpretation of its longitudinal change as a dynamic and independently informative marker of aging trajectories.

To clarify this rationale, we have revised the Introduction and Methods to highlight the longitudinal validity of biological age measures. → (Introduction, p. 4; Methods, p. 24).

- (Introduction, p. 4) "By linking these longitudinal activity rhythm profiles with repeated measures of biological age, quantified using the clinical biomarker-based phenotypic age (PhenoAge)[1], we aim to capture the dynamic progression of biological aging. PhenoAge is calculated based on a mortality prediction score derived from nine clinical biomarkers (albumin, alkaline phosphatase, creatinine, C-reactive protein

(CRP), glucose, mean cell volume, red cell distribution width, white blood cell count, and lymphocyte percentage) and chronological age, reflecting the age at which the average NHANES III mortality risk matches the predicted mortality risk[1]. It has been validated across multi-ethnic cohorts to predict disease, physical functioning, cognitive performance, and mortality, and whose longitudinal changes provide prognostic information beyond baseline levels[1,3,7–10]. Our findings provide among the first large-scale longitudinal evidence that rest-activity rhythm shapes trajectories of biological aging and support its role as a scalable and accessible biomarker, laying the groundwork for precision prevention strategies targeting circadian health to promote healthy longevity."

- (Methods, p. 24) "The primary outcome was clinical biomarker-based phenotypic age (PhenoAge), a validated biomarker-based estimate of biological age that predicts morbidity, functional decline, and all-cause mortality[2]. Beyond cross-sectional associations, longitudinal changes in phenotypic age have been shown to be associated with disease and mortality risk, supporting its use as a dynamic marker of aging trajectories[8]. PhenoAge has also been associated with wearable-derived rhythmicity in prior cross-sectional analyses[11,12]. It is derived from nine clinical biomarkers, including albumin, alkaline phosphatase, creatinine, C-reactive protein (CRP), glucose, mean cell volume, red cell distribution width, white blood cell count, and lymphocyte percentage, along with chronological age[2]. These biomarkers were selected using a Cox proportional hazards elastic net model for mortality prediction in the National Health and Nutrition Examination Survey III (NHANES III) cohort[2]. PhenoAge estimation is based on the parametrization of two Gompertz proportional hazards models: one incorporating the selected biomarkers and chronological age to estimate an individual-level mortality risk, and the other using chronological age alone as a baseline. PhenoAge is then derived by equating the predicted mortality risks from both models, reflecting the age at which an individual's mortality risk is equivalent of the refence population."

* Regarding fast vs slow agers, "... cardiovascular disease, and diabetes did not differ significantly between the two groups" — is that odd? Would you not expect faster agers to also have more diabetes and CVD diagnoses?

Response 4, Reviewer 3: We thank the reviewer for this insightful observation. While it may seem intuitive that faster agers would have a higher burden of cardiometabolic diseases, the absence of cross-sectional differences is not unexpected. We provide four complementary explanations.

1. **Comorbidity burden and treatment intensity (Charlson Comorbidity Index (CCI) and medication use)**: To further characterize baseline health status, we evaluated comorbidity burden using the Charlson Comorbidity Index (CCI) and major medication use. CCI was derived from ICD-9/10 codes using established algorithms, and medication use was defined based on prescriptions for key cardiometabolic and CNS-active drug classes. We observed significant differences in CCI distribution between groups, driven primarily by higher proportions of individuals with no comorbidity among slow agers and high comorbidity burden among fast agers. Medication use was consistently more prevalent among fast agers across all classes.

Our results indicate that while the prevalence of specific diseases did not differ significantly between groups, overall comorbidity burden, as measured by the Charlson Comorbidity Index (CCI), was significantly higher among fast agers (**Table 1**). This suggests that differences are driven not by the presence of individual diagnoses, but by the accumulation and distribution of multiple conditions. Consistent with this interpretation, fast agers also exhibited substantially higher medication use across cardiometabolic and CNS-active drug classes, supporting a higher overall treatment burden and underlying morbidity.

- (Table 1, p. 1)

Table 1. Baseline characteristics of the study population by biological aging status.

Mean (SD) or n (%)	All (n=2222)	Fast Aging (n=1021)	Slow Aging (n=1201)	*p*-value
Age, years	60.62 (14.01)	61.09 (13.82)	60.23 (14.17)	0.147
PhenoAge, years	59.90 (15.72)	66.43 (14.57)	54.34 (14.48)	<0.001
Sex				<0.001
Female	1522 (68.5)	636 (62.3)	886 (73.8)	
Male	693 (31.2)	380 (37.2)	313 (26.1)	
Race				0.008
White	1839 (82.8)	838 (82.1)	1001 (83.3)	
Black	134 (6.0)	78 (7.6)	56 (4.7)	
Other	249 (11.2)	105 (10.3)	144 (12.0)	
BMI, kg/m^2	29.84 (6.98)	31.76 (7.48)	28.21 (6.07)	<0.001
Education				0.187
College or above	2084 (93.8)	958 (93.8)	1126 (93.8)	
12 years or GED	119 (5.4)	50 (4.9)	69 (5.7)	
Income				<0.001
High	1015 (45.7)	428 (41.9)	587 (48.9)	
Medium	669 (30.1)	303 (29.7)	366 (30.5)	
Low	395 (17.8)	224 (21.9)	171 (14.2)	
Missing	143 (6.4)	66 (6.5)	77 (6.4)	
Employment				0.011
Employed	1260 (56.7)	564 (55.2)	696 (58.0)	
Retired	732 (32.9)	345 (33.8)	387 (32.2)	
Unemployed	159 (7.2)	88 (8.6)	71 (5.9)	
Other	71 (3.2)	24 (2.4)	47 (3.9)	
Smoking				0.048
Yes	788 (35.5)	394 (38.6)	394 (32.8)	
No	1434 (64.5)	627 (61.4)	807 (67.2)	
Drinking				0.36
Yes	2168 (97.6)	1000 (97.9)	1168 (97.3)	
No	54 (2.4)	21 (2.1)	33 (2.7)	
Comorbidities				
Cancer	207 (9.3)	92 (9.0)	115 (9.6)	0.702
CVD	247 (11.1)	107 (10.5)	140 (11.7)	0.417
Type 2 diabetes	212 (9.5)	97 (9.5)	115 (9.5)	1.000

Daily steps	7715.83 (3429.04)	7215.22 (3374.21)	8141.42 (3419.17)	<0.001
Sleep duration, hours	6.39 (1.67)	6.32 (1.67)	6.46 (1.66)	0.048
No recent healthcare contact	181 (8.1)	84 (8.2)	97 (8.1)	0.959
Limited healthcare access	518 (23.3)	232 (22.7)	286 (23.8)	0.578
Logistic barrier	429 (19.3)	202 (19.8)	227 (18.9)	0.637
Finance barrier	312 (14.0)	147 (14.4)	165 (13.7)	0.701
Length of follow-up, years	6.84 (2.2)	6.77 (2.23)	6.90 (2.15)	0.165
Charlson Comorbidity Index (CCI) score				<0.001
0	841 (37.8)	315 (30.9)	526 (43.8)	
1	463 (20.8)	212 (20.8)	251 (20.9)	
2	343 (15.4)	163 (16.0)	180 (15.0)	
≥3	575 (25.9)	331 (32.4)	244 (20.3)	
Medication use				
Antihypertensive	824 (37.1)	464 (45.4)	360 (30.0)	<0.001
Statin	530 (23.9)	303 (29.7)	227 (18.9)	<0.001
Antidiabetic	251 (11.3)	189 (18.5)	62 (5.2)	<0.001
CNS-active	289 (13.0)	171 (16.7)	118 (9.8)	<0.001

Values are mean (SD) for continuous variables and n (%) for categorical variables. *p*-values are based on *t*-tests or chi-square tests, as appropriate.

2. **Conceptual distinction between biological aging and disease burden:** PhenoAge acceleration (ΔAge) captures physiological aging relative to chronological age and reflects early, subclinical biological processes rather than accumulated and clinically recognized disease burden. As such, ΔAge is conceptually distinct from baseline comorbidity prevalence, and differences in ΔAge may not necessarily manifest as differences in diagnosed disease at a single time point.

3. **Consistency with prior literature:** The observed pattern is consistent with prior literature. In large population-based cohorts such as the UK Biobank, biological age measures, including PhenoAge, have shown weaker or inconsistent associations with *prevalent* disease, while demonstrating stronger and more consistent associations with incident outcomes and mortality[17]. In contrast, stronger cross-sectional associations are typically observed in higher-risk or disease-enriched populations[18].

4. **Healthy cohort effect:** The All of Us Research Program cohort, particularly the subset with both wearable and electronic health record data, is relatively healthy compared to the general population, reflecting volunteer participation and technology adoption patterns. Prior work has shown that wearable users in the All of Us cohort are more likely to be younger, female, and non-Hispanic White, indicating a selection toward healthier and more health-conscious individuals[19,20]. Such characteristics may reduce variability in disease prevalence and attenuate cross-sectional differences between groups. This

interpretation is further supported by prior analyses within the All of Us cohort, which have reported substantially lower prevalence of coronary artery disease among wearable users compared to the excluded individuals[21].

5. **Robustness of disease definition:** To assess whether this finding was driven by disease definitions, we conducted sensitivity analyses using more restrictive criteria. Specifically, we limited cardiovascular disease to ischemic heart disease, heart failure, and stroke, and restricted cancer to malignant neoplasms only. The results remained unchanged, with no significant differences in baseline comorbidity prevalence between fast and slow agers (**Table R2**), indicating that this finding is not driven by disease definition.

Table R2. Comparison of baseline comorbidities between fast and slow agers using restricted definitions of cancer and cardiovascular disease.

Mean (SD) or n (%)	All (n=2222)	Fast Aging (n=1021)	Slow Aging (n=1201)	*p*-value
Cancer (malignant only)	53 (2.4)	23 (2.3)	30 (2.5)	0.812
CVD (ischemic heart disease, heart failure, stroke only)	76 (3.4)	33 (3.2)	43 (3.6)	0.739

In summary, the absence of differences in baseline comorbidities between fast and slow agers is not unexpected. Rather, it suggests that ΔAge may capture early, preclinical biological aging processes that is yet be reflected in diagnosed disease at baseline but are more strongly associated with future disease risk and progression. We have revised the manuscript to clarify this point in the Methods, Result, and Discussion sections. → (Methods, p. 29; Results, p. 5; Discussion, p. 18)

- (Methods, p. 29) "In addition, we quantified overall comorbidity burden using the Charlson Comorbidity Index (CCI) using ICD codes[13] (**Supplementary Table 20**). CCI was computed as the weighted sum of predefined comorbid conditions and categorized as 0, 1, 2, and ≥3. We further assessed medication use as a proxy for treatment burden and underlying health status. We focused on major medication classes relevant to cardiometabolic and neurocognitive aging based on recent research on medication and biological aging[14], including antihypertensive medications, lipid-lowering agents (statins), antidiabetic medications, and central nervous system (CNS)-active medications (antidepressants and sedatives) (**Supplementary Table 21**)."

- (Results, p. 5) "Education, current alcohol consumption, and comorbidities of cancer, cardiovascular disease (CVD), and diabetes were comparable between groups. However, comorbidity burden, as measured by the Charlson Comorbidity Index (CCI), differed significantly between groups (p<0.001). Individuals with no comorbidity (CCI=0) were more prevalent among slow agers (43.8% vs. 30.9%), whereas a high comorbidity burden (CCI≥3) was more common among fast agers (32.4% vs. 20.3%), indicating that differences were primarily driven by the extremes of comorbidity burden. Similarly, medication use differed significantly by biological aging status (all p<0.001). Fast agers had a higher prevalence of antihypertensive (45.4% vs. 30.0%), statin (29.7% vs. 18.9%), antidiabetic (18.5% vs. 5.2%), and

CNS-active medication use (16.7% vs. 9.8%) compared to slow agers. These patterns suggest a higher treatment burden and greater cardiometabolic and neurocognitive morbidity among individuals with accelerated biological aging."

- (Discussion, p. 18) "We did not observe significant differences in the baseline prevalence of major comorbidities, including cancer, cardiovascular disease, and diabetes, between fast and slow agers. However, overall comorbidity burden, as measured by the Charlson Comorbidity Index (CCI), and medication use differed substantially between groups, with fast agers exhibiting higher multimorbidity and greater treatment burden. This may suggest that accelerated biological aging is more strongly reflected in the accumulation and severity of conditions rather than the presence of individual diagnoses at a single time point.

  This pattern further supports that biological age measures may capture underlying physiological processes that may precede clinically manifest disease and therefore may not be fully reflected in cross-sectional disease prevalence. Prior evidence similarly shows that biological aging metrics are more strongly associated with incident disease and mortality than with prevalent conditions[17].

  In addition, the *All of Us* cohort, particularly the subset with wearable data, represents a relatively healthy population, with a higher proportion of younger, non-Hispanic White females. Such characteristics may reduce variability in baseline disease prevalence and attenuate cross-sectional differences between groups. Accordingly, differences in biological aging may not necessarily manifest as differences in diagnosed conditions at baseline, particularly in relatively healthy population-based cohorts."

* "slow agers ... exhibited higher MESOR" (line 124) — first mention of MESOR, would appreciate a definition/description of this statistic.

Response 5, Reviewer 3: At its first mention, we define MESOR (Midline Estimating Statistic Of Rhythm) as the rhythm-adjusted mean level of activity across the 24-hour cycle estimated from the cosinor model. → (Results, p. 6)

- (Results, p. 6) "Across age ranges, slow agers steadily exhibited higher MESOR (Midline Estimating Statistic Of Rhythm, a rhythm-adjusted mean level of activity across the 24-hour cycle estimated from the cosinor model), cosinor amplitude, RA (relative amplitude), M10 (mean activity during the 10 most active hours), and IS (interdaily stability; day-to-day rhythm regularity) compared with fast agers."

* "latent nonlinear variation in 24-h profiles" (line 134) — on initial read it was unclear that these refer to a representation of the daily step-count data, could be clarifying to be explicit that these are 24-h step-count profiles.

Response 6, Reviewer 3: We have revised the text to clarify that the "latent nonlinear variation" refers specifically to variability in the 24-hour step-count profiles derived from wearable devices. This clarification has been added at first mention in the Results section. → (Results, p. 7)

- (Results, p. 7) "Functional principal component analysis (fPCA) was performed on the 24-hour smartwatch-based step-count trajectories to identify dominant modes of variation in daily activity rhythms (**Fig. 2**)."

* The fPCA analysis is super interesting, and the fPCs seem to clearly identify general behavioral patterns wrt activity. These bases are, however, not informed by aging or pace of aging — would an analyses like partial least squares (or a functional variant) yield bases that reveal variability specific to accelerated aging?

Response 7, Reviewer 3: We appreciate the reviewer's insightful comment. Our objective was to identify interpretable, population-level behavioral phenotypes that characterize circadian rest-activity rhythm structure, rather than to derive features optimized for predicting accelerated aging. In this context, functional principal component analysis (fPCA) and partial least squares (PLS) address distinct statistical objectives: fPCA captures dominant modes of variation in the functional predictor, whereas PLS maximizes covariance with the outcome and is therefore outcome-dependent. To assess this directly, we conducted additional analyses using PLS, selecting four components via 10-fold cross-validated Receiver Operating Characteristic-Area Under the Curve (ROC-AUC).

1. **Correlation between fPC and PLS components:** We found that the PLS components were strongly correlated with combinations of fPCs rather than representing entirely new axes (**Supplementary Fig. 9**). For instance, PLS1 was nearly identical to fPC1 (r=0.96), indicating that the dominant source of aging-related variation is already captured by the primary mode of variation in the fPC space. Similarly, PLS2 showed a strong correlation with fPC3 (r= −0.84), suggesting that activity timing contributes substantially to aging-related differences. fPC3 and fPC4 further reflected combinations of fPCs rather than novel axes. PLS3 was primarily associated with a mixture of fPC2 and fPC4 (r=0.53 and r= −0.65, respectively). PLS4 exhibited weaker and more distributed correlations across fPCs, suggesting it captures residual variation not dominated by a single interpretable behavioral dimension.

   This indicates that PLS did not identify additional behavioral dimensions beyond those captured by fPCA. Instead, it reweighted existing modes of variation to maximize association with accelerated aging, indicating that the dominant aging-relevant signal is already represented within the primary fPCA components.

   - (Supplementary Information, p. 12)

   Supplementary Fig. 9 Correlation between functional principal components (fPCs) and partial least squares (PLS) components.

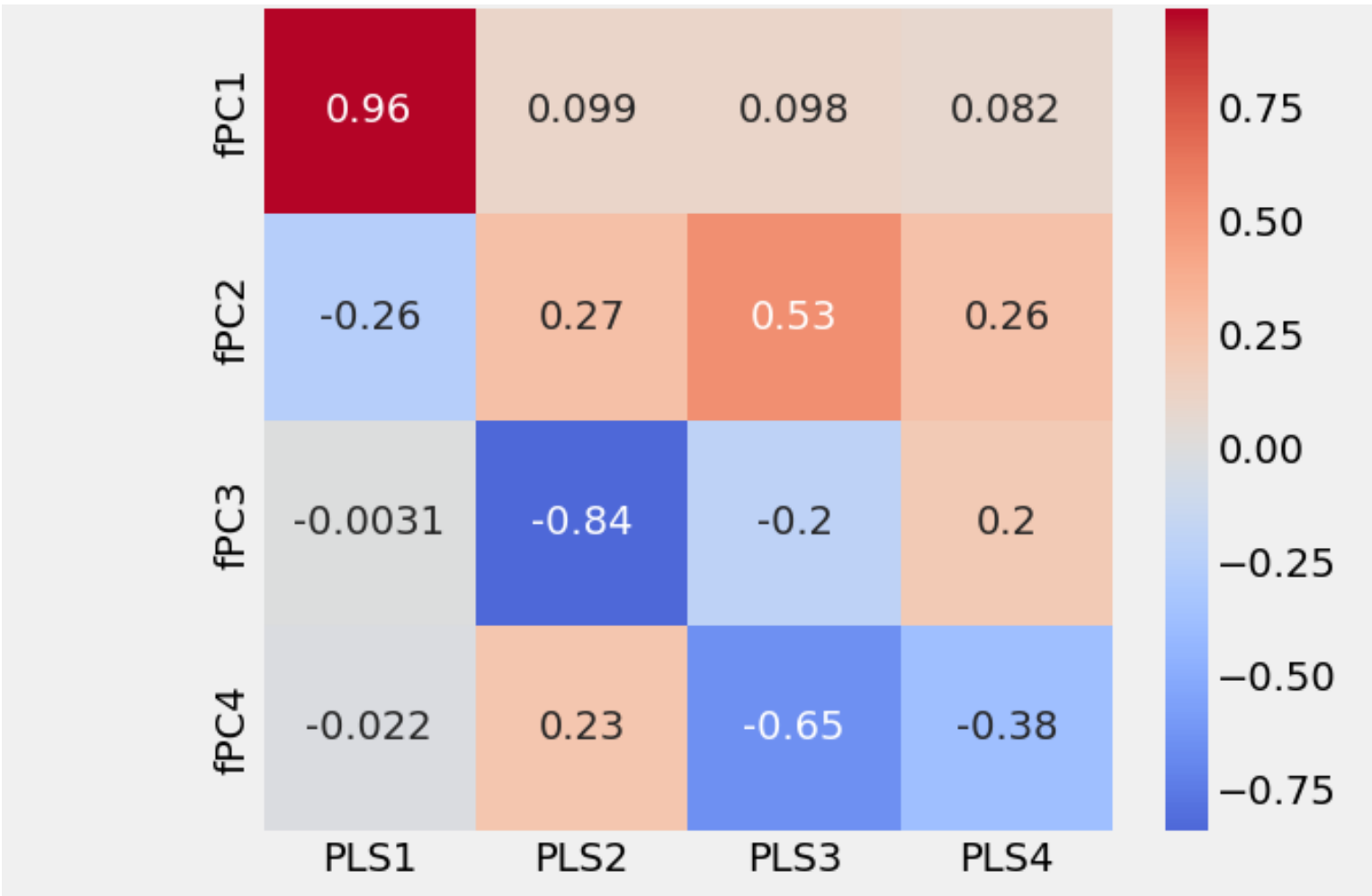

2. **Comparison of associations of fPCA- and PLS-derived components:** In addition, **Supplementary Table 17** supports this interpretation. While PLS components were statistically associated with accelerated aging, their effect sizes were generally modest and reflected reparameterizations of the same underlying variation captured by fPCs. For example, the strongest protective association was observed for fPC1 (OR=0.81, 95% CI: 0.75-0.88), whereas the corresponding PLS1 showed a much smaller effect size (OR=0.99, 95% CI: 0.98-0.99). Similarly, PLS2 and PLS3 aligned with fPC3- and fPC2/4-related variation, respectively, without suggesting introducing qualitatively distinct patterns. Although PLS4 showed a larger effect size (OR=1.16, 95% CI: 1.13-1.19), its weaker and more diffuse correlation with fPCs suggests that it captures residual variation that is less directly interpretable in terms of circadian behavioral structure.

   - (Supplementary Information, p. 30)

   Supplementary Table 17. Associations of fPCA- and PLS-derived components with accelerated biological aging at baseline.

fPC components	OR (95% CI)	PLS components	OR (95% CI)
fPC1	0.810 (0.745, 0.878)	PLS1	0.985 (0.980, 0.990)
fPC2	1.044 (0.965, 1.130)	PLS2	1.037 (1.024, 1.051)
fPC3	0.956 (0.884, 1.035)	PLS3	0.945 (0.929, 0.961)
fPC4	1.003 (0.927, 1.086)	PLS4	1.161 (1.130, 1.193)

All models are adjusted for age, sex, race/ethnicity, education, income, employment, lifetime smoking, current alcohol consumption, and comorbidities (cancer, cardiovascular disease, and type 2 diabetes). Partial least squares (PLS) components were selected based on 10-fold cross-validated receiver operating characteristic area under the curve (ROC-AUC).

In summary, these findings indicate that PLS seems to redistribute existing fPCA-derived variation to optimize association with the outcome. In contrast, fPCA provides a more interpretable and outcome-agnostic representation of the underlying behavioral structure. Importantly, fPCA yields the functional principal components are orthogonal in function space (i.e., their inner products over time are zero). In contrast, in partial least squares (PLS), only the latent X-scores are forced to be mutually orthogonal, while the associated loading/weight vectors and Y-side components are not generally orthogonal. As a result, PLS does not provide an orthogonal functional basis in the same way fPCA does. This further supports the use of fPCA as the primary analytical framework in our study. We have added these findings to the manuscript. → (Methods, p. 31; Results, p. 12; Discussion, p. 18; Supplementary Fig. 9 & Table 17)

- (Methods, p. 31) "To complement the fPCA-based analysis, we conducted additional analyses using partial least squares (PLS), a supervised dimensionality reduction approach. PLS components were defined to maximize covariance between the predictor matrix and the outcome. The number of PLS components was selected as four based on 10-fold cross-validated receiver operating characteristic–area under the curve (ROC-AUC). For each fold, models were trained on the training set and evaluated on the held-out set, and the number of components corresponding to the optimal cross-validated performance was retained. To compare the latent structures identified by fPCA and PLS, we quantified the pairwise Pearson correlations. We

then evaluated associations of both fPCA- and PLS-derived components with accelerated biological aging consistent with the primary analysis framework."

- (Results, p. 12) "In addition, supervised approaches (e.g., partial least squares (PLS)) yielded components closely aligned with the primary fPCA modes and did not identify new behavioral dimensions (**Supplementary Fig. 9; Supplementary Table 17**), indicating that the dominant aging-related variation is largely captured by the fPCA-derived structure. For instance, PLS1 was nearly identical to fPC1 (r=0.96), indicating that the dominant source of aging-related variation is already captured by the primary mode of variation in the fPC space. Similarly, PLS2 showed a strong correlation with fPC3 (r=−0.84), suggesting that activity timing contributes substantially to aging-related differences. fPC3 and fPC4 further reflected combinations of fPCs rather than novel axes. PLS3 was primarily associated with a mixture of fPC2 and fPC4 (r=0.53 and r=−0.65, respectively). PLS4 exhibited weaker and more distributed correlations across fPCs, suggesting it captures residual variation not dominated by a single interpretable behavioral dimension. This indicates that PLS did not identify additional behavioral dimensions beyond those captured by fPCA. Instead, it reweighted existing modes of variation to maximize association with accelerated aging, indicating that the dominant aging-relevant signal is already represented within the primary fPCA components."

- (Discussion, p. 18) "Additional analyses using supervised approaches (e.g., partial least squares (PLS)) yielded components closely aligned with the primary fPCA modes and did not identify substantively new behavioral dimensions, suggesting that the dominant aging-related variation is already captured within the fPCA framework. These findings further indicate that PLS primarily redistributes existing fPCA-derived variation to optimize association with the outcome, rather than identifying independent functional modes. In contrast, fPCA provides a more interpretable and outcome-agnostic representation of the underlying behavioral structure. Importantly, fPCA yields an orthogonal decomposition of the functional trajectories, with components that are mutually orthogonal in function space (i.e., their inner products over time are zero). By comparison, PLS enforces orthogonality only among the latent X-scores, while the associated loading (weight) vectors and Y-side components are not generally orthogonal, and thus does not provide an orthogonal functional basis in the same way as fPCA. Together, these properties support the use of fPCA as the primary analytical framework in our study."

* "Sex-stratified analyses further refined these insights ..." (line 282) — How confident can we be that the sex-specific differences reveal genuine variability between the sexes as opposed to some statistical artifact?

Response 8, Reviewer 3: To formally evaluate whether the observed sex-specific patterns reflect true effect modification rather than statistical artifacts, we tested sex-by-circadian phenotype interaction terms within baseline, follow-up, and longitudinal models (**Supplementary Table 3**).

Several circadian features demonstrated statistically significant interactions with sex, particularly for amplitude, relative amplitude (RA), L5, and M10 start time. Importantly, these interactions were not isolated findings but showed consistent direction and magnitude across models. For example, amplitude and RA exhibited significant interactions at baseline

and in longitudinal analyses (Amplitude: OR=1.21, p=0.035; longitudinal OR=1.43, p=0.011; RA: OR=1.30, p=0.003; longitudinal OR=1.53, p=0.001), indicating robust sex-dependent effects. Similarly, L5 and M10 start time showed consistent negative interactions across baseline and longitudinal models, further supporting reproducibility over time.

In contrast, interaction effects for fPC components were weaker and did not reach statistical significance, suggesting that sex-specific differences are more strongly captured by specific circadian dimensions, such as intensity and timing, rather than by global functional modes of variation.

- (Supplementary Information, p. 16)

Supplementary Table 3. Sex interaction effects for circadian and functional features on accelerated biological aging.

	Baseline		Follow-up		Longitudinal	
Features	OR (95% CI)	*p*-value	OR (95% CI)	*p*-value	OR (95% CI)	*p*-value
fPC1*sex	1.049 (0.881, 1.249)	0.592	1.316 (0.884, 1.958)	0.176	1.044 (0.837, 1.303)	0.701
fPC2*sex	0.886 (0.751, 1.046)	0.153	0.968 (0.657, 1.427)	0.871	0.865 (0.697, 1.073)	0.186
fPC3*sex	1.114 (0.946, 1.313)	0.196	1.181 (0.812, 1.719)	0.384	1.214 (0.980, 1.504)	0.076
fPC4*sex	1.122 (0.951, 1.325)	0.172	1.223 (0.828, 1.805)	0.312	1.218 (0.983, 1.510)	0.072
MESOR*sex	1.055 (0.885, 1.259)	0.550	0.983 (0.735, 1.315)	0.908	1.115 (0.846, 1.470)	0.440
Amplitude*sex	**1.207 (1.013, 1.438)**	**0.035**	1.106 (0.825, 1.481)	0.501	**1.427 (1.084, 1.879)**	**0.011**
Acrophase*sex	0.978 (0.827, 1.156)	0.793	**0.705 (0.530, 0.937)**	**0.016**	0.924 (0.736, 1.161)	0.497
RA*sex	**1.298 (1.092, 1.543)**	**0.003**	1.309 (0.986, 1.738)	0.063	**1.531 (1.178, 1.988)**	**0.001**
M10*sex	1.115 (0.935, 1.330)	0.226	1.017 (0.757, 1.365)	0.913	1.235 (0.938, 1.626)	0.133
L5*sex	**0.827 (0.697, 0.982)**	**0.030**	**0.741 (0.559, 0.984)**	**0.038**	**0.697 (0.537, 0.905)**	**0.007**
M10 start*sex	**0.806 (0.681, 0.953)**	**0.012**	0.917 (0.693, 1.214)	0.547	**0.714 (0.548, 0.930)**	**0.013**
L5 start*sex	**0.816 (0.690, 0.964)**	**0.017**	1.045 (0.792, 1.379)	0.754	0.872 (0.679, 1.120)	0.283
IS*sex	1.162 (0.978, 1.380)	0.087	1.249 (0.940, 1.660)	0.126	1.203 (0.949, 1.526)	0.127
IV*sex	0.926 (0.782, 1.097)	0.375	0.859 (0.650, 1.134)	0.283	0.878 (0.681, 1.133)	0.318

All models are adjusted for age, sex, race/ethnicity, education, income, employment, lifetime smoking, current alcohol consumption, and comorbidities (cancer, CVD, type 2 diabetes). Longitudinal model includes a random intercept. Significant associations ($p<0.05$) are shown in bold.

As noted, interaction terms were used to assess whether associations differed between males and females. Because interaction tests evaluate differences in effect sizes between groups, they generally have lower statistical power than stratified analyses. In addition, the smaller sample size of males in our cohort (31.2%, n=693) compared to females (68.5%, n=1,522) may further limit power to detect interaction effects for some features. Accordingly, null interaction findings should be interpreted with caution.

Our findings are also consistent prior literature indicating sex-specific differences in circadian regulation and aging-related vulnerability. For example, a prospective cohort study of 820 older adults with wrist-worn actigraphy reported that age-related declines in circadian

amplitude and rhythm regularity may be more pronounced in women, particularly for measures of activity intensity and stability[22].

In conclusion, these results provide converging evidence that the observed sex-specific associations are unlikely to be solely explained by statistical artifacts and instead reflect underlying biological heterogeneity. We have clarified these points in the Methods, Results, Discussion and included additional robustness analyses in the Supplementary Information. → (Methods, p. 30; Results, p. 9; Supplementary Table 3)

- (Methods, p. 30) "Third, we evaluated effect modification by sex by including sex-by-metric interaction terms in regression models across baseline, follow-up, and longitudinal analyses."

- (Results, p. 9) "While several associations were statistically significant in sex-stratified analyses, not all corresponding interaction terms reached statistical significance (**Supplementary Table 3**), indicating that between-sex differences in effect size were modest for some features."

* In fact, elsewhere in the manuscript (line 128, line 501) sex-stratified analyses are viewed as a robustness or sensitivity check — finding similar effects bolsters the finding of the full-data model. Here, seeing timing and regularity related associations appearing stronger in women, while weaker in men. What sensitivity checks or robustness would give us faith in this differential result?

Response 9, Reviewer 3: To assess the robustness of the observed sex-specific differences, we conducted complementary analyses examining within-sex consistency, effect modification testing, and reproducibility.

First, sex-stratified analyses demonstrated that key associations were present within both males and females, although effect sizes differed (**Supplementary Tables 1-2**), indicating that the observed relationships are not driven by a single subgroup.

Second, as detailed in **Response 8**, Reviewer 3, we formally tested effect modification using sex-by-circadian phenotype interaction terms across baseline, follow-up, and longitudinal models (**Supplementary Table 3**), which provided evidence of consistent interaction effects for several features, including amplitude, relative amplitude (RA), L5, and M10 start time.

Third, interaction effects for fPCA-derived components were not statistically significant, suggesting that sex-specific differences are more strongly captured by specific circadian dimensions (e.g., intensity and timing) rather than global functional modes of variation.

Finally, we note that interaction tests generally have lower statistical power than stratified analyses, and the smaller sample size in males (31.2%, n=693) may further limit detection of interaction effects. Accordingly, null interaction findings should be interpreted with caution.

In summary, these analyses provide converging evidence supporting the robustness of the observed sex-specific differences.

* "circadian rhythmicity exerts a lasting influence on biological aging trajectories" (line 347-348) — This strikes me as a pretty squarely causal claim. I would temper this kind of claim, as the evidence from the analysis certainly doesn't definitely show that step-count circadian rhythms cause accelerated or decelerated aging (based on PhenoAge).

Response 10, Reviewer 3: We have revised the relevant statements to use associative language in the Discussion section. → (Discussion, p. 22)

- (Discussion, p. 22) "Building on this foundation, this study provides longitudinal evidence that wearable-derived circadian rhythmicity metrics is associated with biological aging trajectories. By leveraging both conventional CRAR metrics and functional data analysis, we show that rhythm intensity is consistently associated with slower PhenoAge acceleration, while timing and fragmentation show sex-specific associations."

* "When up to two biomarkers were missing, values were imputed using the cohort mean" (line 401-402) — Why not impute missing PhenoAge variables with more sophisticated approach like MICE? Is there a reason to think that these values are missing not at random, or at least if missing at random other observables can explain away the missingness patterns? What effect would this have on the conclusions of the analyses?

Response 11, Reviewer 3: We thank the reviewer for this valuable comment regarding the handling of missing biomarker data. We addressed this through additional analyses focusing on the missingness mechanism, sensitivity to imputation strategy, and result robustness.

1. **Missingness mechanism:** To evaluate the plausibility of a missing-at-random assumption, we examined whether missingness in biomarker data was systematically associated with observed covariates. We modeled missingness as a function of demographic, socioeconomic, and clinical variables. We did not find strong evidence that missingness was structured by these measured covariates.

2. **Sensitivity to imputation strategy:** We performed extensive sensitivity analyses to assess the impact of the imputation strategy. Specifically, we implemented multiple imputation by chained equations (MICE) and derived PhenoAge using imputed biomarker values. The resulting PhenoAge estimates were highly correlated with those obtained using mean imputation across the overall sample and by sex (r=0.916-0.943; **Supplementary Fig. 8**), indicating that the overall ranking and distribution of biological aging measures were largely preserved across imputation approaches.

    - (Supplementary Information, p. 11)
      Supplementary Fig. 8 Correlation of PhenoAge estimates derived from mean imputation and multiple imputation (MICE). Pearson correlation coefficients (r).

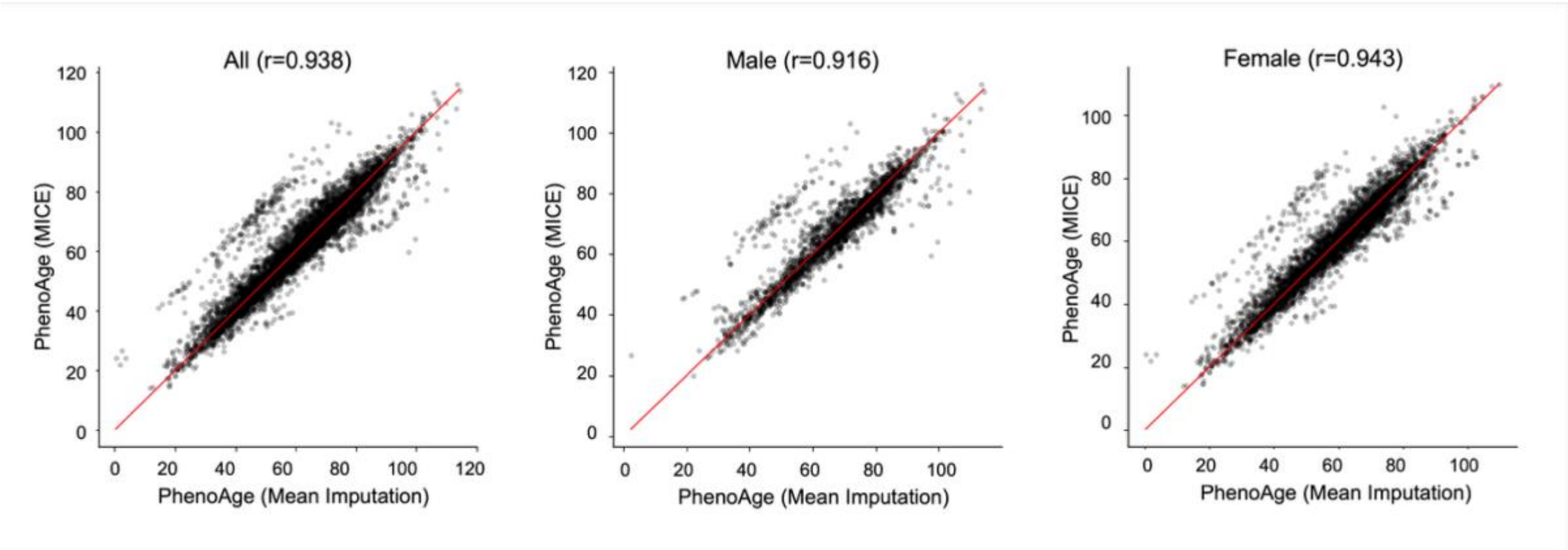

3. **Result robustness:** We finally compared the longitudinal associations between circadian rhythm features and biological aging across imputation methods (**Supplementary Table 6**). Effect estimates were highly consistent in both direction and magnitude. Key findings, including the protective associations of rhythm robustness (e.g., amplitude, relative amplitude, M10) and the adverse associations of nocturnal activity (e.g., L5), remained stable across methods. While minor differences in statistical significance were observed for some timing-related variables (e.g., acrophase, M10 start, L5 start), these differences were modest and did not alter the overall interpretation.

   - (Supplementary Information, p. 19)

   Supplementary Table 6. Comparison of longitudinal associations between circadian rhythm metrics and biological aging across imputation methods.

Features	Mean Imputation OR (95% CI)	*p*-value	MICE OR (95% CI)	*p*-value
fPC1	**0.832 (0.757, 0.915)**	**<0.001**	**0.818 (0.737, 0.884)**	**<0.001**
fPC2	1.059 (0.966, 1.162)	0.219	**1.108 (1.026, 1.195)**	**0.008**
fPC3	0.938 (0.857, 1.028)	0.172	0.962 (0.892, 1.036)	0.306
fPC4	1.016 (0.928, 1.113)	0.726	1.061 (0.984, 1.144)	0.124
MESOR	**0.654 (0.574, 0.744)**	**<0.001**	**0.649 (0.597 ,0.707)**	**<0.001**
Amplitude	**0.623 (0.553, 0.702)**	**<0.001**	**0.637 (0.586, 0.693)**	**<0.001**
Acrophase	**1.150 (1.036, 1.277)**	**0.009**	1.034 (0.953, 1.122)	0.423
RA	**0.764 (0.682, 0.855)**	**<0.001**	**0.709 (0.649, 0.775)**	**<0.001**
M10	**0.612 (0.536, 0.698)**	**<0.001**	**0.632 (0.581, 0.688)**	**<0.001**
L5	1.055 (0.945, 1.178)	0.343	**1.138 (1.043, 1.242)**	**0.004**
M10 start	**1.133 (1.004, 1.278)**	**0.043**	1.061 (0.971, 1.159)	0.188
L5 start	1.052 (0.943, 1.173)	0.363	**1.090 (1.000, 1.188)**	**0.049**
IS	**0.895 (0.809, 0.991**	**0.033**	**0.843 (0.780, 0.910)**	**<0.001**
IV	0.891 (0.792, 1.002)	0.054	0.954 (0.881, 1.034)	0.468

All models are adjusted for age, sex, race/ethnicity, education, income, employment, lifetime smoking, current alcohol consumption, and comorbidities (cancer, CVD, type 2 diabetes). Longitudinal model includes a random intercept. Significant associations (p<0.05) are shown in bold.

Together, these analyses demonstrate that our findings are robust to the choice of imputation method and are unlikely to be driven by the imputation strategy. This has been clarified in the revised manuscript, and the corresponding sensitivity analyses are provided in the Supplementary Information. → (Methods, p. 25; Results, p. 11; Discussion, p. 14; Supplementary Fig. 8 & Table 6)

- (Methods, p. 25) “When up to two biomarkers were missing, values were imputed using the cohort mean; for CRP, which exhibited >50% missing values, the global mean was substituted. This imputation has been shown to exert minimal influence on downstream analyses. <u>To evaluate the robustness of this approach, we conducted</u>

sensitivity analyses using alternative imputation strategies, including multiple imputation by chained equations (MICE). PhenoAge estimates were recalculated using imputed biomarker values, and agreement between estimates derived from mean imputation and MICE was assessed. In addition, we evaluated whether associations between circadian rhythm metrics and biological aging were consistent across imputation methods."

- (Results, p. 11) "Our findings were also robust to the handling of missing biomarker data. Using multivariate imputation by chained equations (MICE), the resulting PhenoAge estimates were strongly correlated with those obtained using mean imputation across the overall sample and by sex (r=0.916-0.943). Furthermore, longitudinal associations between functional and circadian rhythm features and biological aging remained consistent in both magnitude and direction across imputation approaches (**Supplementary Fig. 8; Supplementary Table 6**)."

- (Discussion, p. 14) "Our findings were robust to the handling of missing biomarker data. Sensitivity analyses using multivariate imputation by chained equations imputation (MICE) yielded highly consistent results, with strong agreement in PhenoAge estimates and stable associations between circadian rhythm metrics and biological aging across imputation approaches. These results reduce the likelihood that our findings are driven by the choice of imputation method."

* Further, what kind of selection effects could influence your analysis by relying on the EHR for reconstructing the outcome? What kind of selection effects could result from relying on longitudinal labs that go into the PhenoAge calculation?

Response 12, Reviewer 3: We thank the reviewer for this important point regarding potential selection effects arising from reliance on EHR-derived laboratory data to reconstruct outcomes and compute PhenoAge.

Individuals who are more engaged with the healthcare system are more likely to have repeated laboratory measurements captured in the EHR, which increases the likelihood of both (1) being included in the analytic sample and (2) having more complete estimation of longitudinal biomarkers such as PhenoAge. Conversely, individuals with limited healthcare access or lower engagement may have fewer recorded laboratory measurements, leading to underrepresentation and potential bias in both exposure and outcome ascertainment.

To address this concern, we conducted several targeted analyses designed to evaluate distinct sources of potential selection bias.

1. **Comparison of included vs. excluded participants:** First, we compared baseline characteristics between individuals included in the longitudinal analysis (i.e., those with wearable data and repeated laboratory measurements) and those excluded (i.e., those with wearable data but without repeated laboratory measurements). In this comparison, we further characterized healthcare access and engagement using the All of Us Healthcare Utilization and Access survey.

   We constructed four domains: (1) no recent healthcare contact within the past 12 months, capturing lack of recent interaction with the healthcare system; (2) limited healthcare access, defined by absence of a usual source of care or prolonged gaps in care; (3) sociological and logistical barriers, including challenges such as transportation difficulties, inability to take time off work, or caregiving responsibilities; and (4) financial

barriers, including cost-related obstacles such as inability to afford copayments, deductibles, or out-of-pocket expenses.

As shown in **Supplementary Table 15**, inclusion in the analytic sample was associated with healthcare engagement. Excluded individuals were younger, had lower income, lower comorbidities, and higher prevalence of limited healthcare access and barriers, indicating systematically reduced clinical capture (all p<0.05).

- (Supplementary Information, p. 28)

Supplementary Table 15. Baseline characteristics of the study population by inclusion and exclusion status.

Mean (SD) or n (%)	Included (n=2222)	Excluded (n=2249)	*p*-value
Age, years	60.62 (14.01)	55.67 (15.09)	<0.001
PhenoAge, years	59.90 (15.72)	55.07 (17.08)	<0.001
Sex			0.107
Female	1522 (68.5)	1604 (71.3)	
Male	693 (31.2)	634 (28.2)	
Race			<0.001
White	1839 (82.8)	1763 (78.4)	
Black	134 (6.0)	130 (5.8)	
Other	249 (11.2)	356 (15.8)	
BMI, kg/m^2	29.84 (6.98)	30.18 (8.04)	0.139
Education			0.237
College or above	2084 (93.8)	2100 (93.4)	
12 years or GED	119 (5.4)	122 (5.4)	
Income			<0.001
High	1015 (45.7)	894 (39.8)	
Medium	669 (30.1)	683 (30.4)	
Low	395 (17.8)	522 (23.2)	
Missing	143 (6.4)	150 (6.7)	
Employment			<0.001
Employed	1260 (56.7)	1403 (62.4)	
Retired	732 (32.9)	534 (23.7)	
Unemployed	159 (7.2)	207 (9.2)	
Other	71 (3.2)	105 (4.7)	
Smoking			0.228
Yes	788 (35.5)	758 (33.7)	
No	1434 (64.5)	1496 (66.3)	
Drinking			0.033
Yes	2168 (97.6)	2169 (96.4)	
No	54 (2.4)	80 (3.6)	
Comorbidities			
Cancer	207 (9.3)	155 (6.9)	0.007
CVD	247 (11.1)	185 (8.2)	0.001
Type 2 diabetes	212 (9.5)	33 (1.5)	<0.001
Daily steps	7715.83 (3429.04)	6566.24 (3175.47)	<0.001
Sleep duration, hours	6.39 (1.67)	6.24 (1.65)	0.013

No recent healthcare contact	181 (8.1)	274 (12.2)	<0.001
Limited healthcare access	518 (23.3)	706 (31.4)	<0.001
Logistic barrier	429 (19.3)	555 (24.7)	<0.001
Finance barrier	312 (14.0)	446 (19.8)	<0.001
Length of follow-up, years	6.84 (2.2)	5.15 (2.5)	<0.001

Values are mean (SD) for continuous variables and n (%) for categorical variables. *p*-values are based on *t*-tests or chi-square tests, as appropriate.

2. **Adjusting for healthcare utilization and access:** Second, to account for these observed differences, we additionally adjusted for healthcare utilization and access measures in statistical analyses, including visit frequency, usual source of care, and structural, logistical, and financial barriers to care (**Supplementary Table 16, Model 2**). Effect estimates remained consistent after this adjustment, suggesting that differential healthcare engagement does not materially explain the observed associations.

3. **Adjusting for the length of follow-up:** Finally, we further adjusted for the length of follow-up as individuals with longer monitoring periods are more likely to have repeated laboratory measurements. Because length of follow-up may be related to the probability of observing both exposures and outcomes in EHR data, this adjustment provides an additional control for differential ascertainment. The results remained robust after this adjustment (**Supplementary 16, Model 3**).

- (Supplementary Information, p. 29)

Supplementary Table 16. Sensitivity analyses adjusting for healthcare engagement and the length of follow-up.

Features	Model 1 OR (95% CI)	*p*-value	Model 2 OR (95% CI)	*p*-value	Model 3 OR (95% CI)	*p*-value
fPC1	**0.832 (0.757, 0.915)**	**<0.001**	**0.834 (0.752, 0.925)**	**0.001**	**0.823 (0.747, 0.905)**	**<0.001**
fPC2	1.059 (0.966, 1.162)	0.219	1.053 (0.953, 1.164)	0.309	1.049 (0.956, 1.151)	0.309
fPC3	0.938 (0.857, 1.028)	0.172	0.940 (0.852, 1.038)	0.220	0.932 (0.851, 1.022)	0.135
fPC4	1.016 (0.928, 1.113)	0.726	1.030 (0.933, 1.137)	0.554	1.010 (0.921, 1.107)	0.837
MESOR	**0.654 (0.574, 0.744)**	**<0.001**	**0.652 (0.580, 0.732)**	**<0.001**	**0.647 (0.570, 0.736)**	**<0.001**
Amplitude	**0.623 (0.553, 0.702)**	**<0.001**	**0.621 (0.547, 0.705)**	**<0.001**	**0.666 (0.595, 0.747)**	**<0.001**
Acrophase	**1.150 (1.036, 1.277)**	**0.009**	**1.156 (1.045, 1.278)**	**0.005**	**1.169 (1.051, 1.301)**	**0.004**
RA	**0.764 (0.682, 0.855)**	**<0.001**	**0.738 (0.659, 0.825)**	**<0.001**	**0.725 (0.649, 0.811)**	**<0.001**
M10	**0.612 (0.536, 0.698)**	**<0.001**	**0.618 (0.545, 0.702)**	**<0.001**	**0.636 (0.565, 0.716)**	**<0.001**
L5	1.055 (0.945, 1.178)	0.343	1.055 (0.943, 1.181)	0.346	1.072 (0.960, 1.198)	0.216
M10 start	**1.133 (1.004, 1.278)**	**0.043**	1.112 (0.993, 1.244)	0.065	1.116 (0.996, 1.250)	0.059
L5 start	1.052 (0.943, 1.173)	0.363	1.067 (0.948, 1.201)	0.283	1.065 (0.949. 1.195)	0.285
IS	**0.895 (0.809, 0.991**	**0.033**	**0.894 (0.808, 0.989)**	**0.030**	**0.891 (0.800, 0.992)**	**0.036**
IV	0.891 (0.792, 1.002)	0.054	0.905 (0.811, 1.010)	0.073	**0.891 (0.800, 0.992)**	**0.035**

Odds ratios (ORs) and 95% confidence intervals (CIs) are derived from linear mixed-effects regression models. Model 1 include a random intercept and adjust for age, sex, race/ethnicity, education, income,

employment, lifetime smoking, current alcohol consumption, and comorbidities (cancer, CVD, type 2 diabetes). Model 2 additionally adjust for healthcare utilization and access (no recent healthcare contact, limited healthcare access, logistic barrier, and financial barrier). Model 3 additionally adjust for the length of follow-up. Significant associations (p<0.05) are shown in bold.

In summary, these analyses indicate that selection into the analytic sample can be related to healthcare engagement and access; however, the consistency of findings after adjustment for healthcare utilization, access to care, and follow-up duration suggests that the observed associations are unlikely to be materially driven by selection bias. We have clarified these points in the Methods, Discussion, and Supplementary Information. → (Methods, p. 30; Discussion, p. 19; Supplementary Tables 15-16).

- (Methods, p. 30) "<u>Fourth, to assess potential selection related to healthcare engagement, we compared baseline characteristics between individuals included in the longitudinal analysis (i.e., those with wearable data and repeated laboratory measurements) and those excluded (i.e., those with wearable data but without repeated laboratory measurements). Healthcare access and utilization were further characterized using the *All of Us* Healthcare Utilization and Access survey, including domains of recent healthcare contact, access to care, logistical barriers, and financial barriers. Fifth, we additionally adjusted for healthcare utilization and follow-up duration in statistical models to account for differential healthcare engagement and observation time.</u>"

- (Discussion, p. 19) "<u>We also evaluated, in sensitivity analyses, whether selection related to healthcare engagement may influence inclusion in the analytic sample and the completeness of laboratory-based measurements used to derive PhenoAge. Individuals with higher healthcare utilization are more likely to have repeated laboratory data captured in EHRs, whereas those with limited access may be underrepresented. In our data, inclusion was associated with healthcare engagement, as excluded individuals were younger, had lower income, fewer comorbidities, and a higher prevalence of barriers to care. Adjustment for healthcare utilization, access to care, and the length of follow-up did not materially change the observed associations. Because behavioral phenotypes were derived from wearable data independently of healthcare utilization, whereas selection primarily affects the availability and completeness of laboratory-based outcomes, this form of selection may preferentially influence measurement completeness rather than the underlying associations. Accordingly, while selection related to healthcare engagement may affect sample representativeness, it is less likely to fully account for the observed relationships.</u>"

* Would this imputation strategy result — more or less constant imputation for missing values — push longitudinal delta PhenoAges toward zero?

Response 13, Reviewer 3: Mean imputation is expected to reduce variability by replacing missing observations with the mean. If such shrinkage were substantial at the individual level, it could attenuate effect estimates toward the null, particularly in longitudinal analyses. However, this pattern was not observed in our results. As detailed in previous **Response 11** to Reviewer 3, associations between circadian rhythm features and biological aging remained stable in both magnitude and direction across imputation methods, including for

longitudinal models. Moreover, key features such as amplitude, relative amplitude, and M10 consistently demonstrated strong associations, indicating that meaningful longitudinal variation was preserved. These findings suggest that the imputation strategy did not significantly attenuate longitudinal changes in PhenoAge.

* “Participants with delta Age > 0 were classified as fast aging, and those with delta Age <= 0 as slow aging” (line 407-408) — Why dichotomize delta Age in your analyses? Could intrinsic variability in the lab create noise around delta Age = 0? Would grouping variables in to tertiles or quantiles demonstrate a stronger effect in the extremes? Why not analyze delta Age as it relates to activity rhythmicity and amplitude directly?

Response 14, Reviewer 3: We thank the reviewer for this important comment. Our primary motivation for dichotomizing ΔAge was to provide an interpretable classification of accelerated versus slower biological aging, consistent with prior literature and facilitating clinical interpretability. To address this concern that dichotomization may obscure variability, particularly near the threshold of zero, we conducted a series of sensitivity analyses.

1. **Modeling continuous ΔAge as an outcome:** We modeled ΔAge as a continuous outcome (**Supplementary Table 7**). Across baseline, follow-up, and longitudinal analyses, we observed consistent and robust associations between circadian activity patterns and biological aging. In particular, metrics reflecting rhythm intensity, timing, and stability demonstrated strong and consistent associations with ΔAge. These findings confirm that the observed relationships are not dependent on dichotomization.

    - (Supplementary Information, p. 20)

Supplementary Table 7. Sensitivity analyses of associations between functional and circadian rhythm features and continuous ΔAge.

	Baseline		Follow-up		Longitudinal	
**Features**	**Beta (95% CI)**	***p*-value**	**Beta (95% CI)**	***p*-value**	**Beta (95% CI)**	***p*-value**
fPC1	**-0.925 (-1.220, -0.629)**	**<0.001**	0.459 (-0.175, 1.093)	0.156	**-0.251 (-0.404, -0.097)**	**0.001**
fPC2	-0.245 (-0.041, 0.531)	0.093	0.380 (-0.245, 1.005)	0.233	0.085 (0.234, 0.076)	0.262
fPC3	-0.101 (-0.385, 0.183)	0.486	0.102 (-0.504, 0.708)	0.741	-0.051 (-0.197, 0.095)	0.493
fPC4	0.001 (-0.286, 0.288)	0.994	0.504 (-0.103, 1.110)	0.104	0.019 (-0.126, 0.164)	0.799
MESOR	**-1.050 (-1.346, -0.754)**	**<0.001**	**-1.258 (-1.760, -0.756)**	**<0.001**	**-0.756 (-0.947, -0.564)**	**<0.001**
Amplitude	**-1.039 (-1.333, -0.746)**	**<0.001**	**-1.241 (-1.743, -0.738)**	**<0.001**	**-0.786 (-0.973, -0.599)**	**<0.001**
Acrophase	**0.437 (0.146, 0.728)**	**0.003**	**0.502 (0.008, 0.997)**	**0.047**	0.096 (-0.046, 0.238)	0.187
RA	**-0.791 (-1.087, -0.496)**	**<0.001**	-0.410 (-0.927, 0.107)	0.120	**-0.401 (-0.577, -0.225)**	**<0.001**
M10	**-1.165 (-1.459, -0.872)**	**<0.001**	**-1.316 (-1.816, -0.817)**	**<0.001**	**-0.871 (-1.063, -0.679)**	**<0.001**
L5	0.186 (-0.109, 0.481)	0.567	-0.219 (-0.724, 0.286)	0.394	-0.063 (-0.237, 0.111)	0.478
M10 start	**0.303 (0.006, 0.600)**	**0.046**	0.419 (-0.078, 0.915)	0.098	**0.192 (0.010, 0.374)**	**0.039**
L5 start	**0.454 (0.160, 0.748)**	**0.002**	0.179 (-0.316, 0.673)	0.478	0.117 (-0.052, 0.285)	0.175
IS	**-0.468 (-0.762, -0.173)**	**0.002**	**-0.721 (-1.221, -0.221)**	**0.005**	**-0.270 (-0.416, -0.123)**	**<0.001**
IV	-0.155 (-0.448, 0.138)	0.300	-0.203 (-0.701, 0.295)	0.812	-0.020 (-0.186, 0.145)	0.809

Beta coefficients and 95% confidence intervals (CIs) are derived from linear regression models. All models are adjusted for age, sex, race/ethnicity, education, income, employment, lifetime smoking, current alcohol consumption, and comorbidities (cancer, CVD, type 2 diabetes). Longitudinal model includes a random intercept. Significant associations (*p*<0.05) are shown in bold.

2. **Quartile groups of key circadian features as predictors:** We further evaluated associations across quartile groups of key circadian features (**Supplementary Tables 8-9**) to assess whether effects were more pronounced at the extremes. We observed clear dose-response patterns, with higher quartiles of amplitude and regularity associated with progressively lower ΔAge, while delayed acrophase was associated with higher ΔAge. These results indicate that the associations are not driven by threshold effects around ΔAge=0 but rather reflect graded relationships across the full distribution.

   - (Supplementary Information, p. 21)

Supplementary Table 8. Sensitivity analyses of associations between wearable feature quartiles and continuous ΔAge.

			Baseline			
**Quartile Groups**	**Amplitude Beta (95% CI)**	***p*-value**	**Acrophase Beta (95% CI)**	***p*-value**	**IS Beta (95% CI)**	***p*-value**
Q1	Reference		Reference		Reference	
Q2	**-1.940 (-2.852, -1.029)**	**<0.001**	0.447 (-0.479, 1.372)	0.344	-0.182 (-1.098, 0.735)	0.698
Q3	**-3.088 (-4.006, -2.169)**	**<0.001**	**1.037 (0.110, 1.963)**	**0.028**	**-0.936 (-1.866, -0.006)**	**0.049**
Q4	**-3.106 (-4.030, -2.183)**	**<0.001**	**1.233 (0.312, 2.155)**	**0.009**	**-1.322 (-2.252, -0.391)**	**0.005**
			**Follow up**			
**Quartile Groups**	**Amplitude OR (95% CI)**	***p*-value**	**Acrophase OR (95% CI)**	***p*-value**	**IS OR (95% CI)**	***p*-value**
Q1	Reference		Reference		Reference	
Q2	**-3.008 (-4.552, -1.464)**	**<0.001**	0.314 (-1.224, 1.852)	0.689	**-1.782 (-3.314, -0.250)**	**0.023**
Q3	**-2.822 (-4.390, -1.254)**	**<0.001**	**2.030 (0.478, 3.583)**	**0.010**	**-1.752 (-3.317, -0.186)**	**0.028**
Q4	**-4.245 (-5.823, -2.667)**	**<0.001**	1.114 (-0.443, 2.670)	0.160	**-2.390 (-3.965, -0.814)**	**0.003**
			**Longitudinal**			
**Quartile Groups**	**Amplitude OR (95% CI)**	***p*-value**	**Acrophase OR (95% CI)**	***p*-value**	**IS OR (95% CI)**	***p*-value**
Q1	Reference		Reference		Reference	
Q2	**-1.428 (-1.898, -0.958)**	**<0.001**	0.028 (-0.428, 0.483)	0.905	**-0.697 (-1.098, -0.296)**	**0.001**
Q3	**-1.995 (-2.511, -1.480)**	**<0.001**	0.156 (-0.313, 0.625)	0.515	**-0.641 (-1.061, -0.222)**	**0.003**
Q4	**-2.440 (-3.021, -1.859)**	**<0.001**	0.269 (-0.181, 0.718)	0.242	**-0.911 (-1.370, -0.452)**	**<0.001**

- (Supplementary Information, p. 22)

Supplementary Table 9. Sensitivity analyses of associations between wearable feature quartiles and accelerated biological aging.

Baseline						
**Quartile Groups**	**Amplitude Beta (95% CI)**	***p*-value**	**Acrophase Beta (95% CI)**	***p*-value**	**IS Beta (95% CI)**	***p*-value**
Q1	Reference		Reference		Reference	
Q2	**0.622 (0.484, 0.800)**	**<0.001**	1.176 (0.914, 1.514)	0.207	0.929 (0.725, 1.192)	0.564
Q3	**0.484 (0.375, 0.625)**	**<0.001**	1.061 (0.823, 1.367)	0.648	0.847 (0.658, 1.090)	0.196
Q4	**0.476 (0.368, 0.616)**	**<0.001**	**1.456 (1.132, 1.871)**	**0.003**	**0.690 (0.535, 0.890)**	**0.004**
**Follow up**						
**Quartile Groups**	**Amplitude OR (95% CI)**	***p*-value**	**Acrophase OR (95% CI)**	***p*-value**	**IS OR (95% CI)**	***p*-value**
Q1	Reference		Reference		Reference	
Q2	**0.447 (0.289, 0.690)**	**<0.001**	1.210 (0.790, 1.853)	0.380	0.701 (0.460, 1.066)	0.097
Q3	**0.472 (0.303, 0.733)**	**<0.001**	1.470 (0.959, 2.253)	0.077	0.658 (0.428, 1.012)	0.057
Q4	**0.339 (0.215, 0.532)**	**<0.001**	1.337 (0.870, 2.055)	0.185	0.742 (0.482, 1.142)	0.175
**Longitudinal**						
**Quartile Groups**	**Amplitude OR (95% CI)**	***p*-value**	**Acrophase OR (95% CI)**	***p*-value**	**IS OR (95% CI)**	***p*-value**
Q1	Reference		Reference		Reference	
Q2	**0.438 (0.308, 0.624)**	**<0.001**	1.296 (0.920, 1.825)	0.138	0.831 (0.611, 1.129)	0.236
Q3	**0.334 (0.229, 0.486)**	**<0.001**	1.021 (0.719, 1.450)	0.909	0.803 (0.584, 1.103)	0.175
Q4	**0.256 (0.171, 0.386)**	**<0.001**	**1.582 (1.127, 2.223)**	**0.008**	0.739 (0.527, 1.104)	0.079

In conclusion, these analyses demonstrate that the associations between circadian rhythm features and biological aging are consistent whether ΔAge is modeled continuously or dichotomized, and they are further supported by graded effects across quantiles. We used dichotomization primarily to enhance interpretability, but the underlying findings are robust to alternative modeling approaches. We have clarified this in the revised manuscript and included the additional analyses in the Supplementary Information. → (Methods, p. 31; Results, p. 12; Discussion, p. 14; Supplementary Tables 7-9)

- (Methods, p. 31) "Sixth, to evaluate the impact of outcome specification, we conducted sensitivity analyses modeling ΔAge as a continuous outcome and examining associations across quartiles of key circadian features."

- (Results, p. 12) "Additional sensitivity analyses yielded consistent results across multiple model specifications. Associations remained stable when modeling ΔAge as a continuous outcome and across quartiles of circadian features, demonstrating graded, dose-response relationships (**Supplementary Tables 7-9**)."

- (Discussion, p. 14) "Results were also consistent across outcome specifications. When modeling ΔAge as a continuous outcome, associations remained similar in

both direction and magnitude across baseline, follow-up, and longitudinal analyses. In addition, analyses across quartiles of circadian features showed dose-response patterns, indicating graded relationships rather than threshold effects."

* Lines 294-296 (and more generally) pluralizing female → females, male → males

Response 15, Reviewer 3: We have revised the manuscript to consistently use "males" and "females" when referring to groups of participants. These changes have been made throughout the manuscript.

- (Results, p. 6) "In females, differences remained stable with age, whereas in males they attenuated after 60. For L5 (mean activity during the least active hours), differences were more pronounced in males."

- (Results, p. 8) "Females showed more pronounced timing-related shifts (fPC2, fPC3), while males displayed distinct fPC4 separation, with fast-aging males exhibiting bimodal rhythms and increased nocturnal activity."

- (Results, p. 9) "Sex-stratified analyses (Supplementary Table 1) showed overall consistency, though timing effects were more pronounced in females, while rhythm intensity remained protective across both sexes."

- (Results, p. 11) "In males, higher fPC1, MESOR, amplitude, and M10 consistently reduced the odds of fast aging by 15-30%, whereas the effect sizes were larger in females, ranging from 22 to 40%. Increased rhythm regularity reduced the odds of accelerated aging by 15% in females, while showing no statistically significant effect in males. Similarly, delayed acrophase increased the odds of fast aging by 12% in males and 18% in females but was statistically significant only in females."

- (Discussion, p. 19) "For example, estrogen enhances pacemaker sensitivity and circadian amplitude, potentially accounting for the stronger phase-related effects observed in females. In addition, females generally exhibit more robust and stable circadian rhythms, whereas males more often display rhythm fragmentation and inconsistency[23] and lower sleep efficiency[24]. Under real-world conditions, males also tend to have later chronotypes[25], predisposing them to higher rates of social jetlag[25] and associated circadian disruption."

* "The cosinor model has been applied to analyze circadian patterns ..." (lines 417-420) — the reader (and this reviewer) would benefit from a bit more discussion about what these features (the CRAR and the MESOR) really capture. The other statistics — M10/L5, RA, IS,and IV — are discussed at a high level with good intuition conveyed (e.g., "A higher IS value indicates greater day-to-day regularity"). What does a higher MESOR really mean in this sense?

Response 16, Reviewer 3: Circadian rest-activity rhythm (CRAR) metrics quantify the 24-hour structure of behavioral activity patterns, capturing aspects of rhythm intensity, timing, regularity, and overall activity level. MESOR (Midline Estimating Statistic Of Rhythm) represents the rhythm-adjusted mean level of activity over the 24-hour cycle.

Importantly, MESOR captures a distinct aspect of rest-activity behavior compared to other circadian metrics such as amplitude and acrophase. MESOR reflects the "overall

activity level" of an individual, while amplitude reflects the "strength of oscillation" and acrophase captures the "timing of peak activity". Thus, a higher MESOR indicates greater mean activity level, whereas a lower MESOR reflects reduced mean activity level.

In our analysis, MESOR complements amplitude, timing, and regularity-based metrics, allowing separation of overall activity level from circadian rhythmic structure. We have added this clarification to the Methods section in the revised manuscript.→ (Methods, p. 25)

- (Methods, p. 25) "Circadian rest-activity rhythm (CRAR) metrics quantify the 24-hour structure of behavioral activity patterns, capturing aspects of rhythm intensity, timing, regularity, and overall activity level and were derived using parametric and non-parametric approaches[23,26,27] and data-driven functional principal component analysis[28]

  ….

  Specifically, a 24-hour single-component cosinor model was fitted to each individual's data to estimate the MESOR (Midline Estimating Statistic of Rhythm), amplitude (half the range of rhythmic variation within the cycle), and acrophase (timing of peak activity)[29]. MESOR reflects the rhythm-adjusted mean level of activity across the 24-hour cycle and can be interpreted as the overall activity level. Amplitude captures the strength of rhythmic oscillation, and acrophase reflects the timing of peak activity. Model fitting used an iterative least-squares procedure, assuming a fixed circadian period of 24 hours, consistent with prior work[12]."

* "Then we projected these trajectories onto a nine-term Fourier basis, ..." (line 452) — why do both a 9-term Fourier basis projection followed by only retaining the first four functional PCs? Is it possible that the initial Fourier smoothing removes relevant or predictive variability in the signal? What kinds of artifacts does this first Fourier stage jettison? Why retain only four PCs?

Response 17, Reviewer 3: We thank the reviewer for this valuable comment. The Fourier projection and subsequent fPCA serve distinct and complementary roles: signal regularization and data-driven feature extraction.

First, minute-level activity data were represented using a nine-term Fourier basis expansion, an established approach for capturing dominant circadian patterns while reducing high-frequency noise, with demonstrated robustness across wearable devices, datasets, and sampling frequencies[22,30–32]. This step enables smoothness and periodicity consistent with circadian biology. Raw wearable signals contain short-term fluctuations arising from behavioral irregularity, device noise, and wear artifacts, which do not reflect the underlying circadian structure. The Fourier projection suppresses these non-circadian components, ensuring that downstream analyses capture physiologically meaningful variation.

Second, fPCA is applied to the smoothed trajectories to identify dominant inter-individual variation. While the Fourier basis imposes biologically motivated structure, fPCA extracts data-driven orthogonal components representing amplitude, timing, and waveform characteristics. The use of nine Fourier terms reflects a bias-variance trade-off, providing sufficient flexibility to capture circadian waveform complexity without overfitting high-frequency noise.

Finally, we retained the first four functional principal components based on variance explained, stability, and interpretability. The first four components collectively explain approximately 90% of the total variance (fPC1: accounting for 51% of the variance, fPC2:

18%, fPC3: 13%, fPC4: 6%). Additional components contributed minimal incremental variance and did not improve model performance, supporting a parsimonious representation of circadian variation. We have clarified this rationale in the revised manuscript. → (Methods, p. 27)

- (Methods, p. 27) “We then projected these trajectories onto a nine-term Fourier basis expansion, consistent with prior studies that effectively captures diurnal periodicity with minimal overfitting[31,33]. This step reduces high-frequency variation prior to dimensionality reduction, allowing fPCA to capture dominant modes of variability rather than noise-driven fluctuations. Empirical evaluation of 7-10 harmonics demonstrated that harmonics beyond nine terms provided only marginal gains in variance explained while increasing model complexity. The resulting smoothed functions were subjected to fPCA, which decomposes the covariance matrix into an orthonormal basis of eigenfunctions that capture principal components modes of variation. We retained the first four functional principal components (fPCs), which jointly explained 88% of the total variance. Additional components contributed minimal incremental variance and did not improve model stability or interpretability, supporting a parsimonious representation of circadian variation.”

* Fig 1 — It’s difficult to suss out statistical artifacts from genuine cohort differences in these plots. Could they benefit by visualizing some notion of uncertainty, or perhaps the difference between two averages (with error bars)?

Response 18, Reviewer 3: To visualize age-related trajectories of circadian rhythm metrics, we used locally weighted scatterplot smoothing (LOWESS). To quantify uncertainty in the estimated trajectories, we constructed 95% confidence bands using a bootstrap resampling approach. Specifically, we repeatedly resampled (n=1000) the data with replacement and refit the LOWESS curve and then derived pointwise confidence intervals from the empirical distribution of the smoothed estimates. We have revised the manuscript to include these smoothed trajectories with corresponding confidence bands (**Fig. 1**) and have clarified this approach in the Methods section. → (Methods, p. 30; Fig. 1, p. 3)

- (Methods, p. 30) “Age-related trajectories of circadian rhythm metrics were visualized using locally weighted scatterplot smoothing (LOWESS). To quantify uncertainty, we constructed 95% confidence bands using bootstrap resampling (n=1000).”

- (Fig. 1, p. 3)

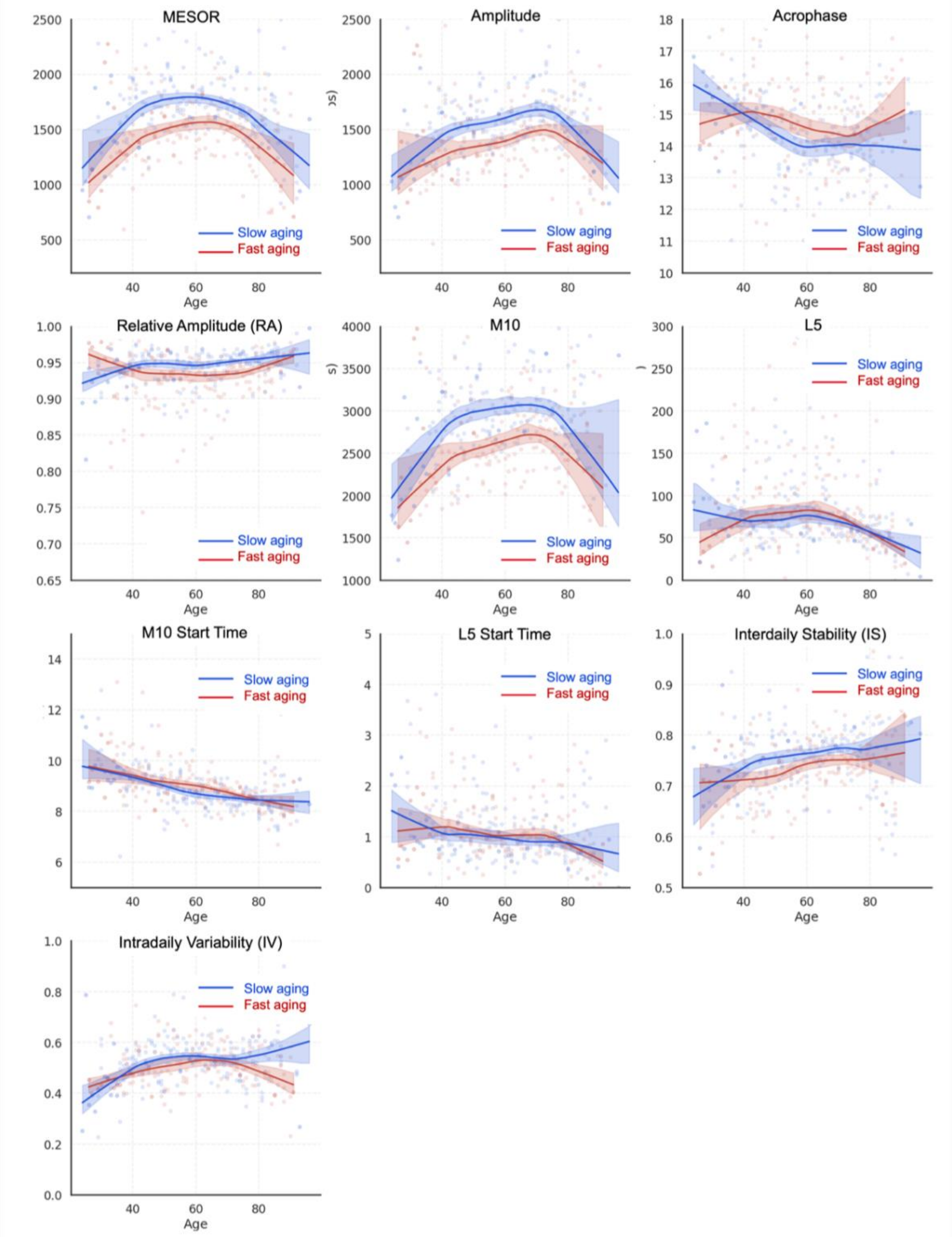

Fig. 1 Age-associated trends in circadian rest-activity rhythm (CRAR) metrics by biological age acceleration. Points are color-coded by biological aging status at baseline (blue=slow aging, red=fast aging). Locally weighted scatterplot smoothing (LOWESS) lines are overlaid to visualize smoothed trajectories by aging group. Shaded areas represent 95% confidence bands derived from 1000 bootstrap resampling.

* Fig 2(a) — what is the range depicted by the multiple curves? The extremes of the data distribution? Quartiles?

Response 19, Reviewer 3: The curves depict the mean trajectory (gray) and the primary modes of variation captured by each functional principal component (fPC). Specifically, the colored curves represent positive (++++) and negative (----) deviations along each fPC axis (i.e., mean ± scaled eigenfunctions). These curves illustrate the direction and magnitude of variation associated with each fPC, rather than representing specific percentiles or ranges of the observed data. We revised the Figure 2 caption to explicitly clarify this information. → (Fig. 2, p. 4)

- (Fig. 2 caption, p. 4) "**Fig. 2 Functional principal components (fPCs) of 24-hour activity profiles and their association with biological age acceleration.** (a) The mean 24-hour activity profile (gray) and variation along each functional principal component are shown. Colored curves represent positive (++++) and negative (----) deviations from the mean trajectory (i.e., mean ± scaled eigenfunctions), illustrating the dominant temporal patterns captured by each fPC. (b) Line plots depict group-level deviations from the population mean, computed as the mean fPC score multiplied by the corresponding eigenfunction for each biological aging group. These curves illustrate how circadian rest-activity patterns differ across fast aging vs. slow aging groups. fPC scores were standardized (mean=0, variance=1), and eigenfunctions were scaled to the range [-1, 1] for visualization."

* In the linear models used and presented, could there be any interactions between features? Could timing factors have different effect sizes given a fixed amplitude level? Similarly, could effects differ by chronological age?

Response 20, Reviewer 3: We thank the reviewer for this comment. We tested interaction terms across key circadian domains, including intensity, timing, and stability, as well as interactions between circadian metrics and chronological age (**Supplementary Table 11**). In general, we did not observe significant interaction effects. The estimated interaction terms were consistently close to the null with relatively narrow confidence intervals, suggesting limited evidence for effect modification. For example, the interaction between amplitude and acrophase (intensity*timing) yielded an OR of 0.997 (95% CI: 0.909-1.093; p=0.945), and the interaction between amplitude and age showed an OR of 0.999 (95% CI: 0.995-1.011; p=0.508). Similar patterns were observed across other interaction terms except IS*age.

- (Supplementary Information, p. 24)

Supplementary Table 11. Interaction effects between circadian metrics and chronological age.

Interaction Term	Domain	OR (95% CI)	*p*-value
Amplitude*Acrophase	Intensity*Timing	0.997 (0.909, 1.093)	0.945
Amplitude*IS	Intensity*Stability	0.910 (0.823, 1.005)	0.063
Acrophase*IS	Timing*Stability	1.039 (0.947, 1.139)	0.417
Amplitude*Age	Intensity*Age	0.999 (0.990, 1.008)	0.807
Acrophase*Age	Timing*Age	1.003 (0.995, 1.011)	0.508
IS*Age	Stability*Age	1.009 (1.001, 1.018)	0.024

These results support an approximately additive or weakly interacting structure among circadian domains and are consistent with the use of a parsimonious model without interaction terms. We have clarified these analyses in the manuscript and included the corresponding results in the Supplementary Information. → (Methods, p. 31; Results, p. 12; Discussion, p. 13; Supplementary Table 8).

- (Methods, p. 31) "Seventh, we evaluated interaction terms between circadian domains (e.g., intensity, timing, and stability) as well as between circadian metrics and chronological age."

- (Results, p. 12) "Interaction analyses showed limited evidence for effect modification across circadian domains or with chronological age, with most interaction terms close to the null (**Supplementary Tables 11-12**)."

- (Discussion, p. 14) "We found limited evidence for interaction effects across circadian domains or with age. Effect estimates were generally close to the null, suggesting that these features contribute largely independently, or with only weak interaction, to biological aging. These findings support a parsimonious modeling approach. Furthermore, adjustment for physical activity and sleep regularity led to only partial attenuation of effect estimates with substantial associations remaining for circadian measures."

* It seems like the most durable/robust findings of these analyses is that intensity of activity is associated with slower PhenoAge. How much of this effect could be explained by the fact that "exercise is protective" in general?

Response 21, Reviewer 3: We thank the reviewer for this important point. Physical activity is a well-established protective factor for health. However, conventional exercise metrics, such as moderate-to-vigorous physical activity (MVPA) minutes, are scalar summaries of activity volume and do not capture the temporal organization, stability, or full 24-hour structure of rest-activity rhythms. In contrast, our approach leverages data-driven features derived from the entire activity profile, capturing aspects of behavioral organization beyond total activity volume.

To assess whether our findings are explained by overall activity volume, we conducted additional analyses adjusting for average daily MVPA minutes derived from smartwatch data. MVPA minutes were defined as the average daily duration of fairly and very active minutes collected from Fitbit smartwatch. Attenuation was quantified as the relative change in regression coefficients after adjustment for MVPA, calculated as $(\widehat{\beta_1}-\widehat{\beta_2})/\widehat{\beta_1}\times100$, where $\widehat{\beta_1}$ and $\widehat{\beta_2}$ denote estimated coefficients from models without and with MVPA adjustment, respectively. This metric reflects the proportion of the original association explained by MVPA (**Supplementary Table 13**).

- (Supplementary Information, p. 26)

Supplementary Table 13. Associations of circadian rhythm metrics with biological aging before and after adjustment for moderate-to-vigorous physical activity (MVPA).

Features	Unadjusted ($\widehat{\beta_1}$)	MVPA-adjusted ($\widehat{\beta_2}$)	Attenuation (%)
fPC1	-0.184	-0.134	27.2

fPC2	0.057	0.052	8.8
fPC3	-0.064	-0.038	40.6
fPC4	0.016	0.014	12.5
MESOR	-0.424	-0.450	-6.1
Amplitude	-0.473	-0.472	0.2
Acrophase	0.140	0.134	4.3
RA	-0.269	-0.274	-1.9
M10	-0.491	-0.525	-6.9
L5	0.054	0.068	-25.9
M10 start	0.125	0.083	33.6
L5 start	0.051	0.057	-11.8
IS	-0.111	-0.094	15.3
IV	-0.115	-0.088	23.5

Adjustment for MVPA led to partial attenuation of the association for activity intensity (fPC1) ($\widehat{\beta_1}$=−0.184 vs $\widehat{\beta_2}$=−0.134; attenuation = 27.2%), indicating that activity volume explains part of the observed relationship. However, minimal attenuation was observed for amplitude and relative amplitude (RA), suggesting that circadian intensity captures behavioral dimensions beyond exercise duration and is largely independent of overall activity volume. Timing- and stability-related features, such as M10 start time, interdaily stability (IS), and intradaily variability (IV), showed some levels of attenuation, indicating partial overlap with activity patterns captured by MVPA.

Interestingly, several features (e.g., MESOR, M10, L5, and L5 start time) exhibited negative attenuation, with stronger effect sizes after adjustment. This pattern is consistent with suppression, suggesting that activity volume may partially mask underlying associations between circadian organization and biological aging.

To summarize, these findings indicate that circadian features derived from the 24-hour activity profile, including intensity, timing, and regularity, provide additional and complementary information beyond scalar measures of exercise volume. We have revised the manuscript to include these additional analyses. → (Results, p. 12; Discussion, p. 14)

- (Results, p. 12) "Furthermore, adjustment for physical activity such as moderate-to-vigorous activity (MVPA) minutes and sleep-related metrics resulted in only partial attenuation of effect estimates, with substantial associations remaining for circadian measures (**Supplementary Tables 13-14**)."

- (Discussion, p. 14) "Furthermore, adjustment for physical activity and sleep regularity led to only partial attenuation of effect estimates with substantial associations remaining for circadian measures."

* A key differentiating factor of this study is the deep dive into rhythmic factors (like timing, relative amplitude, acrophase, stability). However those seem to be less reliable or variable from analysis to analysis.

Response 22, Reviewer 3: We thank the reviewer for this important observation regarding the variability of rhythm-based features. This can be explained by several factors.

1. **Differential effect size across circadian metrics:** Timing (e.g., acrophase) and stability (e.g., IS) often have smaller effect sizes and are therefore more sensitive to covariate adjustment, sample composition, and model specification. In contrast, intensity measures (e.g., amplitude) capture overall circadian signal strength and tend to show stronger and more monotonic associations.

2. **Non-linear and heterogeneous associations:** Our findings suggest that some of the associations are non-linear and heterogeneous across the distribution of circadian features. As shown in **Table R3,** acrophase demonstrates stronger associations at the extreme quartiles, rather than a monotonic gradient. This indicates that some rhythm features operate through threshold-dependent or non-linear mechanisms, rather than uniform linear effects.

Table R3 (excerpt from Supplementary Table 9). Sensitivity analyses of associations between acrophase and accelerated biological aging.

	Acrophase					
**Quartile Groups**	**Baseline OR (95% CI)**	***p*-value**	**Follow up OR (95% CI)**	***p*-value**	**Longitudinal OR (95% CI)**	***p*-value**
Q1	Reference		Reference		Reference	
Q2	1.176 (0.914, 1.514)	0.207	1.210 (0.790, 1.853)	0.380	1.296 (0.920, 1.825)	0.138
Q3	1.061 (0.823, 1.367)	0.648	1.470 (0.959, 2.253)	0.077	1.021 (0.719, 1.450)	0.909
Q4	**1.456 (1.132, 1.871)**	**0.003**	1.337 (0.870, 2.055)	0.185	**1.582 (1.127, 2.223)**	**0.008**

3. **Consistency with prior literature:** This observation is consistent with prior literature. Previous cross-sectional studies based on short-term (e.g., ~1-week) actigraphy have shown that intensity-related metrics (e.g., relative amplitude) tend to exhibit more consistent associations with outcomes such as mortality, disease incidence, and systemic inflammation, whereas timing- and regularity-related features often show weaker or less consistent linear associations[34,35]. This suggests that these features may be sensitive to contextual and behavioral variability, particularly in real-world settings.

4. **Amplification in real-world longitudinal data:** This variability is further amplified in real-world, free-living longitudinal data, such as in our study. Unlike controlled or short-term studies, our data capture day-to-day behavioral fluctuations, environmental influences, and adherence variability over extended periods. As timing and regularity metrics are inherently influenced by external constraints (e.g., work schedules, social timing, environmental factors), their associations with health outcomes are expected to show greater heterogeneity across analyses in such settings. At the same time, this variability reflects behavior in real-world, free-living settings, thereby enhancing the external validity and generalizability of our findings.

5. **Robustness across sensitivity analyses:** Importantly, despite this variability, we observed **consistent effect directions across models and sensitivity analyses**, including longitudinal models, alternative parameterizations of predictors and outcomes

(continuous vs. categorical), effect modification analyses, adjustment for MVPA, health utilization, the length of follow-up, with consistent effect directions observed throughout.

In summary, the variability of timing and regularity features across analyses likely reflects their smaller effect sizes, non-linear and threshold-dependent relationships, and greater sensitivity to behavioral and contextual factors. These patterns are consistent with prior literature and are expected in real-world, free-living longitudinal settings, rather than indicating a lack of reliability. We have revised the manuscript to clarify this interpretation and have included additional sensitivity analyses and detailed results in the Discussion section. → (Discussion, p. 14)

- (Discussion, p. 14) "Timing- and regularity-related features (e.g., acrophase, IS) exhibited greater variability across models compared to intensity-related metrics (e.g., amplitude, RA). This likely reflects their smaller effect sizes and greater sensitivity to model parameterization and behavioral context. In addition, these features tend to show non-linear and heterogeneous relationships across their distributions, with effects more pronounced at the extremes. Such variability is further expected in real-world, free-living data, where timing and regularity are likely to be influenced by external constraints (e.g., work schedules, social timing, environmental factors). Despite this variability, effect directions remained consistent across analyses, supporting the robustness of the overall findings. This variability also enhances the external validity and generalizability of the results by reflecting behavior under real-world conditions."

* Furthermore, could a "strong circadian rhythm" (e.g., measured by MESOR, if i'm not mistaken) also pick up on regularity in sleep patterns? To what extent are we seeing evidence of the benefits of exercise and a predictable sleep schedule?

Response 23, Reviewer 3: We thank the reviewer for this important question. To address this question, we quantified sleep regularity using two complementary measures: variability in sleep duration and variability in sleep onset timing. For both metrics, we focused on the wearable-derived main sleep episode. Sleep duration was computed as total minutes asleep per day, and sleep onset was defined as the start time of the main sleep episode. We then quantified variability across days by calculating the standard deviation of daily sleep duration and the sleep onset timing. Using these definitions, we find that circadian rhythm metrics are only partially correlated with sleep regularity and physical activity, indicating that they capture related but largely distinct dimensions of behavior. This is supported by three complementary lines of evidence.

(1) **Correlation between circadian and sleep measures:** The correlation analyses demonstrate that circadian (amplitude, acrophase, interdaily stability (IS)), exercise (moderate-to-vigorous physical activity (MVPA)), and sleep features (sleep duration variability, sleep onset variability) capture related but distinct dimensions of behavior (**Fig. R1**). In this plot, amplitude showed moderate correlation with MVPA (r=0.62) and IS (r=0.38). However, correlations between amplitude and sleep regularity measures, both sleep duration variability and sleep onset variability, were weak (r= -0.14 to -0.19). This indicates that circadian rhythm metrics are not redundant with sleep regularity or physical activity but instead reflect complementary behavioral dimensions.

Fig. R1 Correlation and partial correlation structure of circadian rhythm, physical activity, and sleep regularity metrics.

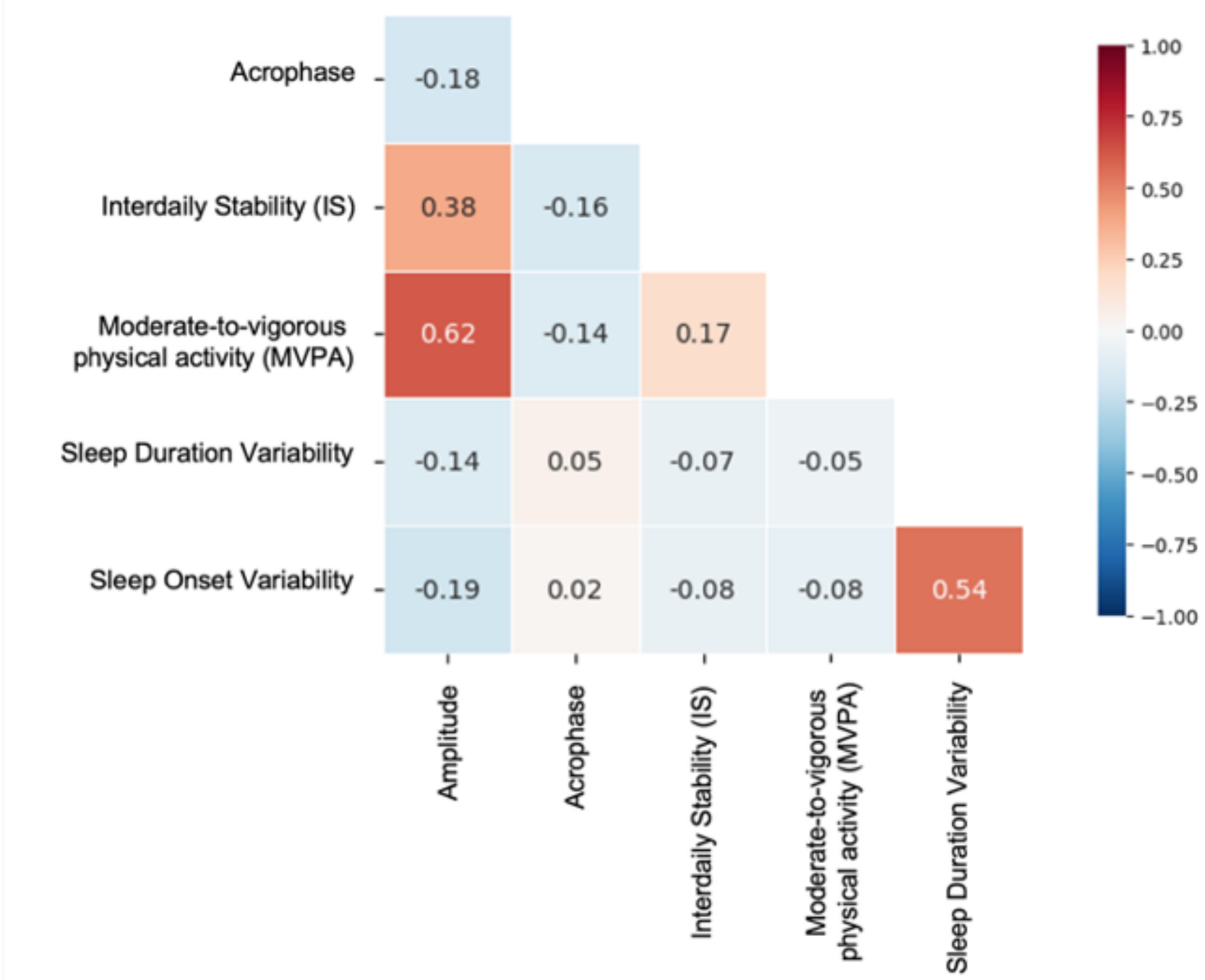

(2) Interaction between circadian rhythm and sleep regularity: In statistical models, we found no evidence of interaction between circadian rhythm measures and sleep regularity (**Table R4**). Specifically, interaction terms between amplitude and both sleep duration variability and sleep onset variability were not statistically significant. This suggests that the association between circadian rhythm strength and accelerated aging is not modified by sleep regularity.

Table R4. Interaction effects between circadian domains and sleep regularity.

Interaction Term	OR (95% CI)	*p*-value
Amplitude*Sleep Duration Variability	1.049 (0.950, 1.158)	0.345
Amplitude*Sleep Onset Variability	0.946 (0.869, 1.030)	0.203

(3) Adjusting for both physical activity and sleep regularity: The associations of circadian rhythm metrics with biological aging remained robust after jointly adjusting for both physical activity (MVPA) and sleep regularity (**Supplementary Table 14**). Effect estimates were only modestly attenuated for intensity-related features; for example, amplitude showed minimal attenuation (~4.4%). Greater attenuation was observed for certain timing- and fragmentation-related metrics, including acrophase (~16.4%), M10 start (~38.4%), L5 start (~31.4%), intradaily variability (IV; ~23.5%), and fPC4 (~43.8%), which captures biphasic activity patterns. These results indicate that while core circadian amplitude and overall rhythm strength remain largely robust and independent of physical activity and sleep regularity, aspects related to timing and rhythm fragmentation may be partially explained by sleep-related behavioral patterns. Nevertheless, the overall consistency of effect supports the robustness of the primary findings.

- (Supplementary Information, p. 27)

Supplementary Table 14. Associations of circadian rhythm metrics with biological aging before and after adjustment for MVPA and sleep regularity.

Features	Unadjusted ($\widehat{\beta_1}$)	MVPA- and sleep-adjusted ($\widehat{\beta_2}$)	Attenuation (%)
fPC1	-0.184	-0.157	14.7
fPC2	0.057	0.052	8.8
fPC3	-0.064	-0.064	0.0
fPC4	0.016	0.009	43.8
MESOR	-0.424	-0.459	-8.3
Amplitude	-0.473	-0.452	4.4
Acrophase	0.140	0.117	16.4
RA	-0.269	-0.301	-11.9
M10	-0.491	-0.542	-10.4
L5	0.054	0.096	-77.8
M10 start	0.125	0.077	38.4
L5 start	0.051	0.035	31.4
IS	-0.111	-0.118	-6.3
IV	-0.115	-0.088	23.5

In summary, core measures of circadian rhythm strength remain largely robust after adjustment for physical activity and sleep regularity, whereas certain timing- and fragmentation-related features show partial overlap with sleep-related behavioral patterns. Overall, these metrics capture a broader dimension of 24-hour behavioral organization, integrating activity intensity, temporal structure, and rhythm robustness, providing information beyond physical activity and sleep regularity in relation to biological aging. We have revised our manuscript to clarify this interpretation in the Methods and Discussion and have included additional details in the Supplementary Information. → (Methods, p. 27; Results, p. 12; Discussion, p. 21; Supplementary Table 14)

- (Methods, p. 27) "**Assessment of sleep metrics**
  Sleep metrics were derived from Fitbit daily summary and sequence-level sleep data. Sleep episodes were reconstructed from timestamped sleep stage records by ordering consecutive segments within each participant and defining distinct episodes using gaps of ≥30 minutes. Main sleep episodes were identified using records with `is_main_sleep = TRUE`, while non-main sleep records (`is_main_sleep = FALSE`) were used to characterize daytime napping behavior. For each episode, sleep onset was defined as the earliest start time and wake time as the latest end time, and sleep duration was calculated as the elapsed time between onset and wake. Sleep midpoint was defined as the temporal midpoint between sleep onset and wake time.

  From the main sleep episodes, we derived total sleep duration, sleep onset time, sleep end time, sleep midpoint, and sleep efficiency, calculated as the ratio of

minutes asleep to minutes in bed. Daytime sleep (naps) was identified from non-main sleep episodes using the same episode definition. For each participant, we derived nap count, average nap duration, and mean nap onset time. All sleep metrics were first computed at the daily level and subsequently aggregated to the participant-year level by averaging across valid days. These sleep-derived measures were used to evaluate correspondence between activity-derived circadian patterns and independently measured sleep behaviors."

- (Results, p. 12) "Furthermore, adjustment for physical activity such as moderate-to-vigorous activity (MVPA) minutes and sleep-related metrics resulted in only partial attenuation of effect estimates, with substantial associations remaining for circadian measures (**Supplementary Tables 13-14**)."

- (Discussion, p. 21) "We also found that the associations of circadian metrics remained robust across extensive sensitivity analyses, including adjustment for physical activity, sleep regularity, health utilization, and the length of follow-up, as well as across different imputation methods. These findings support the robustness of circadian metrics as indicators of biological aging trajectories."

Bibliography

1. Levine, M. E. Modeling the Rate of Senescence: Can Estimated Biological Age Predict Mortality More Accurately Than Chronological Age? *The Journals of Gerontology Series A: Biological Sciences and Medical Sciences* **68**, 667–674 (2013).
2. Levine, M. E. *et al.* An epigenetic biomarker of aging for lifespan and healthspan. *Aging (Albany NY)* **10**, 573–591 (2018).
3. Mak, J. K. L. *et al.* Clinical biomarker-based biological aging and risk of cancer in the UK Biobank. *Br J Cancer* **129**, 94–103 (2023).
4. Kwon, D. & Belsky, D. W. A toolkit for quantification of biological age from blood chemistry and organ function test data: BioAge. *GeroScience* **43**, 2795–2808 (2021).
5. Zheng, J., Liu, T., Luo, Y. & Wei, X. Development and validation of a modified phenoage for biological aging and chronic diseases in a chinese cohort. *Archives of Gerontology and Geriatrics* **142**, 106114 (2026).
6. Gao, Y. *et al.* Association between phenotypic age and in-hospital outcomes in patients with acute myocardial infarction: A retrospective observational study. *Int J Cardiol Heart Vasc* **58**, 101670 (2025).
7. Li, X. *et al.* Longitudinal trajectories, correlations and mortality associations of nine biological ages across 20-years follow-up. *eLife* **9**, e51507 (2020).
8. Kuo, P.-L. *et al.* Longitudinal phenotypic aging metrics in the Baltimore Longitudinal Study of Aging. *Nat Aging* **2**, 635–643 (2022).
9. Murabito, J. M. *et al.* Measures of Biologic Age in a Community Sample Predict Mortality and Age-Related Disease: The Framingham Offspring Study. *J Gerontol A Biol Sci Med Sci* **73**, 757–762 (2018).
10. Kwan, K. J. S. *et al.* Evaluating the potential of phenotypic age to enhance cardiovascular risk prediction over chronological age in the UK Biobank. *Sci Rep* **15**, 27858 (2025).
11. Xu, Y. *et al.* Blunted rest-activity circadian rhythm is associated with increased rate of biological aging: an analysis of NHANES 2011-2014. *J Gerontol A Biol Sci Med Sci* glac199 (2022) doi:10.1093/gerona/glac199.
12. Shim, J., Fleisch, E. & Barata, F. Circadian rhythm analysis using wearable-based accelerometry as a digital biomarker of aging and healthspan. *npj Digit. Med.* **7**, 1–14 (2024).
13. Glasheen, W. P. *et al.* Charlson Comorbidity Index: ICD-9 Update and ICD-10 Translation. *Am Health Drug Benefits* **12**, 188–197 (2019).
14. Lopes De Oliveira, T. *et al.* Effects from medications on functional biomarkers of aging in three longitudinal studies of aging in Sweden. *Aging Cell* **23**, e14132 (2024).
15. Liu, Z. *et al.* A new aging measure captures morbidity and mortality risk across diverse subpopulations from NHANES IV: A cohort study. *PLoS Med* **15**, e1002718 (2018).
16. Krivonosov, M. I. *et al.* A new cognitive clock matching phenotypic and epigenetic ages. *Transl Psychiatry* **12**, 364 (2022).
17. Kuo, C.-L., Pilling, L. C., Liu, Z., Atkins, J. L. & Levine, M. E. Genetic associations for two biological age measures point to distinct aging phenotypes. *Aging Cell* **20**, e13376 (2021).
18. Han, W. M. *et al.* Association of Phenotypic Aging Marker with comorbidities, frailty and inflammatory markers in people living with HIV. *BMC Geriatr* **22**, 1010 (2022).
19. The All of Us Research Program Investigator. The “All of Us” Research Program. *New England Journal of Medicine* **381**, 668–676 (2019).
20. Bailey, C. P. *et al.* Fitbit Physical Activity and Sleep Data in the All of Us Research Program: Data Exploration and Processing Considerations for Research. *Med Sci Sports Exerc* **57**, 2946–2953 (2025).
21. Master, H. *et al.* Association of step counts over time with the risk of chronic disease in the All of Us Research Program. *Nat Med* **28**, 2301–2308 (2022).
22. Erickson, M. L. *et al.* Age Is Associated With Dampened Circadian Patterns of Rest and Activity: The Study of Muscle, Mobility, and Aging (SOMMA). *J Gerontol A Biol Sci Med Sci* **79**, glae049 (2024).

23. Li, J., Somers, V. K., Lopez-Jimenez, F., Di, J. & Covassin, N. Demographic characteristics associated with circadian rest-activity rhythm patterns: a cross-sectional study. *Int J Behav Nutr Phys Act* **18**, 107 (2021).
24. Su, S., Li, X., Xu, Y., McCall, W. V. & Wang, X. Epidemiology of accelerometer-based sleep parameters in US school-aged children and adults: NHANES 2011–2014. *Sci Rep* **12**, 7680 (2022).
25. Roenneberg, T., Pilz, L. K., Zerbini, G. & Winnebeck, E. C. Chronotype and Social Jetlag: A (Self-) Critical Review. *Biology* **8**, 54 (2019).
26. Cai, R. *et al.* Circadian disturbances and frailty risk in older adults. *Nat Commun* **14**, 7219 (2023).
27. Neikrug, A. B. *et al.* Characterizing Behavioral Activity Rhythms in Older Adults Using Actigraphy. *Sensors (Basel)* **20**, 549 (2020).
28. Crainiceanu, C. M., Goldsmith, J., Leroux, A. & Cui, E. *Functional Data Analysis with R*. (Chapman and Hall/CRC, New York, 2024). doi:10.1201/9781003278726.
29. Cornelissen, G. Cosinor-based rhythmometry. *Theor Biol Med Model* **11**, 16 (2014).
30. Zeitzer, J. M. *et al.* Phenotyping Apathy in Individuals With Alzheimer Disease Using Functional Principal Component Analysis. *Am J Geriatr Psychiatry* **21**, 391–397 (2013).
31. Zeitzer, J. M. *et al.* Daily Patterns of Accelerometer Activity Predict Changes in Sleep, Cognition, and Mortality in Older Men. *J Gerontol A Biol Sci Med Sci* **73**, 682–687 (2018).
32. Winer, J. R. *et al.* Impaired 24-h activity patterns are associated with an increased risk of Alzheimer's disease, Parkinson's disease, and cognitive decline. *Alzheimers Res Ther* **16**, 35 (2024).
33. Shi, B., Wei, P. & Huang, X. Functional principal component based landmark analysis for the effects of longitudinal cholesterol profiles on the risk of coronary heart disease. *Stat Med* **40**, 650–667 (2021).
34. Xu, Y., Su, S., McCall, W. V. & Wang, X. Blunted rest-activity rhythm is associated with increased white blood-cell-based inflammatory markers in adults: an analysis from NHANES 2011-2014. *Chronobiology International* **39**, 895–902 (2022).
35. Xu, Y. *et al.* Blunted rest-activity circadian rhythm increases the risk of all-cause, cardiovascular disease and cancer mortality in US adults. *Sci Rep* **12**, 20665 (2022).

We thank the editor and reviewers for their careful assessment of our revised manuscript. We have included all modifications made in response to the reviewers' feedback and addressed each comment individually. Reviewer comments are presented in blue, and our responses in black.

Reviewer #1

The authors have done an excellent job of addressing my concerns in a remarkably thorough response. I appreciate the effort to temper causal language and provide more transparency about the study limitations. I have no further comments.

Response, Reviewer 1: We thank the reviewer for the careful reading and constructive feedback. No additional manuscript changes were requested.

Reviewer #2

The authors have addressed my concerns. Minor edit error: The Table 3 is missing OR values.

Response, Reviewer 2: We thank the reviewer for noting this issue. We have checked Table 3 and confirmed that all OR values and 95% confidence intervals are displayed correctly in the revised manuscript.

Reviewer #3

Thank you to the authors for their thorough and substantive responses to the reviews. The revisions address many of my concerns, and the additional (many) sensitivity analyses assuage concerns about robustness and generalizability of the effect; I genuinely appreciate your taking the time to conduct these analyses and curate this evidence. A few points remain where I'd appreciate further clarification or refinement.

Response, Reviewer 3: We thank the reviewer for the constructive assessment and for identifying the remaining points requiring clarification. We revised the manuscript to address these points in three ways. First, we clarified the evidence base for using repeated PhenoAge longitudinally and described the limitations of interpreting ΔPhenoAge as a longitudinal endpoint. Second, we further softened causal and promotional wording throughout the manuscript. Third, we revised the PLS/fPCA text to avoid implying that either basis is more fundamental and describe them as complementary rotations of the same underlying functional variation optimized under different criteria.

1. PhenoAge as a longitudinal endpoint (Response 3):

I appreciate the additional context on PhenoAge and the references provided. My remaining concern is specifically about ΔPhenoAge as a longitudinal endpoint. The cited Kuo et al. study, as I read it, developed a new phenotypic aging metric explicitly designed to be longitudinally meaningful — which, if my interpretation is correct, doesn't directly speak to the longitudinal utility of the original PhenoAge construct (the specific nine-biomarker combination used here). I don't doubt that longitudinal changes in PhenoAge can be predictive of health outcomes, but it would strengthen the manuscript to cite evidence supporting that claim directly, rather than via Kuo. As written, the citation risks being read as stronger support than it actually provides.

To be clear: I'm not arguing against PhenoAge as an endpoint — it's a reasonable choice. I would just like the evidence base for its longitudinal use, and its limitations in that setting, to

be presented transparently. No endpoint is perfect, and acknowledging that explicitly would strengthen rather than weaken the paper.

Response 1, Reviewer 3: We thank the reviewer for this important clarification. In response, we have made two key revisions.

First, we revised the framing of Kuo et al. We now cite this study as broader contextual support for longitudinal phenotypic aging metrics rather than as direct validation of within-person change in the original PhenoAge construct. To provide more directly relevant longitudinal evidence, we added Lin 2026, a large Taiwan Biobank study of approximately 69,000 participants with a mean follow-up of 4.9 years that evaluated within-individual changes in PhenoAge-derived measures in relation to modifiable lifestyle and environmental factors[1]. Because Lin 2026 used a six-marker PhenoAge-derived estimate due to biomarker availability rather than the exact original construct, we cite it as additional longitudinal evidence rather than as definitive validation of our endpoint. It is to note that the six-marker estimate showed high correlation with the original PhenoAge construct in NHANES III, with Pearson correlations of 0.960 in men and 0.936 in women.

Second, we added explicit language acknowledging the potential limitations of ΔPhenoAge in a longitudinal setting, noting that changes in PhenoAge may reflect true physiological change, but may also be affected by short-term within-person biomarker variability, EHR measurement timing, and unmeasured confounding. We also clarified that the observational design supports associations, not causal inference.

We have incorporated these changes in the Introduction and Discussion sections. → (Introduction, p. 4; Discussion, p. 20).

- (Introduction, p. 4) “It has been validated across multi-ethnic cohorts to predict disease, physical functioning, cognitive performance, and mortality, and longitudinal changes have also been reported to provide prognostic information beyond baseline levels[2–7]. Further supporting the use of PhenoAge as a longitudinal phenotypic marker, recent evidence demonstrates that within-individual changes in a PhenoAge estimate derived from a reduced biomarker set are systematically associated with modifiable lifestyle and environmental factors[1].”

- (Discussion, p. 20) “We interpret ΔPhenoAge as a longitudinal phenotypic marker of physiological aging trajectories, rather than as a definitive measure of biological aging rate. Changes in PhenoAge may reflect true physiological change, but may also be affected by short-term within-person biomarker variability, EHR measurement timing, and unmeasured confounding. Given the observational design of this study, our findings should be interpreted as associations rather than causal effects.”

2. Further, in response 2, your proposed text reads “Our findings provide among the first large-scale longitudinal evidence that rest-activity rhythm shapes trajectories of biological aging...” Again I would temper such a causal claim, and say that rest-activity rhythm associates with (or even robustly associates with, as you’ve shown) trajectories of biological aging.

Response 2, Reviewer 3: Thank you for this suggestion. We revised the manuscript to avoid causal wording and now describe the findings as associations. Specifically, we removed language stating that rest-activity rhythms "shape" biological aging trajectories and replaced it with wording stating that rest-activity rhythms are associated with biological aging trajectories. We also removed novelty-oriented wording such as "among the first" from the

quoted text and softened statements that could imply causality or established clinical utility.
→ (Abstract, p. 2; Introduction, p. 4; Results, p. 11; Discussion, p. 13-20)

- (Abstract, p. 2) "Disrupted rest-activity rhythms have been associated with aging and chronic disease, yet longitudinal evidence from free-living populations has been lacking."

- (Introduction, p. 4) "Our findings provide large-scale longitudinal evidence that wearable-derived rest-activity rhythms are robustly associated with trajectories of biological aging and support the potential utility of rest-activity measures as scalable and accessible digital biomarkers of biological aging trajectories."

- (Results, p. 11) "Together, the longitudinal analyses extend the cross-sectional findings by showing that within-individual changes in CRAR are longitudinally associated with changes in biological aging.

- (Discussion, p. 13) "… we demonstrated that circadian rest-activity rhythms were robustly associated with biological aging trajectories, assessed using clinical biomarker-based PhenoAge."

- (Discussion, p. 13) "… these findings move beyond single time-point associations and support the potential utility of consumer wearable-derived rest-activity rhythms as digital biomarkers of biological aging."

- (Discussion, p. 16) "In our cohort, greater regularity was associated with lower odds of accelerated aging in females…"

- (Discussion, p. 17) "… our study advances prior cross-sectional evidence and supports a longitudinal association of rest-activity rhythm patterns and aging trajectories."

- (Discussion, p. 19) "Our dual-domain framework, highlighting rhythm intensity as a broadly consistent marker associated with lower odds of accelerated aging …"

- (Discussion, p. 20) "At the population level, future intervention studies could test whether strategies that enhance rhythm intensity, such as structured physical activity programs, are associated with slower biological aging trajectories."

3. PLS vs. fPCA (Figure 2a):

I take the authors' point that the PLS and fPCA bases overlap substantially, which is largely tautological given that they are different rotations of the same underlying variation. My original question was about interpreting the bases themselves rather than the overlap (as in Figure 2). The framing that PLS "redistributes existing fPCA-derived variation" reads as though one analysis precedes the other in a meaningful way, but I don't think that's quite right — neither basis is more fundamental, they are simply different rotations chosen under different criteria.

Response 3, Reviewer 3: We thank the reviewer for clarifying this point. We revised both the Results and Discussion to avoid hierarchical language implying that either basis is more fundamental. We now describe fPCA and PLS as complementary rotations of the same underlying functional variation, optimized under different criteria: variance explained for fPCA and covariance with the outcome for PLS. We present fPCA as the primary representation because it is outcome-agnostic and interpretable, not because it is more fundamental than PLS. → (Results, p. 12; Discussion, p. 17)

- (Results, p. 12) "Supervised approaches based on partial least squares (PLS) components were closely aligned with fPCs, consistent with both methods capturing overlapping aspects of the same underlying functional variation under different optimization criteria (**Supplementary Fig. 9; Supplementary Table 17**). PLS1 was strongly correlated with fPC1 (r=0.96), and PLS2 was strongly correlated with fPC3 (r=-0.84), indicating overlap with activity-intensity and timing-related variation. PLS3 was correlated with both fPC2 (r=0.53) and fPC4 (r=-0.65), while PLS4 showed weaker and more distributed correlations across fPCs. These results support the use of PLS as a complementary supervised sensitivity analysis and fPCA as an outcome-agnostic representation of the major modes of rest-activity variation."

- (Discussion, p.17) "Partial least squares (PLS) and functional principal component analysis (fPCA) yielded highly concordant low-dimensional representations of rest-activity trajectories. Both methods form linear combinations of the same underlying functional variation but optimize different criteria: variance explained for fPCA and covariance with the outcome for PLS. We present fPCA as the primary representation in this study because it is outcome-agnostic and provides an orthogonal decomposition of the functional trajectories, with components that are mutually orthogonal in function space (i.e., with zero inner products over time), thereby facilitating interpretation and subsequent analyses."

References

1. Lin, W.-Y. Modifiable lifestyle factors and 4.9-Year changes in phenotypic age in the Taiwan biobank. *Front. Aging* **7**, (2026).
2. Li, X. *et al.* Longitudinal trajectories, correlations and mortality associations of nine biological ages across 20-years follow-up. *eLife* **9**, e51507 (2020).
3. Kuo, P.-L. *et al.* Longitudinal phenotypic aging metrics in the Baltimore Longitudinal Study of Aging. *Nat Aging* **2**, 635–643 (2022).
4. Levine, M. E. Modeling the Rate of Senescence: Can Estimated Biological Age Predict Mortality More Accurately Than Chronological Age? *The Journals of Gerontology Series A: Biological Sciences and Medical Sciences* **68**, 667–674 (2013).
5. Murabito, J. M. *et al.* Measures of Biologic Age in a Community Sample Predict Mortality and Age-Related Disease: The Framingham Offspring Study. *J Gerontol A Biol Sci Med Sci* **73**, 757–762 (2018).
6. Mak, J. K. L. *et al.* Clinical biomarker-based biological aging and risk of cancer in the UK Biobank. *Br J Cancer* **129**, 94–103 (2023).
7. Kwan, K. J. S. *et al.* Evaluating the potential of phenotypic age to enhance cardiovascular risk prediction over chronological age in the UK Biobank. *Sci Rep* **15**, 27858 (2025).
